# Efficient Dimerization Disruption of *Leishmania infantum* Trypanothione Reductase by Triazole-phenyl-thiazoles

**DOI:** 10.1021/acs.jmedchem.1c00206

**Published:** 2021-05-04

**Authors:** Alejandro Revuelto, Héctor de Lucio, Juan Carlos García-Soriano, Pedro A. Sánchez-Murcia, Federico Gago, Antonio Jiménez-Ruiz, María-José Camarasa, Sonsoles Velázquez

**Affiliations:** † Instituto de Química Médica (IQM-CSIC), c/ Juan de la Cierva 3, E-28006 Madrid, Spain; ‡ Departamento de Biología de Sistemas, Universidad de Alcalá, E-28805 Alcalá de Henares, Madrid, Spain; § Área de Farmacología, Departamento de Ciencias Biomédicas, Unidad Asociada al IQM-CSIC, Universidad de Alcalá, E-28805 Alcalá de Henares, Madrid, Spain

## Abstract

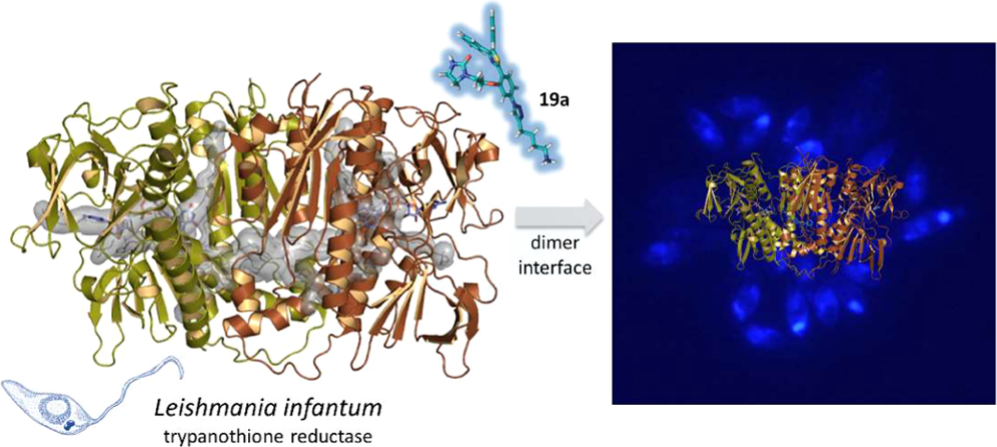

Inhibition of *Leishmania infantum* trypanothione disulfide reductase (*Li*TryR) by disruption of its homodimeric interface has proved to be an alternative and unexploited strategy in the search for novel antileishmanial agents. Proof of concept was first obtained by peptides and peptidomimetics. Building on previously reported dimerization disruptors containing an imidazole-phenyl-thiazole scaffold, we now report a new 1,2,3-triazole-based chemotype that yields noncompetitive, slow-binding inhibitors of *Li*TryR. Several compounds bearing (poly)aromatic substituents dramatically improve the ability to disrupt *Li*TryR dimerization relative to reference imidazoles. Molecular modeling studies identified an almost unexplored hydrophobic region at the interfacial domain as the putative binding site for these compounds. A subsequent structure-based design led to a symmetrical triazole analogue that displayed even more potent inhibitory activity over *Li*TryR and enhanced leishmanicidal activity. Remarkably, several of these novel triazole-bearing compounds were able to kill both extracellular and intracellular parasites in cell cultures.

## Introduction

Leishmaniasis is an infectious disease caused by intracellular protozoan parasites from more than 20 *Leishmania* species and transmitted to humans through the bite of infected female phlebotomine sandflies. According to the World Health Organization (WHO), leishmaniasis is one of the major neglected tropical diseases that affects 12 million people in 98 countries, being especially prevalent in tropical and subtropical areas.^1^ Visceral leishmaniasis (VL), also known as Kala-azar, is the most severe form of the disease with more than 60 000 human deaths annually. VL is caused by *Leishmania donovani* and *Leishmania infantum* parasites worldwide, including Mediterranean countries.^2^ No effective vaccine for humans has so far been developed, and leishmaniasis control mainly relies on chemotherapy.^3,4^ However, currently available treatments are inadequate because of high costs, toxicity, drug resistance, and the need for parenteral administration.^5,6^ Therefore, there is an urgent need to find new effective innovative drugs against this disease.

Herein, we report a target-based approach for the discovery of novel agents against *L. infantum* parasites targeting a protein that is essential for parasite survival and exclusive for these parasites.^7,8^ We have focused on trypanothione (Try) disulfide reductase (TryR) because it is one of the few genetically and chemically validated drug targets, and validation is one important criterion in target assessment.^9−11^ Among other highly relevant functions, this enzyme is essential for antioxidant defense in trypanosomatids. The Try/TryR couple in these parasites substitutes for the glutathione/glutathione-disulfide reductase (GR) pair characteristic of most eukaryotic organisms. Accordingly, the absence of TryR in mammalian cells, the significant differences between TryR and human GR (hGR) (the active sites of the two enzymes have opposite net charges and different volumes), and its crucial role for parasite survival make TryR an attractive target for new chemotherapeutics.^12−15^ Since this enzyme was identified, many potent and selective competitive polycationic TryR inhibitors (which mimic the positively charged Try in the active site) have been described. However, there are scarce reports of potent TryR-inhibiting compounds with adequate antiparasitic activity.^16,17^ This is so because it has been shown that survival of the parasites is only affected when TryR activity is reduced by more than 90%, probably as a consequence of the high intracellular concentrations of its natural substrate (Try) in the cell.^18^ This implies that, to be effective against the parasites, competitive inhibitors must have very high binding affinities so as to give rise to inhibition constants in the low nanomolar range. Nonetheless, one of the most potent (submicromolar) competitive inhibitors of Trypanosoma *brucei* (*Tb*TryR) described to date is almost inactive against *L. donovani*.^19^ This limitation, together with the need for very high affinities imposed by the high intracellular concentrations of both the natural substrate (between 3 and 50 μM under steady-state conditions and up to 150 μM under oxidative stress) and TryR itself (0.5–1.3 μM), make noncompetitive and/or irreversible inhibitors emerge as more attractive alternatives for targeting this enzyme.^20a,20b,21^

The crystal structure of TryR from *L. infantum* (*Li*TryR) was first solved by Baiocco et al.^22^ and is very similar to that of other trypanosomatids.^13,14,16,23,24^ This enzyme is a twofold symmetrical homodimer that contains two active sites and two reduced nicotinamide adenine dinucleotide phosphate (NADPH)- and flavin adenine dinucleotide (FAD)-binding domains separated by a large and well-characterized interface.^25^ Each Try binding site is located in a large solvent-exposed cavity at the interface between the two monomers. Several X-ray crystal structures of TryR in complex with a variety of competitive reversible inhibitors have shown that the large pocket allows not only several binding modes for small-molecule inhibitors but also the simultaneous binding of more than one inhibitor molecule.^23^ Such unpredictable binding modes, due to the large size of the active site, make the rational design of competitive, high-affinity inhibitors of TryR a very challenging task.

Bearing in mind the homodimeric nature of this enzyme, back in 2013 we devised an alternative inhibition strategy that aims to disrupt the protein–protein interactions responsible for *Li*TryR homodimerization.^25^ Initially, by means of a combination of molecular modeling and site-directed mutagenesis studies, we identified and validated E436—an amino acid located at an interfacial α-helix spanning from P435 to M447—as a hotspot for dimer stabilization and assessed its importance for the catalytic activity of the enzyme. As a “proof of concept” of this novel approach, we designed and tested a small library of linear peptides that represent rational variations of this α-helix. From these studies, the 13-mer-modified peptide sequence PKIIQSVGIS-Nle-K-Nle (**1**) emerged as a potent enzyme dimerization disruptor that also behaves as a strong inhibitor (in the submicromolar range) of the oxidoreductase activity of *Li*TryR.^25^ Next, a variety of helix-stabilized cyclic peptides^26,27^ and α,β^3^-peptide foldamers^28^ were successfully explored in our search to find peptidomimetics with increased proteolytic stability. However, conjugation with cell-penetrating peptides was always required to facilitate the cellular uptake of these peptide- or peptidomimetic-based *Li*TryR dimerization disruptors and to kill the parasites in cell culture.^28,29^ Thus, the design of small molecules with druglike properties better than those of the prototype peptide or the previous peptidomimetics appeared as a desirable goal.

**Figure 1:**
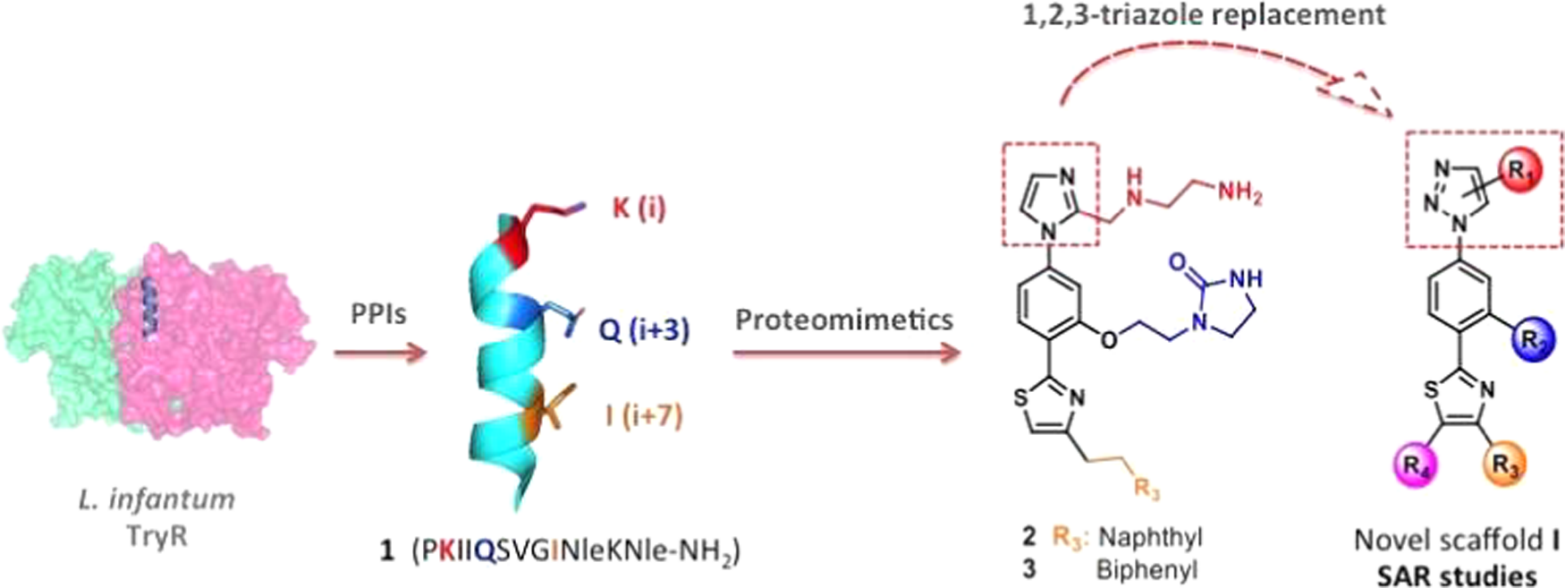
From a peptide dimerization disruptor of *Li*TryR (**1**) and in-house imidazoles **2** and **3** to the newly designed triazole-phenyl-thiazole scaffold **I**.

In this regard, we recently disclosed the results of our first steps toward nonpeptide disruptors of *Li*TryR dimerization with promising leishmanicidal activity by an α-helical mimetic approach.^30^ Among the reported proteomimetic scaffolds,^31−33^ the pyrrolopyrimidine^34^ and the 5-6-5 imidazole-phenyl-thiazole^35^ cores were selected to dictate the spatial orientations of the side chains of three key residues (K2, Q5, and I9) in the linear peptide prototype **1**. Imidazole-based compounds **2** and **3** (Figure 1), bearing a naphthalene or a biphenyl polyaromatic R_3_ substituent to mimic the hydrophobic isoleucine residue, emerged as potent inhibitors of the *Li*TryR oxidoreductase activity, while pyrrolopyrimidines were shown to be much less active. Although these small molecules displayed a moderate capacity to disrupt the *Li*TryR dimer, this effect was much less pronounced than that observed for the previous peptide-based inhibitors. Remarkably, the imidazole-based compounds were cell-permeable and showed significant leishmanicidal activity against both amastigote and promastigote forms of *Leishmania* parasites. However, these molecules displayed a cytotoxic activity similar to that observed for their peptidic and peptidomimetic predecessors and exhibited low selectivity indexes (SIs).

1,2,3-Triazole-containing compounds have been widely investigated because they are considered privileged scaffolds in different areas of medicinal chemistry,^36−38^ including several examples in the antileishmanial field.^39^ Interest in this molecular architecture has also been fueled by its expedient synthesis through “click chemistry”.^40^ In this scenario, we decided to apply the bioisosteric replacement of imidazole by 1,2,3-triazole in our recently described imidazole-phenyl-thiazoles.^30^ We herein report the synthesis and structure–activity relationship (SAR) studies of novel 1,2,3-triazole-phenyl-thiazole compounds of general formulae **I** (Figure 1) to demonstrate the potential of this novel chemotype for *Li*TryR dimerization disruption and leishmanicidal activity against *L. infantum* parasites. A whole set of 26 triazole-based compounds modified at four different positions in the new scaffold (R_1_–R_4_ substituents) was prepared and evaluated. Five of these molecules display activities that very closely resemble that of the peptide prototype **1** and likewise behave as noncompetitive, slow-binding inhibitors.^41^ Molecular modeling studies identified a putative binding site for these compounds at an almost unexplored central interfacial cavity of the enzyme and provided a structural rationale to the observed SAR results that was supported by ensuing rational design, synthesis, and activity of a new symmetrical triazole analogue with the desired properties.

## Results and Discussion

### Chemistry

**Scheme 1:**
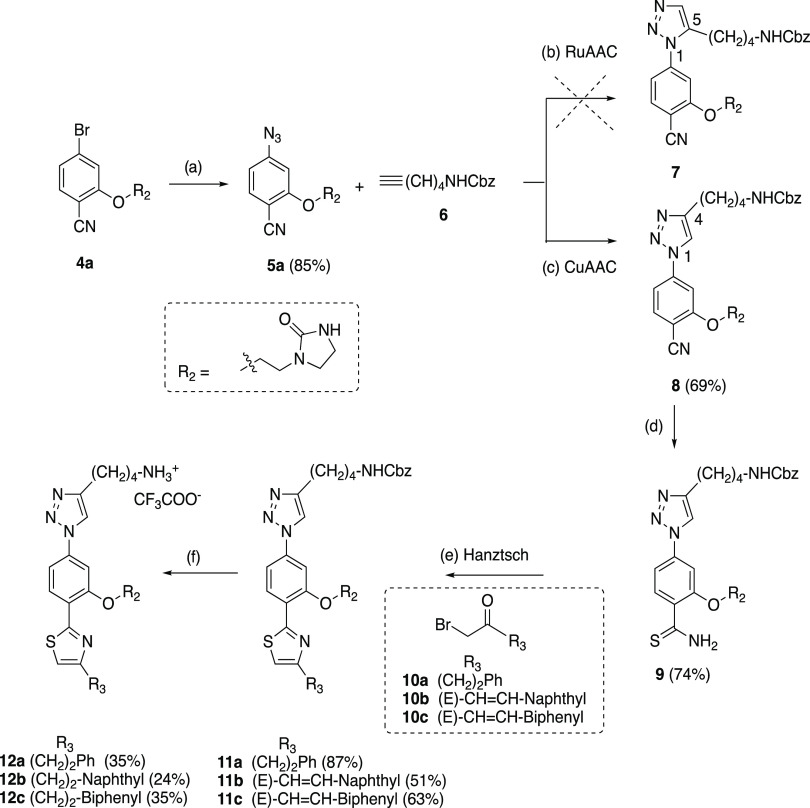
Synthesis of the First Series of 1,4-Disubstituted 1,2,3-Triazole Compounds **12a**–**c** Reagents and conditions: (a) NaN_3_, DMSO, molecular sieves 4 Å, 100 °C, 72 h; (b) Cp*RuCI(PPh_3_)_2_ (5 or 10 mol %), DMF, rt to 100 °C; (c) CuSO_4_·5H_2_O, sodium ascorbate, H_2_O/EtOH 1:1, rt, 8 h; (d) (NH_4_)_2_S 20% aq, DMF, 80 °C, 4 h; (e) α-bromomethylketones **10a**–**c**, ^*i*^PrOH, 70 °C, 5 h; (f) H_2_, Pd/C 10%, TFA, THF/MeOH 1:1, rt, 2 h.

The synthesis of the target triazole-phenyl-thiazole type **I** compounds is exemplified by the preparation of the first series of 1,2,3-triazole derivatives **12a**–**c** (bearing R_1_–R_3_ substituents similar to those present in the predecessor imidazole analogues **2** and **3**), as shown in Scheme 1. The synthetic route started from the previously described bromide intermediate **4a**(30) bearing an imidazolidinone ethyl R_2_ group (used as a Gln mimetic in the previous imidazole derivatives) by treatment with sodium azide in anhydrous dimethyl sulfoxide (DMSO) at 100 °C for 72 h to afford the azide intermediate **5a** in 85% yield. The addition of molecular sieves with pore openings of 4 Å was required to avoid the partial hydrolysis of the azide group to the undesired amine. The next step in the synthetic route was the 1,3-dipolar cycloaddition of the azide intermediate with terminal alkynes substituted with the appropriate R_1_ group (as a Lys mimetic) to generate the 1,2,3-triazole ring. Initial attempts to use the ruthenium-catalyzed azide–alkyne cycloaddition (RuAAC)^40b^ from **5a** and commercially available N-Cbz-protected terminal alkyne **6** in the presence of catalytic amounts (5 or 10%) of a cyclopentadienyl Ru catalyst [Cp*RuCl(PPh_3_)_2_] at different temperatures in dry dimethylformamide (DMF) and an inert atmosphere did not produce the expected 1,5-disubstituted 1,2,3-triazole **7**. Only unreacted starting materials or complex decomposition mixtures were observed under these conditions. In contrast, the 1,4-disubstituted 1,2,3-triazole regioisomer **8** could be easily obtained in 56% yield by the 1,3-dipolar copper(I)-catalyzed azide–alkyne cycloaddition (CuAAC)^40a^ of azide **5a** and the terminal alkyne **6** using the CuSO_4_/sodium ascorbate catalyst system at room temperature for 8 h in H_2_O/EtOH (1:1 mixture) as the solvent. Subsequent treatment of nitrile **8** with aqueous (aq) 20% ammonium sulfide at 80 °C for 4 h provided the thioamide intermediate **9** in 74% yield. Next, Hantzsch thiazole synthesis from 1 equiv of **9** and 1 equiv of the commercially available α-bromomethylketone **10a** or the previously synthesized **10b** and **10c**(30) (bearing a naphthyl or a biphenylethyl hydrophobic R_3_ substituent) heated in isopropanol at 70 °C for 36 h gave the expected α/β-unsaturated derivatives **11a**–**c** monosubstituted at the 4-position of the thiazole ring in 87, 51, and 63% yields, respectively (Scheme 1). Finally, treatment of **11a**–**c** with H_2_, Pd/C in tetrahydrofuran (THF)/MeOH mixture in the presence of trifluoroacetic acid (TFA) at room temperature allowed the simultaneous hydrogenolysis of NHCbz and hydrogenation of the double bond to afford the deprotected target compounds **12a**–**c** as monotrifluoroacetate salts in 35, 24, and 35% yields, respectively. Chromatographic purification by Biotage using reverse-phase columns was always required to obtain the final compounds with purities higher than 95% for their biological evaluation.

As will be discussed below, the 1,2,3-triazole compounds **12a**–**c** emerged as more potent dimerization disruptors of *Li*TryR than the reference imidazoles and also behaved as strong inhibitors of the oxidoreductase activity of the enzyme. These results prompted us to develop a novel series of triazole derivatives **12d**–**s** (see structures in Table 1 and Scheme S1) modified at the R_1_–R_3_ substituents to perform a SAR study. Regarding modifications at R_1_ and R_2_, the protonated aminobutyl group linked to the triazole was replaced by OH, Me, or COOH (**12d**–**g**) or by a shorter aminopropyl substituent (**12h**, **12i**), while the R_2_ imidazolidinone (attached to the central phenyl ring through an ethyl linker) was removed (**12j**, **12k**). In terms of R_3_ modifications, bulky alkyl substituents (**12l**, **12m**) or a phenyl group linked to the thiazole ring through a polymethylene spacer of a different length (**12n**–**p***vs***12a**) were explored.

**Table 1:**
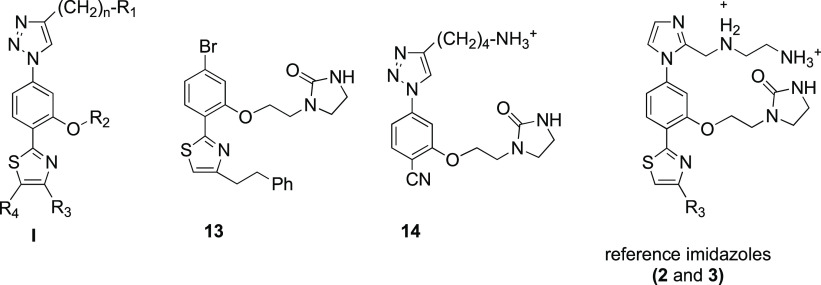
Half-Maximal Inhibitory Concentration (IC_50_) ± Standard Error (SE) Values for Triazole Compounds **I** and the Truncated Analogues **13** and **14** in the TryR Oxidoreductase Activity and *Li*TryR Monomer Displacement Assays^a^^,^^f^

compounds	R_1_	*n*	R_2_	R_3_	R_4_	IC_50_^act^ (μM)^b^	IC_50_^dim^ (μM)^c^
**1**						1.5 ± 0.2	7.0 ± 0.6
**2**				(CH_2_)_2_naphthyl		5.1 ± 0.4	26%^d^
**3**				(CH_2_)_2_biphenyl		8.6 ± 1.4	32%^d^
**12a**	NH_3_^+^	4	(CH_2_)_2_Im*	(CH_2_)_2_Ph	H	14.6 ± 1.0	38.1 ± 1.2
**12b**	NH_3_^+^	4	(CH_2_)_2_Im*	(CH_2_)_2_naphthyl	H	5.9 ± 1.1	7.1 ± 0.6^e^
**12c**	NH_3_^+^	4	(CH_2_)_2_Im*	(CH_2_)_2_biphenyl	H	10.9 ± 1.8	4.8 ± 0.6^e^
Modifications at R_1_							
**12d**	OH	4	(CH_2_)_2_Im*	(CH_2_)_2_Ph	H	>75	>75
**12e**	CH_3_	3	(CH_2_)_2_Im*	(CH_2_)_2_Ph	H	>75	>75
**12f**	CH_3_	3	(CH_2_)_2_Im*	(CH_2_)_2_biphenyl	H	>75	>75
**12g**	COOH	3	(CH_2_)_2_Im*	(CH_2_)_2_Ph	H	>75	>75
**12h**	NH_3_^+^	3	(CH_2_)_2_Im*	(CH_2_)_2_Ph	H	20.8 ± 2.3	>75
**12i**	NH_3_^+^	3	(CH_2_)_2_Im*	(CH_2_)_2_Biph	H	19.7 ± 3.8	10.8 ± 0.1
Modifications at R_2_							
**12j**	NH_3_^+^	4	Me	(CH_2_)_2_Ph	H	20.1 ± 2.5	>75
**12k**	NH_3_^+^	4	Me	(CH_2_)_2b_iphenyl	H	26.8 ± 5.7	16.1 ± 0.7
Modifications at R_3_							
**12l**	NH_3_^+^	4	(CH_2_)_2_Im*	O*^i^*Pr	H	54.5 ± 4.4	>75
**12m**	NH_3_^+^	4	(CH_2_)_2_Im*	CH_2_*^t^*Bu	H	24.1 ± 1.4	>75
**12n**	NH_3_^+^	4	(CH_2_)_2_Im*	Ph	H	11.2 ± 0.2	29.0 ± 1.5
**12o**	NH_3_^+^	4	(CH_2_)_2_Im*	CH_2_Ph	H	43.6 ± 1.1	61.2 ± 2.8
**12p**	NH_3_^+^	4	(CH_2_)_2_Im*	(CH_2_)_3_Ph	H	14.6 ± 1.7	19.1 ± 1.1
**12q**	NH_3_^+^	4	(CH_2_)_2_Im*	(CH_2_)_2_THQ*	H	6.9 ± 2.1	>75
**12r**	NH_3_^+^	4	(CH_2_)_2_Im*	(CH_2_)_2_DHB*	H	11.9 ± 0.6	10.7 ± 0.5
**12s**	NH_3_^+^	4	(CH_2_)_2_Im*	(CH_2_)_2_DBF*	H	6.6 ± 1.9	8.8 ± 0.2
Truncated Analogues							
**13**						>75	>75
**14**						>75	>75
Modifications at R_3_ and R_4_							
**19a**	NH_3_^+^	4	(CH_2_)_2_Im*	Ph	Ph	4.3 ± 1.0	7.1 ± 1.7
**19b**	NH_3_^+^	4	(CH_2_)_2_Im*	biphenyl	Ph	18.4 ± 3.9	11.3 ± 0.2
**19c**	NH_3_^+^	4	(CH_2_)_2_Im*	PhOPh	Ph	16.9 ± 2.7	9.0 ± 0.5
**19d**	NH_3_^+^	4	(CH_2_)_2_Im*	(CH_2_)_2_biphenyl	Ph	19.8 ± 2.3	5.7 ± 0.5
**19e**	NH_3_^+^	4	(CH_2_)_2_Im*	(CH_2_)_2_PhOPh	Ph	19.3 ± 3.7	6.7 ± 1.0

a Linear peptide **1** and imidazole-based compounds **2** and **3** were included as reference compounds.

b Enzymatic activity >75 indicates that the IC_50_ value is higher than 75 μM (maximum assayed). Results are representative of three independent experiments, each performed in triplicate.

c Dimer quantitation assay (enzyme-linked immunosorbent assay (ELISA)).^25^

d Percentage of inhibition observed at 20 μM as reliable IC_50_ values could not be determined in this case.

e Percentages of inhibition observed at 20 μM were 73 and 78% for **12b** and **12c**, respectively.

f Im* = imidazolidinone, THQ* = tetrahydroquinoline (TFA salt), DHB* = dihydrobenzofuranyl, DBF* = dibenzofuranyl.

**Scheme 2:**
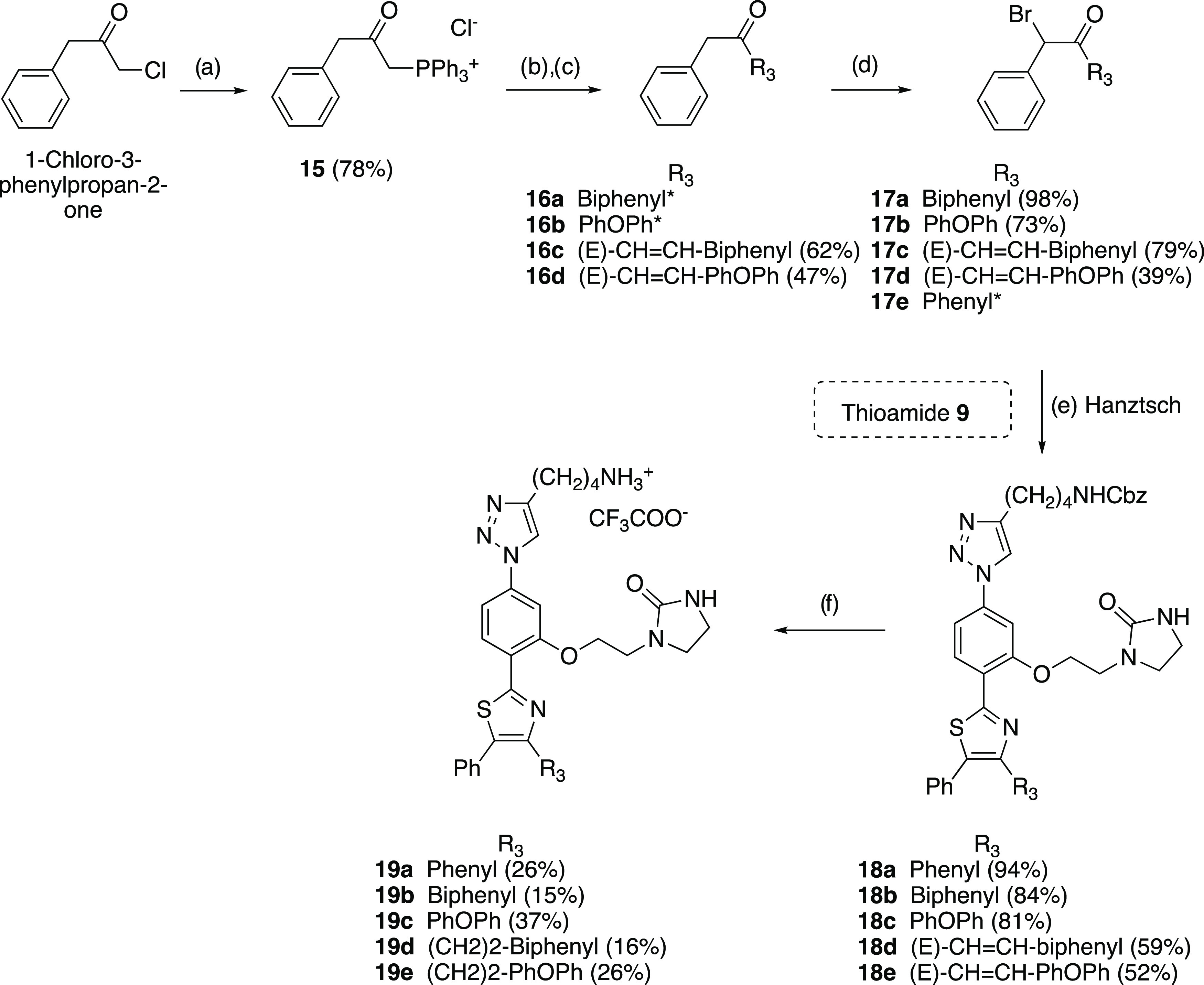
Synthesis of Triazoles Disubstituted at the Thiazole Ring (**19a**–**e**) Reagents and conditions: (a) PPh_3_, dry toluene, 110 °C, 6 h; (b) KOH, dry toluene, 110 °C, 3 h; (c) RCHO, 110 °C, 3 h; (d) NBS, *p*-TsOH, CH_3_CN, rt, 14 h; (e) thioamide **9**, **17a**–**e**, ^*i*^PrOH, 70 °C, 5 h; (f) H_2_, Pd/C 10%, TFA, THF/MeOH 1:1, rt, 2 h. *Commercially available reagents.

Since the presence of a biphenyl or naphthyl substituent at R_3_ was highly relevant for potent enzyme inhibition and antileishmanial activity, other polyheteroaromatic rings were also explored at R_3_ (**12q**–**s**). The synthesis of the novel triazole analogues **12d**–**s** involved (i) a high-yielding nucleophilic aromatic substitution (SN_Ar_) of aryl fluorides with the appropriate alcohols (bearing the R_2_ substituent),^30^ (ii) CuAAC reactions between azide intermediates and terminal alkynes (containing the R_1_ substituent), (iii) Hanztsch thiazole synthesis of thioamides with suitable α-bromomethylketones (with the R_3_ substituent), and (iv) and a final step of catalytic hydrogenation to yield the deprotected final compounds using similar protocols to those described above. It should be noted that in the catalytic hydrogenation of the protected triazole compound intermediate **11q** (see Scheme S1) bearing a quinoline moiety as the R_3_ substituent, the 1,2,3,4-tetrahydroquinoline compound **12q** (see Scheme S1) was isolated as a ditrifluoroacetate salt due to the partial hydrogenation and salt formation of the quinoline ring nitrogen atom. The synthetic experimental details of the target **12q**–**s** compounds and the noncommercially available key α-bromomethyl-ketone intermediates **10m**, **10p**–**s** are included in the Supporting Information (see Schemes S1 and S2). Scaffold-truncated simplified compounds (**13** and **14**, see Table 1), possessing only two aromatic rings and substituents, were also prepared for the SAR studies following similar synthetic protocols (see also the Supporting Information for experimental details; Scheme S3). To further investigate the SAR of the triazole-based compounds, we next focused on the preparation of an additional series of triazole analogues **19a**–**e** (Scheme 2) disubstituted at both positions 4 and 5 of the thiazole ring. Polyaromatic groups directly attached or linked through an ethyl spacer to the 4-position and a phenyl group at the 5-position were selected as R_3_ and R_4_ substituents, respectively. As shown in Scheme 2, the preparation of these compounds required the previous synthesis of the noncommercially available α-bromobenzylketones **17a**–**d**. Thus, **17a**–**c** bearing a biphenyl or a phenyloxyphenyl group at R_3_ directly attached to the carbonyl were synthesized by bromination of the commercially available benzylketones **16a**–**c** with *N*-bromosuccinimide (NBS) in *p*-toluensulfonic acid (*p*-TsOH) in good to excellent yields.

**Scheme 3:**
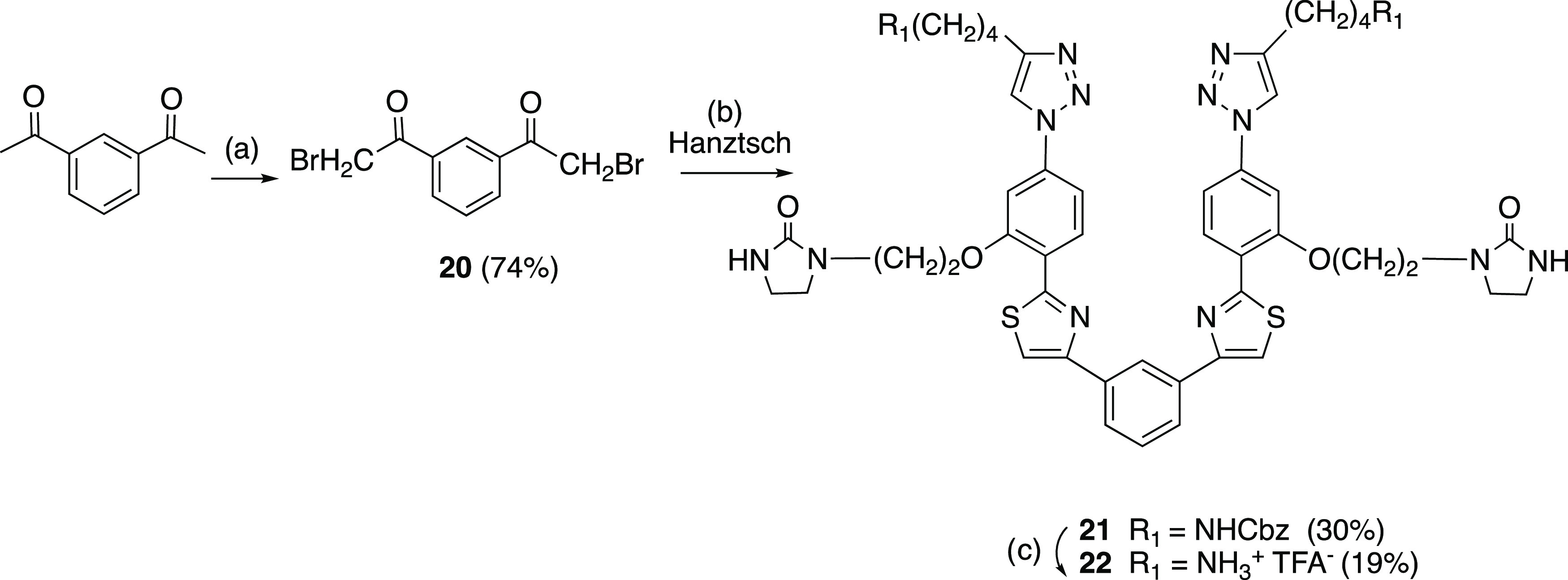
Synthesis of the Newly Designed Symmetrical Analogue 22 Reagents and conditions: (a) NBS, *p*-TsOH, acetonitrile, rt, 14 h; (b) thioamide **9**, ^*i*^PrOH, 70 °C, 5 h; (c) H_2_ Pd/C 10%, TFA, THF/MeOH, 1:1, rt, 2 h.

On the other hand, α-bromobenzyl α,β-unsaturated ketone **17d** with a polyaromatic group was obtained by regioselective bromination of the most favorable benzylic position with NBS in *p*-TsOH of the corresponding benzylketones **16d**, which was previously synthesized from 1-chloro-3-phenylpropan-2-one by treatment with PPh_3_ to give the phosphonium salt **15** followed by a Wittig procedure with the corresponding polyaromatic aldehydes in moderate yields. Next, Hantzsch thiazole synthesis from thioamide intermediate **9** with the commercial **17e** or synthetic α-bromobenzylketones **17a**–**d**, followed by catalytic hydrogenolysis of the N-Cbz group and simultaneous olefin hydrogenation of **18a**–**e** using 10% Pd/C in the presence of TFA, afforded the desired unprotected disubstituted thiazole derivatives **19a**–**e** as monotrifluoroacetate salts in low to moderate yields. Finally, we also designed (see section Computational Studies below) and prepared (Scheme 3) a new symmetrical triazole analogue (**22**) by treating 2 equiv of thioamide intermediate **9** with 1 equiv of α-bromomethylketone **20** (previously synthesized by bromination with NBS of commercially available 1,3-diacetyl benzene) under Hantzsch reaction conditions, followed by catalytic hydrogenolysis of the NHCbz group using similar procedures to those described above.

### Biological and Structural Studies

#### Enzymatic Assays

##### *L. infantum* TryR Inhibition and Dimerization Assays

All of the novel triazole-phenyl-thiazole modified at substituents R_1_–R_3_ (**12a**–**s**), disubstituted thiazole analogues bearing R_3_ and R_4_ substituents (**19a**–**e**), and truncated simplified compounds (**13** and **14**) were evaluated for their effect on both the oxidoreductase activity and the dimerization status of *Li*TryR.^25^ The prototype peptide **1** and imidazole-containing compounds **2** and **3** were used as reference molecules. Screening was performed at different concentrations ranging from 0.1 to 75 μM. A concentration of 1 μM TS_2_ was used for this initial screening. This low substrate concentration is sufficient for comparison of compound inhibitory activity in the oxidoreductase assay. The best candidates were then characterized further to obtain their dissociation constants. The IC_50_ values determined in the activity (IC_50_^act^) and dimerization (IC_50_^dim^) assays are shown in Table 1.

According to the results obtained with the first series of triazole-based compounds **12a**–**c** (Table 1), analogues containing a naphthyl or a biphenyl group at R_3_ (**12b**, **12c**) potently inhibited the TryR oxidoreductase activity, showing similar IC_50_^act^ values (in the low micromolar range) to those obtained for the reference imidazole compounds **2** and **3**. Remarkably, **12a**–**c** were much more potent *Li*TryR dimerization disruptors than their imidazole counterparts. Thus, in contrast to **2** and **3**, for which no reliable IC_50_^dim^ could be measured and only ∼30% dimerization inhibition was observed at a concentration of 20 μM, the IC_50_^dim^ values for **12b**, **12c** (7.1 and 4.8 μM, respectively) were similar to (or even slightly lower than) those obtained for the potent prototype peptide **1**. Furthermore, replacement of the naphthyl or biphenyl groups at R_3_ by a phenyl (**12b**, **12c***vs***12a**) slightly increased the IC_50_^act^ value, while it significantly decreased (in a 5–8-fold range) the ability to disrupt the *Li*TryR dimer. We took this result as a clear indication that the presence of a polyaromatic substituent at R_3_ is relevant to potent *Li*TryR dimer disruption.

SAR studies on the second series of triazole compounds (**12d**–**s**) with variations at the R_1_–R_3_ substituents began with modifications at the R_1_ position. As shown in Table 1, the presence of a positively charged amino alkyl group at R_1_ appears as an absolute requirement for inhibition of both activity and dimerization, as evidenced by the complete loss of effect observed when the amino group was replaced by hydroxyl, methyl, or carboxylic acid groups (**12d**–**g***vs***12a**, **12c**). As regards the length of the R_1_ spacer attached to the amino group, shortening the original tetramethylene linker to a trimethylene brought about a ∼2-fold reduction of potency in both assays (**12h**, **12i***vs***12a**, **12c**). The existence of the imidazolidinone ring as the R_2_ substituent on the central phenyl ring also appeared important for optimal activity since its removal (**12j**, **12k***vs***12a**, **12c**) resulted in compounds twice or thrice less active in both assays. Modifications at the R_3_ substituent (located at the 4-position of the thiazole ring) had a pronounced effect on enzymatic activity inhibition and, even more so, on dimerization disruption. Thus, the presence of a (poly)aromatic substituent at R_3_ was crucial for enhanced potency since compounds **12l**, **12m**, with bulky aliphatic groups, exhibit much less inhibitory activity on the enzyme and no destabilization effect on the *Li*TryR homodimer in comparison with the (poly)aromatic analogues **12a**–**c**. In comparison, varying the length of the linker connecting the phenyl group to the thiazole (**12n**–**p***vs***12a**) turned out to be not so crucial because, in general, it did not significantly affect the inhibitory activity.

In view of these results, and even though the phenylpropyl derivative **12p** displayed the lowest IC_50_^dim^ value, for the next round of modifications, we decided to focus on shortening the propylene linker at R_3_ to an ethylene (**12a**) or even removing it altogether (**12n**) because of the better synthetic accessibility of the corresponding bromoketones. On the other hand, replacement of the naphthyl or the biphenyl group at R_3_ in the most potent analogues **12b**, **12c** by polyheteroaromatics such as a dihydrobenzofuranyl substituent (**12r**) or a larger hydrophobic tricyclic dibenzofuranyl moiety (**12s**) also yielded very potent compounds but did not further increase the inhibitory activity of the most potent analogues (**12b**, **12c**) in any of the two assays. In contrast, the introduction of a positively charged tetrahydroquinoline ring (**12q**) annihilated the dimerization disruption capacity of the molecules even though potent inhibition of the redox activity of the enzyme was retained. In contrast, no activity in any of the assays was observed for the simplified truncated scaffold derivatives **13** and **14**, in which the appropriately substituted triazole or thiazole rings were removed. This additional evidence further supports the key role played by these two rings (and the R_1_ and R_3_ substituents) in this type of inhibitor.

Finally, we also evaluated a new series of triazole compounds (**19a**–**e**) disubstituted at the 4- and 5-positions of the thiazole ring with R_3_ and R_4_ aromatic moieties. Some of these molecules combined excellent inhibition in the redox assay with potent *Li*TryR dimer disruption ability. Interestingly, both activities were significantly improved by a factor of 2–4 for **19a**, in which a phenyl group is bonded at both R_3_ and R_4_ positions, in comparison with the R_3_ phenyl monosubstituted analogue **12n**. Introduction of a larger biphenyl or a diphenylether group as the R_3_ substituent (maintaining the phenyl group at R_4_) significantly decreased the inhibitory activity of the highly potent diphenylsubstituted analogue, but their dimerization disruption ability was affected only slightly (*cf*. **19b**, **19c***vs***19a**). Also, the combination of R_3_ polyaromatics attached to the thiazole ring through an ethylene linker with an R_4_ phenyl group resulted in a significant deleterious effect on the potency of the compounds as inhibitors of the *Li*TryR oxidoreductase activity, although the potent disrupting effect on dimerization was maintained (*cf*. **19d**, **19e***vs***19b**, **19c** or the corresponding monosubstituted **12c**).

In general, the most active compounds in the enzymatic activity assay were also the best dimerization disruptors in the ELISA with the exception of the highly potent tetrahydroquinoline inhibitor **12q**, which did not show any dimerization disruption effect, or the thiazole-disubstituted analogues **19b**–**e**, which behave as moderate redox inhibitors but highly potent dimerization disruptors of the enzyme. All in all, our SAR study showed that (i) a positively charged butylamine (R_1_), (ii) an imidazolidinone (R_2_) together with a hydrophobic poly(hetero)aromatic group as R_3_ (**12b**, **12c**; **12r**, **12s**) substituents, or (iii) the combination of a phenyl as R_3_ with an additional phenyl as R_4_ (**19a**) are highly relevant to optimal enzyme inhibition and dimerization disruption in this series.

To gain insight into the selectivity of the compounds for *Li*TryR, representative molecules **12a**–**c**, **12n**, **12r**, and **19a** were studied as inhibitors of human glutathione-disulfide reductase (hGR), the closest related host enzyme. Remarkably, none of these compounds showed any inhibitory activity against this enzyme. As an example, a comparison of the activities of *Li*TryR and hGR in the presence of compounds **12b** and **12c** at 50 μM of their respective substrates is shown in Figure S15.

##### Mechanism of *Li*TryR Inactivation

**Figure 2:**
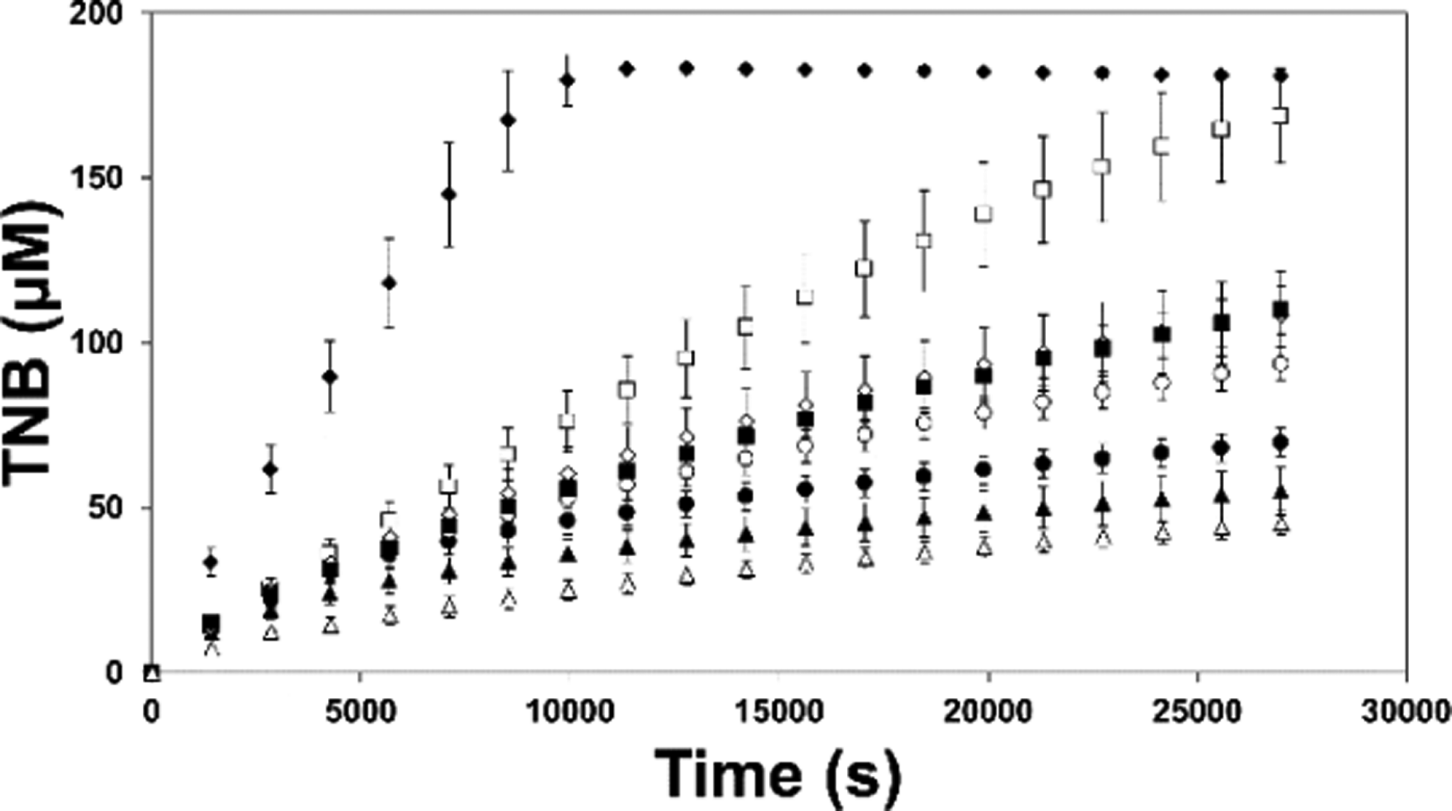
Time-dependent inhibition of *Li*TryR. Reaction progress curves of *Li*TryR in the absence (◆) and presence of 25 μM mepacrine (**□**), 10 μM peptide **1** (▲), 10 μM **12c** (**○**), 10 μM **12b** (●), 10 μM **12r** (◊), 10 μM **12s** (■), or 10 μM **19a** (**△**). [DTNB] = 100 μM. [NADPH] = 150 μM. Data represent the mean ± SD of three independent experiments.

As already described for **1**,^41^ the peptidic precursor of all other compounds, a progressive loss of *Li*TryR activity is observed during the course of the reaction in the presence of **12b**, **12c**, **12r**, **12s**, and **19a** (Figure 2), selected as the most potent inhibitors in these series. In stark contrast, reaction rates in the presence of the classical competitive inhibitor mepacrine are almost constant until 5,5′-dithiobis(2-nitrobenzoic acid) (DTNB) (which allows substrate regeneration) is exhausted. The progress curves of the reactions in the presence of the selected compounds are typical of slow-binding inhibitors.^42a,42b^

**Scheme 4:**
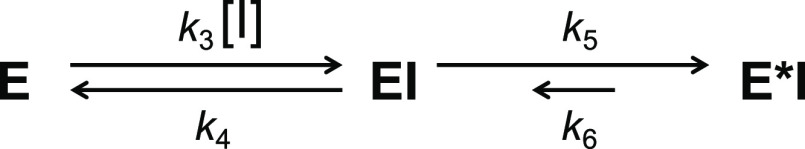
Two-Step Mechanism of Time-Dependent Inhibition of *Li*TryR The first step is a rapid equilibrium that generates the enzyme–inhibitor (EI) complex. This process is governed by the association rate (*k*_3_) and the dissociation rate (*k*_4_) constants. The second step consists of slow and reversible inactivation of the enzyme that is governed by the forward isomerization rate constant (*k*_5_) and the much smaller reverse rate constant (*k*_6_).

Despite the loss of activity observed during the reaction, dependency of the initial velocities (*v*_i_) on **19a** and TS_2_ concentrations could be fitted to a model in which a rapid equilibrium is reached between the free enzyme and inhibitor (E + I) and the enzyme/inhibitor (EI) complex (Scheme 4). This analysis, based on *v*_i_ values, was performed both in a DTNB-coupled assay and in a direct NADPH-oxidation assay (Figure S1 and Table S1). Our data consistently reveal a nonlinear noncompetitive hyperbolic inhibition mechanism.

Peptide **1** has already been described as a pseudoirreversible time-dependent noncompetitive inhibitor that is able to promote a two-step process of enzyme isomerization (Scheme 4).^41^ The kinetic data obtained for **19a**, the most potent compound, were also analyzed considering a similar mechanism of enzyme inactivation.

In this inactivation model, a rapid and reversible primary inhibition event generates an enzyme–inhibitor binary complex (EI complex). The process of generating an inactive form of *Li*TryR upon **19a** binding is governed by *k*_5_ and *k*_6_ first-order rate constants, which describe an isomerization equilibrium between the initial enzyme–inhibitor complex (EI) and a second high-affinity complex (E*I) (Scheme 4). This slow isomerization process gives rise to a characteristic bending of the reaction curve that is defined by the first-order rate constant *k*_obs_, whose value describes the conversion from the initial reaction rate (*v*_i_) to the final steady-state velocity (*v*_s_).

**Figure 3:**
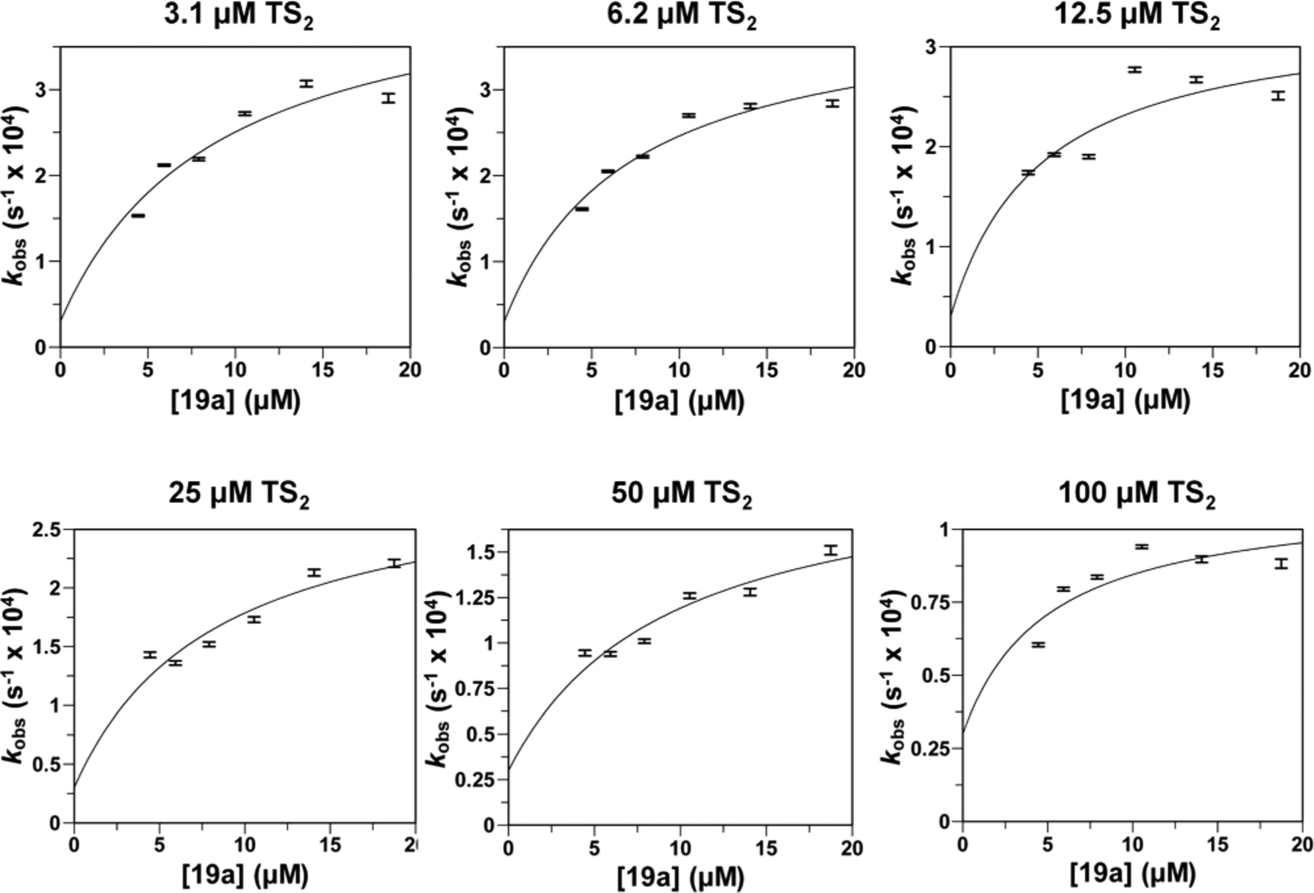
Concentration dependence of the observed rate constants of *Li*TryR (0.8 nM) inactivation by **19a** at different TS_2_ concentrations. Plot of the *k*_obs_ values (±standard errors) as a function of **19a** concentration at six different TS_2_ concentrations (3.1, 6.2, 12.5, 25, 50, and 100 μM) (*k*_obs_ determinations for every **19a** concentration at the different fixed TS_2_ concentrations assayed are shown in the Supporting Information). Curves were fitted using eq 2, and the results of the fits are shown in Table 2.

Adjustment to eq 1 (see equations in Experimental Section) of the progress curves of the reactions catalyzed by *Li*TryR allowed us to obtain the *k*_obs_ values for six **19a** concentrations (4.4, 5.9, 7.9, 10.5, 14.1, and 18.7 μM) at six different TS_2_ concentrations (3.1, 6.2, 12.5, 25, 50, and 100 μM) (Figures S2–S7 and Table S2). The different *k*_obs_ values obtained at each substrate concentration can be used to determine the apparent inhibitory constants *K*_i_^app^ and *K*_i_*^app^ defined as the dissociation constant for the initial EI complex and the constant for the overall dissociation from E*I to E + I, respectively (eq 2).^42a,42b^ Based on the relationship among *k*_obs_, *v*_i_, and *v*_s_ (*k*_6_ = *k*_obs_ × *v*_i_/*v*_s_) at all of the trypanothione disulfide (TS_2_) and **19a** concentrations assayed, *k*_6_ varies between 3.9 × 10^–6^ and 6.5 × 10^–5^ s^–1^. Taking into consideration the extremely slow process of recovery of the enzymatic activity after complete inactivation of *Li*TryR (400 nM) upon incubation with 25 μM **19a** for 16 h and subsequent 2500-fold dilution (Figure S13), a value of 3 × 10^–5^ s^–1^ was selected as a conservative upper value for *k*_6_. Fixing this value for *k*_6_ rendered very good estimations of *K*_i_^app^ and *K*_i_*^app^ after the fitting process for all of the substrate concentrations assayed (Figure 3 and Table 2).

**Table 2:** Apparent Inhibition Constants for *Li*TryR Time-Dependent Inhibition by **19a**^a^

**[TS**_**2**_**] (μM)**	*K*_i_^app^ (**μM**)	*K*_i_*^app^ (**μM**)
3.1	8.9 ± 3.1	0.6 ± 0.1
6.2	7.2 ± 1.7	0.5 ± 0.1
12.5	5.2 ± 2.6	0.5 ± 0.2
25	8.3 ± 3.0	0.8 ± 0.2
50	9.3 ± 3.2	1.4 ± 0.3
100	5.0 ± 2.7	1.3 ± 0.6

a *K*_i_^app^ and *K*_i_*^app^ values at different TS_2_ concentrations were obtained by fitting to eq 2 the *k*_obs_ values for every **19a** concentration at the different fixed TS_2_ concentrations assayed. Results are the estimated values of the nonlinear regression ± associated standard errors.

The relationships between the different *K*_i_^app^ and *K*_i_ values in slow-binding inhibitors are the same as those for classical linear reversible inhibitors.^42a,42b^ By applying eq 3 to fit the *K*_i_^app^ values obtained at the six different TS_2_ concentrations assayed (Figure S8A), we could estimate the values of α (0.8 ± 0.4) and *K*_i_ (7.8 ± 1.7 μM). A similar approach (eq 4 and Figure S8B) was followed to estimate the values of α* (5.5 ± 2.9) and *K*_i_* (0.5 ± 0.1 μM) for the two-step global process of enzyme inhibition. According to these results, formation of the initial EI complex is not affected by the presence of substrate, a behavior that is characteristic of a pure noncompetitive process (α ∼ 1). The isomerization step from EI to E*I strongly enhances the inhibitory activity of **19a**, which is characterized by an overall *K*_i_* value of 0.5 μM. This isomerization process is, as expected, slightly impaired by the presence of substrate (α = 5.5) because of the generation of the enzyme–substrate–inhibitor complex, which decreases the concentration of the EI complex and thereafter the rate of E*I generation. Despite this small substrate effect, an α value of 5.5 for the overall process is still in the range of α values characteristic of noncompetitive (mixed) inhibitors. The overall inhibitory process is depicted in Scheme S5. Because of the ability of **19a** to cause enzyme dissociation, the characteristic isomerization of the enzyme in this mechanism of slow-binding inhibition is expected to be related to homodimer disruption.

Interestingly, compounds **12b** and **12q** have similar IC_50_ values in the reductase assay but only **12b** interferes with dimerization. The kinetic analyses reveal that both compounds are time-dependent inhibitors but only inhibition caused by **12b** follows a two-step process of enzyme isomerization (Figure S11 and Table S3). Plots of *k*_obs_*vs* inhibitor concentration for **12q** fit to a straight line that is indicative of a single-step binding mechanism (Figure S12, Table S4, and Scheme S4), which agrees with its inability to disrupt the homodimer.

### Computational Studies

**Figure 4:**
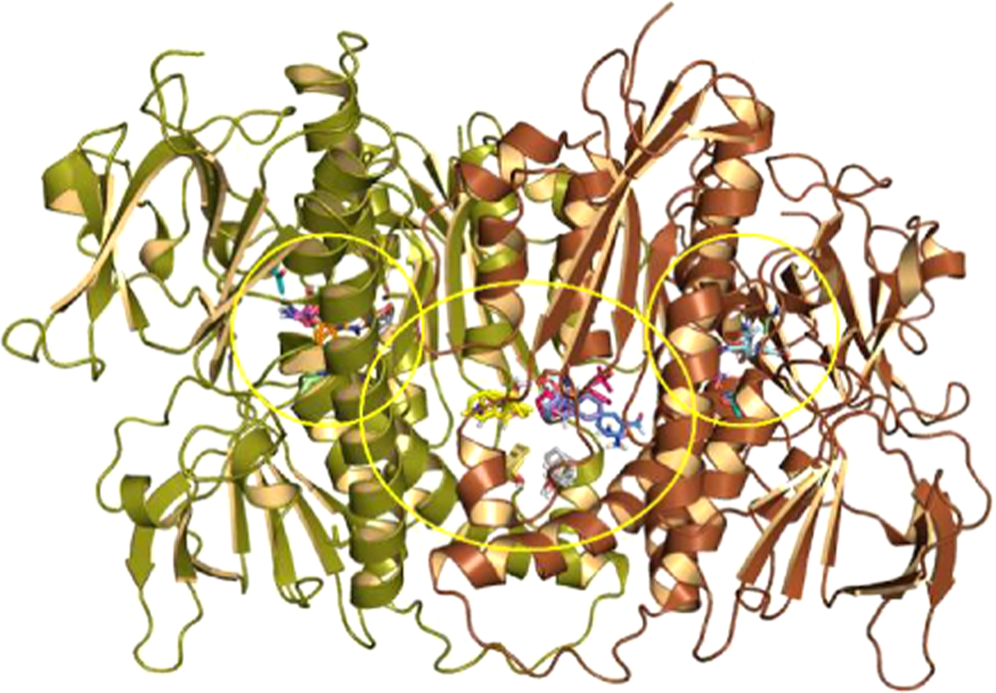
*Li*TryR dimer structure. FTMap identified the central interfacial cavity and the two adjacent sites where the two FAD cofactors are lodged as putative ligand-binding sites (circled by yellow lines).

Despite numerous attempts to form cocrystals with *Li*TryR or soak crystals of the enzyme with moderately or highly potent triazole-based dimerization disruptors **12a**, **12b**, and the heteroaromatic **12r**, no diffraction-quality crystal containing an enzyme/drug complex could be obtained. Of note, similar problems were encountered in previous cocrystallization efforts with the predecessor imidazoles **2** and **3**.^30^ Because of these so-far insurmountable hurdles, we had to rely on molecular modeling tools to try and shed light on the atomistic details of the binding process. Docking the most potent triazoles **12b**, **12c** into the cavity of one *Li*TryR monomer that lodges the ^435^Pro–Met^447^ α-helix of the other monomer, while feasible, failed to account for the SAR results discussed above. Indeed, the biologically essential naphthyl or biphenyl R_3_ substituents could not be properly accommodated unless the ligand folded onto itself in an unrealistic fashion due to lack of room. Therefore, we sought alternative binding sites in *Li*TryR, as found in Protein Data Bank (PDB) entry 2JK6(22b) using the FTMap web server.^43^ This procedure highlighted the existence of a large, hydrophobic, and putatively druggable cavity right at the center of the dimer that is lined by residues from both monomers and located very close to the interfacial helices that were mimicked by the original peptides and peptidomimetics (Figure 4).

**Figure 5:**
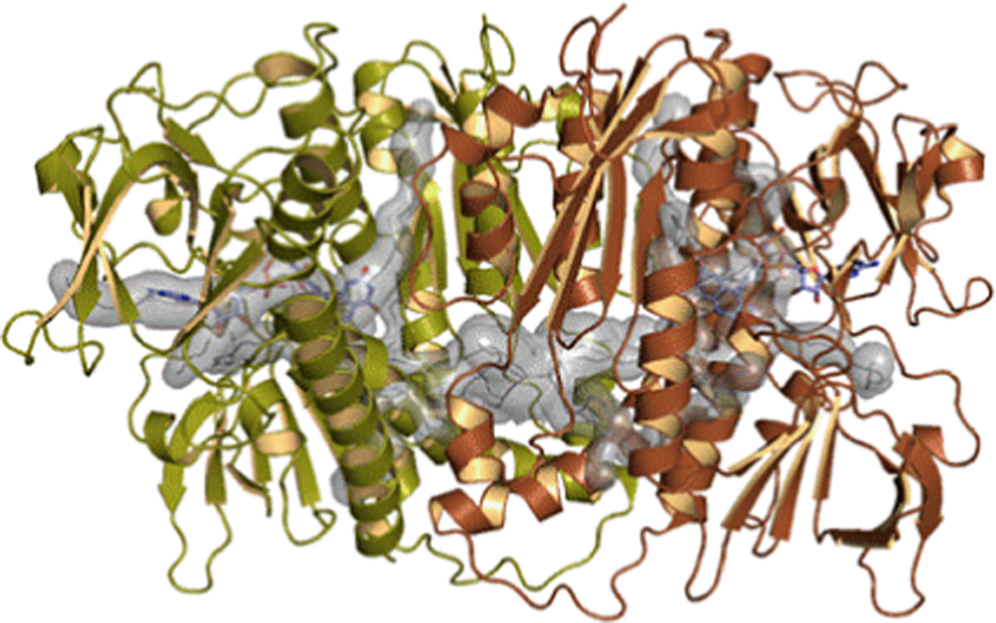
Visualization of the potential access routes from the bulk solvent to the interfacial cavity (2.808 Å^3^, 0.77 “druggability”) as determined by the CAVER Web server using default parameters.^48^ In comparison, each nicotinamide adenine dinucleotide phosphate (NADP) binding site occupies 1.343 Å^3^ and its druggability is 0.63. The tunnels are depicted as a continuous semitransparent gray surface. The central vertical α-helices encompass the amino acid residues originally identified as hotspots for dimerization. The FAD prosthetic groups are displayed as stick models for reference purposes only.

Interestingly, this central cavity at the *Li*TryR dimer interface is reminiscent of that present in *Plasmodium falciparum* GR^44^ or hGR and is shown to bind some noncompetitive or uncompetitive inhibitors such as safranin, menadione,^45^ xanthenes,^46^ or *N*-arylisoalloxazines.^47^ Ligand accessibility to this site was assessed by means of the CAVER web server,^48^ which identified several tunnels connecting this intermonomer cavity to the bulk solvent (Figure 5).

**Figure 6:**
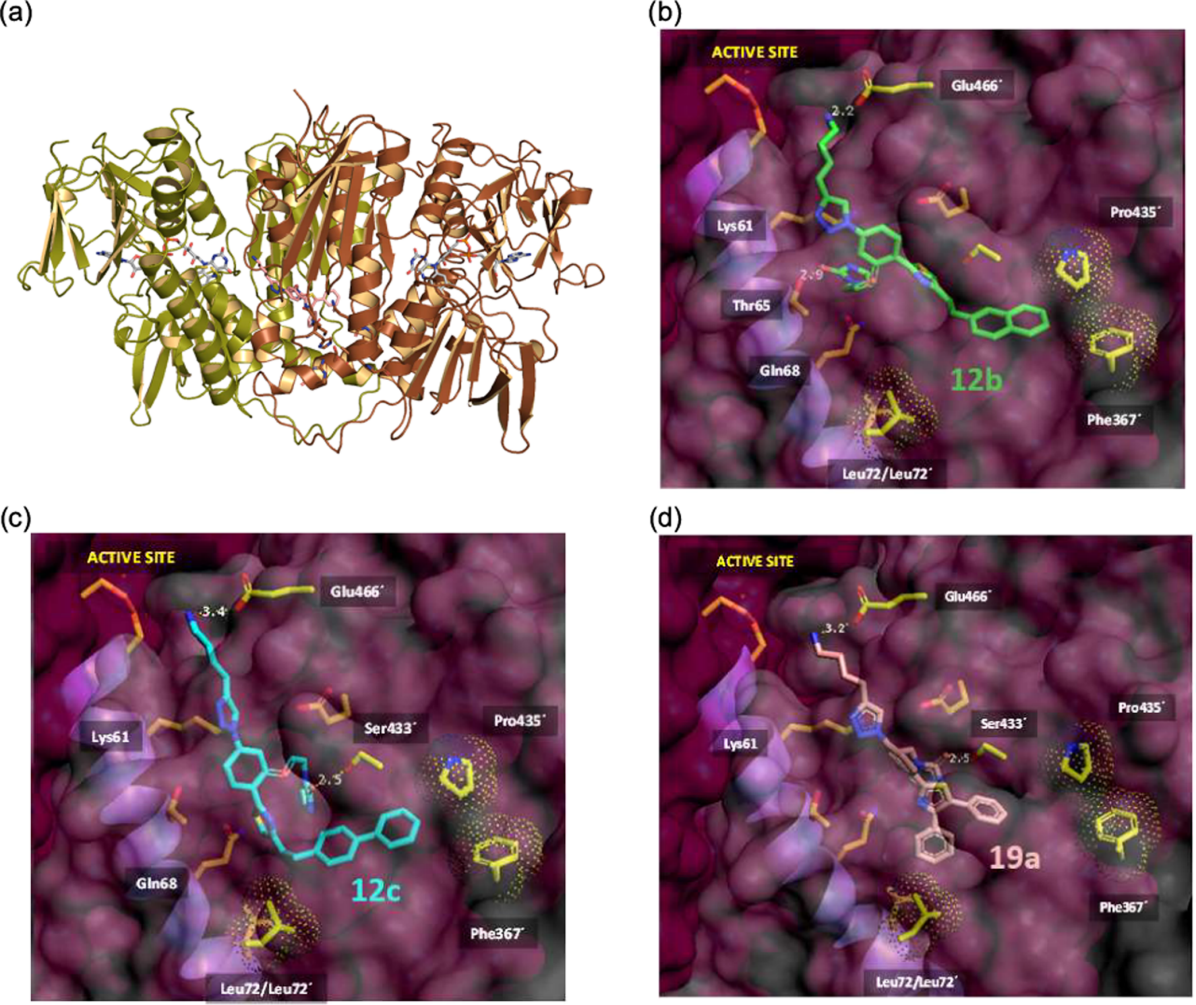
Proposed ligand-binding modes within *Li*TryR. (a) Cartoon representation of dimeric *Li*TryR showing the two FAD prosthetic groups (sticks, C atoms in gray) close to the active sites and a docked **19a** molecule (C atoms in pink) filling half of the central interfacial hydrophobic cavity and part of the tunnel connecting it to the bulk solvent. (b) Details of the best-scoring binding pose for **12b** (C atoms in green). (c) Details of the best-scoring binding pose for **12c** (C atoms in cyan). (d) Details of the best-scoring binding pose for **19a** (C atoms in pink).

We next performed docking studies with the most potent triazole dimerization inhibitors **12b**, **12c** and the disubstituted thiazole analogue **19a** centering the search within the proposed interfacial cavity of the *Li*TryR homodimer (Figure 6). The best ligand poses had in common the positioning of the (hetero)aromatic scaffold inside the connection tunnel and the terminal ammonium group of the R_1_ substituent at the hydrogen-bonding distance from the carboxylate of Glu466′ (*d*(N_**12b**_···O_Glu466′_) = 2.2 Å, *d*(N_**12c**_···O _Glu466′_) = 3.4 Å).

The proposed binding modes also revealed the potential of the imidazolidinone group of the R_2_ substituent to act as a hydrogen bond acceptor from the hydroxyl group of either Thr65 (*d*(N_**12b**_···O_Thr65_) = 2.9 Å), for the naphthyl compound **12b**, or Ser433′ (*d*(N_**12c**_···O_Ser433′_) = 2.5 Å), for biphenyl compounds **12c** and **19b**. The unhindered rotation of the central benzene ring of the scaffold seems to play a fundamental role to distinctly orient the R_2_ substituent in each complex. Regarding the R_3_ hydrophobic substituents at the thiazole, the bulky naphthyl and biphenyl aromatic groups for compounds **12b**, **12c** could be accommodated in one of the hydrophobic subpockets of the interfacial cavity that is mainly defined by Pro435′ and Phe367′, in good accord with the previous FTMap results. In addition, for the R_3_ and R_4_ disubstituted phenyl-thiazole analogue **19a**, one of the phenyl groups attached to the thiazole ring was found to point directly toward the second hydrophobic subpocket Leu72–Leu72′ at the bottom of the cavity, another location pinpointed by FTMap. This binding mode nicely accounts for the above-mentioned dramatic improvement of inhibitory potency relative to the monosubstituted phenyl-thiazole **12n**.

Our molecular dynamics simulations and binding energy decomposition results using **19a** as a representative inhibitor point to Glu436 as one of the major contributors to ligand binding (see Figure S16 and Movie S1 in the Supporting Information). We have previously shown that this residue is a hotspot for *Li*TryR dimerization.^25^ Indeed, in all X-ray crystal structures of TryR, this glutamate’s carboxylate from one monomer is involved in two short hydrogen bonds with the peptide backbone of Ser464 and Ala465 from the other monomer. Inhibitor binding appears to promote the loss of these interdimer hydrogen-bonding interactions (Figure S17) due to a wedge effect brought about by hydrophobic interactions involving the phenyl rings on the one side of the molecule and strong electrostatic interactions on the other side, which involve not only the free amino group but also the triazole ring. Indeed, the negative electrostatic potential generated by the two unsubstituted ring nitrogens faces the positive dipole of the short ^400^MetGly^405^ α-helix. Taken together, the theoretical calculations provide a rationale for the observed SAR results. Furthermore, the residue in hGR (PDB id: 1XAN) that is positionally equivalent to Leu72 in *Li*TryR is Phe78, whose aromatic side chain provides a flat platform for the stacking of ligands such as safranin or xanthene^45,46^ but prevents the binding of our novel compounds, hence their remarkable selectivity. The central cavity putatively targeted by **19a** and analogues is lined by the residues listed in Table S5, which also shows the positionally equivalent residues in hGR. The overall lack of identity in several crucial regions is in consonance with the observed marked selectivity for TryR.

**Figure 7:**
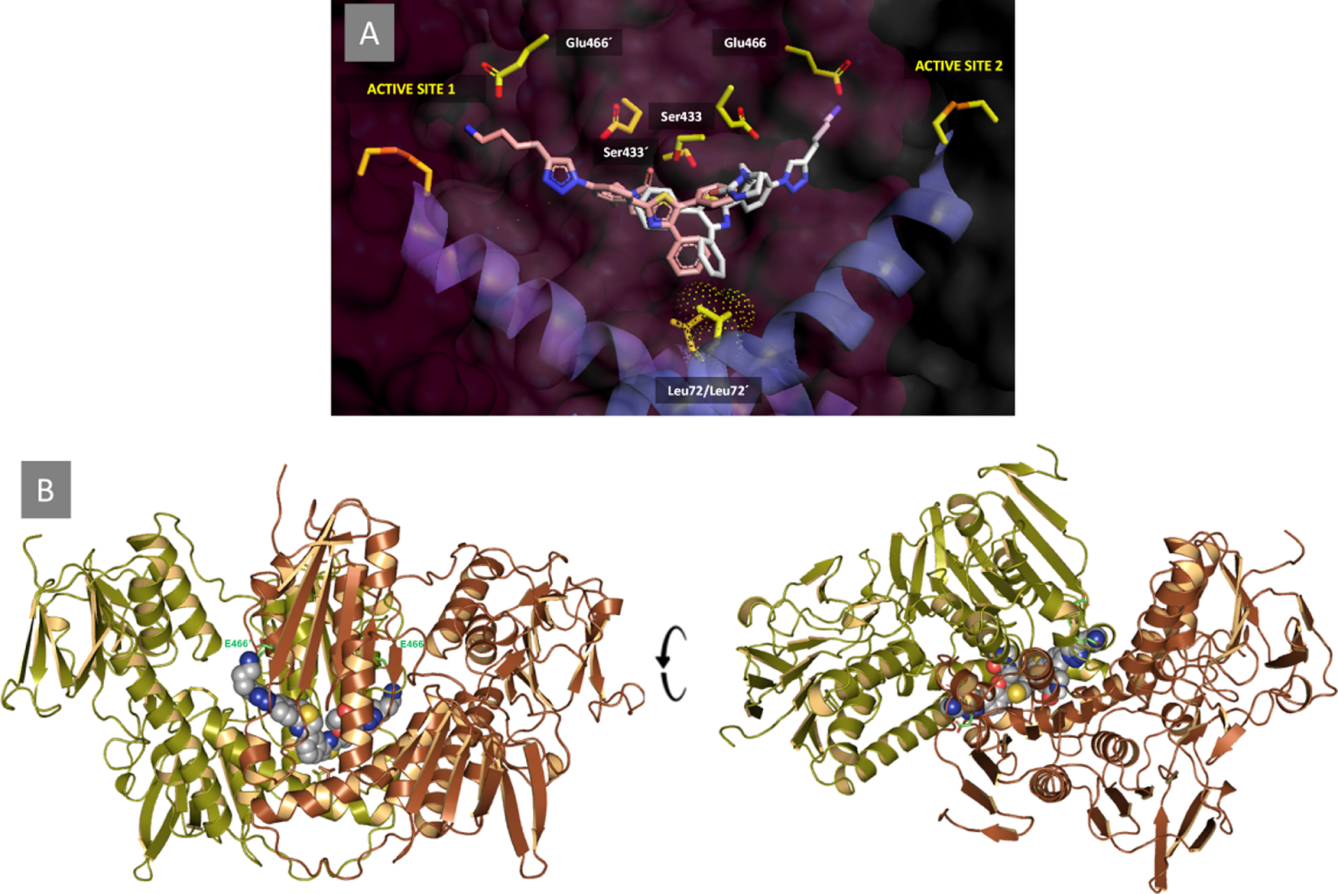
Rationale for the design of the *C*_2_-symmetric compound **22**. (A) Best docking pose for **19a** (C atoms in pink) with the tail extended into one *Li*TryR monomer reproduced for the other monomer (C atoms in white). Note how the propylamino group is extended and oriented toward the carboxylate of Glu466 in both cases. (B) Best-scoring result provided by the automated docking program^49^ for **22** (CPK model) inside the central interfacial cavity and connecting tunnels. The side chains of Glu466 and Glu466′ are displayed as sticks with C atoms colored in green.

To further support the feasibility of the proposed binding mode and improve the binding affinity for the putative target site, a new *C*_2_-symmetric compound **22** was rationally designed with a view to exploiting the *C*-2 symmetry of this homodimeric enzyme and simultaneously occupy both entrance/exit tunnels (Figure 5). The putative *Li*TryR/**19a** complex provided the starting point for the structure-based design. As shown in Figure 7A, reproducing the binding mode of **19a** within one monomer on the other monomer immediately reveals the possibility of merging both single molecular entities into one by making use of a suitable linker bonded to the R_3_ substituent. To this end, a 1,3-disubstituted phenyl was selected as an appropriate spacer to yield the *C*_2_-symmetric compound **22**, which was also docked into the putative binding site (Figure 7B).

The good agreement found between the intended binding mode for **22** and that found by the automated docking program^49^ (Figure 7) is noteworthy. In addition to the hydrophobic contacts between the aromatic rings and the walls of the cavity, the two terminal R_1_ butylammonium groups of **22** are presumed to establish simultaneous salt bridges with Glu466/Glu467 and Glu466′/Glu467′, whereas the carbonyl groups of the two imidazolidinone moieties can establish hydrogen bonds with the protonated nitrogens of Lys61/Lys61′, the side-chain carboxamide of Gln68, and the backbone NH of Ser433/Ser433′.

The activity of the new symmetric compound **22** was evaluated in the two enzymatic assays. Encouragingly, the compound emerged not only as the most effective *Li*TryR inhibitor of the entire series, with an IC_50_^act^ value of 0.4 ± 0.03 μM, but also as a very potent dimerization disruptor (IC_50_^dim^ = 8.0 ± 0.2 μM). In comparison with the corresponding monomeric triazole ligand **12n** (substituted with an R_3_ phenyl group), **22** was 28- and 4-fold more potent, respectively, in both assays (see Table 1). Taken together, these experimental results support the proposed binding mode of the monomeric and symmetric triazole-based compounds at this interfacial site.

### Inhibitory Activity on Other TryR Enzymes from the *Trypanosoma* Genus

TryR is an essential enzyme not only for *Leishmania* but also for *Trypanosoma* parasites. Given the high degree of identity/similarity among the amino acid sequences of TryRs from these two genera, some degree of activity against so closely related enzymes would not be unexpected for our compounds. To test this hypothesis, the most active members of these series were tested as inhibitors of the oxidoreductase activity of TryRs from *T. brucei* (*Tb*TryR) and *Trypanosoma congolense* (*Tco*TryR), the causative agents of sleep sickness and nagana, respectively. The results shown in Table 3 demonstrate, in fact, very similar activities for **12a**–**c**, **12n**, **12r**, **19a**, and **22** as inhibitors of the three enzymes.

**Table 3:** IC_50_ ± SE Values (μM) for Selected Triazole Analogues **12a**–**c**, **12n**, **12r**, **19a**, and **22** in the Oxidoreductase Activity of TryR from *L. infantum*, *T. brucei*, and *T. congolense*

compounds	*Li*TryR	*Tb*TryR	*Tco*TryR
**12a**	14.6 ± 1.0	13.3 ± 1.9	11.0 ± 1.3
**12b**	5.9 ± 1.1	9.7 ± 2.1	4.8 ± 1.9
**12c**	10.9 ± 1.8	10.0 ± 2.5	4.8 ± 1.8
**12n**	9.0 ± 0.2	12.5 ± 1.9	9.3 ± 0.8
**12r**	11.9 ± 0.6	15.0 ± 2.9	10.1 ± 1.4
**19a**	4.3 ± 1.0	7.3 ± 0.6	5.6 ± 0.8
**22**	0.4 ± 0.03	1.0 ± 0.3	0.5 ± 0.1

### *In Vitro* Antileishmanial Evaluation and Cytotoxicity

#### Compound Internalization into the Parasites

**Figure 8:**
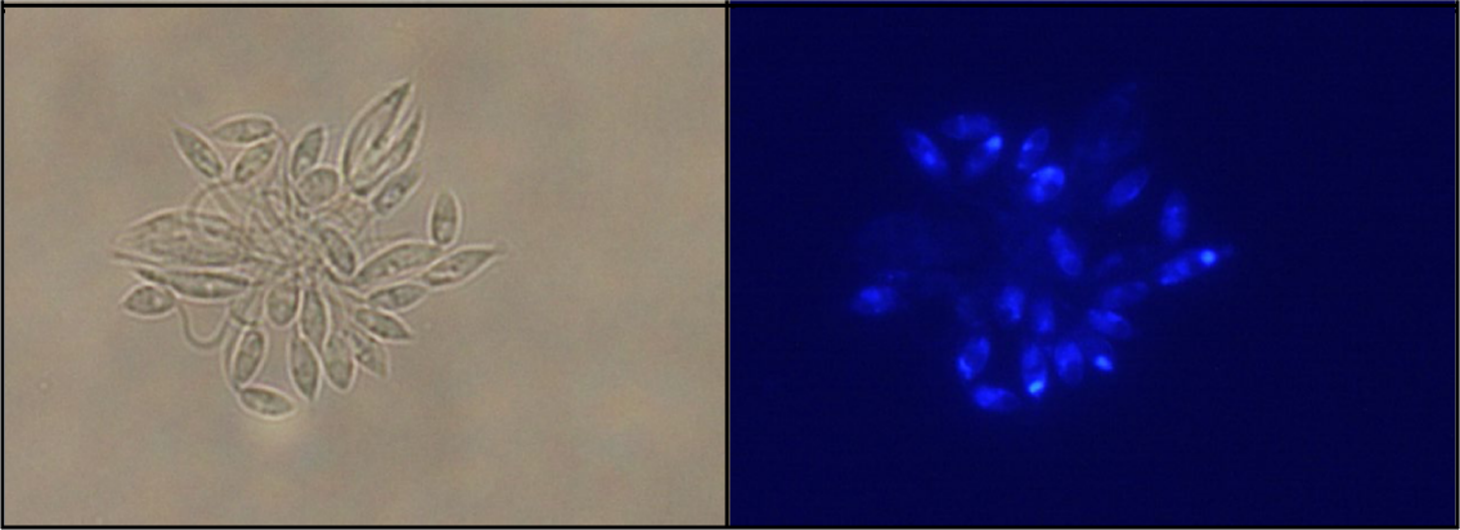
Fluorescence microscopy images of *L. infantum* promastigotes treated with 25 μM compound **12a** for 1 h.

Our earlier attempts to develop *Li*TryR dimerization disruptors as leishmanicidal molecules were hampered by the poor ability of previously synthesized compounds to cross the parasite plasma membrane. The peptidomimetics herein described, endowed with fluorescent properties, no longer suffer from this limitation. Figure 8 shows the intense blue fluorescence emitted by promastigotes incubated for 1 h with compound **12a**. Strong fluorescent spots can be observed in the apical region of some parasites, just opposite the flagellar pocket that is expected to be the main entry region for these compounds.

#### Activity on Extracellular Parasites and Human Cells

Representative examples of triazole-based *Li*TryR enzyme inhibitors were tested *in vitro* against *L. infantum* axenic promastigotes and amastigotes. Cytotoxicity was assessed employing the human liver cancer cell line HepG2. Miltefosine, the R_9_ conjugate of the prototype peptide **1**, and imidazole-based compounds **2** and **3** were included as reference drugs. According to the data shown in Table 4, among the 18 triazole compounds tested, 9 showed half-maximal effective concentration (EC_50_) values below 10 μM against *Leishmania* promastigotes or amastigotes (**12b**, **12c**, **12i–k**, **12n**, **19b**, **19d**, and **19e**) that are similar to those observed for the reference compounds.

**Table 4:** *In Vitro* Activity on *L. infantum* Parasites and Cytotoxic Activity of Representative Triazole Compounds^a^^,^^b^

compounds	EC_50_ (μM) promastigotes^c^	EC_50_ (μM) amastigotes^c^	EC_50_ (μM) HepG2^c^^,^^d^	SI_p_/SI_a_^e^
**12a**	16.4 ± 0.9	13.9 ± 0.6	20.3 ± 2.4	1.2/1.5
**12b**	8.4 ± 0.3	15.3 ± 1.7	33.3 ± 2.7	4.0/2.2
**12c**	4.9 ± 0.4	11.6 ± 0.8	43.1 ± 2.6	8.8/3.7
**12h**	12.7 ± 0.3	31.6 ± 5.8	25.7 ± 2.2	2.0/<1
**12i**	5.8 ± 0.7	5.2 ± 0.3	60.4 ± 3.0	10.4/11.6
**12j**	8.5 ± 0.1	7.1 ± 0.7	12.4 ± 0.7	1.8/1.7
**12k**	ND^f^	4.0 ± 0.2	7.4 ± 0.6	ND/1.9
**12m**	42.6 ± 3.9	25.2 ± 0.5	44.9 ± 1.3	1.0/1.8
**12n**	5.4 ± 0.08	11.5 ± 0.3	16.4 ± 2.3	3.0/1.4
**12o**	>75	>75	67.9 ± 1.5	<1.0/<1.0
**12q**	ND	27.0 ± 2.8	43.3 ± 1.0	ND/1.6
**12r**	23.8 ± 0.5	22.3± 0.6	29.4 ± 0.7	1.2/1.3
**12s**	ND	68.8 ± 3.2	>75	ND/>1.1
**19a**	10.7 ± 1.3	15.6 ± 3.0	26.5 ± 4.1	2.5/1.7
**19b**	ND	9.4 ± 0.5	17.3 ± 0.1	ND/1.8
**19c**	ND	22.6 ± 5.7	≥ 75	ND/≥3.3
**19d**	ND	9.1 ± 0.2	12.1 ± 0.1	ND/1.3
**19e**	ND	8.4 ± 0.1	6.6 ± 0.6	ND/<1
**22**	3.8 ± 0.1	4.9 ± 0.2	30.5 ± 4.1	8.0/6.2
miltefosine	47.6 ± 0.6	2.0 ± 0.1	>75	>1.6/>37.5
R_9_-peptide **1**	3.1 ± 0.6	3.4 ± 0.2	61.7 ± 6.1	19.9/18.1
**2**	12.8 ± 0.7	12.8 ± 1.3	27.1 ± 2.4	2.1/2.1
**3**	5.3 ± 0.3	5.3 ± 0.2	14.2 ± 0.9	2.7/2.7

a Miltefosine, linear peptide **1** conjugated to R_9_, and imidazole-containing compounds **2** and **3** were included as in-house reference compounds.

b Results are representative of three independent experiments, each performed in triplicate. EC_50_ ± SE values are indicated.

c The half-maximal effective concentration (EC_50_) is defined as that causing a 50% reduction of proliferation in *L. infantum* promastigotes (EC_50p_) and amastigotes (EC_50a_) or human hepatocellular carcinoma (HepG2) cells. Dead parasites were identified by their increased permeability to propidium iodide (PI).

d Cytotoxicity in HepG2 cells was measured using the crystal violet method.

e The selectivity index (SI) is the ratio of EC_50_ against human cells to EC_50_ against *L. infantum* promastigotes (SI_p_) and amastigotes (SI_a_).

f ND = not determined.

Of all the compounds bearing a butylamine at R_1_, an imidazolidinone at R_2_, and different R_3_ substituents (**12a**–**c**), the biphenylethyl analogue **12c** displayed better activity against both promastigotes and amastigotes than those containing naphthyl or phenylethyl substituents (**12c** > **12b** > **12a**) and also lower cytotoxicity. This results in an improved selectivity index (SI) of 8.8 for promastigotes (SI_p_) and 3.7 for amastigotes (SI_a_) in comparison with the SIs of the corresponding biphenyl imidazole analogue **3** (SI_p_ = SI_a_ = 2.7). On the other hand, a combination of a shorter propyl spacer at R_1_ and the biphenyl group at R_3_ gave **12i**, the most potent and selective compound of the entire series, with EC_50_ values of 5.8 against promastigotes (EC_50p_) and 5.2 μM against amastigotes (EC_50a_) and the highest SIs (SI_p_ = 10.4 and SI_a_ = 11.6). The imidazolidinone moiety at R_2_ could be eliminated without compromising the leishmanicidal activity (**12j**, **12k**), but a significant increase in cytotoxicity was detected, which suggests a positive effect of the imidazolidinone on reducing the cytotoxicity. As regards R_3_, the biphenylethyl substituent could be replaced by a phenyl resulting in an equipotent leishmanicidal analogue (**12c***vs***12n**), in contrast to the higher IC_50_ values observed for **12n** in the enzymatic assays. However, the SI for **12n** was low due to an increase in toxicity. The introduction of polyheteroaromatic rings gave rise to a significant reduction of the leishmanicidal activity (**12q**–**s***vs***12c**) in contrast to the high potency observed for these analogues in the enzymatic assays. Disubstituted thiazole analogues **19a**–**e** also showed high leishmanicidal activities against amastigotes with EC_50_ values in the 8.4−22.6 μM range but, with the exception of **19c**, low SIs (SI < 2.5) due to higher toxicities. Remarkably, the symmetrical triazole analogue **22** turned out to be one of the most potent and selective agents against *L. infantum* amastigotes with an EC_50a_ value of 4.9 μM and a SI_a_ of 6.2.

It should be noted that a direct correlation between *in vitro* TryR inhibitory activity and *in cellulo* effects is not to be expected due to differences in cellular permeability or the possibility that this enzyme is not the only target for these compounds. Therefore, among the newly tested triazole-based analogues, the propylamine biphenyl derivative **12i** and the *C*-2 symmetric **22** displayed the best leishmanicidal activity and the lowest *in cellulo* cytotoxicity with SI values that exceeded those obtained for the imidazoles **2** and **3** by 5–6-fold. It is interesting to point out that, despite the relatively poor IC_50_ value obtained for **12i** in the oxidoreductase activity assay, its behavior in the dimerization assay is similar to that of the most potent compounds from these series. This finding suggests that their ability to disrupt the dimer may be largely responsible for the leishmanicidal activity of these compounds. As IC_50_ values were calculated taking into consideration only the initial velocities of the reactions and because of the time-dependent behavior of these molecules, compounds such as **12i** may show good activities in the dimerization assay while displaying poor IC_50_ values.

#### Leishmanicidal Activity in Intracellular Amastigotes

The most potent and selective compounds were further tested for their leishmanicidal activity in amastigote-infected THP-1 cells. The EC_50_ values obtained are summarized in Table 5. Miltefosine, edelfosine, amphotericin B, the R_9_-conjugate of the prototype peptide **1**, and compounds **2** and **3** from the precursor imidazole series were included as reference compounds. In our view, the fact that the EC_50_ values cover a range from 22 to >75 μM indicates that not all of the compounds are able to cross the plasma and phagolysosomal membranes in the macrophages. Among the monomeric triazole-based compounds, only the phenyl derivative **12a**, with an EC_50_ value of 32.4 μM, and the biphenyl derivatives **12c**, **12i**, with EC_50_ values of 46.5 and 54.5 μM, respectively, displayed moderate effectiveness against intracellular amastigotes. Interestingly, the symmetric triazole molecule **22** emerged, once again, as the most potent and selective leishmanicidal compound of all of the triazole-based series (EC_50_ = 22 μM), showing a SI of 2.2, better than that obtained for edelfosine (SI = 1.4). Accordingly, derivatives **12a**, **12c**, **12i**, and **22** constitute the first analogues designed against the dimerization interface of TryR that show leishmanicidal activity toward intracellular amastigotes. These results confirm the interest and potential of both monomeric and symmetric triazole-based compounds as antileishmanial agents.

**Table 5:** *In Vitro* Antileishmanial Activity for the Most Representative Triazole-Based Compounds in Amastigote-Infected THP-1 Monocyte-Derived Macrophages^a^

compounds	EC_50_ (μM) intracellular amastigotes^b^	EC_50_ (μM) THP-1^c^
**12a**	32.4 ± 6.7	45 ± 3.1
**12b**	>75	>75
**12c**	46.5 ± 1.5	>75 (100% at 75 μM)^d^
**12i**	54.4 ± 15.5	>75 (100% at 75 μM)^d^
**12q**	>75	>75 (67% at 75 μM)^d^
**19c**	>75	>75 (63% at 75 μM)^d^
**22**	22.2 ± 6.4	48.4 ± 14.5
mdelfosine	2.5 ± 0.02	3.5 ± 0.3
miltefosine	0.6 ± 0.1	>25
amphotericin B	0.07 ± 0.01	>5
R_9_-peptide **1**	>75	14.5 ± 2.9
**2**	>75	27.9 ± 4.4
**3**	>75	15.5 ± 1.8

a Edelfosine, miltefosine, and amphotericin B were included as reference leishmanicidal drugs. The R_9_-peptide **1** and the imidazole-containing compounds **2** and **3** were included as in-house reference compounds for structure–activity comparisons.

b The half-maximal effective concentration (EC_50_) is defined as that causing a 50% reduction in the number of *L. infantum* amastigotes per cell in the culture or amastigote-infected THP-1 monocyte-derived macrophages. Living intracellular amastigotes were identified by their green fluorescence due to green fluorescent protein (GFP) expression and their lack of permeability to propidium iodide; EC_50_ ± SE values are indicated.

c Cytotoxicity was assessed in THP-1 monocyte-derived macrophages by measuring lactate dehydrogenase (LDH) activity.

d Cell viability of amastigote-infected THP-1 monocyte-derived macrophages at 75 μM (maximum concentration assayed). Results are representative of three independent experiments.

## Conclusions

The development of new and more efficient antileishmanial drugs is a priority in the field of neglected tropical diseases. From a small library of 26 triazole-phenyl-thiazole compounds, we have identified the most potent small-molecule dimerization disruptors of *Li*TryR described to date. Similar to prototype peptide **1**, they behave as slow-binding, noncompetitive inhibitors with overall *K*_i_* values of 0.5 μM (**19a**). These molecules are also endowed with high antileishmanial activity against both extracellular and intracellular parasites and improved selectivity indexes when compared to those obtained for previous in-house imidazole-based compounds.

Molecular modeling studies carried out for the most potent *Li*TryR triazole-containing dimerization inhibitors **12b**, **12c**, and **19a** identified a previously unexplored but apparently druggable binding site consisting of a large central cavity at the dimer interface of the enzyme. In the absence of crystallographic evidence, despite extensive efforts, this putative binding mode provided a rationale for the observed SAR and a basis for the subsequent structure-based design of a new symmetrical triazole analogue **22** that displays improved *Li*TryR inhibitory activities over those of its parental monomeric triazole. This rationally designed compound shows a dramatic enhancement in leishmanicidal potency, in particular against intracellular amastigotes.

All in all, our results provide the first evidence for ligand binding to a so-far unexplored region of *Li*TryR and lay the ground for further design of innovative TryR inhibitors that may become valuable candidates in the process of discovering new drugs against leishmaniasis.

## Experimental Section

### Chemical Procedures

Experiments dealing with air- and water-sensitive reagents/compounds were performed in an argon atmosphere. Hygroscopic species were previously dried under vacuum for 24 h by P_2_O_5_ as the drying agent. Unless otherwise noted, analytical-grade solvents and commercially available reagents were used without further purification. Anhydrous CH_2_Cl_2_ was dried by refluxing over CaH_2_. EtOH was dried with 4 Å molecular sieves previously activated in a microwave oven. THF was dried by reflux over sodium/benzophenone. Pressured reactions were carried out on a Sigma-Aldrich Ace Glass flask (50 or 100 mL) supplied with a Teflon cap. Microwave-assisted experiments were performed on a Biotage Initiator 2.0 single-mode cavity instrument from Biotage (Uppsala). Experiments were carried out in sealed microwave reaction vials at the standard absorbance level (400 W maximum power). Lyophilizations were carried out on a Telstar 6-80 lyophilizer.

Monitoring of the reactions was performed by analytical thin-layer chromatography (TLC) on silica gel 60 F_254_ (Merck) or by high-performance liquid chromatography coupled to mass spectrometry (HPLC–MS) using a Waters Alliance 2695 Separation Module with a Waters Micromass ZQ Detector.

Compounds were purified by (a) flash column chromatography (Merck) with silica gel 60 (230–400 mesh); (b) high-performance flash column chromatography (HPFC) on a Biotage Isolera One using cartridges of Claricep i-Series, Silica, 40 g (40–60 μm, 60 Å) for normal phase chromatography, and KP-C18-HS 12g (21 × 55 mm^2^) for the reverse phase; (c) centrifugal circular thin-layer chromatography (CCTLC) on a Chromatotron (Kiesegel 60 PF_254_ gipshaltig, Merck), with layer thicknesses of 1 and 2 mm and flow rates of 2 and 3 mL/min; or (d) semipreparative HPLC purifications on a Waters 2695 HPLC system equipped with a Photodiode Array 2998 coupled to a 3100 Mass Detector mass spectrometer using a C18 Sunfire column (19 mm × 150 mm).

The purity of the compounds was checked by analytical HPLC on a Waters 600 system equipped with a C18 Sunfire column (4.6 mm × 150 mm, 3.5 μm) and a selectable wavelength UV detector. Alternatively, an Agilent Technologies 1120 Compact LC provided with a reverse-phase column ACE 5 C18-300 (4.6 mm × 150 mm, 3.5 μm) and integrated diode detector PDA 996 was used. As the mobile phase, mixtures of A (CH_3_CN) and B (H_2_O) (0.05% TFA) on isocratic or gradient mode with a flow rate of 1 mL/min were used. The following gradients were used: from 10% A to 100% A in 10 min (gradient A) and from 1% A to 100% A in 10 min (gradient B). The purity of the final compounds was also determined to be >95% by elemental analysis with a LECO CHNS-932 apparatus. Deviations of the elemental analysis results from the calculated value were within ±0.4%.

Melting points (m.p.) were measured on a Mettler Toledo M170 apparatus and are uncorrected. Infrared (IR) spectra were recorded on a PerkinElmer Spectrum One instrument and KBr pellet as a sample support. Mass spectrum (MS) on electrospray ionization (ESI) mode was recorded on a Hewlett-Packard 1100SD in positive mode. High-resolution mass spectra (HRMS) were obtained on an Agilent 6520 Accurate-Mass Q-TOF LC-MS equipped with an HP-1200 (Agilent) liquid chromatograph coupled to a mass spectrometer with a Q-TOF 6520 hybrid mass analyzer. Usually, an error equal to or lower than 5 ppm was allowed. Nuclear magnetic resonance (NMR) spectra were recorded on a Varian INNOVA-300 operating at 300 MHz (^1^H) and 75 MHz (^13^C), a Varian INNOVA-400 operating at 400 MHz (^1^H) and 100 MHz (^13^C), a Varian MERCURY-400 operating at 400 MHz (^1^H) and 100 MHz (^13^C), and a Varian SYSTEM-500 operating at 500 MHz (^1^H) and 125 MHz (^13^C). The assignments were performed by means of different standard homonuclear and heteronuclear correlation experiments (gradient heteronuclear single quantum coherence (gHSQC), gradient heteronuclear multiple bond coherence (gHMBC), and nuclear Overhauser enhancement spectroscopy (NOESY)) when required.

#### 4-Azido-2-(2-(2-oxoimidazolidin-1-yl)ethoxy)benzo-nitrile (**5a**)

A solution of bromoarene **4a**(30) (500 mg, 1.61 mmol) and NaN_3_ (1.57 g, 24.2 mmol) in anhydrous DMSO (30 mL) in the presence of 4 Å molecular sieves, and under an argon atmosphere, was heated at 100 °C for 72 h. The reaction was allowed to cool to room temperature and diluted with H_2_O (60 mL), and the aqueous phase was extracted with CH_2_Cl_2_ (3 × 50 mL). The combined organic layers were dried (Na_2_SO_4_), filtered, evaporated to dryness, and lyophilized. The residue was purified by flash column chromatography (CH_2_Cl_2_/MeOH, 100:3) to give 372 mg (85%) of **5a** as a yellow solid; m.p.: 158–161 °C; IR (KBr), ν (cm^–1^): 3230 (NH-st), 3090 (C_sp_–H st), 2223 (C≡N st), 2125 (N=N=N st), 1702 (C=O st); ^1^H NMR (CDCl_3_, 300 MHz) δ (ppm): 7.53 (d, *J* = 8.3 Hz, 1H, Ar), 6.70 (dd, *J* = 8.3, 2.0 Hz, 1H, Ar), 6.53 (d, *J* = 1.8 Hz, 1H, Ar), 4.71 (bs, 1H, NHCON), 4.20 (t, *J* = 4.7 Hz, 2H, OCH_2_), 3.81–3.76 (m, 2H, CH_2_CH_2_NHCON), 3.64 (t, *J* = 4.9 Hz, 2H, OCH_2_CH_2_), 3.44 (t, *J* = 8.1 Hz, 2H, CH_2_CH_2_NHCON); ^13^C NMR (CDCl_3_, 75 MHz) δ (ppm): 162.8 (NHCON), 161.8 (OC_Ar_), 146.8 (C_Ar_), 134.9 (CH_Ar_), 116.2 (CN), 111.8 (CH_Ar_), 103.2 (CH_Ar_), 98.3 (C_Ar_), 69.4 (OCH_2_), 47.8 (CH_2_CH_2_NHCON), 43.2 (OCH_2_CH_2_), 38.6 (CH_2_CH_2_NHCON); MS (ESI, positive mode) *m*/*z*: 567.2 [2M + Na]^+^, 295.0 [M + Na]^+^, 273.0 [M + H]^+^. NaN_3_ may be toxic and explosive. Thus, for safety precautions, a polycarbonate safety screen in a properly functioning fume hood was always used to perform this reaction.

#### Benzyl-4-(1-(4-cyano-3-((1-(2-oxoimidazolidin-1-yl)ethoxy)phenyl)-1*H*-1,2,3-triazol-4-yl)butyl)carbamate (**8**)

To a solution of azide **5a** (450 mg, 1.65 mmol), commercially available benzyl-5-hexynylcarbamate (523 mg, 2.15 mmol), and CuSO_4_·5H_2_O (42 mg, 0.17 mmol) in EtOH (25 mL) were added sodium ascorbate (131 mg, 0.66 mmol) and H_2_O (25 mL). The reaction mixture was stirred at room temperature in the dark overnight. The mixture was evaporated to dryness, and the residue dissolved in CH_2_Cl_2_ (50 mL) was washed with H_2_O (3 × 50 mL), dryed (Na_2_SO_4_), filtered, and evaporated under reduced pressure. The residue was purified by flash column chromatography (CH_2_Cl_2_/MeOH, 100:3) to provide **8** (573 mg, 69%) as a colorless oil. ^1^H NMR (CDCl_3_, 400 MHz) δ (ppm): 7.86 (s, 1H, Ar), 7.61 (d, *J* = 8.3 Hz, 1H, Ar), 7.49 (s, 1H, Ar), 7.34 (d, *J* = 7.1 Hz, 1H, Ar), 7.32–7.17 (m, 5H, Ar), 5.05 (bs, 1H, NHCbz), 5.02 (s, 2H, NHCOOCH_2_), 4.68 (bs, 1H, NHCON), 4.26 (t, *J* = 5.2 Hz, 2H, OCH_2_), 3.66 (t, *J* = 7.9 Hz, 2H, CH_2_CH_2_NHCON), 3.57 (t, *J* = 5.2 Hz, 2H, OCH_2_CH_2_), 3.33 (t, *J* = 8.3 Hz, 2H, CH_2_CH_2_NHCON), 3.18 (q, *J* = 6.7 Hz, 2H, CH_2_NHCbz), 2.76 (t, *J* = 7.3 Hz, 2H, TrizCH_2_), 1.71 (quin, *J* = 7.4 Hz, 2H, TrizCH_2_CH_2_), 1.52 (quin, *J* = 7.2 Hz, 2H, CH_2_CH_2_NHCbz); ^13^C NMR (CDCl_3_, 75 MHz) δ (ppm): 162.8 (NHCON), 161.5 (OC_Ar_), 156.6 (NHCOO), 149.2 (C_Ar_), 141.5 (C_Ar_), 136.6 (CH_Ar_), 135.1 (CH_Ar_), 128.6 (CH_Ar_), 128.2 (CH_Ar_), 119.1 (CH_Ar_), 115.7 (CN), 112.1 (CH_Ar_), 104.1 (CH_Ar_), 101.5 (C_Ar_), 69.0 (OCH_2_), 66.7 (NHCOOCH_2_), 47.3 (CH_2_CH_2_NHCON), 42.8 (OCH_2_CH_2_), 40.8 (CH_2_NHCbz), 38.5 (CH_2_CH_2_NHCON), 29.4 (CH_2_CH_2_NHCbz), 26.1 (TrizCH_2_CH_2_), 25.1 (TrizCH_2_); HRMS (ES, positive mode) *m*/*z*: calcd for C_26_H_29_N_7_O_4_ 503.2281; found 503.2279 (−0.33 ppm).

#### Benzyl-4-(1-(4-carbamothioyl-3-((1-(2-oxoimidazolidin-1-yl)ethoxy)phenyl)-1*H*-1,2,3-triazol-4-yl)butyl)carbamate (**9**)

To a solution of benzonitrile **8** (400 mg, 0.79 mmol) in DMF (25 mL), 20% aq (NH_4_)_2_S (3.78 mL, 55.6 mmol) was added and then heated to 80 °C for 4 h. The reaction mixture was allowed to cool to room temperature, and then, CH_2_Cl_2_ (50 mL) was added. The mixture was successively washed with HCl 0.1 N (2 × 50 mL), H_2_O (1 × 50 mL), and brine (1 × 50 mL). The organic layers were dried (Na_2_SO_4_), filtered, and evaporated to dryness. The crude was purified by flash column chromatography (CH_2_Cl_2_/MeOH, 100:5) to give **9** (354 mg, 74%) as a yellow oil. ^1^H NMR (CDCl_3_, 400 MHz) δ (ppm): 9.63 (bs, 1H, SCNH_2_), 9.50 (bs, 1H, SCNH_2_), 8.71 (d, *J* = 8.7 Hz, 1H, Ar), 7.78 (s, 1H, Ar), 7.45 (d, *J* = 2.0 Hz, 1H, Ar), 7.36–7.17 (m, 5H, Ar), 7.10 (dd, *J* = 8.7, 2.0 Hz, 1H, Ar), 5.96 (bs, 1H, NHCbz), 5.02 (s, 2H, NHCOOCH_2_), 4.98 (bs, 1H, NHCON), 4.16 (t, *J* = 4.4 Hz, 2H, OCH_2_), 3.64 (t, *J* = 4.4 Hz, 2H, OCH_2_CH_2_), 3.50 (dd, *J* = 9.7, 6.7 Hz, 2H, CH_2_CH_2_NHCON), 3.40 (dd, *J* = 9.1, 6.2 Hz, 2H, CH_2_CH_2_NHCON), 3.17 (q, *J* = 6.7 Hz, 2H, CH_2_NHCbz), 2.73 (t, *J* = 7.5 Hz, 2H, TrizCH_2_), 1.70 (quin, *J* = 7.5 Hz, 2H, TrizCH_2_CH_2_), 1.53 (quin, *J* = 7.5 Hz, 2H, CH_2_CH_2_NHCbz); ^13^C NMR (CDCl_3_, 75 MHz) δ (ppm): 196.5 (SCNH_2_), 163.5 (NHCON), 156.3 (NHCOO), 149.1 (C_Ar_), 140.3 (C_Ar_), 138.4 (CH_Ar_), 136.7 (CH_Ar_), 128.7 (CH_Ar_), 128.2 (CH_Ar_), 125.1 (C_Ar_), 119.1 (CH_Ar_), 111.3 (CH_Ar_), 104.0 (CH_Ar_), 66.8 (OCH_2_), 66.6 (NHCOOCH_2_), 45.4 (CH_2_CH_2_NHCON), 43.2 (OCH_2_CH_2_), 40.9 (CH_2_NHCbz), 38.3 (CH_2_CH_2_NHCON), 29.8 (CH_2_CH_2_NHCbz), 26.4 (TrizCH_2_CH_2_), 25.3 (TrizCH_2_); MS (ESI, positive mode) *m*/*z*: 538.3 [M + H]^+^.

#### General Procedure for the Synthesis of Thiazoles **11a**–**c** by Hantzsch Cyclization

A solution of thioamide **9** (1 equiv) in ^*i*^PrOH (15 mL) was treated with the appropriated α-haloketone (**10a**–**c**) (1 equiv). The reaction mixture was stirred at 70 °C for 3–6 h in a pressure flask, and then, it was allowed to cool to room temperature and concentrated to dryness under reduced pressure. The residue was purified by flash column chromatography or CCTLC on a Chromatotron (eluents are specified in each case).

##### Benzyl-(4-(1-(3-(2-(2-oxoimidazolidin-1-yl)ethoxy)-4-(4-phenethylthiazol-2-yl)phenyl)-1*H*-1,2,3-triazol-4-yl)butyl)carbamate (**11a**)

Following the general procedure, thioamide **9** (100 mg, 0.19 mmol) and the commercially available 1-bromo-4-phenylbutan-2-one (**10a** 42 mg, 0.19 mmol) in ^*i*^PrOH (15 mL) reacted at 70 °C for 4 h. After the workup, the residue was purified by CCTLC on the Chromatotron (CH_2_Cl_2_/MeOH, 100:3) to yield 108 mg (87%) of **11a** as a colorless oil. ^1^H NMR (CDCl_3_, 400 MHz) δ (ppm): 8.46 (d, *J* = 8.5 Hz, 1H, Ar), 7.81 (s, 1H, Ar), 7.51 (d, *J* = 2.0 Hz, 1H, Ar), 7.38–7.06 (m, 11H, Ar), 6.86 (s, 1H, Ar), 5.06 (t, *J* = 6.0 Hz, 1H, NHCbz), 5.01 (s, 2H, NHCOOCH_2_), 4.34 (t, *J* = 5.7 Hz, 2H, OCH_2_), 3.69 (t, *J* = 5.7 Hz, 2H, OCH_2_CH_2_), 3.50 (dd, *J* = 9.0, 6.7 Hz, 2H, CH_2_CH_2_NHCON), 3.26 (t, *J* = 7.9 Hz, 2H, CH_2_CH_2_NHCON), 3.18 (q, *J* = 7.4 Hz, 2H, CH_2_NHCbz), 3.12–2.97 (m, 4H, CH_2_CH_2_Ph), 2.76 (t, *J* = 7.4 Hz, 2H, TrizCH_2_), 1.71 (quin, *J* = 7.4 Hz, 2H, TrizCH_2_CH_2_), 1.59 (quin, *J* = 7.1 Hz, 2H, CH_2_CH_2_NHCbz); ^13^C NMR (CDCl_3_, 75 MHz) δ (ppm): 162.8 (NHCON), 160.3 (OC_Ar_), 156.6 (C_Ar_), 156.0 (C_Ar_), 155.9 (C_Ar_), 148.7 (C_Ar_), 141.7 (C_Ar_), 138.3 (C_Ar_), 136.7 (C_Ar_), 130.0 (CH_Ar_), 128.6 (CH_Ar_), 128.6 (CH_Ar_), 128.4 (CH_Ar_), 128.2 (CH_Ar_), 126.1 (CH_Ar_), 122.7 (C_Ar_), 119.1 (CH_Ar_), 115.1 (CH_Ar_), 112.4 (CH_Ar_), 104.5 (CH_Ar_), 67.8 (OCH_2_), 66.7 (NHCOOCH_2_), 46.4 (CH_2_CH_2_NHCON), 43.0 (OCH_2_CH_2_), 40.8 (CH_2_NHCbz), 38.4 (CH_2_CH_2_NHCON), 35.6 (CH_2_CH_2_Ph), 33.4 (CH_2_CH_2_Ph), 29.4 (CH_2_CH_2_NHCbz), 26.4 (TrizCH_2_CH_2_), 25.2 (TrizCH_2_); HRMS (ES, positive mode) *m*/*z*: calcd for C_36_H_39_N_7_O_4_S 665.2784; found 665.2791 (1.00 ppm).

##### Benzyl-(*E*)-(4-(1-(4-(4-(2-(naphthalen-2-yl)vinyl)thiazol-2-yl)-3-(2-(2-oxoimidazolidin-1-yl)ethoxy)phenyl)-1*H*-1,2,3-triazol-4-yl)butyl)carbamate (**11b**)

According to the general procedure, thioamide **9** (520 mg, 0.97 mmol) and α-bromoketone **10b**(30) (267 mg, 0.97 mmol) were reacted at 70 °C for 5 h in *^i^*PrOH. After the workup, the residue was purified by flash column chromatography (CH_2_Cl_2_/MeOH, 100:4) to give **11b** (365 mg, 51%) as a yellow oil. ^1^H NMR (CDCl_3_, 400 MHz) δ (ppm): 8.61 (d, *J* = 8.5 Hz, 1H, Ar), 7.90–7.64 (m, 7H, Ar, ThiazCH=CH), 7.55 (s, 1H, Ar), 7.45–7.36 (m, 3H, Ar), 7.30–7.14 (m, 7H, Ar, ThiazCH=CH), 5.03 (s, 2H, NHCOOCH_2_), 4.97 (bs, 1H, NHCON), 4.48 (bs, 1H, NHCbz), 4.39 (t, *J* = 5.7 Hz, 2H, OCH_2_), 3.73 (t, *J* = 5.7 Hz, 2H, OCH_2_CH_2_), 3.52 (dd, *J* = 9.0, 6.6 Hz, 2H, CH_2_CH_2_NHCON), 3.28 (t, *J* = 7.9 Hz, 2H, CH_2_CH_2_NHCON), 3.20 (q, *J* = 6.7 Hz, 2H, CH_2_NHCbz), 2.78 (t, *J* = 7.3 Hz, 2H, TrizCH_2_), 1.74 (quin, *J* = 7.5 Hz, 2H, TrizCH_2_CH_2_), 1.59–1.51 (m, 2H, CH_2_CH_2_NHCbz); ^13^C NMR (CDCl_3_, 75 MHz) δ (ppm): 162.7 (NHCON), 161.0 (OC_Ar_), 156.6 (C_Ar_), 156.2 (C_Ar_), 153.5 (C_Ar_), 148.8 (C_Ar_), 138.6 (C_Ar_), 136.7 (C_Ar_), 134.7 (CH_Ar_), 133.8 (C_Ar_), 133.3 (C_Ar_), 131.5 (CH_Ar_), 130.3 (CH_Ar_), 128.7 (CH_Ar_), 128.4 (CH_Ar_), 128.3 (CH_Ar_), 128.2 (CH_Ar_), 127.8 (CH_Ar_), 127.2 (C_Ar_), 126.5 (CH_Ar_), 126.1 (CH_Ar_), 123.6 (CH_Ar_), 121.8 (CH_Ar_), 119.2 (CH_Ar_), 117.1 (CH_Ar_), 112.5 (CH_Ar_), 104.5 (CH_Ar_), 67.7 (OCH_2_), 66.8 (NHCOOCH_2_), 46.4 (CH_2_CH_2_NHCON), 43.0 (OCH_2_CH_2_), 40.9 (CH_2_NHCbz), 38.4 (CH_2_CH_2_NHCON), 29.5 (CH_2_CH_2_NHCbz), 26.4 (TrizCH_2_CH_2_), 25.2 (TrizCH_2_); HRMS (ES, positive mode) *m*/*z*: calcd for C_40_H_39_N_7_O_4_S 713.2784; found 713.2779 (−0.72 ppm).

##### Benzyl-(*E*)-(4-(1-(4-(4-(2-([1,1′-biphenyl]-4-yl)vinyl) thiazol-2-yl)-3-(2-(2-oxoimidazolidin-1-yl)ethoxy)phenyl)-1*H*-1,2,3-triazol-4-yl)butyl)carbamate (**11c**)

Following the general Hantzsch synthesis, **9** (200 mg, 0.37 mmol) reacted with α-bromoketone **10c**(30) (112 mg, 0.37 mmol) for 5 h. After the workup, the residue was purified by flash column chromatography (CH_2_Cl_2_/MeOH, 100:3) to give 174 mg (63%) of **11c** as a colorless oil. ^1^H NMR (CDCl_3_, 400 MHz) δ (ppm): 8.57 (d, *J* = 8.5 Hz, 1H, Ar), 7.81 (s, 1H, Ar), 7.61–7.45 (m, 8H, Ar, ThiazCH=CH), 7.40–7.31 (m, 3H, Ar), 7.31–7.16 (m, 7H, Ar), 7.12 (d, *J* = 16.0 Hz, 1H, ThiazCH=CH), 5.02 (s, 2H, NHCOOCH_2_), 4.68 (bs, 1H, NHCON), 4.36 (t, *J* = 5.7 Hz, 2H, OCH_2_), 3.70 (t, *J* = 5.8 Hz, 2H, OCH_2_CH_2_), 3.50 (dd, *J* = 9.0, 6.7 Hz, 2H, CH_2_CH_2_NHCON), 3.26 (t, *J* = 7.9 Hz, 2H, CH_2_CH_2_NHCON), 3.16 (q, *J* = 6.6 Hz, 2H, CH_2_NHCbz), 2.76 (t, *J* = 7.3 Hz, 2H, TrizCH_2_), 1.75 (quin, *J* = 7.5 Hz, 2H, TrizCH_2_CH_2_), 1.55 (quin, *J* = 7.3 Hz, 2H, CH_2_CH_2_NHCbz); ^13^C NMR (CDCl_3_, 75 MHz) δ (ppm): 162.7 (NHCON), 160.9 (OC_Ar_), 156.6 (C_Ar_), 156.2 (C_Ar_), 153.4 (C_Ar_), 148.8 (C_Ar_), 140.7 (C_Ar_), 140.6 (C_Ar_), 138.6 (CH_Ar_), 136.7 (C_Ar_), 136.2 (CH_Ar_), 130.9 (CH_Ar_), 130.3 (CH_Ar_), 128.9 (CH_Ar_), 128.6 (CH_Ar_), 128.3 (CH_Ar_), 127.5 (CH_Ar_), 127.0 (CH_Ar_), 122.4 (C_Ar_), 121.5 (CH_Ar_), 119.1 (CH_Ar_), 117.4 (CH_Ar_), 112.4 (CH_Ar_), 104.4 (CH_Ar_), 67.7 (OCH_2_), 66.8 (NHCOOCH_2_), 46.4 (CH_2_CH_2_NHCON), 42.9 (OCH_2_CH_2_), 40.9 (CH_2_NHCbz), 38.4 (CH_2_CH_2_NHCON), 29.4 (CH_2_CH_2_NHCbz), 26.4 (TrizCH_2_CH_2_), 25.2 (TrizCH_2_); HRMS (ES, positive mode) *m*/*z*: calcd for C_42_H_41_N_7_O_4_S 739.2941; found 739.2972 (4.21 ppm).

#### General Procedure for N-Cbz Deprotection

A solution of the corresponding Cbz-protected compound (1 equiv) in a 1:1 mixture of THF/MeOH (20 mL) containing Pd/C (10%) (20% w/w) and TFA (0.5–1.5 mL) was hydrogenated at room temperature for 2 h under atmospheric pressure using a balloon filled with hydrogen gas (three cycles of vacuum + hydrogen). The Pd/C was filtered through Whatman poly(tetrafluoroethylene) (PTFE) filter paper, and the solvent was removed under reduced pressure and co-evaporated with mixtures of CH_2_Cl_2_/MeOH (5 × 10 mL). The residue was purified by HPFC on an SP1 Isolera Biotage using reverse-phase columns (from 0% of CH_3_CN to 100% of CH_3_CN in 45 min) to afford to give the final deprotected compounds as trifluoroacetate salts.

#### 1-(2-(5-(4-(4-Ammoniumbutyl)-1*H*-1,2,3-triazol-1-yl)-2-(4-phenethylthiazol-2-yl)phenoxy)ethyl)imidazolidin-2-one 2,2,2-Trifluoroacetate (**12a**)

According to the general hydrogenolysis procedure, a solution of **11a** (55 mg, 0.08 mmol), in 1:1 THF/MeOH (20 mL) containing Pd/C (10%) (20% w/w) (18 mg) and TFA (0.5 mL), was hydrogenated. The residue was purified by HPFC on the SP1 Isolera Biotage using reverse-phase columns (from 0% of CH_3_CN to 100% of CH_3_CN in 45 min) to afford **12a (**19 mg, 35%) as a colorless oil. ^1^H NMR (CD_3_OD, 400 MHz) δ (ppm): 8.47 (s, 1H, Ar), 8.46 (d, *J* = 8.5 Hz, 1H, Ar), 7.70 (d, *J* = 2.0 Hz, 1H, Ar), 7.58 (dd, *J* = 8.6, 2.0 Hz, 1H, Ar), 7.33–7.17 (m, 5H, Ar), 7.15 (s, 1H, Ar), 4.48 (t, *J* = 5.5 Hz, 2H, OCH_2_), 3.74 (t, *J* = 5.5 Hz, 2H, OCH_2_CH_2_), 3.60 (dd, *J* = 9.3, 6.9 Hz, 2H, CH_2_CH_2_NHCON), 3.36 (dd, *J* = 9.3, 6.9 Hz, 2H, CH_2_CH_2_NHCON), 3.15–3.03 (m, 4H, CH_2_CH_2_Ph), 3.00 (t, *J* = 7.4 Hz, 2H, CH_2_NH_3_^+^), 2.87 (t, *J* = 7.2 Hz, 2H, TrizCH_2_), 1.89–1.81 (m, 2H, TrizCH_2_CH_2_), 1.81–1.70 (m, 2H, CH_2_CH_2_NH_3_^+^); ^13^C NMR (CD_3_OD, 100 MHz) δ (ppm): 165.1 (NHCON), 162.0 (OC_Ar_), 157.4 (C_Ar_), 157.1 (C_Ar_), 149.3 (C_Ar_), 142.8 (C_Ar_), 139.7 (C_Ar_), 130.8 (CH_Ar_), 129.5 (CH_Ar_), 129.4 (CH_Ar_), 127.0 (CH_Ar_), 123.8 (C_Ar_), 121.6 (CH_Ar_), 116.9 (CH_Ar_), 113.6 (CH_Ar_), 105.7 (CH_Ar_), 68.4 (OCH_2_), 47.0 (CH_2_CH_2_NHCON), 43.9 (OCH_2_CH_2_), 40.4 (CH_2_NH_3_^+^), 39.3 (CH_2_CH_2_NHCON), 36.7 (CH_2_CH_2_Ph), 34.3 (CH_2_CH_2_Ph), 28.0 (CH_2_CH_2_NH_3_^+^), 27.1 (TrizCH_2_CH_2_), 25.6 (TrizCH_2_); HPLC (gradient A, Agilent): *R*_t_ = 7.1 min; HRMS (ES, positive mode) *m*/*z*: calcd for C_28_H_33_N_7_O_2_S 531.2416; found 531.2424 (1.35 ppm); anal. calcd for C_28_H_33_N_7_O_2_S·TFA: C, 55.80; H, 5.31; N, 15.18; S, 4.97; found: C, 55.94; H, 5.50; N, 15.08; S, 4.51.

#### 1-(2-(5-(4-(4-Ammoniumbutyl)-1*H*-1,2,3-triazol-1-yl)-2-(4-(2-(naphthalen-2-yl)ethyl)thiazol-2-yl)phenoxy)ethyl)imidazolidin-2-one 2,2,2-Trifluoroacetate (**12b**)

Following the deprotection procedure, compound **11b** (218 mg, 0.31 mmol), Pd/C (10%) (20% w/w) (44 mg), and TFA (1.3 mL) in a 1:1 mixture of THF/MeOH (30 mL) were hydrogenated. After the workup, the residue was purified by reverse phase on the Biotage to yield 51 mg (24%) of **12b** as a colorless oil. ^1^H NMR (DMSO-*d*_6_, 400 MHz) δ (ppm): 8.77 (s, 1H, Ar), 8.49 (d, *J* = 8.6 Hz, 1H, Ar), 7.89–7.66 (m, 8H, Ar, NH_3_^+^), 7.52–7.34 (m, 3H, Ar), 7.34 (s, 1H, Ar), 4.47 (t, *J* = 5.7 Hz, 2H, OCH_2_), 3.63 (t, *J* = 5.6 Hz, 2H, OCH_2_CH_2_), 3.48 (dd, *J* = 8.9 Hz, *J* = 6.7 Hz, 2H, CH_2_CH_2_NHCON), 3.33–3.04 (m, 6H, CH_2_CH_2_NHCON, CH_2_NH_3_^+^, ThiazCH_2_CH_2_), 2.93–2.86 (m, 2H, ThiazCH_2_CH_2_), 2.77 (t, *J* = 7.2 Hz, 2H, TrizCH_2_), 1.84–1.71 (m, 2H, TrizCH_2_CH_2_), 1.67–1.56 (m, 2H, CH_2_CH_2_NH_3_^+^); ^13^C NMR (DMSO-*d*_6_, 75 MHz) δ (ppm): 162.1 (NHCON), 159.4 (OC_Ar_), 155.6 (C_Ar_), 155.3 (C_Ar_), 147.7 (C_Ar_), 139.0 (C_Ar_), 137.9 (C_Ar_), 133.2 (CH_Ar_), 131.6 (C_Ar_), 129.1 (CH_Ar_), 127.7 (CH_Ar_), 127.4 (CH_Ar_), 127.3 (C_Ar_), 126.2 (CH_Ar_), 125.9 (CH_Ar_), 125.2 (CH_Ar_), 121.4 (CH_Ar_), 120.4 (CH_Ar_), 116.1 (CH_Ar_), 112.0 (CH_Ar_), 104.3 (CH_Ar_), 67.6 (OCH_2_), 45.3 (CH_2_CH_2_NHCON), 42.4 (OCH_2_CH_2_), 37.5 (CH_2_CH_2_NHCON), 34.9 (ThiazCH_2_CH_2_), 32.5 (ThiazCH_2_CH_2_), 26.5 (CH_2_CH_2_NH_3_^+^), 25.7 (TrizCH_2_CH_2_), 24.8 (TrizCH_2_); HPLC (gradient A, Agilent): *R*_t_ = 7.8 min; HRMS (ES, positive mode) *m*/*z*: calcd for C_32_H_35_N_7_O_2_S 581.2573; found 581.2572 (−0.13 ppm); anal. calcd for C_32_H_35_N_7_O_2_S·TFA: C, 58.69; H, 5.22; N, 14.09; S, 4.61; found: C, 58.20; H, 5.01; N, 14.09; S, 4.12.

#### 1-(2-(2-(4-(2-([1,1′-Biphenyl]-4-yl)ethyl)thiazol-2-yl)-5-(4-(4-ammoniumbutyl)-1*H*-1,2,3-triazol-1-yl)phenoxy)ethyl)imidazolidin-2-one 2,2,2-Trifluoroacetate (**12c**)

Following the hydrogenolysis procedure, compound **11c** (50 mg, 0.07 mmol), Pd/C (10%) (20% w/w) (10 mg), and TFA (0.5 mL) in a 1:1 mixture of THF/MeOH (20 mL) were hydrogenated. After the workup, the residue was purified by reverse phase on the Biotage to give **12c (**17 mg, 35%) as a colorless oil. ^1^H NMR (DMSO-*d*_6_, 500 MHz) δ (ppm): 8.77 (s, 1H, Ar), 8.47 (d, *J* = 8.6 Hz, 1H, Ar), 7.75 (d, *J* = 2.1 Hz, 1H, Ar), 7.69 (dd, *J* = 8.6, 2.1 Hz, 1H, Ar), 7.66–7.62 (m, 2H, Ar), 7.60–7.56 (m, 2H, Ar), 7.48–7.39 (m, 3H, Ar), 7.38–7.29 (m, 3H, Ar), 6.41 (bs, 1H, NHCON), 4.47 (t, *J* = 5.7 Hz, 2H, OCH_2_), 3.63 (t, *J* = 5.7 Hz, 2H, OCH_2_CH_2_), 3.49 (dd, *J* = 9.0, 6.7 Hz, 2H, CH_2_CH_2_NHCON), 3.21 (dd, *J* = 9.3, 6.5 Hz, 2H, CH_2_CH_2_NHCON), 3.17–2.96 (m, 4H, CH_2_NH_3_^+^, ThiazCH_2_CH_2_), 2.74 (t, *J* = 7.4 Hz, 2H, TrizCH_2_), 2.69 (t, *J* = 7.2 Hz, 2H, ThiazCH_2_CH_2_), 1.72 (quin, *J* = 7.6 Hz, 2H, TrizCH_2_CH_2_), 1.58–1.42 (m, 2H, CH_2_CH_2_NH_3_^+^); ^13^C NMR (DMSO-*d*_6_, 100 MHz) δ (ppm): 162.1 (NHCON), 159.4 (OC_Ar_), 155.6 (C_Ar_), 155.3 (C_Ar_), 148.1 (C_Ar_), 140.7 (C_Ar_), 140.0 (C_Ar_), 138.0 (C_Ar_), 137.8 (C_Ar_), 129.0 (CH_Ar_), 128.9 (CH_Ar_), 128.9 (CH_Ar_), 127.2 (CH_Ar_), 126.5 (CH_Ar_), 126.5 (CH_Ar_), 121.3 (C_Ar_), 120.3 (CH_Ar_), 116.0 (CH_Ar_), 112.0 (CH_Ar_), 104.3 (CH_Ar_), 67.6 (OCH_2_), 45.4 (CH_2_CH_2_NHCON), 42.4 (OCH_2_CH_2_), 37.7 (CH_2_NH_3_^+^), 37.5 (CH_2_CH_2_NHCON), 34.3 (ThiazCH_2_CH_2_), 32.5 (ThiazCH_2_CH_2_), 30.1 (CH_2_CH_2_NH_3_^+^), 25.9 (TrizCH_2_CH_2_), 24.7 (TrizCH_2_); HPLC (gradient A, Agilent): *R*_t_ = 8.4 min; HRMS (ES, positive mode) *m*/*z*: calcd for C_34_H_37_N_7_O_2_S 607.2729; found 607.2740 (1.73 ppm); anal. calcd for C_34_H_37_N_7_O_2_S·TFA: C, 59.91; H, 5.31; N, 13.58; S, 4.44; found: C, 60.08; H, 5.09; N, 13.18; S, 4.20.

#### Phosphonium (2-Oxo-3-phenylpropyl)triphenyl Chloride (**15**)

A solution of 1-chloro-3-phenylpropan-2-one (250 mg, 1.48 mmol) and triphenylphosphine (583 mg, 2.22 mmol) in anhydrous toluene (8 mL) and under an argon atmosphere was heated to 110 °C for 6 h in a pressure tube. After cooling to room temperature, the white solid was filtered and washed with cooled diethyl ether (3 × 10 mL) to give **15** (498 mg, 78%) as a white solid;^50^ m.p.: 200–202 °C; ^1^H NMR (DMSO-*d*_6_, 400 MHz) δ (ppm): 7.93–7.64 (m, 15H, Ar), 7.31–7.20 (m, 3H, Ar), 7.12 (d, *J* = 6.7 Hz, 2H, Ar), 5.91 (d, *J* = 12.7 Hz, 2H, Ar), 4.14 (s, 2H, CH_2_CO).

#### (*E*)-4-([1,1′-Biphenyl]-4-yl)-1-phenylbut-3-en-2-one (**16c**)

A stirred solution of **15** (250 mg, 0.58 mmol) and KOH (130 mg, 2.37 mmol) in dry toluene (15 mL) was heated at 110 °C in a pressure flask for 3 h. After cooling to room temperature, [1,1′-biphenyl]-4-carboxaldehyde (158 mg, 0.87 mmol) was added dropwise. The reaction mixture was stirred at 110 °C for 3 additional hours, cooled, and evaporated under reduced pressure. The residue was dissolved in EtOAc (30 mL), washed with H_2_O (2 × 30 mL) and brine (1 × 30 mL), dried (Na_2_SO_4_), filtered, and evaporated to dryness. The residue was purified by flash column chromatography (hexane/EtOAc, 95:5) to yield **16c** (107 mg, 62%) as a white solid; m.p.: 121–126 °C; ^1^H NMR (CDCl_3_, 400 MHz) δ (ppm): 7.59 (d, *J* = 16.1 Hz, 1H, CH=CHCO), 7.56–7.48 (m, 6H, Ar), 7.37 (t, *J* = 7.4 Hz, 2H, Ar), 7.34–7.25 (m, 3H, Ar), 7.24–7.15 (m, 3H, Ar), 6.74 (d, *J* = 16.0 Hz, 1H, CH=CHCO), 3.88 (s, 2H, CH_2_CO); ^13^C NMR (CDCl_3_, 75 MHz) δ (ppm): 197.4 (CO), 143.5 (ArCH=CH), 143.1 (C_Ar_), 140.2 (CH_Ar_), 134.6 (C_Ar_), 133.5 (C_Ar_), 129.6 (CH_Ar_), 129.0 (CH_Ar_), 129.0 (CH_Ar_), 128.9 (CH_Ar_), 128.1 (C_Ar_), 127.7 (ArCH=CH), 127.2 (CH_Ar_), 127.1 (CH_Ar_), 125.1 (CH_Ar_), 48.6 (CH_2_CO); MS (ESI, positive mode) *m*/*z*: 321.0 [M + Na]^+^, 299.3 [M + H]^+^.

#### (*E*)-4-(4-Phenyloxyphenyl)-1-phenylbut-3-en-2-one (**16d**)

Following a procedure similar to that described for **16c**, a solution of phosphonium salt **15** (700 mg, 1.62 mmol), KOH (728 mg, 13 mmol), and 4-phenoxybenzaldehyde (0.34 mL, 1.95 mmol) in dry toluene (20 mL) was reacted in a pressure flask. After the workup, the residue was purified by flash column chromatography (hexane/EtOAc, 95:5) to give 241 mg (47%) of **16d** as a white solid; m.p.: 95–97 °C; ^1^H NMR (CDCl_3_, 400 MHz) δ (ppm): 7.51 (d, *J* = 16.0 Hz, 1H, CH=CHCO), 7.47–7.33 (m, 2H, Ar), 7.30–7.21 (m, 4H, Ar), 7.19–7.13 (m, 3H, Ar), 7.06 (tt, *J* = 7.4, 1.1 Hz, 1H, Ar), 6.97–6.90 (m, 2H, Ar), 6.89–6.83 (m, 2H, Ar), 6.60 (d, *J* = 16.0 Hz, 1H, CH=CHCO), 3.83 (s, 2H, CH_2_CO); ^13^C NMR (CDCl_3_, 75 MHz) δ (ppm): 197.3 (CO), 159.9 (OC_Ar_), 156.1 (OC_Ar_), 142.8 (ArCH=CH), 134.7 (C_Ar_), 130.2 (CH_Ar_), 130.0 (CH_Ar_), 129.6 (CH_Ar_), 129.2 (C_Ar_), 128.9 (CH_Ar_), 127.1 (ArCH=CH), 124.3 (CH_Ar_), 124.0 (CH_Ar_), 119.8 (CH_Ar_), 118.5 (CH_Ar_), 48.5 (CH_2_CO); MS (ESI, positive mode) *m*/*z*: 337.0 [M + Na]^+^, 315.0 [M + H]^+^.

#### General Procedure for the Synthesis of α-Bromomethylketones **17a**–**d**

To a solution of the corresponding ketone (1 equiv) in CH_3_CN, *p-*TsOH (1.3–2.6 equiv) and NBS (1.3–2.6 equiv) were successively added at room temperature and the mixture was stirred for 6 h/overnight. After quenching with H_2_O (30 mL), the reaction mixture was carefully concentrated under reduced pressure without heating. The aqueous crude was extracted with EtOAc (3 × 30 mL), and the organic layers were dried (Na_2_SO_4_), filtered, and evaporated to dryness. The residue was purified by flash column chromatography or CCTLC on the Chromatotron (the eluents are specified in each case) to give the desired α-bromoketones **17a**–**d**.

##### 2-([1,1′-Biphenyl]-4-yl)-1-bromo-1-phenylethan-2-one (**17a**)

Following the general bromination procedure, *p-*TsOH (173 mg, 0.92 mmol) and NBS (165 mg, 0.92 mmol) were successively added to a solution of the commercially available **16a** (50 mg, 0.18 mmol) in CH_3_CN (15 mL). The reaction was stirred at room temperature overnight. After the workup, the residue was purified by CCTLC on the Chromatotron (hexane/EtOAc, 80:20) to give 62 mg (98%) of **17a** as a white solid; m.p.: decompose without melting; ^1^H NMR (CDCl_3_, 400 MHz) δ (ppm): 8.08 (d, *J* = 8.2 Hz, 2H, Ar), 7.67 (d, *J* = 8.4 Hz, 2H, Ar), 7.63–7.54 (m, 4H, Ar), 7.47 (t, *J* = 7.5 Hz, 2H, Ar), 7.43–7.29 (m, 4H, Ar), 6.43 (s, 1H, BrCHCO).

##### 2-(4-Phenyloxyphenyl)-1-bromo-1-phenylethan-2-one (**17b**)

The general bromination procedure was followed with the commercially available **16b** (150 mg, 0.52 mmol), *p-*TsOH (297 mg, 1.56 mmol), and NBS (278 mg, 1.56 mmol) in CH_3_CN (22 mL) at room temperature overnight. The residue was purified by CCTLC on the Chromatotron (hexane/CH_2_Cl_2_, 70:30) to afford **17b** (140 mg, 73%) as a white solid; m.p.: 116-118 °C; ^1^H NMR (CDCl_3_, 400 MHz) δ (ppm): 7.97 (d, *J* = 8.9 Hz, 2H, Ar), 7.53 (dd, *J* = 8.1, 1.6 Hz, 2H, Ar), 7.44–7.29 (m, 5H, Ar), 7.22 (tt, *J* = 7.3, 1.0 Hz, 1H, Ar), 7.06 (dd, *J* = 7.9, 1.6 Hz, 2H, Ar), 6.96 (d, *J* = 8.9 Hz, 2H, Ar), 6.34 (s, 1H, BrCHCO); ^13^C NMR (CDCl_3_, 75 MHz) δ (ppm): 189.7 (CO), 162.7 (OC_Ar_), 155.1 (OC_Ar_), 136.2 (C_Ar_), 131.7 (CH_Ar_), 130.3 (CH_Ar_), 129.2 (CH_Ar_), 129.2 (CH_Ar_), 128.5 (C_Ar_), 125.1 (CH_Ar_), 120.6 (CH_Ar_), 117.4 (CH_Ar_), 51.2 (BrCHCO); MS (ESI, positive mode) *m*/*z*: 389 [M + Na]^+^ with a Br isotopic pattern.

##### (*E*)-4-([1,1′-Biphenyl]-4-yl)-1-bromo-1-phenylbut-3-en-2-one (**17c**)

Following the general procedure, compound **16c** (200 mg, 0.67 mmol) dissolved in CH_3_CN (25 mL) was sequentially treated with *p-*TsOH (191 mg, 1.00 mmol) and NBS (179 mg, 1.00 mmol) at room temperature for 4 h. After the workup, the residue was purified by flash column chromatography (hexane/EtOAc, 90:10) to give **17c** (253 mg, 79%) as a white solid; m.p.: 118–120 °C; ^1^H NMR (CDCl_3_, 400 MHz) δ (ppm): 7.71 (d, *J* = 15.8 Hz, 1H, CH=CHCO), 7.56–7.49 (m, 6H, Ar), 7.47–7.42 (m, 2H, Ar), 7.41–7.34 (m, 2H, Ar), 7.35–7.24 (m, 4H, Ar), 6.94 (d, *J* = 15.8 Hz, 1H, CH=CHCO), 5.61 (s, 1H, BrCHCO); ^13^C NMR (CDCl_3_, 75 MHz) δ (ppm): 190.4 (CO), 145.0 (ArCH=CH), 143.9 (C_Ar_), 140.1 (CH_Ar_), 135.4 (C_Ar_), 133.2 (C_Ar_), 129.3 (CH_Ar_), 129.3 (CH_Ar_), 129.2 (CH_Ar_), 129.1 (CH_Ar_), 128.1 (C_Ar_), 127.7 (ArCH=CH), 127.2 (CH_Ar_), 127.1 (CH_Ar_), 121.4 (CH_Ar_), 55.5 (BrCHCO).

##### (*E*)-4-(4-Phenyloxyphenyl)-1-phenylbut-3-en-2-one (**17d**)

According to the general bromination procedure, the α,β-unsaturated compound **16d** (211 mg, 0.67 mmol), *p-*TsOH (140 mg, 0.74 mmol), and NBS (131 mg, 0.74 mmol) dissolved in CH_3_CN (25 mL) were reacted at room temperature for 4 h. After the workup, the crude was purified by flash column chromatography (hexane/EtOAc, 97:3) to give **17d** (150 mg, 39%) as a yellow oil. ^1^H NMR (CDCl_3_, 400 MHz) δ (ppm): 7.72 (d, *J* = 15.7 Hz, 1H, CH=CHCO), 7.56–7.47 (m, 4H, Ar), 7.43–7.32 (m, 5H, Ar), 7.18 (tt, *J* = 7.4, 1.1 Hz, 1H, Ar), 7.10–7.01 (m, 2H, Ar), 6.97 (d, *J* = 8.8 Hz, 2H, Ar), 6.89 (d, *J* = 15.7 Hz, 1H, CH=CHCO), 5.66 (s, 1H, BrCHCO); ^13^C NMR (CDCl_3_, 75 MHz) δ (ppm): 190.4 (CO), 160.4 (OC_Ar_), 156.0 (OC_Ar_), 144.8 (ArCH=CH), 135.5 (C_Ar_), 130.6 (CH_Ar_), 130.1 (CH_Ar_), 129.2 (CH_Ar_), 129.1 (C_Ar_), 129.1 (CH_Ar_), 128.9 (ArCH=CH), 124.5 (CH_Ar_), 120.2 (CH_Ar_), 120.0 (CH_Ar_), 118.5 (CH_Ar_), 55.5 (BrCHCO); MS (ESI, positive mode) *m*/*z*: 417.0 [M + Na]^+^, 393.0 [M + H]^+^ with a Br isotopic pattern.

#### Benzyl-(4-(1-(4-(4,5-diphenylthiazol-2-yl)-3-(2-(2-oxoimidazolidin-1-yl)ethoxy)phenyl)-1*H*-1,2,3-triazol-4-yl)butyl)carbamate (**18a**)

Following the general procedure of Hantzsch synthesis, the thioamide **9** (150 mg, 0.28 mmol) and the commercially available 1-bromo-1,2-diphenylethan-2-one **17e** (77 mg, 0.28 mmol) reacted in *^i^*PrOH (20 mL). After the workup, the final residue was purified by CCTLC in the Chromatotron (CH_2_Cl_2_/MeOH, 97:3) to provide 191 mg (94%) of **18a** as a colorless oil. ^1^H NMR (CDCl_3_, 400 MHz) δ (ppm): 8.62 (d, *J* = 8.6 Hz, 1H, Ar), 7.89 (s, 1H, Ar), 7.64–7.57 (m, 3H, Ar), 7.44–7.23 (m, 14H, Ar), 5.19 (t, *J* = 6.0 Hz, 1H, NHCbz), 5.08 (s, 2H, NHCOOCH_2_), 4.87 (bs, 1H, NHCON), 4.44 (t, *J* = 5.9 Hz, 2H, OCH_2_), 3.75 (t, *J* = 5.8 Hz, 2H, OCH_2_CH_2_), 3.57 (dd, *J* = 9.0, 6.7 Hz, 2H, CH_2_CH_2_NHCON), 3.29 (t, *J* = 8.0 Hz, 2H, CH_2_CH_2_NHCON), 3.25 (q, *J* = 6.9 Hz, 2H, CH_2_NHCbz), 2.82 (t, *J* = 7.4 Hz, 2H, TrizCH_2_), 1.84–1.71 (m, 2H, TrizCH_2_CH_2_), 1.62 (quin, *J* = 6.2 Hz, 2H, CH_2_CH_2_NHCbz); ^13^C NMR (CDCl_3_, 75 MHz) δ (ppm): 162.2 (NHCON), 158.3 (OC_Ar_), 156.6 (C_Ar_), 155.9 (C_Ar_), 149.3 (C_Ar_), 148.7 (C_Ar_), 138.4 (C_Ar_), 136.7 (C_Ar_), 135.1 (C_Ar_), 134.3 (C_Ar_), 132.2 (CH_Ar_), 129.7 (CH_Ar_), 129.6 (CH_Ar_), 129.2 (CH_Ar_), 129.1 (CH_Ar_), 128.8 (CH_Ar_), 128.6 (CH_Ar_), 128.4 (CH_Ar_), 128.2 (CH_Ar_), 127.9 (C_Ar_), 122.5 (CH_Ar_), 119.1 (CH_Ar_), 112.4 (CH_Ar_), 104.4 (CH_Ar_), 67.7 (OCH_2_), 66.7 (NHCOOCH_2_), 46.4 (CH_2_CH_2_NHCON), 42.9 (OCH_2_CH_2_), 40.8 (CH_2_NHCbz), 38.4 (CH_2_CH_2_NHCON), 29.4 (CH_2_CH_2_NHCbz), 26.3 (TrizCH_2_CH_2_), 25.2 (TrizCH_2_); HRMS (ES, positive mode) *m*/*z*: calcd for C_40_H_39_N_7_O_4_S 713.2784; found 713.2786 (0.23 ppm).

#### Benzyl-(4-(1-(3-(2-(2-oxoimidazolidin-1-yl)ethoxy)-4-(4-([1,1′-biphenyl]-4-yl)-5-phenylthiazol-2-yl)phenyl)-1*H*-1,2,3-triazol-4-yl)butyl)carbamate (**18b**)

Following the general procedure for the synthesis of thiazoles by Hantzsch cyclization, thioamide **9** (194 mg, 0.36 mmol) reacted with α-bromoketone **17a** (127 mg, 0.36 mmol) in *^i^*PrOH (20 mL). After the workup, the residue was purified by CCTLC on the Chromatotron (CH_2_Cl_2_/MeOH, 95:5) to give **18b** (244 mg, 84%) as a colorless oil. ^1^H NMR (CDCl_3_, 400 MHz) δ (ppm): 8.66 (d, *J* = 8.6 Hz, 1H, Ar), 7.60 (s, 1H, Ar), 7.71 (d, *J* = 8.1 Hz, 2H, Ar), 7.65–7.51 (m, 3H, Ar), 7.56 (d, *J* = 8.1 Hz, 2H, Ar), 7.52–7.18 (m, 14H, Ar), 5.13 (bs, 1H, NHCON), 5.10 (s, 2H, NCOOCH_2_), 4.67 (bs, 1H, NHCbz), 4.47 (t, *J* = 5.9 Hz, 2H, OCH_2_), 3.78 (t, *J* = 5.9 Hz, 2H, OCH_2_CH_2_), 3.59 (dd, *J* = 9.0, 6.7 Hz, 2H, CH_2_CH_2_NHCON), 3.37–3.22 (m, 4H, CH_2_CH_2_NHCON, CH_2_NHCbz), 2.84 (t, *J* = 7.3 Hz, 2H, TrizCH_2_), 1.86–1.74 (m, 2H, TrizCH_2_CH_2_), 1.64 (quin, *J* = 7.2 Hz, 2H, CH_2_CH_2_NHCbz); ^13^C NMR (CDCl_3_, 75 MHz) δ (ppm): 162.7 (NHCON), 158.4 (OC_Ar_), 156.6 (C_Ar_), 156.0 (NHCOO), 149.0 (C_Ar_), 148.8 (C_Ar_), 140.8 (C_Ar_), 140.5 (C_Ar_), 138.5 (C_Ar_), 136.7 (C_Ar_), 134.4 (C_Ar_), 134.1 (CH_Ar_), 132.3 (C_Ar_), 129.8 (CH_Ar_), 129.5 (CH_Ar_), 128.9 (CH_Ar_), 128.9 (CH_Ar_), 128.6 (CH_Ar_), 128.3 (CH_Ar_), 128.2 (CH_Ar_), 127.5 (C_Ar_), 127.1 (CH_Ar_), 122.5 (CH_Ar_), 119.2 (C_Ar_), 112.5 (CH_Ar_), 104.5 (CH_Ar_), 67.7 (NHCOOCH_2_), 66.8 (OCH_2_), 46.4 (CH_2_CH_2_NHCON), 42.9 (OCH_2_CH_2_), 40.9 (CH_2_NHCbz), 38.4 (CH_2_CH_2_NHCON), 29.4 (CH_2_CH_2_NHCbz), 26.4 (TrizCH_2_CH_2_), 25.2 (TrizCH_2_); HRMS (ES, positive mode) *m*/*z*: calcd for C_46_H_43_N_7_O_4_S 789.3097; found 789.3063 (−4.31 ppm).

#### Benzyl-(4-(1-(3-(2-(2-oxoimidazolidin-1-yl)ethoxy)-4-(4-(4-phenyloxyphenyl-1-yl)-5-phenylthiazol-2-yl)phenyl)-1*H*-1,2,3-triazol-4-yl)butyl)carbamate (**18c**)

Following the general procedure for the synthesis of thiazoles by Hantzsch cyclization, thioamide **9** (190 mg, 0.35 mmol) and α-bromoketone **17b** (130 mg, 0.35 mmol) reacted in *^i^*PrOH (20 mL). The final residue was purified by CCTLC in the Chromatotron (CH_2_Cl_2_/MeOH, 95:5) to yield 235 mg (81%) of **18c** a colorless oil. ^1^H NMR (CDCl_3_, 400 MHz) δ (ppm): 8.63 (d, *J* = 8.5 Hz, 1H, Ar), 7.90 (s, 1H, Ar), 7.65–7.55 (m, 3H, Ar), 7.47–7.24 (m, 13H, Ar), 7.11 (t, *J* = 7.4 Hz, 1H, Ar), 7.04 (d, *J* = 8.0 Hz, 2H, Ar), 6.95 (d, *J* = 8.3 Hz, 2H, Ar), 5.14 (bs, 1H, NHCON), 5.09 (s, 2H, NCOOCH_2_), 4.73 (bs, 1H, NHCbz), 4.46 (t, *J* = 5.9 Hz, 2H, OCH_2_), 3.77 (t, *J* = 5.9 Hz, 2H, OCH_2_CH_2_), 3.59 (dd, *J* = 9.0, 6.7 Hz, 2H, CH_2_CH_2_NHCON), 3.37–3.21 (m, 4H, CH_2_CH_2_NHCON, CH_2_NHCbz), 2.84 (t, *J* = 7.3 Hz, 2H, TrizCH_2_), 1.84–1.74 (m, 2H, TrizCH_2_CH_2_), 1.63 (quin, *J* = 7.2 Hz, 2H, CH_2_CH_2_NHCbz); ^13^C NMR (CDCl_3_, 75 MHz) δ (ppm): 162.7 (NHCON), 158.3 (OC_Ar_), 157.2 (C_Ar_), 157.0 (OC_Ar_), 156.6 (OC_Ar_), 156.0 (NHCOO), 148.8 (C_Ar_), 138.5 (C_Ar_), 136.7 (C_Ar_), 133.7 (C_Ar_), 132.3 (C_Ar_), 130.7 (CH_Ar_), 130.1 (C_Ar_), 129.9 (CH_Ar_), 129.7 (CH_Ar_), 129.7 (CH_Ar_), 128.9 (CH_Ar_), 128.6 (CH_Ar_), 128.2 (C_Ar_), 128.2 (CH_Ar_), 123.6 (CH_Ar_), 122.5 (C_Ar_), 119.3 (CH_Ar_), 119.1 (C_Ar_), 118.6 (CH_Ar_), 112.5 (CH_Ar_), 104.5 (CH_Ar_), 67.7 (NHCOOCH_2_), 66.8 (OCH_2_), 46.4 (CH_2_CH_2_NHCON), 42.9 (OCH_2_CH_2_), 40.9 (CH_2_NHCbz), 38.4 (CH_2_CH_2_NHCON), 29.4 (CH_2_CH_2_NHCbz), 26.3 (TrizCH_2_CH_2_), 25.2 (TrizCH_2_); HRMS (ES, positive mode) *m*/*z*: calcd for C_46_H_43_N_7_O_5_S 805.3046; found 805.3054 (0.92 ppm).

#### Benzyl-(*E*)-(4-(1-(4-(4-(2-([1,1′-biphenyl]-4-yl)vinyl)-5-phenylthiazol-2-yl)-3-(2-(2-oxoimidazolidin-1-yl)ethoxy) phenyl)-1*H*-1,2,3-triazol-4-yl)butyl)carbamate (**18d**)

Following the general Hantzsch cyclization procedure, thioamide **9** (200 mg, 0.37 mmol) and α-bromoketone **17c** (140 mg, 0.37 mmol) reacted in *^i^*PrOH (20 mL). After the workup, the residue was purified by CCTLC on the Chromatotron (CH_2_Cl_2_/MeOH, 95:5) to give **18d** (183 mg, 59%) as a yellow oil. ^1^H NMR (DMSO-*d*_6_, 400 MHz) δ (ppm): 8.72 (s, 1H, Ar), 8.61 (d, *J* = 8.4 Hz, 1H, Ar), 7.80–7.71 (m, 3H, Ar, ThiazCH=CH), 7.70–7.53 (m, 10H, Ar), 7.51–7.42 (m, 3H, Ar), 7.39–7.27 (m, 3H, Ar, NHCbz), 7.24 (d, *J* = 15.7 Hz, 1H, ThiazCH=CH), 6.42 (bs, 1H, NHCON), 5.02 (s, 2H, NHCOOCH_2_), 4.48 (t, *J* = 5.7 Hz, 2H, OCH_2_), 3.66 (t, *J* = 5.6 Hz, 2H, OCH_2_CH_2_), 3.49 (dd, *J* = 9.0, 6.6 Hz, 2H, CH_2_CH_2_NHCON), 3.23 (dd, *J* = 9.0, 6.7 Hz, 2H, CH_2_CH_2_NHCON), 3.08 (q, *J* = 6.6 Hz, 2H, CH_2_NHCbz), 2.72 (t, *J* = 7.1 Hz, 2H, TrizCH_2_), 1.70 (quin, *J* = 7.5 Hz, 2H, TrizCH_2_CH_2_), 1.52 (quin, *J* = 7.2 Hz, 2H, CH_2_CH_2_NHCbz); ^13^C NMR (DMSO-*d*_6_, 75 MHz) δ (ppm): 161.8 (NHCON), 158.0 (OC_Ar_), 155.8 (NHCOO), 147.9 (C_Ar_), 146.6 (C_Ar_), 139.4 (C_Ar_), 139.3 (C_Ar_), 138.3 (C_Ar_), 137.1 (C_Ar_), 135.8 (C_Ar_), 135.0 (C_Ar_), 131.6 (C_Ar_), 130.9 (CH_Ar_), 129.9 (CH_Ar_), 128.9 (CH_Ar_), 128.6 (CH_Ar_), 128.0 (C_Ar_), 128.0 (CH_Ar_), 127.4 (CH_Ar_), 127.3 (C_Ar_), 127.2 (CH_Ar_), 126.9 (CH_Ar_), 126.7 (C_Ar_), 126.2 (CH_Ar_), 120.9 (CH_Ar_), 119.9 (CH_Ar_), 119.5 (C_Ar_), 112.0 (CH_Ar_), 104.3 (CH_Ar_), 67.3 (NHCOOCH_2_), 64.9 (OCH_2_), 45.0 (CH_2_CH_2_NHCON), 42.3 (OCH_2_CH_2_), 39.9 (CH_2_NHCbz), 37.3 (CH_2_CH_2_NHCON), 28.7 (CH_2_CH_2_NHCbz), 25.8 (TrizCH_2_CH_2_), 24.5 (TrizCH_2_); HRMS (ES, positive mode) *m*/*z*: calcd for C_48_H_45_N_7_O_4_S 815.3254; found 815.3266 (1.56 ppm).

#### Benzyl-(*E*)-(4-(1-(4-(4-(2-([4-phenyloxyphenyl]-1-yl)vinyl)-5-phenylthiazol-2-yl)-3-(2-(2-oxoimidazolidin-1-yl)ethoxy)phenyl)-1*H*-1,2,3-triazol-4-yl)butyl)carbamate (**18e**)

According to the general Hantzsch procedure, thioamide **9** (183 mg, 0.34 mmol) was reacted with α-bromoketone **17d** (134 mg, 0.34 mmol) in *^i^*PrOH (23 mL). After purification of the residue by CCTLC on the Chromatotron (CH_2_Cl_2_/MeOH, 96:4), **18e** (150 mg, 52%) was obtained as a colorless oil. ^1^H NMR (DMSO-*d*_6_, 400 MHz) δ (ppm): 8.74 (s, 1H, Ar), 8.61 (d, *J* = 8.5 Hz, 1H, Ar), 7.85–7.67 (m, 3H, Ar, ThiazCH=CH), 7.64–7.51 (m, 5H, Ar), 7.51–7.24 (m, 9H, Ar), 7.20–7.10 (m, 2H, Ar, ThiazCH=CH), 7.05 (d, *J* = 7.7 Hz, 2H, Ar), 7.00 (d, *J* = 8.6 Hz, 2H, Ar), 6.40 (bs, 1H, NHCON), 5.01 (s, 2H, NHCOOCH_2_), 4.49 (t, *J* = 5.7 Hz, 2H, OCH_2_), 3.66 (t, *J* = 5.6 Hz, 2H, OCH_2_CH_2_), 3.49 (dd, *J* = 9.0, 6.6 Hz, 2H, CH_2_CH_2_NHCON), 3.22 (t, *J* = 7.9 Hz, 2H, CH_2_CH_2_NHCON), 3.07 (q, *J* = 6.6 Hz, 2H, CH_2_NHCbz), 2.74 (t, *J* = 7.5 Hz, 2H, TrizCH_2_), 1.69 (quin, *J* = 7.5 Hz, 2H, TrizCH_2_CH_2_), 1.53 (quin, *J* = 7.2 Hz, 2H, CH_2_CH_2_NHCbz); ^13^C NMR (DMSO-*d*_6_, 75 MHz) δ (ppm): 162.1 (NHCON), 158.1 (C_Ar_), 156.6 (OC_Ar_), 156.3 (OC_Ar_), 156.1 (OC_Ar_), 156.0 (NHCOO), 148.1 (C_Ar_), 146.7 (OC_Ar_), 138.4 (C_Ar_), 137.3 (C_Ar_), 134.8 (C_Ar_), 132.0 (C_Ar_), 131.6 (C_Ar_), 131.1 (C_Ar_), 130.1 (C_Ar_), 129.4 (CH_Ar_), 129.2 (CH_Ar_), 128.4 (CH_Ar_), 128.3 (CH_Ar_), 127.7 (CH_Ar_), 123.7 (CH_Ar_), 120.9 (CH_Ar_), 120.3 (C_Ar_), 118.9 (CH_Ar_), 118.7 (CH_Ar_), 112.0 (CH_Ar_), 104.3 (CH_Ar_), 67.4 (NHCOOCH_2_), 65.1 (OCH_2_), 45.1 (CH_2_CH_2_NHCON), 42.4 (OCH_2_CH_2_), 40.0 (CH_2_NHCbz), 37.5 (CH_2_CH_2_NHCON), 28.9 (CH_2_CH_2_NHCbz), 26.0 (TrizCH_2_CH_2_), 24.7 (TrizCH_2_); HRMS (ES, positive mode) *m*/*z*: calcd for C_48_H_45_N_7_O_5_S 831.3203; found 831.3174 (−3.45 ppm).

#### 1-(2-(5-(4-(4-Ammoniobutyl)-1*H*-1,2,3-triazol-1-yl)-2-(4,5-diphenylthiazol-2-yl)phenoxy)ethyl)imidazolidin-2-one 2,2,2-Trifluoroacetate (**19a**)

Following the general Cbz removal procedure, **18a** (100 mg, 0.14 mmol), Pd/C 10% (20 mg) and TFA (1 mL) afforded, after purification, compound **19a** (25 mg, 26%) as a colorless oil. ^1^H NMR (CD_3_OD, 400 MHz) δ (ppm): 8.56 (d, *J* = 8.6 Hz, 1H, Ar), 8.46 (s, 1H, Ar), 7.71 (d, *J* = 8.0 Hz, 1H, Ar), 7.59 (dd, *J* = 8.6, 2.0 Hz, 1H, Ar), 7.56–7.51 (m, 2H, Ar), 7.44–7.27 (m, 8H, Ar), 4.51 (t, *J* = 5.5 Hz, 2H, OCH_2_), 3.76 (t, *J* = 5.5 Hz, 2H, OCH_2_CH_2_), 3.62 (dd, *J* = 9.3, 6.9 Hz, 2H, CH_2_CH_2_NHCON), 3.32 (dd, *J* = 9.3, 6.9 Hz, 2H, CH_2_CH_2_NHCON), 3.00 (q, *J* = 6.5 Hz, 2H, CH_2_NH_3_^+^), 2.86 (t, *J* = 7.2 Hz, 2H, TrizCH_2_), 1.95–1.65 (m, 4H, TrizCH_2_CH_2_, CH_2_CH_2_NH_3_^+^); ^13^C NMR (CD_3_OD, 75 MHz) δ (ppm): 165.0 (NHCON), 159.9 (OC_Ar_), 157.5 (C_Ar_), 150.6 (C_Ar_), 149.3 (C_Ar_), 139.8 (C_Ar_), 136.4 (C_Ar_), 135.8 (C_Ar_), 133.3 (CH_Ar_), 130.6 (CH_Ar_), 130.5 (CH_Ar_), 130.3 (CH_Ar_), 129.9 (CH_Ar_), 129.3 (CH_Ar_), 129.3 (CH_Ar_), 129.0 (CH_Ar_), 129.0 (CH_Ar_), 123.7 (CH_Ar_), 121.6 (CH_Ar_), 113.6 (CH_Ar_), 105.6 (CH_Ar_), 68.2 (OCH_2_), 46.9 (CH_2_CH_2_NHCON), 43.9 (OCH_2_CH_2_), 40.4 (CH_2_NH_3_^+^), 39.3 (CH_2_CH_2_NHCON), 28.0 (CH_2_CH_2_NH_3_^+^), 27.1 (TrizCH_2_CH_2_), 25.6 (TrizCH_2_); HPLC (gradient A, Agilent): *R*_t_ = 9.3 min; HRMS (ES, positive mode) *m*/*z*: calcd for C_32_H_33_N_7_O_2_S 579.2416; found 579.242 (0.61 ppm); anal. calcd for C_32_H_34_N_7_O_2_S·TFA: C, 58.87; H, 4.94; N, 14.13; S, 4.62; found: C, 59.15; H, 5.36 N, 14.62; S, 5.03.

#### 1-(2-(5-(4-(4-Ammoniobutyl)-1*H*-1,2,3-triazol-1-yl)-2-(4-([1,1′-biphenyl]-4-yl)-5-phenylthiazol-2-yl)phenoxy) ethyl)imidazolidin-2-one 2,2,2-Trifluoroacetate (**19b**)

The general N-deprotection procedure described for **12a**–**c** was followed with **18b** (100 mg, 0.13 mmol), Pd/C 10% (20 mg), and TFA (0.8 mL) to give, after workup and purification, compound **19b** (15 mg, 15%) as a colorless oil. ^1^H NMR (DMSO-*d*_6_, 400 MHz) δ (ppm): 8.79 (s, 1H, Ar), 8.57 (d, *J* = 8.6 Hz, 1H, Ar), 7.80 (d, *J* = 8.1 Hz, 1H, Ar), 7.77–7.60 (m, 10H, Ar, NH_3_^+^), 7.54–7.41 (m, 7H, Ar), 7.37 (t, *J* = 7.5 Hz, 1H, Ar), 6.40 (bs, 1H, NHCON), 4.53 (t, *J* = 5.6 Hz, 2H, OCH_2_), 3.66 (t, *J* = 5.6 Hz, 2H, OCH_2_CH_2_), 3.51 (dd, *J* = 9.0, 6.7 Hz, 2H, CH_2_CH_2_NHCON), 3.23 (t, *J* = 7.8 Hz, 2H, CH_2_CH_2_NHCON), 2.85 (t, *J* = 7.5 Hz, 2H, CH_2_NH_3_^+^), 2.78 (t, *J* = 7.2 Hz, 2H, TrizCH_2_), 1.76 (quin, *J* = 7.2 Hz, 2H, TrizCH_2_CH_2_), 1.63 (quin, *J* = 7.8 Hz, 2H, CH_2_CH_2_NH_3_^+^); ^13^C NMR (DMSO-*d*_6_, 100 MHz) δ (ppm): 162.1 (NHCON), 157.9 (OC_Ar_), 155.8 (C_Ar_), 148.0 (C_Ar_), 147.7 (C_Ar_), 139.4 (C_Ar_), 139.4 (C_Ar_), 138.3 (C_Ar_), 133.9 (C_Ar_), 133.7 (C_Ar_), 131.5 (CH_Ar_), 129.3 (CH_Ar_), 129.2 (CH_Ar_), 129.1 (CH_Ar_), 129.0 (CH_Ar_), 128.8 (CH_Ar_), 128.5 (CH_Ar_), 127.6 (CH_Ar_), 126.5 (CH_Ar_), 126.5 (CH_Ar_), 121.1 (CH_Ar_), 120.4 (C_Ar_), 112.1 (CH_Ar_), 104.3 (CH_Ar_), 67.4 (OCH_2_), 45.1 (CH_2_CH_2_NHCON), 42.4 (OCH_2_CH_2_), 38.6 (CH_2_NH_3_^+^), 37.5 (CH_2_CH_2_NHCON), 26.5 (CH_2_CH_2_NH_3_^+^), 25.5 (TrizCH_2_CH_2_), 24.4 (TrizCH_2_); HPLC (gradient A, Agilent): *R*_t_ = 9.3 min; HRMS (ES, positive mode) *m*/*z*: calcd for C_38_H_37_N_7_O_2_S 655.2729; found 655.2748 (2.87 ppm); anal. calcd for C_38_H_38_N_7_O_2_S·TFA: C, 62.41; H, 4.98; N, 12.74; S, 4.16; found: C, 62.36; H, 5.06; N, 12.37; S, 4.25.

#### 1-(2-(5-(4-(4-Ammoniobutyl)-1*H*-1,2,3-triazol-1-yl)-2-(4-([4-phenyloxyphenyl]-1-yl)-5-phenylthiazol-2-yl) phenoxy)ethyl)imidazolidin-2-one 2,2,2-Trifluoroacetate (**19c**)

Following the general Cbz deprotection procedure, **18c** (120 mg, 0.15 mmol), Pd/C 10% (24 mg), and TFA (0.9 mL) were reacted. Workup and purification yielded **19c** (44 mg, 37%) as a colorless oil. ^1^H NMR (DMSO-*d*_6_, 400 MHz) δ (ppm): 8.78 (s, 1H, Ar), 8.53 (d, *J* = 8.6 Hz, 1H, Ar), 7.79 (d, *J* = 2.1 Hz, 1H, Ar), 7.83–7.72 (m, 3H, NH_3_^+^), 7.71 (dd, *J* = 8.6, 2.0 Hz, 1H, Ar), 7.56 (d, *J* = 8.7 Hz, 2H, Ar), 7.50–7.32 (m, 7H, Ar), 7.17 (t, *J* = 7.4 Hz, 1H, Ar), 7.06 (d, *J* = 7.9 Hz, 2H, Ar), 6.98 (d, *J* = 8.7 Hz, 2H, Ar), 6.39 (bs, 1H, NHCON), 4.51 (t, *J* = 5.6 Hz, 2H, OCH_2_), 3.65 (t, *J* = 5.6 Hz, 2H, OCH_2_CH_2_), 3.50 (dd, *J* = 9.0, 6.7 Hz, 2H, CH_2_CH_2_NHCON), 3.22 (t, *J* = 7.9 Hz, 2H, CH_2_CH_2_NHCON), 2.91–2.80 (m, 2H, CH_2_NH_3_^+^), 2.77 (t, *J* = 7.2 Hz, 2H, TrizCH_2_), 1.76 (quin, *J* = 7.2 Hz, 2H, TrizCH_2_CH_2_), 1.64 (quin, *J* = 8.1 Hz, 2H, CH_2_CH_2_NH_3_^+^); ^13^C NMR (DMSO-*d*_6_, 100 MHz) δ (ppm): 162.1 (NHCON), 158.1 (CF_3_COO^–^), 157.8 (C_Ar_), 156.5 (OC_Ar_), 156.2 (OC_Ar_), 155.5 (OC_Ar_), 147.9 (C_Ar_), 147.7 (C_Ar_), 138.3 (C_Ar_), 133.3 (C_Ar_), 131.5 (C_Ar_), 130.4 (CH_Ar_), 130.1 (CH_Ar_), 129.8 (CH_Ar_), 129.2 (CH_Ar_), 129.0 (CH_Ar_), 128.8 (CH_Ar_), 128.3 (C_Ar_), 123.8 (CH_Ar_), 121.1 (C_Ar_), 120.4 (CH_Ar_), 119.0 (CH_Ar_), 118.1 (CH_Ar_), 115.8 (CF_3_COO^–^), 112.1 (CH_Ar_), 104.3 (CH_Ar_), 67.3 (OCH_2_), 45.1 (CH_2_CH_2_NHCON), 42.4 (OCH_2_CH_2_), 38.6 (CH_2_NH_3_^+^), 37.5 (CH_2_CH_2_NHCON), 26.5 (CH_2_CH_2_NH_3_^+^), 25.5 (TrizCH_2_CH_2_), 24.4 (TrizCH_2_); HPLC (gradient A, Agilent): *R*_t_ = 9.8 min; HRMS (ES, positive mode) *m*/*z*: calcd for C_38_H_37_N_7_O_3_S 671.2679; found 671.2673 (−0.81 ppm); anal. calcd for C_38_H_38_N_7_O_3_S·TFA: C, 61.14; H, 4.87; N, 12.48; S, 4.08; found: C, 61.55; H, 4.99; N, 12.12; S, 4.34.

#### 1-(2-(2-(4-(2-([1,1′-Biphenyl]-4-yl)ethyl)-5-phenyl-thiazol-2-yl)-5-(4-(4-ammoniobutyl)-1*H*-1,2,3-triazol-1-yl)phenoxy)ethyl)imidazolidin-2-one 2,2,2-Trifluoroacetate (**19d**)

Following the general hydrogenation procedure, a solution of **18d** (91 mg, 0.11 mmol), Pd/C 10% (18 mg), and TFA (0.7 mL) was reacted to give, after purification, **19d** (14 mg, 16%) as a colorless oil. ^1^H NMR (DMSO-*d*_6_, 400 MHz) δ (ppm): 8.78 (s, 1H, Ar), 8.54 (d, *J* = 8.6 Hz, 1H, Ar), 7.78 (d, *J* = 2.1 Hz, 1H, Ar), 7.72 (dd, *J* = 8.5, 2.0 Hz, 1H, Ar), 7.73–7.65 (m, 3H, NH_3_^+^), 7.62 (d, *J* = 7.2 Hz, 2H, Ar), 7.54 (d, *J* = 8.2 Hz, 2H, Ar), 7.49–7.38 (m, 7H, Ar), 7.33 (tt, *J* = 7.4, 1.3 Hz, 1H, Ar), 7.26 (d, *J* = 8.2 Hz, 2H, Ar), 6.39 (bs, 1H, NHCON), 4.50 (t, *J* = 5.6 Hz, 2H, OCH_2_), 3.65 (t, *J* = 5.6 Hz, 2H, OCH_2_CH_2_), 3.49 (dd, *J* = 8.9, 6.7 Hz, 2H, CH_2_CH_2_NHCON), 3.21 (t, *J* = 7.9 Hz, 2H, CH_2_CH_2_NHCON), 3.16–3.12 (m, 4H, ThiazCH_2_CH_2_), 2.85 (t, *J* = 7.5 Hz, 2H, CH_2_NH_3_^+^), 2.78 (t, *J* = 7.2 Hz, 2H, TrizCH_2_), 1.76 (quin, *J* = 7.6 Hz, 2H, TrizCH_2_CH_2_), 1.63 (quin, *J* = 7.5 Hz, 2H, CH_2_CH_2_NH_3_^+^); ^13^C NMR (DMSO-*d*_6_, 100 MHz) δ (ppm): 162.1 (NHCON), 157.4 (C_Ar_), 155.6 (OC_Ar_), 150.1 (C_Ar_), 147.7 (C_Ar_), 140.7 (C_Ar_), 140.1 (C_Ar_), 138.1 (C_Ar_), 137.9 (C_Ar_), 133.4 (C_Ar_), 131.4 (C_Ar_), 129.0 (CH_Ar_), 128.9 (CH_Ar_), 128.9 (CH_Ar_), 128.9 (CH_Ar_), 128.8 (CH_Ar_), 128.0 (CH_Ar_), 127.2 (CH_Ar_), 126.6 (CH_Ar_), 126.5 (CH_Ar_), 121.3 (C_Ar_), 120.4 (CH_Ar_), 112.1 (CH_Ar_), 104.3 (CH_Ar_), 67.4 (OCH_2_), 45.2 (CH_2_CH_2_NHCON), 42.4 (OCH_2_CH_2_), 38.7 (CH_2_NH_3_^+^), 37.5 (CH_2_CH_2_NHCON), 34.6 (ThiazCH_2_CH_2_), 31.2 (ThiazCH_2_CH_2_), 26.5 (CH_2_CH_2_NH_3_^+^), 25.6 (TrizCH_2_CH_2_), 24.4 (TrizCH_2_); HPLC (gradient A, Agilent): *R*_t_ = 9.8 min; HRMS (ES, positive mode) *m*/*z*: calcd for C_40_H_41_N_7_O_2_S 683.3042; found 683.3050 (1.06 ppm); anal. calcd for C_40_H_42_N_7_O_2_S·TFA: C, 63.22; H, 5.31; N, 12.29; S, 4.02; found: C, 63.40; H, 5.64; N, 12.50; S, 3.93.

#### 1-(2-(2-(4-(2-([4-Phenyloxyphenyl]-1-yl)ethyl)-5-phenyl-thiazol-2-yl)-5-(4-(4-ammoniobutyl)-1*H*-1,2,3-triazol-1-yl)phenoxy)ethyl)imidazolidin-2-one 2,2,2-Trifluoroacetate (**19e**)

Following the general Cbz deprotection procedure, reaction of **18e** (80 mg, 0.10 mmol), Pd/C 10% (16 mg), and TFA (0.6 mL) afforded **19e** (20 mg, 26%) as a colorless oil. ^1^H NMR (DMSO-*d*_6_, 400 MHz) δ (ppm): 8.78 (s, 1H, Ar), 8.56–8.50 (m, 1H, Ar), 7.77 (d, *J* = 2.0 Hz, 1H, Ar), 7.81–7.64 (m, 4H, Ar, NH_3_^+^), 7.62 (d, *J* = 7.2 Hz, 1H, Ar), 7.54 (d, *J* = 8.1 Hz, 1H, Ar), 7.50–7.31 (m, 7H, Ar), 7.26 (d, *J* = 7.9 Hz, 1H, Ar), 7.16 (d, *J* = 8.5 Hz, 1H, Ar), 7.10 (t, *J* = 7.4 Hz, 1H, Ar), 6.94 (d, *J* = 8.0 Hz, 1H, Ar), 6.90 (d, *J* = 8.5 Hz, 1H, Ar), 6.39 (bs, 1H, NHCON), 4.49 (t, *J* = 5.7 Hz, 2H, OCH_2_), 3.64 (t, *J* = 5.7 Hz, 2H, OCH_2_CH_2_), 3.48 (dd, *J* = 9.0, 6.6 Hz, 2H, CH_2_CH_2_NHCON), 3.21 (t, *J* = 7.9 Hz, 2H, CH_2_CH_2_NHCON), 3.15–3.06 (m, 4H, ThiazCH_2_CH_2_), 2.85 (t, *J* = 7.5 Hz, 2H, CH_2_NH_3_^+^), 2.78 (t, *J* = 7.2 Hz, 2H, TrizCH_2_), 1.75 (quin, *J* = 7.4 Hz, 2H, TrizCH_2_CH_2_), 1.64 (quin, *J* = 7.0 Hz, 2H, CH_2_CH_2_NH_3_^+^); ^13^C NMR (DMSO-*d*_6_, 100 MHz) δ (ppm): 162.1 (NHCON), 157.3 (C_Ar_), 157.1 (C_Ar_), 155.6 (OC_Ar_), 154.6 (C_Ar_), 150.0 (C_Ar_), 147.7 (C_Ar_), 140.7 (C_Ar_), 138.1 (C_Ar_), 137.9 (C_Ar_), 136.6 (C_Ar_), 133.4 (C_Ar_), 131.4 (CH_Ar_), 129.9 (CH_Ar_), 129.8 (CH_Ar_), 129.0 (CH_Ar_), 129.0 (CH_Ar_), 128.9 (CH_Ar_), 128.9 (CH_Ar_), 128.9 (CH_Ar_), 128.0 (CH_Ar_), 126.6 (CH_Ar_), 126.5 (CH_Ar_), 123.1 (CH_Ar_), 121.3 (C_Ar_), 118.8 (CH_Ar_), 118.1 (CH_Ar_), 112.1 (CH_Ar_), 104.3 (CH_Ar_), 67.4 (OCH_2_), 45.2 (CH_2_CH_2_NHCON), 42.4 (OCH_2_CH_2_), 38.7 (CH_2_NH_3_^+^), 37.5 (CH_2_CH_2_NHCON), 34.6 (CH_2_CH_2_PhOPh), 31.2 (CH_2_CH_2_PhOPh), 26.5 (CH_2_CH_2_NH_3_^+^), 25.6 (TrizCH_2_CH_2_), 24.4 (TrizCH_2_); HPLC (gradient A, Agilent): *R*_t_ = 9.7 min; HRMS (ES, positive mode) *m*/*z*: calcd for C_40_H_41_N_7_O_3_S 699.2992; found 699.2993 (0.17 ppm); anal. calcd for C_40_H_42_N_7_O_3_S·TFA: C, 61.98; H, 5.20; N, 12.05; S, 3.94; found: C, 62.23; H, 5.34; N, 11.68; S, 3.50.

#### 1-(3-(2-Bromo)acetylphenyl)-2-bromoethan-1-one (**20**)

Following the general procedure described for the synthesis α-bromomethylketones **17a**–**e**, *p*-toluensulfonic acid (761 mg, 4.00 mmol) and NBS (713 mg, 4.00 mmol) were successively added to a solution of commercially available diacetyl benzene (250 mg, 1.54 mmol) in CH_3_CN (20 mL) and stirred at room temperature overnight. After the workup, the residue was purified by flash column chromatography (hexane/EtOAc, 90:10) to give **20** (366 mg, 74%) as a white solid.^51^ m.p.: 87–88 °C; ^1^H NMR (CDCl_3_, 400 MHz) δ (ppm): 8.55 (t, *J* = 1.8 Hz, 1H, Ar), 7.20 (dd, *J* = 7.8, 1.8 Hz, 2H, Ar), 7.64 (t, *J* = 7.8 Hz, 1H, Ar), 4.78 (s, 4H, BrCH_2_CO).

#### Dibenzyl-((((1,3-phenylenebis(thiazole-4,2-diyl))bis(3-(2-(2-oxoimidazolidin-1-yl)ethoxy)-4,1-phenylene))bis(1*H*-1,2,3-triazole-1,4-diyl))bis(butane-4,1-diyl))dicarbamate (**21**)

Following the general procedure of Hantzsch synthesis, **9** (100 mg, 0.19 mmol) was reacted with α-bromoketone **20** (30 mg, 0.09 mmol) in *^i^*PrOH (15 mL). After the workup, the residue was purified by CCTLC on the Chromatotron (CH_2_Cl_2_/MeOH, 96:4) to give **21** (68 mg, 30%) as a pink oil. ^1^H NMR (DMSO-*d*_6_, 400 MHz) δ (ppm): 8.75 (s, 2H, Ar), 8.68 (d, *J* = 8.5 Hz, 2H, Ar), 8.37 (s, 2H, Ar), 8.12 (dd, *J* = 7.6, 1.7 Hz, 2H, Ar), 7.79 (d, *J* = 2.0 Hz, 2H, Ar), 7.75 (dd, *J* = 8.7, 1.8 Hz, 2H, Ar), 7.60 (t, *J* = 7.7 Hz, 1H, Ar), 7.46–7.10 (m, 12H, Ar, NHCbz), 6.42 (s, 2H, NHCON), 5.01 (s, 4H, NHCOOCH_2_), 4.52 (t, *J* = 5.6 Hz, 4H, OCH_2_), 3.69 (t, *J* = 5.6 Hz, 4H, OCH_2_CH_2_), 3.53 (dd, *J* = 9.0, 6.7 Hz, 4H, CH_2_CH_2_NHCON), 3.25 (t, *J* = 7.9 Hz, 4H, CH_2_CH_2_NHCON), 3.07 (q, *J* = 6.5 Hz, 4H, CH_2_NHCbz), 2.74 (t, *J* = 7.1 Hz, 4H, TrizCH_2_), 1.70 (quin, *J* = 7.6 Hz, 4H, TrizCH_2_CH_2_), 1.52 (quin, *J* = 7.2 Hz, 4H, CH_2_CH_2_NHCbz); ^13^C NMR (DMSO-*d*_6_, 100 MHz) δ (ppm): 162.1 (NHCON), 160.2 (C_Ar_), 156.1 (OC_Ar_), 155.9 (NHCOO), 153.3 (C_Ar_), 148.1 (C_Ar_), 138.3 (C_Ar_), 137.3 (CH_Ar_), 134.7 (C_Ar_), 129.3 (CH_Ar_), 128.3 (CH_Ar_), 127.7 (CH_Ar_), 126.0 (C_Ar_), 123.7 (C_Ar_), 121.1 (CH_Ar_), 120.3 (CH_Ar_), 116.4 (CH_Ar_), 112.1 (CH_Ar_), 104.3 (CH_Ar_), 67.6 (OCH_2_), 65.1 (NHCOOCH_2_), 45.3 (CH_2_CH_2_NHCON), 42.4 (OCH_2_CH_2_), 37.6 (CH_2_CH_2_NHCON), 28.9 (CH_2_CH_2_NHCbz), 26.0 (TrizCH_2_CH_2_), 24.7 (TrizCH_2_); HRMS (ES, positive mode) *m*/*z*: calcd for C_62_H_64_N_14_O_8_S_2_ 1196.4473; found 1196.4482 (0.72 ppm).

#### 1,1′-((((1,3-Phenylenebis(thiazole-4,2-diyl))bis(5-(4-(4-ammoniobutyl)-1*H*-1,2,3-triazol-1-yl)-2,1-phenylene)) bis(oxy))bis(ethane-2,1-diyl))bis(imidazolidin-2-one)bis(2,2,2-trifluoroacetate) (**22**)

Following the general Cbz removal procedure, **21** (120 mg, 0.11 mmol), Pd/C 10% (24 mg), and TFA (1.0 mL) reacted to gave, after workup and purification, compound **22** (22 mg, 19%) as a colorless oil. ^1^H NMR (DMSO-*d*_6_, 400 MHz) δ (ppm): 8.81 (s, 2H, Ar), 8.75 (s, 1H, Ar), 8.69 (d, *J* = 8.5 Hz, 2H, Ar), 8.40 (s, 2H, Ar), 8.13 (dd, *J* = 7.8, 1.7 Hz, 2H, Ar), 7.94–7.67 (m, 10H, Ar, NH_3_^+^), 7.62 (t, *J* = 7.7 Hz, 1H, Ar), 6.42 (bs, 2H, NHCON), 4.54 (t, *J* = 5.7 Hz, 4H, OCH_2_), 3.69 (t, *J* = 5.6 Hz, 4H, OCH_2_CH_2_), 3.53 (dd, *J* = 8.9, 6.7 Hz, 4H, CH_2_CH_2_NHCON), 3.25 (t, *J* = 7.9 Hz, 4H, CH_2_CH_2_NHCON), 2.90–2.80 (m, 4H, CH_2_NH_3_^+^), 2.78 (t, *J* = 7.3 Hz, 4H, TrizCH_2_), 1.76 (quin, *J* = 7.4 Hz, 4H, TrizCH_2_CH_2_), 1.64 (quin, *J* = 7.7 Hz, 4H, CH_2_CH_2_NH_3_^+^); ^13^C NMR (DMSO-*d*_6_, 100 MHz) δ (ppm): 162.1 (NHCON), 160.2 (OC_Ar_), 158.0 (CF_3_COO^–^), 155.9 (C_Ar_), 153.3 (C_Ar_), 147.7 (C_Ar_), 138.2 (C_Ar_), 134.7 (CH_Ar_), 129.4 (CH_Ar_), 126.0 (CH_Ar_), 123.1 (C_Ar_), 121.2 (C_Ar_), 120.5 (CH_Ar_), 116.5 (CH_Ar_), 112.1 (CH_Ar_), 104.3 (CH_Ar_), 67.6 (OCH_2_), 45.3 (CH_2_CH_2_NHCON), 42.4 (OCH_2_CH_2_), 38.6 (CH_2_NH_3_^+^), 37.6 (CH_2_CH_2_NHCON), 26.5 (CH_2_CH_2_NH_3_^+^), 25.6 (TrizCH_2_CH_2_), 24.4 (TrizCH_2_); HPLC (gradient A, Agilent): *R*_t_ = 6.4 min; HRMS (ES, positive mode) *m*/*z*: calcd for C_46_H_52_N_14_O_4_S_2_ 928.3737; found 928.3740 (0.27 ppm); anal. calcd for C_46_H_52_N_14_O_4_S_2_.2TFA: C, 51.90; H, 4.70; N, 16.95; S, 5.54; found: C, 51.38; H, 5.16; N, 16.98; S, 5.83.

### Biological Methods

#### Chemical Compounds

For *Li*TryR oxidoreductase activity and dimer quantitation assays, stock solutions of synthesized compounds were prepared in anhydrous DMSO at 5 mM. For the leishmanicidal and cytotoxicity assays, stock solutions of synthesized compounds were prepared in anhydrous DMSO at 15 mM. All reagents for the *Li*TryR activity assay were obtained from Sigma-Aldrich (St. Louis, MO) except for TS_2_, which was purchased from Bachem (Bubendorf, Switzerland).

#### *Li*TryR Purification

Recombinant *L. infantum* TryR (*Li*TryR) HIS-tagged and HIS-FLAG-tagged versions were used for all *Li*TryR oxidoreductase activity and dimer quantitation assays. These *Li*TryR versions were purified from *Escherichia coli* as previously described.^25^ Briefly, *pRSETA-HIS-LiTryR* construct alone or in combination with the *pET24a-FLAG-LiTryR* construct was transformed into BL21 (DE3) Rossetta *E. coli* strain. An overnight *E. coli* culture grown at 37 °C in Luria–Bertani (LB) medium with the appropriate antibiotics and vigorous shaking was diluted (1:100) in the same medium and allowed to grow in the same conditions until the OD_600_ was 0.5. Then, *Li*TryR expression was induced by addition of 1 mM isopropyl-β-d-1-thiogalactopyranoside (IPTG) for 16 h at 26 °C. The cells were centrifuged for 5 min at 9000*g* and 4 °C and the wet pellet resuspended in lysis buffer (50 mM Tris, pH 7, 300 mM NaCl, 25 mM imidazole, protease inhibitor cocktail, and 1 mg/mL lysozyme). Following a 30 min incubation on ice, the cell lysate was sonicated on wet ice (50% pulses, potency 7) for 30 min using a Sonifier Cell Disruptor B15 (Branson, Danbury, CT) and centrifuged for 1 h at 50 000*g* and 4 °C. The supernatant was sonicated again as previously described for 10 min and loaded on a HisTrap column (GE Healthcare, Chicago, IL) for 16 h at 4 °C using a P-1 peristaltic pump (GE Healthcare, Chicago, IL). Once loaded, the HisTrap column was connected to an ÄKTA purifier UPC 10 (GE Healthcare, Chicago, IL) and washed using a 5–10% gradient between buffer A (50 mM Tris, pH 7 and 300 mM NaCl) and B (50 mM Tris, pH 7, 300 mM NaCl, and 500 mM imidazole). *Li*TryR was eluted using a 40% gradient between buffers A and B. Fractions containing recombinant *Li*TryR were pooled and loaded into a HiPrep 26/10 Desalting column (GE Healthcare, Chicago, IL) previously equilibrated with buffer A. Finally, *Li*TryR was concentrated to 2 mg/mL using an Amicon Ultra-15 50K (Merck Millipore, Burlington, MA) and an equal volume of glycerol was added before storing at −20 °C.

#### *Tb*TryR and *Tco*TryR Purification

The DNA coding sequences for *Homo sapiens* GR, *T. brucei* TryR, and *T. congolense* TryR were polymerase chain reaction (PCR)-amplified and cloned in the pRSETA plasmid. The DNA coding for hGR (transcript variant 1; NCBI Reference Sequence: NM_000637.5) was purchased from GeneScript. Genomic DNA from *T. congolense* parasites was kindly provided by Dr. Stefan Magez (University of Vrije, Brussels). Genomic DNA from the *T. brucei* S16 cell line was kindly provided by José M. Pérez-Victoria. Instituto de Parasitología y Biomedicina “López—Neyra” CSIC. HIS-tagged recombinant *T. brucei* and *T. congolense* TryRs (*Tb*TryR and *Tco*TryR, respectively) were purified from *E. coli* as already explained for *Li*TryR.

#### TryR Oxidoreductase Activity

TryR oxidoreductase activity was determined spectrophotometrically using a modified version of the DTNB-coupled assay described by Hamilton et al.^52^ Briefly, reactions were carried out at 26 °C in 250 μL of *N*-(2-hydroxyethyl)piperazine-*N*′-ethanesulfonic acid (HEPES) buffer (pH 7.5, 40 mM) containing ethylenediaminetetraacetic acid (EDTA) (1 mM), NADP^+^ (30 μM), DTNB (25 μM), TS_2_ (1 μM), NADPH (150 μM), glycerol (0.02%), DMSO (1.75%), and recombinant TryR (7 nM). DTNB, glycerol, and DMSO concentrations used in this assay do not have any relevant effect on the kinetics of the enzymatic reaction. Reactions were started by addition of a mixture of NADPH and TS_2._

TryR oxidoreductase activity was monitored at 26 °C by an increase in absorbance at 412 nm in an EnSpire Multimode Plate Reader (PerkinElmer, Waltham, MA). 2-Nitro-5-meta mercaptobenzoic acid (TNB) concentration was obtained by multiplying the absorbance values by 100 (50 μM TNB generates 0.5 arbitrary units of absorbance at 412 nm). In this DTNB-coupled assay, one molecule of T(SH)_2_ reduces one molecule of DTNB, producing two TNB molecules. All of the assays were conducted in at least three independent experiments. Data were analyzed using a nonlinear regression model with GraFit 6 software (Erithacus, Horley, Surrey, U.K.).

#### hGR Oxidoreductase Activity

hGR oxidoreductase activity was determined spectrophotometrically using a DTNB-coupled assay. Briefly, reactions were carried out at 37 °C in 250 μL of HEPES buffer (pH 7.5, 40 mM) containing EDTA (1 mM), NADP^+^, (60 μM), DTNB (150 μM), oxidized glutathione (50 μM), NADPH (300 μM), glycerol (0.02%), DMSO (1.75%), and recombinant hGR (7 nM). DTNB, glycerol, and DMSO concentrations used in this assay do not have any relevant effect on the kinetics of the enzymatic reaction. hGR oxidoreductase activity was monitored at 37 °C by an increase in absorbance at 412 nm using an EnSpire Multimode Plate Reader (PerkinElmer, Waltham, MA). TNB concentration was obtained by multiplying the absorbance values by 100 (50 μM TNB generates 0.5 arbitrary units of absorbance at 412 nm). All of the assays were conducted in at least three independent experiments. Data were analyzed using a nonlinear regression model with GraFit 6 software (Erithacus, Horley, Surrey, U.K.).

#### Dimer Quantitation Assay

The stability of the *Li*TryR dimeric form in the presence of 1,2,3-triazole-phenyl-thiazole-based compounds was evaluated using the novel enzyme-linked immunosorbent assay (ELISA) developed in our laboratory.^25^ Briefly, HIS-FLAG-tagged *Li*TryR (400 nM) was incubated in dimerization buffer (1 mL of 300 mM NaCl, 50 mM Tris, pH 8.0) for 16 h at 37 °C with agitation and in a humid atmosphere in the presence of the different compounds ranging from 75 to 3.12 μM. Next, the different solutions were centrifuged at 18 000*g* for 15 min at room temperature. The supernatants (200 μL/well) were added to the α-FLAG-coated plates (Sigma-Aldrich, St. Louis, MO) and incubated for 30 min at 37 °C with agitation in a humid atmosphere. The plates were washed five times with TTBS buffer (2 mM Tris, pH 7.6, 138 mM NaCl, and 0.1% Tween-20) and incubated with diluted monoclonal α-HIS horseradish peroxidase (HRP)-conjugated antibody (200 μL, 1:50 000, Abcam, Cambridge, U.K.) in fatty acid- and essentially globulin-free bovine serum albumin (BSA) (5%) prepared in TTBS buffer for 1 h at room temperature. The plates were washed five times, and *o*-phenylenediamine dihydrochloride (OPD) substrate prepared according to the manufacturer’s instructions was added. The enzymatic reaction was stopped after 5 min with H_2_SO_4_ (100 μL, 0.5 μM), and the absorbances were measured at 490 nm in an EnSpire Multimode Plate Reader (PerkinElmer, Waltham, MA). All of the assays were conducted in triplicate in at least three independent experiments. Data were analyzed using a nonlinear regression model with GraFit 6 software (Erithacus, Horley, Surrey, U.K.).

#### Determination of **19a** Inhibitory Constants

The inhibitory constants *K*_i_ and *K*_i_* for **19a** were calculated following a slightly modified version of the standard DTNB-coupled assay.^52^ TS_2_ was serially diluted (sixfold dilution from 1666 to 52 μM) in a buffer containing 40 mM HEPES (pH 7.5) and 1 mM EDTA. The different TS_2_ solutions were dispensed (15 μL) in a 96-well microplate. **19a** was serially diluted in DMSO (sixfold dilution from 1875 to 445 μM). The different aliquots of **19a** and the equivalent amount of the vehicle (DMSO) were added to a preassay mixture, yielding different mixtures containing 40 mM HEPES (pH 7.5), 1 mM EDTA, 416.67 μM NADPH, 208.33 μM DTNB, 1.45% DMSO, and different **19a** concentrations ranging from 6.2 to 26 μM. These mixtures (180 μL) were subsequently added to the appropriate wells previously filled with different TS_2_ solutions. The assay was initiated by addition of 55 μL of buffer containing 40 mM HEPES (pH 7.5), 1 mM EDTA, 272.73 μM NADP^+^, 0.01% glycerol, and 3.5 nM recombinant *Li*TryR using an automated dispensing system (PerkinElmer, Waltham, MA). The order of addition was essential to avoid enzyme preincubation with the inhibitor or the substrates. The final 250 μL assay contained 40 mM HEPES buffer (pH 7.5) 1 mM EDTA, 300 μM NADPH, 60 μM NADP^+^, 150 μM DTNB, 6.25–50 μM TS_2_, 1.04% DMSO, 0.002% glycerol, and 0.8 nM recombinant *Li*TryR. TryR oxidoreductase activity was monitored at 26 °C by an increase in absorbance at 412 nm in an EnSpire Multimode Plate Reader (PerkinElmer, Waltham, MA). Coupling an automated dispenser module to the spectrophotometer enabled real-time detection of the enzymatic reaction, which was crucial for high-quality nonlinear fits of the experimental data and, especially, to estimate the initial velocity of the progress curves in the presence of **19a**. To guarantee linearity of the enzyme reaction in the absence of an inhibitor, the analysis was performed with data obtained during the first 20 000 s to avoid TS_2_ depletion. Under this condition, all uninhibited reactions followed straight lines until the generation of 230 μM TNB (Figure S10). This product concentration was never reached in the inhibited reactions during the first 20 000 s. All of the assays were conducted in three independent experiments.

#### Data and Statistical Analysis

GraFit 6.0 software (Erithacus, Horley, SRY, U.K.) was used to perform linear and nonlinear regressions. All experiments were undertaken in triplicate to ensure the reliability of single values.

*k*_obs_ values were estimated for every **19a** concentration at all TS_2_ concentrations by fitting the values of the TNB concentration produced during each enzymatic reaction to eq 1.^42b,53^
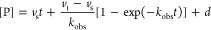
1where *v*_i_ and *v*_s_ are the initial and the steady-state velocities, respectively, *k*_obs_ is the apparent first-order rate constant for the conversion of *v*_i_ into the steady-state velocity (*v*_s_), and *d* is a parameter that indicates the displacement of the curve on the vertical ordinate and takes into account any nonzero value of the measured signal at time zero caused by some of the reagents. The apparent inhibition constants (*K*_i_^app^ and *K*_i_*^app^) for each TS_2_ concentration were determined by fitting the *k*_obs_ values obtained at different **19a** concentrations to eq 2
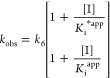
2where *K*_i_^app^ is the apparent value of the *K*_i_ for the initial encounter complex (EI) and *K*_i_*^app^ is the apparent value of the overall inhibition constant *K*_i_* to form a higher-affinity complex (E*I). *k*_6_ is the first-order rate constant that defines the reverse isomerization step that returns E*I to EI.^42a,53^

The *K*_i_ value for the formation of the EI complex was determined by fitting the *K*_i_^app^ values obtained at different TS_2_ concentrations to eq 3. In addition, this graph was used to assess the inhibition modality of **19a** during the first equilibrium of its two-step binding mechanism. The value obtained for α defines the mode of inhibition (competitive, α → infinite; uncompetitive, α → 0; pure noncompetitive, α = 1; mixed, 1 < α < 10).^42a^
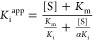
3The overall inhibition constant *K*_i_* value was determined by fitting the *K*_i_*^app^ values obtained at different TS_2_ concentrations to eq 4. In addition, this graph was used to assess the overall inhibition modality of **19a** during its two-step binding mechanism. As explained above, the value obtained for α defines the mode of inhibition.^42a^
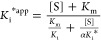
4

#### Leishmania Cell Lines and Culture

*L. infantum* promastigotes (MCAN/ES/89/IPZ229/1/89) were grown in Roswell Park Memorial Institute (RPMI)-1640 medium (Sigma-Aldrich, St. Louis, MO) supplemented with 10% heat-inactivated fetal calf serum (FCS), antibiotics, and 25 mM HEPES (pH 7.2) at 26 °C. *L. infantum* axenic amastigotes (MCAN/ES/89/IPZ229/1/89) were grown in M199 medium (Invitrogen, Leiden, The Netherlands) supplemented with 10% heat-inactivated FCS, 1 g/L β-alanine, 100 mg/L l-asparagine, 200 mg/L sucrose, 50 mg/L sodium pyruvate, 320 mg/L malic acid, 40 mg/L fumaric acid, 70 mg/L succinic acid, 200 mg/L α-ketoglutaric acid, 300 mg/L citric acid, 1.1 g/L sodium bicarbonate, 5 g/L morpholineethanesulfonic acid (MES), 0.4 mg/L hemin, and 10 mg/L gentamicine, pH 5.4, at 37 °C.

#### Axenization of *L. infantum* Promastigotes

Axenization was performed by diluting 0.5 mL of a 7-day stationary phase culture of *L. infantum* promastigotes (2–3 × 10^7^ parasites/mL) in 4.5 mL of amastigotes medium and incubating the culture at 37 °C for 2 or 3 days. *L. infantum* axenic amastigotes apparition was followed by phase-contrast microscopy using an Eclipse Ti inverted microscope (Nikon, Tokyo, Japan).

#### Leishmanicidal Activity

Drug treatment of *L. infantum* promastigotes was performed during the logarithmic growth phase at a concentration of 2 × 10^6^ parasites/mL at 26 °C for 24 h. Drug treatment of *L. infantum* axenic amastigotes was performed during the logarithmic growth phase at a concentration of 1 × 10^6^ parasites/mL at 37 °C for 24 h. EC_50_ was evaluated by flow cytometry by the propidium iodide (PI) exclusion method. After selection of the parasite population based on their forward scatter (FSC) and side scatter (SSC) values, live and dead parasite cells were identified by their permeability to PI. All of the assays were conducted in triplicate in at least three independent experiments. Data were analyzed using a nonlinear regression model with GraFit 6 software (Erithacus, Horley, Surrey, U.K.).

#### Fluorescence Microscopy

Logarithmic growth phase promastigotes were treated with 25 μM **12a** for 1 h at 26 °C. Parasites were fixed using a solution containing EDTA (450 mM) and mounted on polylysine slides (Thermo Scientific, Waltham, MA). Images were captured with an Eclipse Ti inverted microscope (Nikon, Tokyo, Japan).

#### Human Cell Line Culture

THP-1 cells were grown in RPMI-1640 medium (Gibco, Leiden, The Netherlands) supplemented with 10% heat-inactivated FCS, antibiotics, 10 mM HEPES, 2 mM glutamine, and 1 mM sodium pyruvate, pH 7.2, at 37 °C and 5% CO_2_.

Liver hepatocellular carcinoma HepG2 cells were grown in Dulbecco’s modified Eagle’s medium (DMEM) (Sigma-Aldrich, St. Louis, MO) with 10% heat-inactivated FCS, antibiotics, and 10 mM HEPES.

#### Determination of Cellular Toxicity

Drug treatment of liver hepatocellular carcinoma HepG2 cells was performed at a concentration of 10^5^ cells/mL at 37 °C and 5% CO_2_ for 24 h. EC_50_ was evaluated by the crystal violet assay. Briefly, cells were washed with phosphate-buffered saline (PBS) and stained with 200 μL of crystal violet solution (0.2% crystal violet, 2% ethanol) for 10 min at room temperature. Next, the plates were washed twice with tap water and allowed to dry. The stained cells were solubilized with 400 μL of 1% sodium dodecyl sulfate (SDS), and the color intensity was quantified at 570 nm using an EnSpire Multimode Plate Reader (PerkinElmer, Waltham, MA). All of the assays were conducted in triplicate in at least three independent experiments. Data were analyzed using a nonlinear regression model with GraFit 6 software (Erithacus, Horley, Surrey, U.K.).

#### *In Vitro* Infection of THP-1-Derived Macrophages with GFP-Expressing *L. infantum* Axenic Amastigotes

THP-1 monocytes were seeded in a 48-well plate at a concentration of 5 × 10^5^ cells/mL (250 μL/well) in RPMI complete medium supplemented with phorbol 12-myristate 13-acetate (PMA) (100 ng/mL). Differentiation of THP-1 monocytes to macrophages was allowed for 24 h at 37 °C and 5% CO_2_. THP-1-derived macrophages were infected with GFP-expressing axenic amastigotes at 5 × 10^6^ parasites/mL in RPMI complete medium for 24 h (250 μL/well). Extracellular amastigotes were washed twice with PBS. The remaining extracellular parasites were challenged for 24 h with THP-1 medium supplemented with 10% horse serum (Gibco) instead of FCS. Cells were washed twice with PBS, and treatments were performed in 250 μL of fresh RPMI medium supplemented with 10% horse serum for 72 h at 37 °C and 5% CO_2_. Afterward, treatments were removed by two PBS washes. Lysis of infected cells was performed with 120 μL of SDS 0.005% (w/v) in PBS. After a 30 min incubation at 37 °C, lysis was stopped by adding RPMI complete medium with PI at 20 μg/mL (120 μL/well). The lysates were transferred to a 96-well plate (200 μL/well), and the GFP^+^ amastigotes extracted from the infected cells were acquired by a Beckman Coulter FC500 flow cytometer. Each sample (100 μL) was acquired for 20 s.

Cytotoxicity of THP-1 infected macrophages was determined using an in-house developed lactate dehydrogenase (LDH) activity assay. LDH activity assays from the remaining cell lysates (100 μL/well) were carried out at 37 °C in 250 μL of Tris/HCl buffer (pH 8.9, 50 mM) containing dl-lactate (840 mM), NAD^+^ (450 μM), 1-methoxyphenazine methosulfate (45 μM), and nitro blue tetrazolium chloride (40 μM). The color intensity of the generated formazan dye was monitored at 570 nm using an EnSpire Multimode Plate Reader (PerkinElmer, Waltham, MA). CC_50_ values were calculated using a nonlinear regression model with GraFit 6 software (Erithacus, Horley, Surrey, U.K.).

The relative number of GFP^+^ intracellular amastigotes per cell in each sample was estimated after the division of the total number of GFP+ amastigotes by the LDH activity value of the sample. Results were normalized to the vehicle-treated controls. EC_50_ values of intracellular amastigotes were calculated using a nonlinear regression model with GraFit 6 software (Erithacus, Horley, Surrey, U.K.). All of the assays were conducted in at least three independent experiments.

#### Screening for Pan-Assay Interference Compounds (PAINS) and Aggregation

Screening of all tested compounds for pan-assay interference compounds (PAINS) and aggregation *via* public tools (http://zinc15.docking.org/patterns/home/ and https://www.cbligand.org/PAINS/) gave no hits. Dose–response curves for all experiments and compounds were recorded. None of the curves showed Hill slopes that could be a hint for PAINS (curves for representative compounds **12b** and **12c** are presented in Figure S14, Supporting information). Counter-screening against related (glutathione reductase; GR) and unrelated (superoxide dismutase) targets discarded any nonspecific activity of the compounds. Reaction curves for TryR and GR in the presence of compounds **12b** and **12c** are shown in Figure S15. Nifurtimox is included as a well-characterized inhibitor of GR. SAR, considered the most relevant criterion that distinguishes a PAIN from a non-PAIN, is deeply discussed in the manuscript.^54^

### Computational Methods

#### Automated Ligand Docking

A three-dimensional cubic grid consisting of 65 × 65 × 65 points with a spacing of 0.375 Å centered midway between the two active sites of *Li*TryR was defined for ligand docking. Electrostatic, desolvation, and affinity maps for the atom types present in the ligands of this series were calculated using AutoGrid 4.2.6. Then, the Lamarckian genetic algorithm implemented in AutoDock4^49^ was used to generate up to 100 feasible binding poses of the ligands studied. The best poses were selected on the basis of results from intra- and intermolecular energy evaluations.

#### Molecular Dynamics Simulations

The leaprcff14SB AMBER force field and the graphics processing unit (GPU)-based implementation of the pmemd.cuda module of Amber16 in the single-precision–fixed-precision (SPFP) mode were used, as described before.^30^ The molecular dynamics trajectories were analyzed using the cpptraj module of AmberTools18, and the binding energy analysis was carried out with our in-house tool MM-ISMSA.^55^ The molecular graphics program PyMOL^56^ was employed for molecular editing, visualization, and figure preparation.

## Supporting Information Available

Additional synthesis; biological and computational assays; screening of representative compounds for PAINS; ^1^H NMR, ^13^C NMR, and HRMS spectra and HPLC chromatograms for all of the target compounds (PDF)
Molecular formula strings of the prepared compounds and biological data (CSV)
Movie of *Li*TryR-**19a** complex (Movie S1) (AVI)

## Supplementary Material

jm1c00206_si_002.csv

jm1c00206_si_003.avi

## Abbreviations

CCTLC: centrifugal circular thin-layer chromatography
CuAAC: 1,3-dipolar copper(I)-catalyzed azide–alkyne cycloaddition
EC_50a_: half-maximal effective concentration in amastigotes
EC_50p_: half-maximal effective concentration in promastigotes
HepG2: liver hepatocellular carcinoma
IC_50_^act^: half-maximum inhibitory concentration in the activity assay
IC_50_^dim^: half-maximum inhibitory concentration in the dimerization assay
GR: glutathione reductase
*Li*TryR: *Leishmania infantum* trypanothione reductase
LDH: lactate dehydrogenase
PI: propidium iodide
RuAAC: ruthenium-catalyzed azide–alkyne cycloaddition
SI_a_: selectivity index in amastigotes
SI_p_: selectivity index in promastigotes
*Tb*TryR: *Trypanosoma brucei* trypanothione reductase
*Tco*TryR: *Trypanosoma congolense* trypanothione reductase
Try/TryR: thypanothione/trypanothione reductase
TS_2_: trypanothione disulfide
VL: visceral leishmaniasis
