# Supplementary material for: Efficient Dimerization Disruption of Leishmania infantum Trypanothione Reductase by Triazole-phenyl-thiazoles
Source: J Med Chem. 2021 May 4;64(9):6137–60. doi: 10.1021/acs.jmedchem.1c00206 (PMC8480782; doi:10.1021/acs.jmedchem.1c00206)
Supplement: Supplementary file 1 — jm1c00206_si_001.pdf [file jm1c00206_si_001.pdf]

Supporting Information

Efficient dimerization disruption of *Leishmania infantum* trypanothione reductase by triazole-phenyl-thiazoles

Alejandro Revuelto,[†#] Héctor de Lucio,[‡#] Juan Carlos García Soriano,[‡] Pedro A. Sánchez-Murcia,[§⊥] Federico Gago,[§] Antonio Jiménez-Ruiz,[‡*] María-José Camarasa,[†] and Sonsoles Velázquez[†*]

[†] *Instituto de Química Médica (IQM-CSIC), E-28006 Madrid, Spain*

[‡] *Departamento de Biología de Sistemas, Universidad de Alcalá, E-28805 Alcalá de Henares, Madrid, Spain*

[§] *Área de Farmacología, Departamento de Ciencias Biomédicas, Unidad Asociada al IQM-CSIC, Universidad de Alcalá, E-28805 Alcalá de Henares, Madrid, Spain*

[#] *These two authors contributed equally to this work*

[⊥] ***Present address**: Asst. Prof. Dr. Pedro A. Sánchez Murcia, Division of Physiological Chemistry, Otto Loewi Research Center, Medical University of Graz, Neue Stiftingstalstraße 6/III, A-8010 Graz, Austria (pedro.murcia@medunigraz.at)*

Table of contents

Chemistry

Schemes S1-S3 ……………………………………………..……………...................S4-S6

Synthesis of non-commercially available α-bromomethylketones **10m,p-s** S7-S10

Synthesis of intermediates **5b**, **7a-g**, **8a-g**, **11f-s** and target compounds **12d-s**....S11-S41

Synthesis of truncated analogues **13** and **14**...S41-S42

Biology Methods

Figure S1 and **Table S1.** Noncompetitive hyperbolic inhibition of *Li*TryR by **19a**..........S43

Figures S2-S7. Observed rate constant (k_{obs}) for *Li*TryR inactivation at 6 different TS_2 concentrations ……………………………………………………………….....................S44-S48

Table S2. Rate constants for *Li*TryR time-dependent inhibition by **19a** at six different TS_2 concentrations (Table S2).………………………………………...............................S49

Figure S8. Effect of substrate concentration on the apparent inhibition constants $K_\mathrm{i}^\mathrm{app}$and $K_\mathrm{i}^\mathrm{*app}$ upon binding of **19a**……………………...................................S49

Figure S9. Plot of the k_{obs} values as a function of TS_2 concentration at six different **19a** concentrations ……………………………………………………….......S50

Figure S10. Curves for *Li*TryR enzymatic reactions in the absence of inhibitor……......S51

Figure S11. *Li*TryR inhibition by **12b**………………………………………………….........S52

Table S3. Estimation of the kinetic parameters of *Li*TryR inactivation by **12b** …….........S53

Figure S12. *Li*TryR inhibition by **12q**…………………………………………………….........S54

Table S4. Estimation of the kinetic parameters of *Li*TryR inhibition by **12q** ……….........S54

Scheme S4. Reversible single-step mechanism of time-dependent inhibition of *Li*TryR by **12q** ………………………………………………………………........................S55

Figure S13. Residual activity of *Li*TryR after incubation with **19a**………………….......S55

Scheme S5. Global mechanism for *Li*TryR inactivation ……………………………….....S56

Figures S14 and S15. Screening of representative compounds for PAINS S57

Computational Methods

Figure S16. Calculated contributions of individual *Li*TryR residues to the overall solvent-corrected interaction energy ……………………………………….................................S58

Figure S17A. Theoretical model of *Li*TryR in complex with **19a** …………………..S59-S60

Figure S17B. 19a-induced disruptions in hydrogen bonds involving the Glu436 carboxylates OE1 and OE2 ……………………………….…...........................S59-S60

Table S5. Residues making up the interfacial cavity present in TryRs from trypanosomatids and positionally equivalent residues in hGR……………..……..............S60

^{1}H and ^{13}C NMR spectra, HRMS and HPLC data of target compounds................S61-S115

References..S116

Scheme S1. Synthesis of non-commercially available α-bromomethylketones 10m,p-s

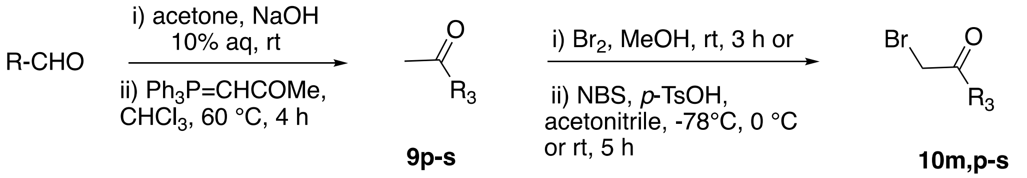

R_3

p (E)-CH=CH-CH_2Ph (**pa**) and (E)-CH_2-CH=CH-Ph (**pb**)
q (E)-CH=CH-Quinolin-6-yl
r (E)-CH=CH-Dihydrobenzofuran-2-yl
s (E)-CH=CH-Dibenzofuran-2-yl

R_3

m -$CH_2{}^t$Bu
p (E)-CH=CH-CH_2Ph
q (E)-CH=CH-Quinolin-6-yl * not isolated
r (E)-CH=CH-Dihydrobenzofuran-2-yl
s (E)-CH=CH-Dibenzofuran-2-yl

Scheme S2. Synthesis of triazole-based type I compounds 12d-s

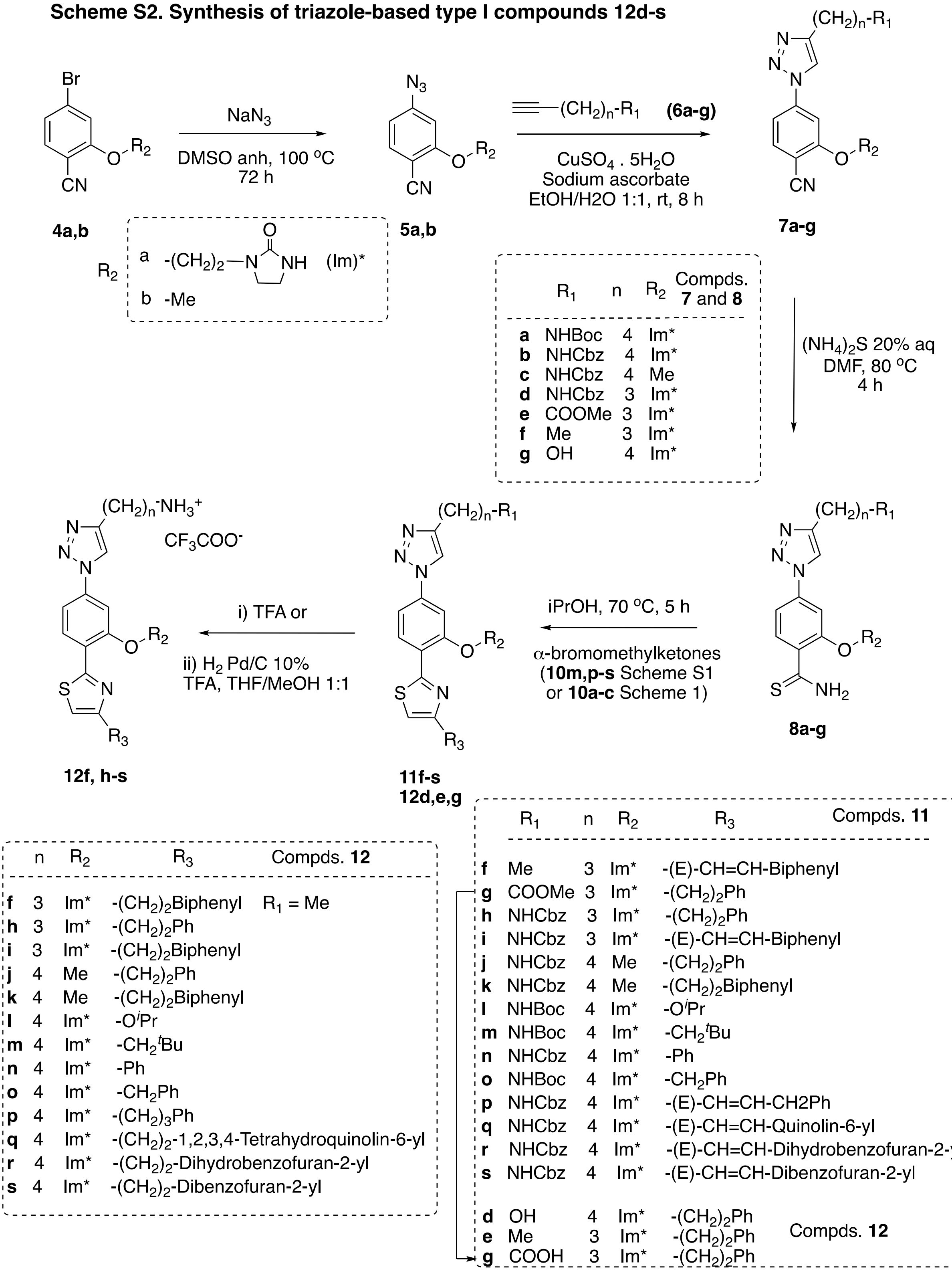

	R_1	n	R_2	Compds. **7** and **8**
**a**	NHBoc	4	Im*	
**b**	NHCbz	4	Im*	
**c**	NHCbz	4	Me	
**d**	NHCbz	3	Im*	
**e**	COOMe	3	Im*	
**f**	Me	3	Im*	
**g**	OH	4	Im*	

	n	R_2	R_3	Compds. **12**
**f**	3	Im*	-$(CH_2)_2$Biphenyl	R_1 = Me
**h**	3	Im*	-$(CH_2)_2$Ph	
**i**	3	Im*	-$(CH_2)_2$Biphenyl	
**j**	4	Me	-$(CH_2)_2$Ph	
**k**	4	Me	-$(CH_2)_2$Biphenyl	
**l**	4	Im*	-O^{i}Pr	
**m**	4	Im*	-CH_2^{t}Bu	
**n**	4	Im*	-Ph	
**o**	4	Im*	-CH_2Ph	
**p**	4	Im*	-$(CH_2)_3$Ph	
**q**	4	Im*	-$(CH_2)_2$-1,2,3,4-Tetrahydroquinolin-6-yl	
**r**	4	Im*	-$(CH_2)_2$-Dihydrobenzofuran-2-yl	
**s**	4	Im*	-$(CH_2)_2$-Dibenzofuran-2-yl	

	R_1	n	R_2	R_3	Compds. **11**
**f**	Me	3	Im*	-(E)-CH=CH-Biphenyl	
**g**	COOMe	3	Im*	-$(CH_2)_2$Ph	
**h**	NHCbz	3	Im*	-$(CH_2)_2$Ph	
**i**	NHCbz	3	Im*	-(E)-CH=CH-Biphenyl	
**j**	NHCbz	4	Me	-$(CH_2)_2$Ph	
**k**	NHCbz	4	Me	-$(CH_2)_2$Biphenyl	
**l**	NHBoc	4	Im*	-O^{i}Pr	
**m**	NHBoc	4	Im*	-CH_2^{t}Bu	
**n**	NHCbz	4	Im*	-Ph	
**o**	NHBoc	4	Im*	-CH_2Ph	
**p**	NHCbz	4	Im*	-(E)-CH=CH-CH2Ph	
**q**	NHCbz	4	Im*	-(E)-CH=CH-Quinolin-6-yl	
**r**	NHCbz	4	Im*	-(E)-CH=CH-Dihydrobenzofuran-2-yl	
**s**	NHCbz	4	Im*	-(E)-CH=CH-Dibenzofuran-2-yl	
**d**	OH	4	Im*	-$(CH_2)_2$Ph	Compds. **12**
**e**	Me	3	Im*	-$(CH_2)_2$Ph	Compds. **12**
**g**	COOH	3	Im*	-$(CH_2)_2$Ph	Compds. **12**

Scheme S3. Synthesis of truncated analogues 13 and 14

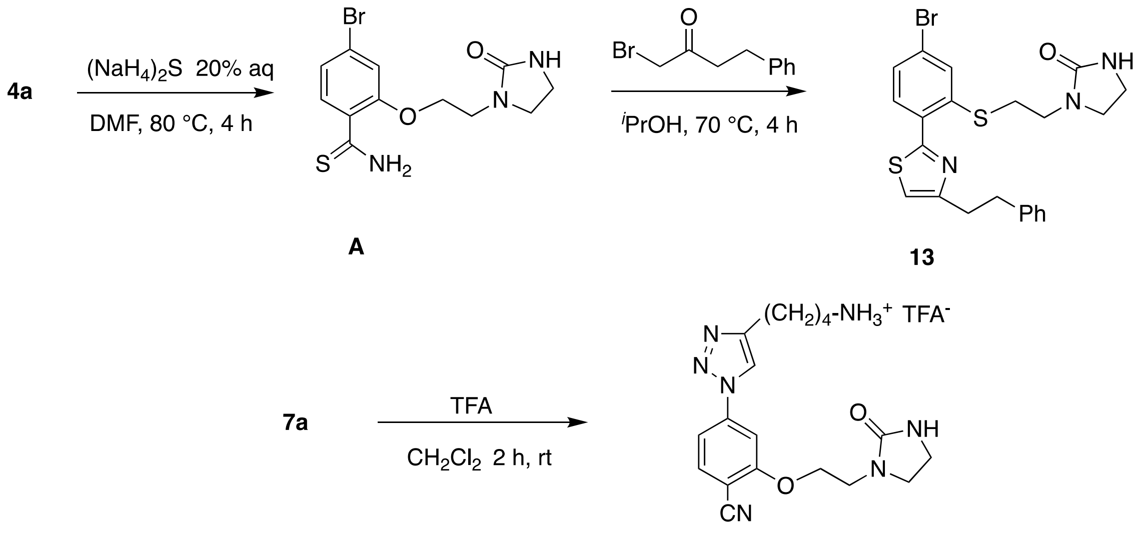

14

Synthesis of non-commercially available α-bromomethylketones

General procedure for the synthesis of methylketones by Wittig reaction (9p-s).

A solution of the corresponding arylaldehyde or alkylaldehyde (1 eq) and 1-(triphenylphosphoranylidene)-2-propanone (0.9 eq) in chloroform (40 mL) was heated at 60 °C for 4 h. The reaction mixture was evaporated under reduced pressure and the residue was purified by flash column chromatography (hexane/EtOAc, 90:10) to obtain the corresponding α,β-unsaturated methylketones **9p-s**.

(E)-5-Phenylpent-3-en-2-one and (E)-5-phenylpent-4-en-2-one (9pa and 9pb). Following the general Wittig procedure, commercial 1-(triphenylphosphoranylidene)-2-propanone (2.87 g, 9.02 mmol) and phenylacetaldehyde (1.17 mL, 9.02 mmol) were reacted. Work-up and purification of the residue gave 1.24 g (86%) of a 75:25 regioisomer mixture of **9pa** and **9pb** (determined by ^{1}H NMR). ^{1}H NMR ($CDCl_3$, 400 MHz) δ (ppm): 7.41 - 7.01 (m, 7H, $Ar^{a,b}$), 7.00 (dt, *J* = 15.9, 1.5 Hz, 0.3H, PhC$\underline{H}^b$=CH), 6.85 (dt, *J* = 15.9, 7.1 Hz, 0.3H, PhCH=C$\underline{H}^b$), 6.83 (dt, *J* = 15.9, 6.8 Hz, 1H, C$\underline{H}^a$=CHCO), 6.00 (dt, *J* = 15.9, 1.6 Hz, 1H, CH=C$\underline{H}^a$CO), 3.46 (dd, *J* = 6.8, 1.6 Hz, 2H, $CH_2{}^a$), 3.25 (dd, *J* = 7.1, 1.3 Hz, 0.5H, $CH_2{}^b$), 2.16 (s, 3H, $CH_3{}^a$), 2.13 (s, 0.7H, $CH_3{}^b$).

(E)-4-(quinolin-6-yl)but-3-en-2-one (9q). Following the general procedure, 1-(triphenylphosphoranylidene)-2-propanone (563 mg, 1.77 mmol) and quinoline-6-carbaldehyde (250 mg, 1.59 mmol) were reacted for 6 h. Purification of the residue by flash column chromatography (hexane/AcOEt, 50:50) gave 1.70 g of an unsolvable mixture of the starting ylide and the desired product **9q**. The mixture was used in the next step without further purification.

(E)-4-(2,3-Dihydrobenzofuran-5-yl)but-3-en-2-one (9r). According to the general procedure of the aldol condensation described in the experimental section and similarly to naphthyl and biphenyl methylketones, 2,3-dihydrobenzofuran-5-carbaldehyde (1 g, 6.73 mmol), acetone (4.98 mL, 67.3 mmol) and NaOH 10% aq (27 mL, 67.3 mmol) were

reacted. After working up, crude was purified by flash column chromatography (hexane/AcOEt, 80:20) to provide 811 mg (64%) of a white solid identified as **(9r)**. M.p: 105-106 °C; ^{1}H NMR ($CDCl_3$, 400 MHz) δ (ppm): 7.36 (d, *J* = 16.2 Hz, 1H, C<u>H</u>=CHCO), 7.31 (s, 1H, Ar), 7.20 (dd, *J* = 8.2, 1.9 Hz, 1H, Ar), 6.68 (d, *J* = 8.6 Hz, 1H, Ar), 6.47 (d, *J* = 16.2 Hz, 1H, CH=C<u>H</u>CO), 4.51 (t, *J* = 8.7 Hz, 2H, OCH_2), 3.12 (t, *J* = 8.7 Hz, 2H, $OCH_2C\underline{H_2}$), 2.24 (s, 3H, CH_3).

(E)-4-(Dibenzo[b,d]furan-2-yl)but-3-en-2-one (9s). Following the general procedure, 1-(triphenylphosphoranylidene)-2-propanone (1.06 g, 3.31 mmol) and dibenzofuran-2-carboxaldehyde (500 mg, 2.55 mmol) were reacted to give **9s** (1.09 g, 90%) as a white solid. M.p.: 141-142 °C; ^{1}H NMR ($CDCl_3$, 400 MHz) δ (ppm): 7.93 (d, *J* = 1.9 Hz, 1H, Ar), 7.80 (d, *J* = 8.5 Hz, 1H, Ar), 7.57 - 7.32 (m, 5H, Ar, C<u>H</u>=CHCO), 7.25 (td, *J* = 7.5, 2.1 Hz, 1H, Ar), 6.64 (d, *J* = 16.2 Hz, 1H, CH=C<u>H</u>CO), 2.29 (s, 3H, CH_3).

1-bromo-4,4-dimethylpentan-2-one (10m). To a stirred solution of commercially available 4,4-dimethylpentan-2-one (1 g, 8.75 mmol) in MeOH (20 mL) was added dropwise Br_2 (0.45 mL, 8.75 mmol) at 0 °C. Then, the reaction mixture was allowed to warm to room temperature and stirred under reflux for 3 h. After quenching with water (20 mL), the aqueous phase was extracted with Et_2O (3 x 30 mL). The combined organics were dried (Na_2SO_4), filtered and carefully concentrated to give **10m** (1.69 g, >99%) as a colorless oil. ^{1}H NMR ($CDCl_3$, 400 MHz) δ (ppm): 3.86 (s, 2H, CH_2Br), 2.52 (s, CH_2-^{t}Bu), 1.03 (s, 9H, CH_3).

General procedure for the preparation of α-bromoketones through bromination with NBS (10p-s). To a solution of the corresponding methylketone (1 eq) in CH_3CN or THF, *p*-TsOH (1.3 eq) and NBS (1.3 eq) were successively added at -78 °C or at room temperature and the mixture was stirred for 6 h/overnight. After quenching with H_2O (30 mL) the reaction mixture was carefully concentrated under reduced pressure without heating. The aqueous crude was extracted with EtOAc (3 x 30 mL), dried (Na_2SO_4),

filtered and evaporated to dryness. The residue was purified by flash column chromatography or by CCTLC to give the desired α-bromoketones **10b**,**c** and **10p-s**.

(E)-1-bromo-5-phenylpent-3-en-2-one (10p). Following the general procedure, *p*-TsOH (890 mg, 4.68 mmol), NBS (833 mg, 4.68 mmol) and the methylketone mixture **(9pa** and **9pb)** (500 mg, 3.12 mmol) were reacted. After the work-up and purification **10p** (130 mg, 17%) was obtained as a colorless oil. ^{1}H NMR (CDCl$_3$, 400 MHz) δ (ppm): 7.37 - 7.31 (m, 2H, Ar), 7.29 - 7.23 (m, 1H, Ar), 7.20 - 7.16 (m, 2H, Ar), 7.12 (dt, *J* = 15.7, 6.8 Hz, 1H, CH=CHCO), 6.28 (dt, *J* = 15.8, 1.6 Hz, 1H, CH=CHCO), 3.98 (s, 2H, CH$_2$Br), 3.59 (dd, *J* = 7.0, 1.7 Hz, 2H, PhCH$_2$); ^{13}C NMR (CDCl$_3$, 75 MHz) δ (ppm): 191.1 (CO), 148.7 (CH$_2$CH=CH), 137.3 (C$_{Ar}$), 129.0 (CH$_{Ar}$), 127.4 (CH$_{Ar}$), 127.1 (CH$_2$CH=CH), 39.0 (Ar-CH$_2$), 32.7 (CH$_2$Br).

(E)-1-bromo-4-(quinolin-6-yl)but-3-en-2-one (10q). Following the general bromination procedure, **9q** (1.59 mmol), *p*-TsOH (454 mg, 2.39 mmol) and NBS (425 mg, 2.39 mmol) were reacted. To give after the work-up compound **10q** that was not isolated due to stability problems and it was used directly in the next step without further purification.

(E)-1-bromo-4-(2,3-dihydrobenzofuran-5-yl)but-3-en-2-one (10r). Following the general procedure, *p*-TsOH (380 mg, 2.00 mmol) and NBS (355 mg, 2.00 mmol) were successively added to a solution of methylketone **9r** (250 mg, 1.33 mmol) and allowed to react for 6 h. Work-up and purification gave **10r** (107 mg, 30%) as a white solid. M.p.: Decompose without melting; ^{1}H NMR (CDCl$_3$, 400 MHz) δ (ppm): 7.65 (d, *J* = 15.9 Hz, 1H, CH=CHCO), 7.46 (d, *J* = 2.0 Hz, 1H, Ar), 7.36 (dd, *J* = 8.3, 1.9 Hz, 1H, Ar), 6.80 (d, *J* = 8.2 Hz, 1H, Ar), 6.64 (d, *J* = 15.9 Hz, 1H, CH=CHCO), 4.64 (t, *J* = 8.7 Hz, 2H, OCH$_2$), 4.05 (s, 2H, CH$_2$Br), 3.24 (t, J = 8.7 Hz, 2H, OCH$_2$CH$_2$); ^{13}C NMR (CDCl$_3$, 75 MHz) δ (ppm): 191.1 (CO), 163.2 (OC$_{Ar}$), 145.8 (ArCH=CH), 130.7 (CH$_{Ar}$), 128.5 (C$_{Ar}$), 126.9 (C$_{Ar}$), 125.2

(ArCH=CH), 119.3 (CH_{Ar}), 110.0 (CH_{Ar}), 72.2 (OCH_2), 33.3 (CH_2Br), 29.3 (OCH_2CH_2); MS (ESI, positive mode) m/z: 289.0 $[M+Na]^+$, 267.0 $[M+H]^+$, both with a Br isotopic pattern.

(E)-1-bromo-4-(dibenzo[b,d]furan-2-yl)but-3-en-2-one (10s). Following the general procedure, methylketone **9s** (200 mg, 0.85 mmol), *p*-TsOH (161 mg, 0.85 mmol) and NBS (151 mg, 0.85 mmol) were reacted. Work-up and purification of the residue by CCTLC on the Chromatotron (hexane/EtOAc, 93:7) gave **10s** (75 mg, 28%) as a white solid. M.p.: 143-145 °C; ^{1}H NMR ($CDCl_3$, 400 MHz) δ (ppm): 8.17 (d, *J* = 1.8 Hz, 1H, Ar), 7.98 (dd, *J* = 7.7, 1.4 Hz, 1H, Ar), 7.87 (d, *J* = 16.0 Hz, 1H, CH=CHCO), 7.70 (dd, *J* = 8.5, 1.8 Hz, 1H, Ar), 7.62 - 7.57 (m, 2H, Ar), 7.51 (td, *J* = 8.0, 1.3 Hz, 1H, Ar), 7.39 (td, *J* = 7.6, 1.0 Hz, 1H, Ar), 7.02 (d, *J* = 16.0 Hz, 1H, CH=CHCO), 4.12 (s, 2H, CH_2Br); ^{13}C NMR ($CDCl_3$, 100 MHz) δ (ppm): 191.0 (CO), 158.0 (OC_{Ar}), 156.9 (OC_{Ar}), 145.7 (ArCH=CH), 143.3 (CH_{Ar}), 129.1 (C_{Ar}), 128.1 (CH_{Ar}), 128.0 (ArCH=CH), 125.1 (C_{Ar}), 123.6 (C_{Ar}), 123.4 (CH_{Ar}), 121.4 (CH_{Ar}), 121.0 (CH_{Ar}), 112.5 (CH_{Ar}), 112.1 (CH_{Ar}), 33.3 (CH_2Br); MS (ESI, positive mode) m/z: 329.0 $[M+Na]^+$ with a Br isotopic pattern.

<u>**Synthesis of intermediates and target compounds 12d-s**</u>

4-Azido-2-methoxybenzonitrile (5b). A solution of **4b**[1] (500 mg, 2.36 mmol) and NaN_3 (2.30 g, 35.4 mmol) in anhydrous DMSO (30 mL) in the presence of 4 Å molecular sieves, and under argon atmosphere, was heated at 100 °C for 72 h. After a similar work-up as described for **5b,** the residue was purified by flash column chromatography (CH_2Cl_2/MeOH, 100:2) to give **5b (**304 mg, 74%) as a white solid. M.p.: 118-120 °C; ^{1}H NMR ($CDCl_3$, 400 MHz) δ (ppm): 7.53 (d, J = 8.3 Hz, 1H, Ar), 6.69 (dd, J = 8.3, 2.0 Hz, 1H, Ar), 6.54 (d, J = 2.0 Hz, 1H, Ar), 3.92 (s, 3H, OCH_3); ^{13}C NMR ($CDCl_3$, 75 MHz) δ (ppm): 162.7 (OC_{Ar}), 146.7 (C_{Ar}), 135.1 (CH_{Ar}), 116.2 (CN), 111.4 (CH_{Ar}), 102.6 (CH_{Ar}), 98.3 (C_{Ar}), 56.4 (OCH_3); MS (ESI, positive mode) m/z: 197.0 $[M+Na]^+$, 175.0 $[M+H]^+$. NaN_3 may be toxic and explosive. Thus, for safety precautions a polycarbonate safety screen in a properly functioning fume hood was always used to perform this reaction.

General procedure for the synthesis of 1,2,3-triazoles by copper-catalyzed 1,3-dipolar azide-alkyne cycloaddition (CuAAC) (7a-g). A solution of the azide intermediates **5a,b** (1 eq) in EtOH was treated with the corresponding terminal alkynes **6a-g** (1.2 eq) and $CuSO_4 \cdot 5H_2O$ (0.1 eq). Sodium ascorbate (0.40 eq) and H_2O were subsequently added and the reaction mixture was stirred at room temperature overnight in the darkness. Then, it was evaporated to dryness and the residue was dissolved in CH_2Cl_2 (50 mL) and washed with H_2O (3 x 50 mL) dried (Na_2SO_4) filtered and evaporated under reduced pressure. The residue was purified by flash column chromatography (eluents are specified in each case).

Tert-butyl-(4-(1-(4-cyano-3-(2-(2-oxoimidazolidin-1-yl)ethoxy)phenyl)-1H-1,2,3-triazol-4-yl)butyl)carbamate (7a). Following the general procedure, azide **5a** (250 mg, 0.92 mmol) reacted with commercially available *tert*-butyl-5-hexynylcarbamate (**6a,** 248 mg, 1.19 mmol). After the work-up the residue was purified by flash column chromatography (CH_2Cl_2/MeOH, 100:2) to give **7a** (242 mg, 56%) as a colorless oil. ^{1}H

NMR ($CDCl_3$, 300 MHz) δ (ppm): 7.93 (s, 1H, Ar), 7.68 (dd, *J* = 8.4, 2.4 Hz, 1H, Ar), 7.57 (s, 1H, Ar), 7.39 (d, *J* = 7.7 Hz, 1H, Ar), 4.78 (br s, 1H, NHCON), 4.69 (br s, 1H, NHBoc), 4.35 - 4.31 (m, 2H, OCH_2), 3.75 (t, *J* = 7.1 Hz, 2H, $C\underline{H_2}CH_2NHCON$), 3.67 - 3.62 (m, 2H, $OCH_2C\underline{H_2}$), 3.45 (t, *J* = 7.4 Hz, 2H, $CH_2C\underline{H_2}NHCON$), 3.15 (q, *J* = 6.0 Hz, 2H, $C\underline{H_2}NHBoc$), 2.82 (t, *J* = 6.6 Hz, 2H, $TrizCH_2$), 1.76 (quin, *J* = 7.7 Hz, 2H, $TrizCH_2C\underline{H_2}$), 1.57 (quin, *J* = 7.5 Hz, 2H, $C\underline{H_2}CH_2NHBoc$), 1.42 (s, 9H, CH_3); ^{13}C NMR ($CDCl_3$, 75 MHz) δ (ppm): 162.8 (NHCON), 161.5 (OC_{Ar}), 156.2 (NHCOO), 149.3 (C_{Ar}), 141.6 (C_{Ar}), 135.1 (CH_{Ar}), 119.1 (CH_{Ar}), 115.7 (CN), 112.1 (CH_{Ar}), 104.1 (CH_{Ar}), 101.6 (C_{Ar}), 79.3 ($O\underline{C}(CH_3)_3$), 69.2 (OCH_2), 47.4 ($\underline{C}H_2CH_2NHCON$), 43.0 ($OCH_2\underline{C}H_2$), 40.3 ($CH_2NHBoc$), 38.6 ($CH_2\underline{C}H_2NHCON$), 29.5 ($\underline{C}H_2CH_2NHBoc$), 28.5 ($CH_3$), 26.3 ($TrizCH_2\underline{C}H_2$), 25.2 ($Triz\underline{C}H_2$); MS (ESI, positive mode) m/z: 492.3 $[M+Na]^+$, 470.3 $[M+H]^+$.

Benzyl-(4-(1-(4-cyano-3-methoxyphenyl)-1H-1,2,3-triazol-4-yl)butyl)carbamate (7c). Following the general procedure azide **5b** (590 mg, 2.71 mmol) was reacted with benzyl 5-hexynylcarbamate (858 mg, 3.52 mmol), $CuSO_4 \cdot 5H_2O$ (68 mg, 0.27 mmol) and sodium ascorbate (215 mg, 1.08 mmol). After the work-up the residue was purified by flash column chromatography (First: hexane/EtOAc, 80:20; Second: CH_2Cl_2/MeOH, 100:2) to give **7c** (963 mg, 88%) as a yellow oil. ^1H NMR ($CDCl_3$, 400 MHz) δ (ppm): 7.79 (s, 1H, Ar), 7.60 (d, *J* = 8.3 Hz, 1H, Ar), 7.49 (d, *J* = 1.9 Hz, 1H, Ar), 7.31 - 7.14 (m, 6H, Ar), 5.02 (s, 2H, $NHCOOC\underline{H_2}$), 4.90 (t, *J* = 6.2 Hz, 1H, NHCbz), 3.95 (s, 3H, OCH_3), 3.17 (q, *J* = 6.7 Hz, 2H, $C\underline{H_2}NHCbz$), 2.76 (t, *J* = 7.5 Hz, 2H, $TrizCH_2$), 1.71 (quin, *J* = 7.5 Hz, 2H, $TrizCH_2C\underline{H_2}$), 1.55 (quin, *J* = 7.2 Hz, 2H, $C\underline{H_2}CH_2NHCbz$); ^{13}C NMR ($CDCl_3$, 100 MHz) δ (ppm): 162.5 (OC_{Ar}), 156.6 (NHCOO), 149.2 (C_{Ar}), 141.5 (C_{Ar}), 136.6 (C_{Ar}), 135.1 (CH_{Ar}), 128.6 (CH_{Ar}), 128.2 (CH_{Ar}), 128.1 (CH_{Ar}), 119.1 (CH_{Ar}), 115.6 (CN), 111.5 (CH_{Ar}), 103.6 (CH_{Ar}), 101.6 (C_{Ar}), 66.7 ($NHCOO\underline{C}H_2$), 56.7 (OCH_3), 40.7 (CH_2NHCbz), 29.5 ($\underline{C}H_2CH_2NHCbz$), 26.3 ($TrizCH_2\underline{C}H_2$), 25.4 ($Triz\underline{C}H_2$); HRMS (ES, positive mode) m/z: calculated for $C_{22}H_{23}N_5O_3$ 405.1801; Found 405.1794 (1.8 ppm).

Benzyl (3-(1-(4-cyano-3-((1-(2-oxoimidazolidin-1-yl)ethoxy)phenyl)-1H-1,2,3-triazol-4-yl)propyl)carbamate (7d). According to the general procedure, azide **5a** (600 mg, 2.20 mmol), benzyl 4-pentynylcarbamate (622 mg, 2.86 mmol), $CuSO_4{\cdot}5H_2O$ (55 mg, 0.22 mmol) and sodium ascorbate (175 mg, 0.88 mmol) were reacted. After the work-up the residue was purified by flash column chromatography (CH_2Cl_2/MeOH, 100:2) to give **7d** (779 mg, 72%) as a colorless oil. ^{1}H NMR ($CDCl_3$, 400 MHz) δ (ppm): 8.01 (s, 1H, Ar), 7.61 (d, *J* = 8.3 Hz, 1H, Ar), 7.55 (s, 1H, Ar), 7.38 (d, *J* = 8.4 Hz, 1H, Ar), 7.32 - 7.18 (m, 5H, Ar), 5.48 (br s, 1H, NHCbz), 5.02 (s, 2H, NHCOOC$\underline{H_2}$), 4.70 (br s, 1H, NHCON), 4.26 (t, *J* = 5.7 Hz, 2H, OCH_2), 3.63 (t, *J* = 8.0 Hz, 2H, C$\underline{H_2}$$CH_2$NHCON), 3.55 (t, *J* = 5.7 Hz, 2H, OCH_2C$\underline{H_2}$), 3.25 (t, *J* = 8.0 Hz, 2H, CH_2C$\underline{H_2}$NHCON), 3.16 (q, *J* = 6.6 Hz, 2H, C$\underline{H_2}$NHCbz), 2.76 (t, *J* = 7.3 Hz, 2H, TrizCH_2), 1.85 - 1.79 (m, 2H, TrizCH_2C$\underline{H_2}$); ^{13}C NMR ($CDCl_3$, 75 MHz) δ (ppm): 162.9 (NHCON), 161.4 (OC_{Ar}), 156.7 (NHCOO), 148.4 (C_{Ar}), 141.5 (C_{Ar}), 136.5 (CH_{Ar}), 135.1 (CH_{Ar}), 128.7 (CH_{Ar}), 128.3 (CH_{Ar}), 119.7 (CH_{Ar}), 115.7 (CN), 112.0 (CH_{Ar}), 104.1 (CH_{Ar}), 101.4 (C_{Ar}), 68.6 (OCH_2), 66.9 (NHCOO$\underline{C}H_2$), 47.2 ($\underline{C}H_2CH_2$NHCON), 42.6 ($OCH_2\underline{C}H_2$), 39.8 (CH_2NHCbz), 38.5 ($CH_2\underline{C}H_2$NHCON), 29.0 (Triz$\underline{C}H_2CH_2$), 22.3 (Triz$\underline{C}H_2$); HRMS (ES, positive mode) m/z: calculated for $C_{25}H_{27}N_7O_4$ 489.2124; Found 489.2126 (0.21 ppm).

Methyl 4-(1-(4-cyano-3-(2-(2-oxoimidazolidin-1-yl)ethoxy)phenyl)-1H-1,2,3-triazol-4-yl)butanoate (7e). Following the general procedure, azide **5a** (472 mg, 1.73 mmol) was reacted with methyl 4-butynylcarboxylate (293 mg, 2.25 mmol), $CuSO_4{\cdot}5H_2O$ (43 mg, 0.18 mmol) and sodium ascorbate (137 mg, 0.69 mmol. Work-up and purification of the residue by flash column chromatography (CH_2Cl_2/MeOH, 100:2) gave **7e** (451 mg, 65%) as a white solid. M.p.: 122.4 - 123.6 °C; ^{1}H NMR ($CDCl_3$, 400 MHz) δ (ppm): 7.90 (s, 1H, Ar), 7.67 (d, *J* = 8.4 Hz, 1H, Ar), 7.54 (d, *J* = 2.0 Hz, 1H, Ar), 7.36 (dd, *J* = 8.4, 2.9 Hz, 1H, Ar), 4.98 (br s, 1H, NHCON), 4.32 (t, *J* = 5.1 Hz, 2H, OCH_2), 3.75 (dd, *J* = 9.0, 6.8 Hz, 2H, C$\underline{H_2}$$CH_2$NHCON), 3.44 (dd, *J* = 9.0, 6.9 Hz, 2H, CH_2C$\underline{H_2}$NHCON), 2.83 (t, *J* = 7.5 Hz, 2H, C$\underline{H_2}$COOMe), 2.41 (t, *J* = 7.3 Hz, 2H, TrizCH_2), 2.05 (quint, *J* = 7.5 Hz, 2H, TrizCH_2C$\underline{H_2}$);

^{13}C NMR ($CDCl_3$, 75 MHz) δ (ppm): 173.7 (COO), 162.8 (NHCON), 161.5 (OC_{Ar}), 148.7 (C_{Ar}), 141.5 (C_{Ar}), 135.0 (CH_{Ar}), 119.2 (CH_{Ar}), 115.6 (CN), 112.0 (CH_{Ar}), 104.1 (CH_{Ar}), 101.6 (C_{Ar}), 69.3 (OCH_2), 51.7 (Me), 47.4 ($\underline{C}H_2CH_2NHCON$), 43.0 ($OCH_2\underline{C}H_2$), 38.6 ($CH_2\underline{C}H_2NHCON$), 33.3 ($CH_2COOMe$), 24.9 ($Triz\underline{C}H_2CH_2$), 24.4 ($Triz\underline{C}H_2$); HRMS (ES, positive mode) m/z: calculated for $C_{19}H_{22}N_6O_4$ 398.1702; Found 398.1702 (-0.16 ppm).

4-(4-Butyl-1H-1,2,3-triazol-1-yl)-2-(2-(2-oxoimidazolidin-1-yl)ethoxy)benzonitrile (7f). According to the general procedure, azide **5a** (269 mg, 0.99 mmol), 1-hexyne (109 mg, 1.28 mmol), $CuSO_4 \cdot 5H_2O$ (25 mg, 0.10 mmol) and sodium ascorbate (78 mg, 0.40 mmol) were reacted. After work-up and purification (CH_2Cl_2/MeOH, 100:1), **7f** (277 mg, 79%) was obtained as a white solid. M.p.: 187-190 °C; ^1H NMR ($CDCl_3$, 400 MHz) δ (ppm): 7.80 (s, 1H, Ar), 7.69 (d, *J* = 8.3 Hz, 1H, Ar), 7.53 (d, *J* = 1.9 Hz, 1H, Ar), 7.36 (dd, *J* = 8.4, 1.9 Hz, 1H, Ar), 4.53 (br s, 1H, NHCON), 4.34 (t, *J* = 5.0 Hz, 2H, OCH_2), 3.79 (t, *J* = 7.6 Hz, 2H, $C\underline{H_2}CH_2NHCON$), 3.68 (t, *J* = 4.9 Hz, 2H, $OCH_2C\underline{H_2}$), 3.46 (t, *J* = 7.7 Hz, 2H, $CH_2C\underline{H_2}NHCON$), 2.80 (t, *J* = 7.7 Hz, 2H, $TrizCH_2$), 1.73 (quin, *J* = 7.5 Hz, 2H, $TrizCH_2C\underline{H_2}$), 1.52 (sex, *J* = 7.4 Hz, 2H, $C\underline{H_2}CH_3$), 0.96 (t, *J* = 7.3 Hz, 3H, CH_3); ^{13}C NMR ($CDCl_3$, 75 MHz) δ (ppm): 162.7 (NHCON), 161.6 (OC_{Ar}), 150.1 (C_{Ar}), 141.6 (C_{Ar}), 135.1 (CH_{Ar}), 118.7 (CH_{Ar}), 115.7 (CN), 112.0 (CH_{Ar}), 104.1 (CH_{Ar}), 101.6 (C_{Ar}), 69.5 (OCH_2), 47.5 ($\underline{C}H_2CH_2NHCON$), 43.1 ($OCH_2\underline{C}H_2$), 38.6 ($CH_2\underline{C}H_2NHCON$), 31.4 ($TrizCH_2\underline{C}H_2$), 25.4 ($Triz\underline{C}H_2$), 22.4 ($\underline{C}H_2CH_3$), 13.9 ($CH_3$); MS (ESI, positive mode) m/z: 377.2 $[M+Na]^+$, 355.2 $[M+H]^+$.

4-(4-(4-Hydroxybutyl)-1H-1,2,3-triazol-1-yl)-2-(2-(2-oxoimidazolidin-1-yl)ethoxy)benzonitrile (7g). Following the general CuAAC procedure, azide **5a** (250 mg, 0.92 mmol), commercially available 5-hexyn-1-ol (122 mg, 1.19 mmol), $CuSO_4 \cdot 5H_2O$ (23 mg, 0.09 mmol) in EtOH (20 mL) and sodium ascorbate (73 mg, 0.40 mmol) were reacted. Purification of the final residue by flash column chromatography (CH_2Cl_2/MeOH, 100:7) provided **7g** (204 mg, 55%) as a white solid. M.p.: 138-140 °C; ^1H NMR (CD_3OD, 400

MHz) δ (ppm): 8.48 (s, 1H, Ar), 7.81 (d, *J* = 8.4 Hz, 1H, Ar), 7.70 (d, *J* = 1.9 Hz, 1H, Ar), 7.61 (dd, *J* = 8.4, 1.9 Hz, 1H, Ar), 4.39 (t, *J* = 5.2 Hz, 2H, OCH_2), 3.76 (dd, *J* = 9.0, 7.2 Hz, 2H, $C\underline{H_2}CH_2NHCON$), 3.64 (t, *J* = 5.1 Hz, 2H, $OCH_2C\underline{H_2}$), 3.61 (t, *J* = 6.4 Hz, 2H, $C\underline{H_2}OH$), 3.43 (dd, *J* = 9.0, 7.2 Hz, 2H, $CH_2C\underline{H_2}NHCON$), 2.82 (t, *J* = 7.6 Hz, 2H, $TrizCH_2$), 1.82 (quin, *J* = 6.5 Hz, 2H, $TrizCH_2C\underline{H_2}$), 1.63 (quin, *J* = 6.5 Hz, 2H, $C\underline{H_2}CH_2OH$); ^{13}C NMR (CD_3OD, 75 MHz) δ (ppm): 165.1 (NHCON), 162.9 (OC_{Ar}), 150.5 (C_{Ar}), 142.9 (C_{Ar}), 136.3 (CH_{Ar}), 121.5 (CH_{Ar}), 116.6 (CN), 113.5 (CH_{Ar}), 105.3 (CH_{Ar}), 102.5 (C_{Ar}), 70.1 (OCH_2), 62.5 (CH_2OH), 50.5 ($\underline{C}H_2CH_2NHCON$), 43.9 ($OCH_2\underline{C}H_2$), 39.4 ($CH_2\underline{C}H_2NHCON$), 33.0 ($\underline{C}H_2CH_2OH$), 26.7 ($TrizCH_2\underline{C}H_2$), 26.1 ($Triz\underline{C}H_2$); MS (ESI, positive mode) m/z: 763.5 $[2M+Na]^+$, 393.2 $[M+Na]^+$, 371.2 $[M+H]^+$.

General procedure for the synthesis of thioamides (8a-g). A solution of the corresponding benzonitriles **7a-g** (1 eq) in DMF was treated with an excess of 20% aq. $(NH_4)_2S$ (70 eq). A change of yellow to dark blue color was instantly observed. The reaction mixture was heated at 80 °C for 4 h and then allowed to cool to room temperature. CH_2Cl_2 (50 mL) was added and the resulting solution was successively washed with HCl 0.1 N (2 x 50 mL), H_2O (1 x 50 mL) and brine (1 x 50 mL). The organic layers were dried (Na_2SO_4), filtered and evaporated to dryness. The crude was purified by flash column chromatography (eluents are specified for each compound).

Tert-butyl-(4-(1-(4-carbamothioyl-3-(2-(2-oxoimidazolidin-1-yl)ethoxy)phenyl)-1H-1,2,3-triazol-4-yl)butyl)carbamate (8a). According to the general procedure, a solution of benzonitrile **7a** in DMF (10 mL) and 20% aq solution of $(NH_4)_2S$ (1 mL, 13.8 mmol) were reacted. Work-up and purification of the crude by flash column chromatography (CH_2Cl_2/MeOH, 100:5) gave 64 mg (67%) of **8a** as a yellow oil. IR (KBr), ν (cm^{-1}): 3351 (NH st), 1682 (C=O st); ^1H NMR (DMSO-d_6, 400 MHz) δ (ppm): 10.10 (br s, 1H, $SCNH_2$), 9.43 (br s, 1H, $SCNH_2$), 8.68 (s, 1H, Ar), 7.88 (dd, *J* = 8.4, 1.1 Hz, 1H, Ar), 7.56 (d, *J* = 1.6 Hz, 1H, Ar), 7.51 (dd, *J* = 8.4, 1.7 Hz, 1H, Ar), 6.82 (br s, 1H, NHCON), 6.38 (br s, 1H,

NHBoc), 4.25 (t, *J* = 5.2 Hz, 2H, OCH_2), 3.55 - 3.45 (m, 4H, $C\underline{H_2}CH_2NHCON$, $OCH_2C\underline{H_2}$), 3.22 (t, *J* = 7.8 Hz, 2H, $CH_2C\underline{H_2}NHCON$), 2.98 - 2.93 (m, 2H, $C\underline{H_2}NHBoc$), 2.71 (t, *J* = 7.5 Hz, 2H, $TrizCH_2$), 1.70 - 1.60 (m, 2H, $TrizCH_2C\underline{H_2}$), 1.51 - 1.41 (m, 2H, $C\underline{H_2}CH_2NHBoc$), 1.36 (s, 9H, CH_3); ^{13}C NMR (DMSO-d_6, 75 MHz) δ (ppm): 197.5 ($SCNH_2$), 162.2 (NHCON), 155.6 (NHCOO), 154.3 (OC_{Ar}), 148.1 (C_{Ar}), 138.5 (C_{Ar}), 132.5 (CH_{Ar}), 129.8 (C_{Ar}), 120.3 (CH_{Ar}), 111.1 (CH_{Ar}), 103.9 (CH_{Ar}), 77.3 ($O\underline{C}(CH_3)_3$), 67.7 (OCH_2), 45.6 ($\underline{C}H_2CH_2NHCON$), 42.6 ($OCH_2\underline{C}H_2$), 37.6 ($CH_2\underline{C}H_2NHCON$), 29.0 ($\underline{C}H_2CH_2NHBoc$), 28.3 ($CH_3$), 26.0 ($TrizCH_2\underline{C}H_2$), 24.7 ($Triz\underline{C}H_2$); MS (ESI, positive mode) m/z: 526.3 $[M+Na]^+$, 504.3 $[M+H]^+$.

Benzyl-(4-(1-(4-carbamothioyl-3-methoxyphenyl)-1H-1,2,3-triazol-4-yl)butyl)carbamate (8c). The general procedure was followed with benzonitrile **7c** (1.07 g, 2.64 mmol). After the work-up, **8c (**1.15 g, >99%) was obtained as a yellow oil that was used in the next step without further purification. ^1H NMR (DMSO-d_6, 400 MHz) δ (ppm): 10.08 (br s, 1H, $SCNH_2$), 9.42 (br s, 1H, $SCNH_2$), 8.67 (s, 1H, Ar), 7.87 (d, *J* = 8.4 Hz, 1H, Ar), 7.54 (d, *J* = 2.0 Hz, 1H, Ar), 7.49 (dd, 1H, *J* = 8.4, 2.0 Hz, Ar), 7.40 - 7.17 (m, 6H, Ar, NHCbz), 5.01 (s, 2H, $NHCOOC\underline{H_2}$), 3.93 (s, 3H, OCH_3), 3.06 (q, *J* = 6.6 Hz, 2H, $C\underline{H_2}NHCbz$), 2.72 (t, *J* = 7.5 Hz, 2H, $TrizCH_2$), 1.68 (quin, *J* = 7.5 Hz, 2H, $TrizCH_2C\underline{H_2}$), 1.50 (quin, *J* = 7.1 Hz, 2H, $C\underline{H_2}CH_2NHCbz$); ^{13}C NMR (DMSO-d_6, 75 MHz) δ (ppm): 197.5 ($SCNH_2$), 156.1 (OC_{Ar}), 155.2 (NHCOO), 148.1 (C_{Ar}), 138.5 (C_{Ar}), 137.3 (C_{Ar}), 132.4 (CH_{Ar}), 129.8 (CH_{Ar}), 128.3 (CH_{Ar}), 127.7 (CH_{Ar}), 127.6 (CH_{Ar}), 120.4 (CH_{Ar}), 110.9 (CH_{Ar}), 103.2 (CH_{Ar}), 65.1 ($NHCOO\underline{C}H_2$), 56.3 (OCH_3), 40.0 (CH_2NHCbz), 28.9 ($\underline{C}H_2CH_2NHCbz$), 26.0 ($TrizCH_2\underline{C}H_2$), 24.7 ($Triz\underline{C}H_2$); HRMS (ES, positive mode) m/z: calculated for $C_{22}H_{25}N_5O_3S$ 439.1678; Found 439.1674 (-0.96 ppm).

Benzyl-(3-(1-(4-carbamothioyl-3-((1-(2-oxoimidazolidin-1-yl)ethoxy)phenyl)-1H-1,2,3-triazol-4-yl)propyl)carbamate (8d). The general procedure was followed with **7d** (779 mg, 1.59 mmol) and 20% aq $(NH_4)_2S$ (7.6 mL, 0.11 mol). Purification of the final residue by flash column chromatography (CH_2Cl_2/MeOH, 100:5) gave **8d** (655 mg, 79%) as a

yellow oil. ^{1}H NMR ($CDCl_3$, 400 MHz) δ (ppm): 9.69 (br s, 1H, $SCNH_2$), 9.49 (br s, 1H, $SCNH_2$), 8.69 (d, *J* = 8.7 Hz, 1H, Ar), 7.84 (s, 1H, Ar), 7.42 (s, 1H, Ar), 7.38 - 7.13 (m, 5H, Ar), 7.09 (dd, *J* = 8.7, 2.0 Hz, 1H, Ar), 6.03 (br s, 1H, NHCON), 5.18 (t, *J* = 6.2 Hz, 1H, NHCbz), 5.02 (s, 2H, $NHCOOC\underline{H_2}$), 4.13 (t, *J* = 4.3 Hz, 2H, OCH_2), 3.63 (t, *J* = 4.5 Hz, 2H, $OCH_2C\underline{H_2}$), 3.49 (dd, *J* = 9.4, 6.4 Hz, 2H, $C\underline{H_2}CH_2NHCON$), 3.38 (dd, *J* = 9.4, 6.5 Hz, 2H, $CH_2C\underline{H_2}NHCON$), 3.19 (q, *J* = 6.7 Hz, 2H, $C\underline{H_2}NHCbz$), 2.74 (t, *J* = 7.4 Hz, 2H, $TrizCH_2$), 1.93 - 1.80 (m, 2H, $TrizCH_2C\underline{H_2}$); ^{13}C NMR ($CDCl_3$, 75 MHz) δ (ppm): 196.9 ($SCNH_2$), 163.3 (NHCON), 156.8 (NHCOO), 156.2 (OC_{Ar}), 148.4 (C_{Ar}), 140.4 (C_{Ar}), 138.1 (CH_{Ar}), 137.0 (CH_{Ar}), 128.7 (CH_{Ar}), 128.2 (CH_{Ar}), 125.6 (C_{Ar}), 119.4 (CH_{Ar}), 111.6 (CH_{Ar}), 104.3 (CH_{Ar}), 66.9 (OCH_2), 66.8 ($NHCOO\underline{C}H_2$), 45.6 ($\underline{C}H_2CH_2NHCON$), 43.3 ($OCH_2\underline{C}H_2$), 40.5 ($CH_2NHCbz$), 38.4 ($CH_2\underline{C}H_2NHCON$), 29.5 ($\underline{C}H_2CH_2NHCbz$), 22.8 ($Triz\underline{C}H_2$); HRMS (ES, positive mode) m/z: calculated for $C_{25}H_{29}N_7O_4S$ 523.2002; Found 523.2007 (0.96 ppm).

Methyl-4-(1-(4-carbamothioyl-3-(2-(2-oxoimidazolidin-1-yl)ethoxy)phenyl)-1H-1,2,3-triazol-4-yl)butanoate (8e). Following the general procedure benzonitrile **7e** (416 mg, 1.04 mmol) and 20% aq $(NH_4)_2S$ (4.44 mL, 65.3 mmol) were reacted, to give, after work-up, **8e** (434 mg, quantitative) as a yellow solid. M.p.: 156.5 - 159.5 °C; ^{1}H NMR ($CDCl_3$, 400 MHz) δ (ppm): 9.55 (br s, 1H, $SCNH_2$), 9.46 (br s, 1H, $SCNH_2$), 8.83 (d, *J* = 8.7 Hz, 1H, Ar), 7.84 (s, 1H, Ar), 7.58 (d, *J* = 2.1 Hz, 1H, Ar), 7.18 (dd, *J* = 8.7, 2.0 Hz, 1H, Ar), 5.76 (br s, 1H, NHCON), 4.27 (t, *J* = 4.5 Hz, 2H, OCH_2), 3.75 (t, *J* = 4.5 Hz, 2H, $OCH_2C\underline{H_2}$), 3.58 (dd, *J* = 8.8, 5.8 Hz, 2H, $C\underline{H_2}CH_2NHCON$), 3.51 (dd, *J* = 9.2, 6.1 Hz, 2H, $CH_2C\underline{H_2}NHCON$), 2.85 (t, *J* = 7.6 Hz, 2H, $C\underline{H_2}COOMe$), 2.43 (t, *J* = 7.4 Hz, 2H, $TrizCH_2$), 2.08 (quint, *J* = 7.5 Hz, 2H, $TrizCH_2C\underline{H_2}$); ^{13}C NMR ($CDCl_3$, 75 MHz) δ (ppm): 196.1 ($SCNH_2$) 173.8 (COO), 163.5 (NHCON), 156.4 (OC_{Ar}), 148.5 (C_{Ar}), 140.2 (C_{Ar}), 138.5 (C_{Ar}), 125.1 (CH_{Ar}), 119.2 (CH_{Ar}), 111.2 (CH_{Ar}), 103.9 (CH_{Ar}), 66.8 (OCH_2), 51.8 (Me), 45.4 ($\underline{C}H_2CH_2NHCON$), 43.3 ($OCH_2\underline{C}H_2$), 38.3 ($CH_2\underline{C}H_2NHCON$), 33.4 ($\underline{C}H_2COOMe$), 25.0 ($Triz\underline{C}H_2CH_2$), 24.6 ($Triz\underline{C}H_2$); HRMS (ES, positive mode) m/z: calculated for $C_{19}H_{24}N_6O_4S$ 432.1580; Found 432.1559 (-4.82 ppm).

4-(4-Butyl-1H-1,2,3-triazol-1-yl)-2-(2-(2-oxoimidazolidin-1-yl)ethoxy)benzothioamide (8f). According to the general thionation procedure, benzonitrile **7f** (550 mg, 1.55 mmol) and 20% aq $(NH_4)_2S$ (7.4 mL, 109 mmol) were reacted. Work-up and purification by flash column chromatography (CH_2Cl_2/MeOH, 100:2) gave **8f** (505 mg, 84%) as a yellow solid. M.p.: 145-148 °C; ^{1}H NMR ($CDCl_3$, 400 MHz) δ (ppm): 9.74 (br s, 1H, $SCNH_2$), 9.59 (br s, 1H, $SCNH_2$), 8.81 (d, *J* = 8.7 Hz, 1H, Ar), 7.79 (s, 1H, Ar), 7.57 (d, *J* = 2.0 Hz, 1H, Ar), 7.16 (dd, *J* = 8.7, 2.0 Hz, 1H, Ar), 6.05 (br s, 1H, NHCON), 4.25 (t, *J* = 4.4 Hz, 2H, OCH_2), 3.75 (t, *J* = 4.5 Hz, 2H, $OCH_2C\underline{H_2}$), 3.58 (dd, *J* = 9.8 , 6.7 Hz, 2H, $C\underline{H_2}CH_2NHCON$), 3.50 (dd, *J* = 9.0, 6.0 Hz, 2H, $CH_2C\underline{H_2}NHCON$), 2.79 (t, *J* = 7.7 Hz, 2H, $TrizCH_2$), 1.71 (quin, *J* = 7.7 Hz, 2H, $TrizCH_2C\underline{H_2}$), 1.42 (sex, *J* = 7.4 Hz, 2H, $C\underline{H_2}CH_3$), 0.95 (t, *J* = 7.4 Hz, 3H, CH_3); ^{13}C NMR ($CDCl_3$, 100 MHz) δ (ppm): 195.9 ($SCNH_2$), 163.6 (NHCON), 156.4 (OC_{Ar}), 149.8 (C_{Ar}), 140.3 (C_{Ar}), 138.4 (CH_{Ar}), 124.9 (C_{Ar}), 118.8 (CH_{Ar}), 111.0 (CH_{Ar}), 103.8 (CH_{Ar}), 66.8 (OCH_2), 45.4 ($\underline{C}H_2CH_2NHCON$), 43.3 ($OCH_2\underline{C}H_2$), 38.3 ($CH_2\underline{C}H_2NHCON$), 31.5 ($TrizCH_2\underline{C}H_2$), 25.4 ($Triz\underline{C}H_2$), 22.4 ($\underline{C}H_2CH_3$), 13.9 ($CH_3$); MS (ESI, positive mode) m/z: 389.2 $[M+H]^+$.

4-(4-(4-Hydroxybutyl)-1H-1,2,3-triazol-1-yl)-2-(2-(2-oxoimidazolidin-1-yl)ethoxy) benzothioamide (8g). According to the general procedure, **7g** (375 mg, 1.01 mmol) and 20% aq $(NH_4)_2S$ (4.8 mL, 70.9 mmol) were reacted. After the work-up **8g (**408 mg, >99%) was obtained as a yellow solid, which was used in the next step without further purification. M.p.: Decompose without melting; ^{1}H NMR (CD_3OD, 400 MHz) δ (ppm): 8.47 (s, 1H, Ar), 8.17 (d, *J* = 8.5 Hz, 1H, Ar), 7.60 (d, *J* = 2.0 Hz, 1H, Ar), 7.49 (dd, *J* = 8.5, 2.0 Hz, 1H, Ar), 4.34 (t, *J* = 5.0 Hz, 2H, OCH_2), 3.70 - 3.58 (m, 6H, $C\underline{H_2}OH$, $OCH_2C\underline{H_2}$, $C\underline{H_2}CH_2NHCON$), 3.41 (dd, *J* = 9.3, 6.9 Hz, 2H, $CH_2C\underline{H_2}NHCON$), 2.86 (t, *J* = 7.6 Hz, 2H, $TrizCH_2$), 1.87 - 1.77 (m, 2H, $TrizCH_2C\underline{H_2}$), 1.68 - 1.59 (m, 2H, $C\underline{H_2}CH_2OH$); ^{13}C NMR (CD_3OD, 75 MHz) δ (ppm): 200.5 ($SCNH_2$), 165.6 (NHCON), 156.9 (OC_{Ar}), 150.6 (C_{Ar}), 141.1 (C_{Ar}), 135.5 (CH_{Ar}), 130.9 (C_{Ar}), 121.9 (CH_{Ar}), 113.2 (CH_{Ar}), 105.9 (CH_{Ar}), 69.2

(OCH_2), 63.0 (CH_2OH), 47.6 ($\underline{C}H_2CH_2NHCON$), 44.4 ($OCH_2\underline{C}H_2$), 39.7 ($CH_2\underline{C}H_2NHCON$), 33.4 ($\underline{C}H_2CH_2OH$), 27.2 ($TrizCH_2\underline{C}H_2$), 26.5 ($Triz\underline{C}H_2$); MS (ESI, positive mode) m/z: 831.3 $[2M+Na]^+$, 427.0 $[M+Na]^+$, 405.2 $[M+H]^+$.

General procedure for the synthesis of thiazoles by Hantzsch cyclization (11f-s and 12d-f). A solution of the thioamides **8** (1 eq) in ^{i}PrOH (15 mL) was treated with the appropriated α-bromomethylketone **10c**, **10m,f-s** (1 eq). The reaction mixture was stirred at 70 °C for 3 - 6 h in a pressure tube and then, it was allowed to cool to room temperature and concentrated to dryness under reduced pressure. The residue was purified by flash column chromatography or by CCTLC on the Chromatotron (eluents are specified in each case).

1-(2-(5-(4-butyl-1H-1,2,3-triazol-1-yl)-2-(4-(2-([1,1'-biphenyl]-4-yl)vinyl)thiazol-2-yl)phenoxy)ethyl)imidazolidin-2-one (11f). Following the general Hantzsch procedure, the thioamide **8f** (140 mg, 0.36 mmol) and α-bromoketone **10c** (108 mg, 0.36 mmol) were reacted for 4 h. The residue was purified by flash column chromatography (CH_2Cl_2/MeOH, 98:2) to yield **11f (**110 mg, 65%) as a colorless oil. ^1H NMR ($CDCl_3$, 400 MHz) δ (ppm): 8.59 (d, J = 8.5 Hz, 1H, Ar), 7.74 (s, 1H, Ar), 7.64 - 7.47 (m, 8H, Ar, $ThiazCH{=}C\underline{H}$), 7.35 (t, J = 7.5 Hz, 2H, Ar), 7.34 - 7.17 (m, 3H, Ar), 7.13 (d, J = 15.9 Hz, 1H, $ThiazC\underline{H}{=}CH$), 4.60 (br s, 1H, NHCON), 4.38 (t, J = 5.4 Hz, 2H, OCH_2), 3.75 (t, J = 5.4 Hz, 2H, $OCH_2C\underline{H_2}$), 3.54 (dd, J = 9.0, 6.6 Hz, 2H, $C\underline{H_2}CH_2NHCON$), 3.34 (dd, J = 9.1, 6.5 Hz, 2H, $CH_2C\underline{H_2}NHCON$), 2.74 (t, J = 7.7 Hz, 2H, $TrizCH_2$), 1.66 (quin, J = 7.6 Hz, 2H, $TrizCH_2C\underline{H_2}$), 1.38 (sex, J = 7.4 Hz, 2H, $C\underline{H_2}CH_3$), 0.90 (t, J = 7.4 Hz, 3H, CH_3); ^{13}C NMR ($CDCl_3$, 100 MHz) δ (ppm): 162.7 (NHCON), 161.0 (OC_{Ar}), 156.3 (C_{Ar}), 153.5 (C_{Ar}), 149.6 (C_{Ar}), 140.7 (C_{Ar}), 140.7 (C_{Ar}), 138.7 (C_{Ar}), 136.3 (C_{Ar}), 131.0 (CH_{Ar}), 130.3 (CH_{Ar}), 128.9 (CH_{Ar}), 127.5 (CH_{Ar}), 127.3 (CH_{Ar}), 127.2 (CH_{Ar}), 127.0 (CH_{Ar}), 122.4 (C_{Ar}), 121.6 (CH_{Ar}), 118.8 (CH_{Ar}), 117.1 (CH_{Ar}), 112.4 (CH_{Ar}), 104.6 (CH_{Ar}), 68.1 (OCH_2), 46.5 ($\underline{C}H_2CH_2NHCON$), 43.2 ($OCH_2\underline{C}H_2$), 38.5 ($CH_2\underline{C}H_2NHCON$), 31.6 ($TrizCH_2\underline{C}H_2$), 25.5

(Triz$\underline{C}H_2$), 22.5 ($\underline{C}H_2CH_3$), 14.0 (CH_3); HRMS (ES, positive mode) m/z: calculated for $C_{34}H_{34}N_6O_2S$ 590.2464; Found 590.2474 (1.66 ppm).

Methyl-4-(1-(3-(2-(2-oxoimidazolidin-1-yl)ethoxy)-4-(4-phenethylthiazol-2-yl)phenyl)-1H-1,2,3-triazol-4-yl)butanoate (11g). Following the Hantzsch procedure, thioamide **8e** (300 mg, 0.69 mmol) and the commercially available 1-bromo-4-phenylbutan-2-one **10a** (158 mg, 0.69 mmol) were reacted for 4 h. After the work-up, the residue was purified by CCTLC on the Chromatotron (CH_2Cl_2/MeOH, 100:3) to yield **11g** (305 mg, 76%) as a colorless oil. ^{1}H NMR ($CDCl_3$, 400 MHz) δ (ppm): 8.47 (d, J = 8.5 Hz, 1H, Ar), 7.80 (s, 1H, Ar), 7.52 (d, J = 2.0 Hz, 1H, Ar), 7.28 (dd, J = 8.5, 2.0 Hz, 1H, Ar), 7.22 - 7.03 (m, 5H, Ar), 6.87 (s, 1H, Ar), 4.81 (br s, 1H, NHCON), 4.36 (t, J = 5.5 Hz, 2H, OCH_2), 3.72 (t, J = 5.5 Hz, 2H, $OCH_2C\underline{H_2}$), 3.61 (s, 3H, Me), 3.53 (dd, J = 9.0, 6.6 Hz, 2H, $C\underline{H_2}CH_2$NHCON), 3.33 (dd, J = 9.1, 6.6 Hz, 2H, $CH_2C\underline{H_2}$NHCON), 3.14 - 2.93 (m, 4H, $C\underline{H_2}C\underline{H_2}$Ph), 2.79 (t, J = 7.5 Hz, 2H, TrizCH_2), 2.37 (t, J = 7.4 Hz, 2H, $C\underline{H_2}$COOMe), 2.02 (quin, J = 7.5 Hz, 2H, Triz$CH_2C\underline{H_2}$); ^{13}C NMR ($CDCl_3$, 100 MHz) δ (ppm): 173.8 (COO), 162.8 (NHCON), 160.2 (OC_{Ar}), 156.1 (C_{Ar}), 156.0 (C_{Ar}), 148.2 (C_{Ar}), 141.6 (C_{Ar}), 138.3 (C_{Ar}), 130.0 (CH_{Ar}), 128.6 (CH_{Ar}), 128.5 (CH_{Ar}), 128.4 (CH_{Ar}), 126.1 (CH_{Ar}), 122.8 (C_{Ar}), 119.2 (CH_{Ar}), 115.2 (CH_{Ar}), 112.4 (CH_{Ar}), 104.6 (CH_{Ar}), 68.1 (OCH_2), 51.7 (CH_3), 46.5 ($\underline{C}H_2CH_2$NHCON), 43.1 ($OCH_2\underline{C}H_2$), 38.4 ($CH_2\underline{C}H_2$NHCON), 35.6 (CH_2CH_2Ph), 33.4 (CH_2CH_2Ph), 33.4 ($\underline{C}H_2$COOMe), 25.0 (Triz$CH_2\underline{C}H_2$), 24.6 (Triz$\underline{C}H_2$); HRMS (ES, positive mode) m/z: calculated for $C_{29}H_{32}N_6O_4S$ 560.2206; Found 560.2200 (-1.11 ppm).

Benzyl-(3-(1-(3-(2-(2-oxoimidazolidin-1-yl)ethoxy)-4-(4-phenethylthiazol-2-yl)phenyl)-1H-1,2,3-triazol-4-yl)propyl)carbamate (11h). Following the general Hantzsch procedure, thioamide **8d** (325 mg, 0.62 mmol), the commercially available 1-bromo-4-phenylbutan-2-one **10a** (141 mg, 0.62 mmol) in ^{i}PrOH (30 mL), was stirred at 70 °C for 4 h. After the work-up, residue was purified by flash column chromatography (CH_2Cl_2/MeOH, 98:2) to yield **11h** (321 mg, 77%) as a colorless oil. ^{1}H NMR ($CDCl_3$, 400 MHz) δ (ppm): 8.36 (d, J = 8.6 Hz, 1H, Ar), 7.85 (s, 1H, Ar), 7.46 (d, J = 2.0 Hz, 1H, Ar),

7.26 (dd, J = 8.6, 2.0 Hz, 1H, Ar), 7.24 - 6.97 (m, 10H, Ar), 6.75 (s, 1H, Ar), 5.30 (br s, 1H, NHCbz), 4.93 (s, 2H, $NHCOOC\underline{H_2}$), 4.43 (br s, 1H, NHCON), 4.25 (t, J = 6.0 Hz, 2H, OCH_2), 3.56 (t, J = 6.0 Hz, 2H, $OCH_2C\underline{H_2}$), 3.38 (t, J = 7.9 Hz, 2H, $C\underline{H_2}CH_2NHCON$), 3.15 - 3.05 (m, 4H, $CH_2C\underline{H_2}NHCON$, $C\underline{H_2}NHCbz$), 3.01 - 2.82 (m, 4H, $C\underline{H_2}C\underline{H_2}Ph$), 2.69 (t, J = 7.1 Hz, 2H, $TrizCH_2$), 1.79 (quin, J = 6.9 Hz, 2H, $TrizCH_2C\underline{H_2}$); ^{13}C NMR ($CDCl_3$, 100 MHz) δ (ppm): 162.8 (NHCON), 160.3 (OC_{Ar}), 156.7 (C_{Ar}), 156.1 (C_{Ar}), 155.9 (C_{Ar}), 148.0 (C_{Ar}), 141.7 (C_{Ar}), 138.4 (C_{Ar}), 136.7 (C_{Ar}), 130.1 (CH_{Ar}), 128.7 (CH_{Ar}), 128.7 (CH_{Ar}), 128.6 (CH_{Ar}), 128.5 (C_{Ar}), 128.4 (CH_{Ar}), 128.3 (CH_{Ar}), 126.1 (CH_{Ar}), 122.7 (C_{Ar}), 119.6 (CH_{Ar}), 115.1 (CH_{Ar}), 112.4 (CH_{Ar}), 104.5 (CH_{Ar}), 67.6 (OCH_2), 66.9 ($NHCOO\underline{C}H_2$), 46.5 ($\underline{C}H_2CH_2NHCON$), 42.8 ($OCH_2\underline{C}H_2$), 40.0 ($CH_2NHCbz$), 38.4 ($CH_2\underline{C}H_2NHCON$), 35.7 ($CH_2CH_2Ph$), 33.5 ($CH_2CH_2Ph$), 29.2 ($TrizCH_2\underline{C}H_2$), 22.5 ($Triz\underline{C}H_2$); HRMS (ES, positive mode) m/z: calculated for $C_{35}H_{37}N_7O_4S$ 651.2628; Found 651.2623 (-0.74 ppm).

Benzyl-(E)-(3-(1-(4-(4-(2-([1,1'-biphenyl]-4-yl)vinyl)thiazol-2-yl)-3-(2-(2-oxoimidazolidin-1-yl)ethoxy)phenyl)-1H-1,2,3-triazol-4-yl)propyl)carbamate (11i). Following the general Hantzsch procedure, thioamide **8d** (281 mg, 0.54 mmol) the α-bromoketone **10c** (1 eq) (162 mg, 0.54 mmol) were reacted for 4 h. The crude was purified by flash column chromatography (CH_2Cl_2/MeOH, 98:2) to give **11i** (210 mg, 53%) as a colorless oil. ^1H NMR ($CDCl_3$, 400 MHz) δ (ppm): 8.59 (d, J = 8.6 Hz, 1H, Ar), 7.98 (s, 1H, Ar), 7.65 - 7.50 (m, 9H, Ar, ThiazCH=C$\underline{H}$), 7.44 - 7.35 (m, 3H, Ar), 7.33 - 7.23 (m, 6H, Ar), 7.13 (d, J = 16.1 Hz, 1H, ThiazC$\underline{H}$=CH), 5.41 (br s, 1H, NHCbz), 5.04 (s, 2H, $NHCOOC\underline{H_2}$), 4.51 (br s, 1H, NHCON), 4.38 (t, J = 5.8 Hz, 2H, OCH_2), 3.69 (t, J = 6.1 Hz, 2H, $OCH_2C\underline{H_2}$), 3.50 (t, J = 7.9 Hz, 2H, $C\underline{H_2}CH_2NHCON$), 3.22 - 3.16 (m, 4H, $CH_2C\underline{H_2}NHCON$, $C\underline{H_2}NHCbz$), 2.81 (t, J = 7.1 Hz, 2H, $TrizCH_2$), 1.92 (quin, J = 7.3 Hz, 2H, $TrizCH_2C\underline{H_2}$); ^{13}C NMR ($CDCl_3$, 100 MHz) δ (ppm): 162.6 (NHCON), 161.0 (OC_{Ar}), 156.2 (C_{Ar}), 156.1 (C_{Ar}), 153.9 (C_{Ar}), 148.0 (C_{Ar}), 140.8 (C_{Ar}), 140.7 (C_{Ar}), 138.7 (C_{Ar}), 136.6 (C_{Ar}), 136.3 (C_{Ar}), 131.0 (CH_{Ar}), 130.4 (CH_{Ar}), 130.2 (CH_{Ar}), 128.9 (CH_{Ar}), 128.7 (CH_{Ar}), 128.4 (CH_{Ar}), 128.3 (CH_{Ar}), 127.5 (CH_{Ar}), 127.3 (CH_{Ar}), 127.1 (CH_{Ar}), 121.6 (CH_{Ar}),

119.7 (CH_{Ar}), 117.0 (CH_{Ar}), 112.5 (CH_{Ar}), 104.4 (CH_{Ar}), 67.5 (OCH_2), 66.9 (NHCOO$\underline{C}H_2$), 46.5 ($\underline{C}H_2CH_2$NHCON), 42.8 ($OCH_2\underline{C}H_2$), 40.0 (CH_2NHCbz), 38.4 ($CH_2\underline{C}H_2$NHCON), 29.2 (Triz$CH_2\underline{C}H_2$), 22.5 (Triz$\underline{C}H_2$); HRMS (ES, positive mode) m/z: calculated for $C_{41}H_{39}N_7O_4S$ 725.2784; Found 725.2797 (1.69 ppm).

Benzyl-(4-(1-(3-methoxy)-4-(4-phenethylthiazol-2-yl)phenyl)-1H-1,2,3-triazol-4-yl)butyl)carbamate (11j). Following the general procedure for thiazole synthesis, thioamide **8c** (125 mg, 0.28 mmol) and the commercially available 1-bromo-4-phenylbutan-2-one **10a** (65 mg, 0.28 mmol) were reacted. After the work-up, the final residue was purified by CCTLC on the Chromatotron (CH_2Cl_2/MeOH, 99:1) to yield **11j** (105 mg, 63%) as a colorless oil. ^{1}H NMR ($CDCl_3$, 400 MHz) δ (ppm): 8.47 (d, J = 8.5 Hz, 1H, Ar), 7.74 (s, 1H, Ar), 7.53 (d, J = 2.1 Hz, 1H, Ar), 7.37 - 7.02 (m, 11H, Ar), 6.87 (s, 1H, Ar), 5.02 (s, 2H, NHCOOCH_2), 4.78 (br s, 1H, NHCbz), 4.03 (s, 3H, OCH_3), 3.19 (q, J = 6.7 Hz, 2H, $C\underline{H_2}$NHCbz), 3.14 - 2.99 (m, 4H, $C\underline{H_2}C\underline{H_2}$Ph), 2.76 (t, J = 7.5 Hz, 2H, TrizCH_2), 1.72 (quin, J = 7.5 Hz, 2H, Triz$CH_2C\underline{H_2}$), 1.56 (quin, J = 7.1 Hz, 2H, $C\underline{H_2}CH_2$NHCbz); ^{13}C NMR ($CDCl_3$, 75 MHz) δ (ppm): 160.4 (OC_{Ar}), 157.2 (C_{Ar}), 156.6 (C_{Ar}), 156.0 (NHCOO), 148.8 (C_{Ar}), 141.7 (C_{Ar}), 138.3 (C_{Ar}), 136.7 (C_{Ar}), 129.7 (CH_{Ar}), 128.6 (CH_{Ar}), 128.6 (CH_{Ar}), 128.6 (CH_{Ar}), 128.5 (CH_{Ar}), 128.2 (CH_{Ar}), 128.2 (C_{Ar}), 126.1 (CH_{Ar}), 122.7 (C_{Ar}), 119.1 (CH_{Ar}), 115.3 (CH_{Ar}), 111.9 (C_{Ar}), 103.9 (CH_{Ar}), 66.8 (NHCOO$\underline{C}H_2$), 56.2 (OCH_3), 40.9 (CH_2NHCbz), 35.7 (CH_2CH_2Ph), 33.5 (CH_2CH_2Ph), 29.6 ($\underline{C}H_2CH_2$NHCbz), 26.5 (Triz$CH_2\underline{C}H_2$), 25.3 (Triz$\underline{C}H_2$); HRMS (ES, positive mode) m/z: calculated for $C_{32}H_{33}N_5O_3S$ 567.2304; Found 567.2312 (1.38 ppm).

Benzyl-(4-(1-(3-methoxy-4-(4-(2-([1,1'-biphenyl]-4-yl)vinyl)thiazol-2-yl)phenyl)-1H-1,2,3-triazol-4-yl)butyl)carbamate (11k). Following the general Hantzsch procedure, thioamide **8c** (292 mg, 0.66 mmol) and α-bromoketone **10c** (200 mg, 0.66 mmol) were reacted for 5h. The final residue was purified by CCTLC on the Chromatotron (CH_2Cl_2/MeOH, 98:2) to give **11k** (250 mg, 57%) as a yellow oil. ^{1}H NMR ($CDCl_3$, 400

MHz) δ (ppm): 8.63 (d, *J* = 8.5 Hz, 1H, Ar), 7.78 (s, 1H, Ar), 7.63 - 7.53 (m, 8H, Ar, ThiazCH=C$\underline{H}$), 7.41 (t, *J* = 7.6 Hz, 2H, Ar), 7.34 - 7.21 (m, 8H, Ar), 7.17 (d, *J* = 16.0 Hz, 1H, ThiazC$\underline{H}$=CH), 5.06 (s, 2H, $NHCOOCH_2$), 4.83 (br s, 1H, NHCbz), 4.08 (s, 3H, OCH_3), 3.22 (q, *J* = 6.7 Hz, 2H, $C\underline{H_2}NHCbz$), 2.79 (t, *J* = 7.5 Hz, 2H, $TrizCH_2$), 1.76 (quin, *J* = 7.5 Hz, 2H, $TrizCH_2C\underline{H_2}$), 1.59 (quin, *J* = 7.1 Hz, 2H, $C\underline{H_2}CH_2NHCbz$); ^{13}C NMR ($CDCl_3$, 100 MHz) δ (ppm): 161.1 (NHCON), 157.4 (OC_{Ar}), 156.6 (NHCOO), 153.3 (C_{Ar}), 148.8 (C_{Ar}), 140.7 (C_{Ar}), 140.6 (C_{Ar}), 138.6 (CH_{Ar}), 136.7 (CH_{Ar}), 136.3 (CH_{Ar}), 130.8 (CH_{Ar}), 129.9 (CH_{Ar}), 128.9 (CH_{Ar}), 128.6 (CH_{Ar}), 128.2 (CH_{Ar}), 128.2 (CH_{Ar}), 127.5 (CH_{Ar}), 127.2 (CH_{Ar}), 127.0 (CH_{Ar}), 122.4 (C_{Ar}), 121.7 (CH_{Ar}), 119.1 (C_{Ar}), 117.2 (C_{Ar}), 111.9 (CH_{Ar}), 103.8 (CH_{Ar}), 66.7 ($NHCOO\underline{C}H_2$), 56.2 (OCH_3), 40.9 (CH_2NHCbz), 29.6 ($\underline{C}H_2CH_2NHCbz$), 26.5 ($TrizCH_2\underline{C}H_2$), 25.3 ($Triz\underline{C}H_2$); HRMS (ES, positive mode) m/z: calculated for $C_{38}H_{35}N_5O_3S$ 641.2461; Found 641.2474 (2.07 ppm).

Tert-butyl-(4-(1-(4-(4-isopropoxythiazol-2-yl)-3-(2-(2-oxoimidazolidin-1-yl)ethoxy)phenyl)-1H-1,2,3-triazol-4-yl)butyl)carbamate and 1-(2-(5-(4-(4-Aminobutyl)-1H-1,2,3-triazol-1-yl)-2-(4-isopropoxythiazol-2-yl)phenoxy)ethyl)imidazolidin-2-one chloride (11l and 12l). Following the general Hantzsch procedure, thioamide **8a** (87 mg, 0.17 mmol) and isopropyl chloroacetate (0.25 mL, 1.99 mmol) in ^iPrOH (8 mL) were reacted. Work-up and purification of the crude by flash column chromatography (from CH_2Cl_2/MeOH, 97:3 to $CH_2Cl_2/MeOH/NH_3$, 85:14:1) gave, from the fastest moving band, compound **11l** (27 mg, 26%) as a colorless oil. ^1H NMR (DMSO-d_6, 400 MHz) δ (ppm): 8.72 (s, 1H, Ar), 8.34 (d, *J* = 8.6 Hz, 1H, Ar), 7.73 (d, *J* = 1.6 Hz, 1H, Ar), 7.66 (dd, *J* = 8.6, 1.8 Hz, 1H, Ar), 6.81 (t, *J* = 1.9 Hz, 1H, Ar), 6.64 (br s, 1H, NHCON), 6.56 (br s, 1H, NHBoc), 4.73 (quin, *J* = 6.1 Hz, 1H, $OC\underline{H}(CH_3)_2$), 4.47 (t, *J* = 5.6 Hz, 2H, OCH_2), 3.63 (t, *J* = 5.6 Hz, 2H, $OCH_2C\underline{H_2}$), 3.49 (t, *J* = 7.8 Hz, 2H, $C\underline{H_2}CH_2NHCON$), 3.23 (t, *J* = 7.9 Hz, 2H, $CH_2C\underline{H_2}NHCON$), 3.01 - 2.91 (m, 2H, $C\underline{H_2}NHBoc$), 2.75 - 2.68 (m, 2H, $TrizCH_2$), 1.71 - 1.60 (m, 2H, $TrizCH_2C\underline{H_2}$), 1.53 - 1.41 (m, 2H, $C\underline{H_2}CH_2NHBoc$), 1.36 (s, 9H, $C(CH_3)_3$), 1.32 (d, *J* = 6.1 Hz, 6H, $CH(C\underline{H_3})_2$);

^{13}C NMR (DMSO-d_6, 100 MHz) δ (ppm): 162.2 (NHCON), 156.7 (OC_{Ar}), 155.9 (OC_{Ar}), 155.6 (NHCOO), 148.2 (C_{Ar}), 137.9 (C_{Ar}), 128.6 (CH_{Ar}), 120.9 (CH_{Ar}), 120.3 (C_{Ar}), 112.0 (CH_{Ar}), 104.3 (CH_{Ar}), 93.1 (CH_{Ar}), 77.4 ($O\underline{C}(CH_3)_3$), 71.8 ($O\underline{C}H(CH_3)_2$), 67.6 (OCH_2), 45.3 ($\underline{C}H_2CH_2NHCON$), 42.4 ($OCH_2\underline{C}H_2$), 37.6 ($CH_2\underline{C}H_2NHCON$), 29.0 ($\underline{C}H_2CH_2NHBoc$), 28.3 ($C(\underline{C}H_3)_3$), 26.1 ($TrizCH_2\underline{C}H_2$), 24.7 ($Triz\underline{C}H_2$), 21.9 ($CH(\underline{C}H_3)_2$); MS (ESI, positive mode) m/z: 1193.5 $[2M+Na]^+$, 608.3 $[M+Na]^+$, 586.3 $[M+H]^+$.

The slowest moving band gave the deprotected compound **12l (**43 mg, 47%) as a yellow oil. ^1H NMR (DMSO-d_6, 400 MHz) δ (ppm): 8.83 (s, 1H, Ar), 8.30 (d, J = 8.6 Hz, 1H, Ar), 7.75 (s, 1H, Ar), 7.65 (d, J = 8.3 Hz, 1H, Ar), 6.60 (s, 1H, Ar), 6.42 (br s, 1H, NHCON), 4.73 (quin, J = 6.1 Hz, 1H, $OC\underline{H}(CH_3)_2$), 4.50 - 4.40 (m, 2H, OCH_2), 3.65 - 3.55 (m, 2H, $OCH_2C\underline{H_2}$), 3.48 (t, J = 7.4 Hz, $C\underline{H_2}CH_2NHCON$), 3.21 (t, J = 7.4 Hz, 2H, $CH_2C\underline{H_2}NHCON$), 2.79 - 2.62 (m, 4H, $TrizCH_2$, $C\underline{H_2}NH_3^+$), 1.75 - 1.65 (m, 2H, $TrizCH_2C\underline{H_2}$), 1.63 - 1.53 (m, 2H, $C\underline{H_2}CH_2NH_3^+$), 1.31 (d, J = 6.1 Hz, 6H, CH_3); ^{13}C NMR (DMSO-d_6, 100 MHz) δ (ppm): 162.1 (NHCON), 156.7 (OC_{Ar}), 155.9 (OC_{Ar}), 148.0 (C_{Ar}), 137.9 (C_{Ar}), 128.5 (CH_{Ar}), 120.8 (CH_{Ar}), 120.5 (C_{Ar}), 111.9 (CH_{Ar}), 104.2 (CH_{Ar}), 93.0 (CH_{Ar}), 71.8 (OCH_2), 67.5 ($O\underline{C}H(CH_3)_2$), 45.3 ($\underline{C}H_2CH_2NHCON$), 42.4 ($OCH_2\underline{C}H_2$), 37.6 ($CH_2\underline{C}H_2NHCON$), 28.6 ($\underline{C}H_2CH_2NH_3^+$), 25.8 ($TrizCH_2\underline{C}H_2$), 24.6 ($Triz\underline{C}H_2$), 21.9 ($CH_3$); HPLC (*Gradient A, Agilent*): R_t = 3.5 min; HRMS (ES, positive mode) m/z: calculated for $C_{23}H_{31}N_7O_3S$ 485.2203; Found 485.2209 (-1.19 ppm); Anal. Calc. for $C_{23}H_{31}N_7O_3S.HCl$: C. 52.92; H. 6.18; N. 18.78; S. 6.14; Found: C. 53.15; H. 6.16; N. 19.05; S. 5.65.

Tert-butyl-(4-(1-(4-(4-neopentylthiazol-2-yl)-3-(2-(2-oxoimidazolidin-1-yl)ethoxy)phenyl)-1H-1,2,3-triazol-4-yl)butyl)carbamate and 1-(2-(5-(4-(4-Aminobutyl)-1H-1,2,3-triazol-1-yl)-2-(4-neopentylthiazol-2-yl)phenoxy)ethyl)imidazolidin-2-one bromide (11m and 12m). Following the general Hantzsch procedure, thioamide **8a** (50 mg, 0.10 mmol) was reacted with α-bromoketone **10m** (77 mg, 0.40 mmol). Purification by flash column chromatography (from CH_2Cl_2/MeOH, 96:4 to CH_2Cl_2/MeOH/NH_3, 85:14:1) gave, from the fastest moving band,

compound **11m** (27 mg, 46%) as a colorless oil. ^{1}H NMR (CD_3OD, 400 MHz) δ (ppm): 8.45 (s, 1H, Ar), 8.44 (d, *J* = 8.6 Hz, 1H, Ar), 7.72 (d, *J* = 2.0 Hz, 1H, Ar), 7.58 (dd, *J* = 8.6, 2.0 Hz, 1H, Ar), 7.21 (s, 1H, Ar), 4.50 (t, *J* = 5.5 Hz, 2H, OCH_2), 3.77 (t, *J* = 5.5 Hz, 2H, $OCH_2C\underline{H_2}$), 3.62 (dd, *J* = 9.2, 6.9 Hz, 2H, $C\underline{H_2}CH_2NHCON$), 3.38 (dd, *J* = 9.3, 6.9 Hz, 2H, $CH_2C\underline{H_2}NHCON$), 3.10 (t, *J* = 6.9 Hz, 2H, $C\underline{H_2}NHBoc$), 2.82 (t, *J* = 7.5 Hz, 2H, $TrizCH_2$), 2.75 (s, 2H, CH_2-^{t}Bu), 1.77 (quin, *J* = 7.6 Hz, 2H, $TrizCH_2C\underline{H_2}$), 1.59 (quin, *J* = 7.6 Hz, 2H, $C\underline{H_2}CH_2NHBoc$), 1.43 (s, 9H, $OC(CH_3)_3$), 1.00 (s, 9H, $CH_2C(C\underline{H_3})_3$); ^{13}C NMR ($CD_3OD$, 75 MHz) δ (ppm): 165.1 (NHCON), 161.1 (OC_{Ar}), 157.4 (C_{Ar}), 155.8 (C_{Ar}), 150.0 (C_{Ar}), 139.7 (C_{Ar}), 130.8 (CH_{Ar}), 123.9 (C_{Ar}), 121.5 (CH_{Ar}), 118.4 (CH_{Ar}), 113.6 (CH_{Ar}), 105.8 (CH_{Ar}), 79.9 ($O\underline{C}(CH_3)_3$), 68.4 (OCH_2), 46.9 ($\underline{C}H_2CH_2NHCON$), 45.7 ($CH_2$-^{t}Bu), 43.9 ($OCH_2\underline{C}H_2$), 41.0 ($CH_2NHBoc$), 39.3 ($CH_2\underline{C}H_2NHCON$), 32.4 ($CH_2\underline{C}(CH_3)_3$), 30.4 ($\underline{C}H_2CH_2NHBoc$), 30.0 ($CH_2C(\underline{C}H_3)_3$), 28.8 ($OC(\underline{C}H_3)_3$), 27.6 ($TrizCH_2\underline{C}H_2$), 26.0 ($Triz\underline{C}H_2$); HRMS (ES, positive mode) m/z: calculated for $C_{30}H_{43}N_7O_4S$ 597.3097; Found 597.3096 (-0.18 ppm).

The slowest moving band afforded 17 mg (30%) of a yellow oil identified as **12m**.

^{1}H NMR (CD_3OD, 400 MHz) δ (ppm): 8.46 (s, 1H, Ar), 8.46 (d, *J* = 8.6 Hz, 1H, Ar), 7.73 (d, *J* = 2.1 Hz, 1H, Ar), 7.59 (dd, *J* = 8.6, 2.1 Hz, 1H, Ar), 7.22 (s, 1H, Ar), 4.50 (t, *J* = 5.5 Hz, 2H, OCH_2), 3.77 (t, *J* = 5.5 Hz, 2H, $OCH_2C\underline{H_2}$), 3.62 (dd, *J* = 9.2, 6.9 Hz, 2H, $C\underline{H_2}CH_2NHCON$), 3.38 (dd, *J* = 9.3, 6.9 Hz, 2H, $CH_2C\underline{H_2}NHCON$), 2.83 (t, *J* = 7.5 Hz, 2H, $C\underline{H_2}NH_3^+$), 2.75 (s, 2H, CH_2-^{t}Bu), 2.70 (t, *J* = 7.2 Hz, 2H, $TrizCH_2$), 1.79 (quin, *J* = 7.6 Hz, 2H, $TrizCH_2C\underline{H_2}$), 1.58 (quin, *J* = 7.6 Hz, 2H, $C\underline{H_2}CH_2NH_3^+$), 1.00 (s, 9H, CH_3); ^{13}C NMR (CD_3OD, 75 MHz) δ (ppm): 165.1 (NHCON), 161.1 (OC_{Ar}), 157.4 (C_{Ar}), 155.8 (C_{Ar}), 150.1 (C_{Ar}), 139.7 (C_{Ar}), 130.8 (CH_{Ar}), 123.9 (C_{Ar}), 121.5 (CH_{Ar}), 118.4 (CH_{Ar}), 113.6 (CH_{Ar}), 105.7 (CH_{Ar}), 68.4 (OCH_2), 46.9 ($\underline{C}H_2CH_2NHCON$), 45.7 ($CH_2$-^{t}Bu), 43.9 ($OCH_2\underline{C}H_2$), 42.2 ($CH_2NH_3^+$), 39.3 ($CH_2\underline{C}H_2NHCON$), 33.2 ($\underline{C}H_2CH_2NH_3^+$), 32.4 ($CH_2\underline{C}(CH_3)_3$), 30.0 $C(\underline{C}H_3)_3$), 27.7 ($TrizCH_2\underline{C}H_2$), 26.1 ($Triz\underline{C}H_2$); HPLC (*Gradient A, Agilent*): R_t = 4.1 min; HRMS (ES, positive mode) m/z: calculated for $C_{25}H_{35}N_7O_2S$ 497.2573; Found 497.2572 (-0.17 ppm); Anal. Calc. for $C_{25}H_{35}N_7O_2S.HBr$: C. 51.90; H. 6.27; N. 16.95; S. 5.54; Found: C. 52.27; H. 6.17; N. 16.79; S. 5.65.

Benzyl-(4-(1-(3-(2-(2-oxoimidazolidin-1-yl)ethoxy)-4-(4-phenylthiazol-2-yl)phenyl)-1H-1,2,3-triazol-4-yl)butyl)carbamate (11n). Following the general Hantzsch procedure, the thioamide **8b** (100 mg, 0.19 mmol) and the commercially available 1-bromo-2-phenylethan-2-one (37 mg, 0.19 mmol) were reacted for 3 h. After the work-up, the residue was purified by flash column chromatography (CH_2Cl_2/MeOH, 95:5) to give **11n** (104 mg, 86%) as a colorless oil. ^{1}H NMR (DMSO-d_6, 400 MHz) δ (ppm): 8.74 (s, 1H, Ar), 8.60 (d, *J* = 8.6 Hz, 1H, Ar), 8.21 (s, 1H, Ar), 8.10 (dd, *J* = 7.7, 2.0 Hz, 2H, Ar), 8.09 (s, 1H, Ar), 7.77 (d, *J* = 1.9 Hz, 1H, Ar), 7.72 (dd, *J* = 8.6, 1.9 Hz, 1H, Ar), 7.48 (t, *J* = 7.8 Hz, 1H, Ar), 7.42 - 7.24 (m, 6H, Ar), 6.41 (br s, 1H, NHCbz), 5.01 (s, 2H, $NHCOOC\underline{H_2}$), 4.50 (t, *J* = 5.7 Hz, 2H, OCH_2), 3.67 (t, *J* = 5.6 Hz, 2H, $OCH_2C\underline{H_2}$), 3.51 (dd, *J* = 9.0, 6.6 Hz, 2H, $C\underline{H_2}CH_2NHCON$), 3.23 (dd, *J* = 9.0, 6.6 Hz, 2H, $CH_2C\underline{H_2}NHCON$), 3.07 (q, *J* = 6.6 Hz, 2H, $C\underline{H_2}NHCbz$), 2.74 (t, *J* = 7.5 Hz, 2H, $TrizCH_2$), 1.70 (quin, *J* = 7.5 Hz, 2H, $TrizCH_2C\underline{H_2}$), 1.51 (quin, *J* = 6.6 Hz, 2H, $C\underline{H_2}CH_2NHCbz$); ^{13}C NMR (DMSO-$d_6$, 75 MHz) δ (ppm): 162.2 (NHCON), 162.1 (OC_{Ar}), 160.1 (C_{Ar}), 156.1 (C_{Ar}), 155.8 (C_{Ar}), 153.4 (C_{Ar}), 148.1 (C_{Ar}), 138.3 (C_{Ar}), 137.3 (C_{Ar}), 134.1 (CH_{Ar}), 129.2 (CH_{Ar}), 128.8 (CH_{Ar}), 128.3 (CH_{Ar}), 128.1 (CH_{Ar}), 127.7 (CH_{Ar}), 126.2 (C_{Ar}), 121.1 (CH_{Ar}), 120.3 (CH_{Ar}), 115.8 (CH_{Ar}), 112.1 (CH_{Ar}), 104.3 (CH_{Ar}), 67.6 (OCH_2), 65.1 ($NHCOO\underline{C}H_2$), 45.3 ($\underline{C}H_2CH_2NHCON$), 42.4 ($OCH_2\underline{C}H_2$), 37.6 ($CH_2\underline{C}H_2NHCON$), 28.9 ($\underline{C}H_2CH_2NHCbz$), 26.0 ($TrizCH_2\underline{C}H_2$), 24.7 ($Triz\underline{C}H_2$); HRMS (ES, positive mode) m/z: calculated for $C_{34}H_{35}N_7O_4S$ 637.2471; Found 637.2474 (0.49 ppm).

Tert-Butyl-(4-(1-(4-(4-benzylthiazol-2-yl)-3-(2-(2-oxoimidazolidin-1-yl)ethoxy)phenyl)-1H-1,2,3-triazol-4-yl)butyl)carbamate and 1-(2-(5-(4-(4-Aminobutyl)-1H-1,2,3-triazol-1-yl)-2-(4-benzylthiazol-2-yl)phenoxy)ethyl)imidazolidin-2-one chloride (11o and 12o). According to the general Hantzsch procedure, thioamide **8a** (74 mg, 0.15 mmol) and 1-chloro-3-benzylpropan-2-one (100 mg, 0.60 mmol) were reacted. The final crude was purified by flash column chromatography (from CH_2Cl_2/MeOH, 95:5 to CH_2Cl_2/MeOH/NH_3, 85:14:1) to give, from the fastest moving band compound **11o** (41 mg, 44%) as a

colorless oil. ^{1}H NMR (DMSO-d$_6$, 300 MHz) δ (ppm): 8.72 (s, 1H, Ar), 8.40 (d, *J* = 8.6 Hz, 1H, Ar), 7.73 (d, *J* = 2.0 Hz, 1H, Ar), 7.66 (dd, *J* = 8.4, 2.0 Hz, 1H, Ar), 7.40 - 7.14 (m, 6H, Ar), 6.80 (br s, 1H, NHCON), 6.39 (br s, 1H, NHBoc), 4.46 (t, *J* = 5.5 Hz, 2H, OCH$_2$), 4.14 (s, 2H, CH$_2$Ph), 3.62 (t, *J* = 5.5 Hz, 2H, OCH$_2$C$\underline{H_2}$), 3.53 - 3.42 (m, 2H, C$\underline{H_2}$CH$_2$NHCON), 3.25 - 3.15 (m, 2H, CH$_2$C$\underline{H_2}$NHCON), 3.00 - 2.90 (m, 2H, C$\underline{H_2}$NHBoc), 2.72 (t, *J* = 7.6 Hz, 2H, TrizCH$_2$), 1.75 - 1.60 (m, 2H, TrizCH$_2$C$\underline{H_2}$), 1.52 - 1.43 (m, 2H, C$\underline{H_2}$CH$_2$Boc), 1.36 (s, 9H, OC(CH$_3$)$_3$)**;** HRMS (ES, positive mode) m/z: calculated for $C_{32}H_{39}N_7O_4S$ 617.2784; Found 617.2775 (-1.44 ppm).

The slowest moving band afforded 44 mg (55%) of a yellow oil that was identified as **12o**. ^{1}H NMR (DMSO-d$_6$, 300 MHz) δ (ppm): 8.73 (s, 1H, Ar), 8.39 (d, *J* = 8.6 Hz, 1H, Ar), 7.74 (s, 1H, Ar), 7.66 (d, *J* = 8.4 Hz, 1H, Ar), 7.55 - 7.05 (m, 5H, Ar), 7.19 (s, 1H, Ar), 6.43 (br s, 1H, NHCON), 4.46 (t, *J* = 5.4 Hz, 2H, OCH$_2$), 4.14 (s, 2H, CH$_2$Ph), 3.62 (t, *J* = 5.4 Hz, 2H, OCH$_2$C$\underline{H_2}$), 3.53 - 3.42 (m, 2H, C$\underline{H_2}$CH$_2$NHCON), 3.25 - 3.15 (m, 2H, CH$_2$C$\underline{H_2}$NHCON), 2.99 - 2.88 (m, 2H, C$\underline{H_2}$NH$_3^+$), 2.75 - 2.67 (m, 2H, TrizCH$_2$), 1.75 - 1.60 (m, 2H, TrizCH$_2$C$\underline{H_2}$), 1.48 - 1.37 (m, 2H, C$\underline{H_2}$CH$_2$NH$_3^+$); ^{13}C NMR (DMSO-d$_6$, 75 MHz) δ (ppm): 162.1 (NHCON), 155.6 (C$_{Ar}$), 155.0 (C$_{Ar}$), 148.3 (C$_{Ar}$), 139.6 (C$_{Ar}$), 138.1 (C$_{Ar}$), 128.9 (CH$_{Ar}$), 128.8 (CH$_{Ar}$), 128.4 (CH$_{Ar}$), 126.1 (CH$_{Ar}$), 121.1 (C$_{Ar}$), 120.0 (CH$_{Ar}$), 116.7 (CH$_{Ar}$), 112.0 (CH$_{Ar}$), 104.3 (CH$_{Ar}$), 67.6 (OCH$_2$), 45.2 ($\underline{C}$H$_2$CH$_2$NHCON), 42.3 (OCH$_2\underline{C}$H$_2$), 40.4 (CH$_2$NH$_3^+$), 37.5 (CH$_2\underline{C}$H$_2$NHCON), 37.0 (CH$_2$Ph), 30.7 ($\underline{C}$H$_2$CH$_2$NH$_3^+$), 29.7 (TrizCH$_2\underline{C}$H$_2$), 26.1 (Triz$\underline{C}$H$_2$); HPLC (*Gradient A, Agilent*): R_t = 4.4 min; HRMS (ES, positive mode) m/z: calculated for $C_{27}H_{31}N_7O_2S$ 517.2260; Found 517.2256 (-0.69 ppm); Anal. Calc. for $C_{27}H_{31}N_7O_2S$.HCl: C. 58.53; H. 5.82; N. 17.69; S. 5.79; Found: C. 59.02; H. 6.15; N. 17.63; S. 6.16.

Benzyl-(E)-(4-(1-(3-(2-(2-oxoimidazolidin-1-yl)ethoxy)-4-(4-(3-phenylprop-1-en-1-yl)thiazol-2-yl)phenyl)-1H-1,2,3-triazol-4-yl)butyl)carbamate (11p). Following the general Hantzsch procedure, thioamide **8b** (250 mg, 0.47 mmol) and α-bromoketone **10p** (112 mg, 0.47 mmol) were reacted. The final residue was purified by flash column

chromatography (CH_2Cl_2/MeOH, 97:3) to give **11p** (153 mg, 48%) as a yellow oil. ^{1}H NMR ($CDCl_3$, 400 MHz) δ (ppm): 8.55 (d, *J* = 8.5 Hz, 1H, Ar), 7.87 (s, 1H, Ar), 7.57 (d, *J* = 1.9 Hz, 1H, Ar), 7.40 - 7.18 (m, 11H, Ar), 7.10 (s, 1H, Ar), 6.86 (dt, *J* = 15.5, 6.9 Hz, 1H, $CH_2CH{=}C\underline{H}$), 6.50 (dt, *J* = 15.5, 1.6 Hz, 1H, $CH_2C\underline{H}{=}CH$), 5.05 (br s, 1H, NHCbz), 5.08 (s, 2H, $NHCOOC\underline{H_2}$), 4.73 (br s, 1H, NHCON), 4.41 (t, *J* = 5.7 Hz, 2H, OCH_2), 3.75 (t, *J* = 5.7 Hz, 2H, $OCH_2C\underline{H_2}$), 3.61 - 3.52 (m, 4H, $C\underline{H_2}CH_2NHCON$, $C\underline{H_2}CH{=}CH$), 3.32 (t, *J* = 7.9 Hz, 2H, $CH_2C\underline{H_2}NHCON$), 3.25 (q, *J* = 6.7 Hz, 2H, $C\underline{H_2}NHCbz$), 2.82 (t, *J* = 7.3 Hz, 2H, $TrizCH_2$), 1.77 (quin, *J* = 7.5 Hz, 2H, $TrizCH_2C\underline{H_2}$), 1.63 (quin, *J* = 7.3 Hz, 2H, $C\underline{H_2}CH_2NHCbz$); ^{13}C NMR ($CDCl_3$, 100 MHz) δ (ppm): 162.7 (NHCON), 160.6 (OC_{Ar}), 156.6 (C_{Ar}), 156.1 (C_{Ar}), 153.3 (C_{Ar}), 148.8 (C_{Ar}), 140.0 (C_{Ar}), 138.5 (C_{Ar}), 136.7 (C_{Ar}), 132.6 (CH_{Ar}), 130.2 (CH_{Ar}), 128.9 (CH_{Ar}), 128.6 (CH_{Ar}), 128.2 (CH_{Ar}), 126.3 (CH_{Ar}), 124.3 (CH_{Ar}), 122.5 (C_{Ar}), 119.1 (CH_{Ar}), 115.1 (CH_{Ar}), 112.4 (CH_{Ar}), 104.5 (CH_{Ar}), 67.8 (OCH_2), 66.7 ($NHCOO\underline{C}H_2$), 46.4 ($\underline{C}H_2CH_2NHCON$), 43.0 ($OCH_2\underline{C}H_2$), 40.9 ($CH_2NHCbz$), 39.3 ($CH_2\underline{C}H_2NHCON$), 38.4 ($Ph\underline{C}H_2CH{=}CH$), 29.4 ($\underline{C}H_2CH_2NHCbz$), 26.4 ($TrizCH_2\underline{C}H_2$), 25.2 ($Triz\underline{C}H_2$); HRMS (ES, positive mode) m/z: calculated for $C_{37}H_{39}N_7O_4S$ 677.2784; Found 677.2781 (-0.53 ppm).

Benzyl-(E)-(4-(1-(3-(2-(2-oxoimidazolidin-1-yl)ethoxy)-4-(4-(2-(quinolin-6-yl)vinyl)thiazol-2-yl)phenyl)-1H-1,2,3-triazol-4-yl)butyl)carbamate (11q). According to the general Hantzsch procedure, thioamide **8b** (200 mg, 0.38 mmol) and the α-bromoketone crude **10q** (0.38 mmol) were reacted. After the work-up, the final residue was purified by CCTLC on the Chromatotron (CH_2Cl_2/MeOH, 90:10) to afford **11q (**230 mg, 80%) as an orange oil. ^{1}H NMR ($CDCl_3$, 400 MHz) δ (ppm): 8.79 (d, *J* = 4.2 Hz, 1H, Ar), 8.59 (d, *J* = 8.6 Hz, 1H, Ar), 8.06 (dd, *J* = 6.5, 2.2 Hz, 1H, Ar), 8.01 (d, *J* = 8.8 Hz, 1H, Ar), 7.91 (dd, *J* = 8.9, 2.0 Hz, 1H, Ar), 7.83 (s, 1H, Ar), 7.80 (s, 1H, Ar), 7.69 (d, *J* = 15.9 Hz, 1H, $ThiazCH{=}C\underline{H}$), 7.53 (s, 1H, Ar), 7.37 (d, *J* = 8.6 Hz, 1H, Ar), 7.34 - 7.15 (m, 8H, Ar, $ThiazC\underline{H}{=}CH$), 5.06 (br s, 1H, NHCbz), 5.02 (s, 2H, $NHCOOC\underline{H_2}$), 4.67 (br s, 1H,NHCON), 4.37 (t, *J* = 5.8 Hz, 2H, OCH_2), 3.71 (t, *J* = 5.9 Hz, 2H, $OCH_2C\underline{H_2}$), 3.52 (dd,

J = 9.0, 6.7 Hz, 2H, $C\underline{H_2}CH_2NHCON$), 3.28 (t, *J* = 8.0 Hz, 2H, $CH_2C\underline{H_2}NHCON$), 3.20 (q, *J* = 6.6 Hz, 2H, $C\underline{H_2}NHCbz$), 2.77 (t, *J* = 7.4 Hz, 2H, $TrizCH_2$), 1.72 (quin, *J* = 7.6 Hz, 2H, $TrizCH_2C\underline{H_2}$), 1.57 (quin, *J* = 7.4 Hz, 2H, $C\underline{H_2}CH_2NHCbz$); ^{13}C NMR ($CDCl_3$, 100 MHz) δ (ppm): 162.7 (NHCON), 161.1 (OC_{Ar}), 156.6 (C_{Ar}), 156.2 (C_{Ar}), 153.1 (NHCOO), 150.2 (C_{Ar}), 148.8 (C_{Ar}), 148.2 (C_{Ar}), 138.7 (C_{Ar}), 136.7 (C_{Ar}), 136.2 (CH_{Ar}), 135.5 (CH_{Ar}), 130.6 (CH_{Ar}), 130.3 (CH_{Ar}), 129.8 (C_{Ar}), 128.7 (CH_{Ar}), 128.6 (CH_{Ar}), 128.2 (CH_{Ar}), 127.4 (CH_{Ar}), 126.5 (CH_{Ar}), 122.9 (CH_{Ar}), 122.3 (C_{Ar}), 121.6 (CH_{Ar}), 119.1 (CH_{Ar}), 117.8 (CH_{Ar}), 112.5 (CH_{Ar}), 104.5 (CH_{Ar}), 67.7 (OCH_2), 66.8 ($NHCOO\underline{C}H_2$), 46.4 ($\underline{C}H_2CH_2NHCON$), 43.0 ($OCH_2\underline{C}H_2$), 40.9 ($\underline{C}H_2NHCbz$), 38.4 ($CH_2\underline{C}H_2NHCON$), 29.5 ($\underline{C}H_2CH_2NHCbz$), 26.4 ($TrizCH_2\underline{C}H_2$), 25.2 ($Triz\underline{C}H_2$); HRMS (ES, positive mode) m/z: calculated for $C_{39}H_{38}N_8O_4S$ 714.2737; Found 714.2734 (-0.38 ppm).

Benzyl-(E)-(4-(1-(4-(4-(2-(2,3-dihydrobenzofuran-5-yl)vinyl)thiazol-2-yl)-3-(2-(2-oxoimidazolidin-1-yl)ethoxy)phenyl)-1H-1,2,3-triazol-4-yl)butyl)carbamate (11r). Following the general Hantzsch procedure, thioamide **8b** (250 mg, 0.47 mmol) and the α-bromoketone **10r** (124 mg, 0.47 mmol) were reacted. After work-up, the residue was purified by flash column chromatography (CH_2Cl_2/MeOH, 97:3) to give **11r** (207 mg, 62%) as a yellow oil. ^1H NMR ($CDCl_3$, 400 MHz) δ (ppm): 8.55 (d, *J* = 8.6 Hz, 1H, Ar), 7.81 (s, 1H, Ar), 7.51 (s, 1H, Ar), 7.46 (d, *J* = 15.8 Hz, 1H, $ThiazCH{=}C\underline{H}$), 7.44 - 7.15 (m, 8H, Ar), 7.12 (s, 1H, Ar), 6.92 (d, *J* = 16.0 Hz, 1H, $ThiazC\underline{H}{=}CH$), 6.71 (d, *J* = 8.2 Hz, 1H Ar), 5.04 (br s, 1H, NHCON), 5.03 (s, 2H, $NHCOOC\underline{H_2}$), 4.68 (br s, 1H, NHCbz), 4.52 (t, *J* = 8.7 Hz, 2H, $OC\underline{H_2}CH_2Ph$), 4.35 (t, *J* = 5.7 Hz, 2H, OCH_2), 3.69 (t, *J* = 5.6 Hz, 2H, $OCH_2C\underline{H_2}$), 3.50 (t, *J* = 7.8 Hz, 2H, $C\underline{H_2}CH_2NHCON$), 3.26 (t, *J* = 8.0 Hz, 2H, $CH_2C\underline{H_2}NHCON$), 3.26 - 3.12 (m, 4H, $C\underline{H_2}NHCbz$, $OCH_2C\underline{H_2}Ph$), 2.76 (t, *J* = 7.3 Hz, 2H, $TrizCH_2$), 1.72 (quin, *J* = 7.3 Hz, 2H, $TrizCH_2C\underline{H_2}$), 1.56 (quin, *J* = 7.6 Hz, 2H, $C\underline{H_2}CH_2NHCbz$); ^{13}C NMR ($CDCl_3$, 100 MHz) δ (ppm): 162.7 (NHCON), 160.7 (OC_{Ar}), 160.3 (OC_{Ar}), 156.6 (C_{Ar}), 156.1 (C_{Ar}), 153.8 (C_{Ar}), 148.8 (C_{Ar}), 138.5 (C_{Ar}), 136.7 (C_{Ar}), 131.5 (C_{Ar}), 130.3 (CH_{Ar}), 130.0 (CH_{Ar}), 128.6 (CH_{Ar}), 128.2 (CH_{Ar}), 128.2 (CH_{Ar}), 127.8 (CH_{Ar}), 127.5 (C_{Ar}), 123.0 (CH_{Ar}), 122.5 (C_{Ar}),

119.1 (CH_{Ar}), 118.9 (CH_{Ar}), 115.8 (CH_{Ar}), 112.5 (CH_{Ar}), 109.5 (CH_{Ar}), 104.5 (CH_{Ar}), 71.6 ($O\underline{C}H_2CH_2Ph$), 67.8 (OCH_2), 66.8 ($NHCOO\underline{C}H_2$), 46.4 ($\underline{C}H_2CH_2NHCON$), 43.0 ($OCH_2\underline{C}H_2$), 40.9 ($CH_2NHCbz$), 38.4 ($CH_2\underline{C}H_2NHCON$), 29.7 ($OCH_2\underline{C}H_2Ph$), 29.5 ($\underline{C}H_2CH_2NHCbz$), 26.4 ($TrizCH_2\underline{C}H_2$), 25.2 ($Triz\underline{C}H_2$); HRMS (ES, positive mode) m/z: calculated for $C_{38}H_{39}N_7O_5S$ 705.2733; Found 705.2728 (-0.82 ppm).

Benzyl-(E)-(4-(1-(4-(4-(2-(dibenzo[b,d]furan-2-yl)vinyl)thiazol-2-yl)-3-(2-(2-oxoimidazolidin-1-yl)ethoxy)phenyl)-1H-1,2,3-triazol-4-yl)butyl)carbamate (11s). Following the general Hantzsch synthesis procedure, thioamide **8b** (150 mg, 0.28 mmol) α-bromoketone **10s** (88 mg, 0.28 mmol) were reacted. The final residue was purified by flash column chromatography (CH2Cl2/MeOH, 98:2) to yield **11s** (121 mg, 58%) as a colorless oil. ^1H NMR (DMSO-d_6, 400 MHz) δ (ppm): 8.70 (br s, 1H, NHCbz), 8.60 (dd, *J* = 8.5, 2.2 Hz, 1H, Ar), 8.19 (d, *J* = 7.6 Hz, 1H, Ar), 7.88 - 7.66 (m, 8H, Ar), 7.60 - 7.50 (m, 1H, Ar), 7.47 - 7.24 (m, 8H, Ar), 6.31 (br s, 1H, NHCON), 5.02 (s, 2H, $NHCOOC\underline{H_2}$), 4.51 (t, *J* = 5.8 Hz, 2H, OCH_2), 3.67 (t, *J* = 5.7 Hz, 2H, $OCH_2C\underline{H_2}$), 3.52 (dd, *J* = 8.8, 6.8 Hz, 2H, $C\underline{H_2}CH_2NHCON$), 3.35 - 3.08 (m, 4H, $CH_2C\underline{H_2}NHCON$, $C\underline{H_2}NHCbz$), 2.76 (t, *J* = 7.5 Hz, 2H, $TrizCH_2$), 1.72 (quin, *J* = 7.2 Hz, 2H, $TrizCH_2C\underline{H_2}$), 1.54 (quin, *J* = 7.2 Hz, 2H, $C\underline{H_2}CH_2NHCbz$); ^{13}C NMR (DMSO-d_6, 75 MHz) δ (ppm): 161.9 (NHCON), 160.1 (OC_{Ar}), 155.8 (OC_{Ar}), 155.8 (OC_{Ar}), 155.0 (NHCOO), 152.6 (C_{Ar}), 147.9 (C_{Ar}), 138.2 (C_{Ar}), 137.1 (C_{Ar}), 132.1 (C_{Ar}), 130.2 (CH_{Ar}), 129.1 (CH_{Ar}), 128.0 (CH_{Ar}), 127.5 (C_{Ar}), 127.4 (CH_{Ar}), 127.3 (CH_{Ar}), 126.1 (CH_{Ar}), 124.0 (C_{Ar}), 123.3 (C_{Ar}), 123.0 (CH_{Ar}), 121.2 (C_{Ar}), 121.1 (CH_{Ar}), 121.0 (CH_{Ar}), 120.0 (CH_{Ar}), 118.7 (CH_{Ar}), 117.4 (CH_{Ar}), 112.0 (CH_{Ar}), 111.6 (CH_{Ar}), 111.4 (CH_{Ar}), 104.5 (CH_{Ar}), 67.5 (OCH_2), 64.9 ($NHCOO\underline{C}H_2$), 45.1 ($\underline{C}H_2CH_2NHCON$), 42.3 ($OCH_2\underline{C}H_2$), 37.4 ($CH_2\underline{C}H_2NHCON$), 28.7 ($\underline{C}H_2CH_2NHCbz$), 25.8 ($TrizCH_2\underline{C}H_2$), 24.5 ($Triz\underline{C}H_2$); HRMS (ES, positive mode) m/z: calculated for $C_{42}H_{39}N_7O_5S$ 753.2733; Found 753.2713 (-2.65 ppm).

1-(2-(5-(4-(4-Hydroxybutyl)-1H-1,2,3-triazol-1-yl)-2-(4-phenethylthiazol-2-yl)phenoxy)ethyl)imidazolidin-2-one (12d). Following the general Hantzsch procedure, thioamide **8g** (50 mg, 0.12 mmol) and the commercially available 1-bromo-4-phenylbutan-2-one **10a** (28 mg, 0.12 mmol) were reacted. After the work-up, the residue was purified flash column chromatography (CH_2Cl_2/MeOH, 95:5) to obtain **12d** (53 mg, 83%) as a colorless oil. ^{1}H NMR (DMSO-d_6, 400 MHz) δ (ppm): 8.74 (s, 1H, Ar), 8.46 (d, *J* = 8.6 Hz, 1H, Ar), 7.75 (d, *J* = 2.1 Hz, 1H, Ar), 7.69 (dd, *J* = 8.6, 2.0 Hz, 1H, Ar), 7.37 (s, 1H, Ar), 7.29 - 7.11 (m, 4H, Ar), 7.20 - 7.15 (m, 1H, Ar), 6.39 (br s, 1H, NHCON), 4.47 (t, *J* = 5.7 Hz, 2H, OCH_2), 4.40 (t, *J* = 5.1 Hz, 2H, OH), 3.63 (t, *J* = 5.6 Hz, 2H, $OCH_2C\underline{H_2}$), 3.54 - 3.36 (m, 4H, $C\underline{H_2}OH$, $C\underline{H_2}CH_2NHCON$), 3.22 (t, *J* = 7.8 Hz, 2H, $CH_2C\underline{H_2}NHCON$), 3.15 - 3.03 (m, 4H, $C\underline{H_2}C\underline{H_2}Ph$), 2.73 (t, *J* = 7.6 Hz, 2H, $TrizCH_2$), 1.73 (quin, *J* = 7.5 Hz, 2H, $TrizCH_2C\underline{H_2}$), 1.58 - 1.37 (m, 2H, $C\underline{H_2}CH_2OH$); ^{13}C NMR (DMSO-d_6, 75 MHz) δ (ppm): 162.5 (NHCON), 159.8 (OC_{Ar}), 156.0 (C_{Ar}), 155.7 (C_{Ar}), 148.7 (C_{Ar}), 141.8 (C_{Ar}), 138.4 (C_{Ar}), 129.4 (CH_{Ar}), 128.7 (CH_{Ar}), 128.6 (CH_{Ar}), 126.2 (CH_{Ar}), 121.6 (C_{Ar}), 120.6 (CH_{Ar}), 116.3 (CH_{Ar}), 112.3 (CH_{Ar}), 104.6 (CH_{Ar}), 68.0 (OCH_2), 60.8 (CH_2OH), 45.7 ($\underline{C}H_2CH_2NHCON$), 42.8 ($OCH_2\underline{C}H_2$), 37.9 ($CH_2\underline{C}H_2NHCON$), 35.1 ($CH_2CH_2Ph$), 33.0 ($CH_2CH_2Ph$), 32.4 ($TrizCH_2\underline{C}H_2$), 25.7 ($Triz\underline{C}H_2$), 25.3 ($\underline{C}H_2CH_2OH$); HPLC (*Gradient A, Agilent*): R_t = 8.0 min; HRMS (ES, positive mode) m/z: calculated for $C_{28}H_{32}N_6O_3S$ 532.2257; Found 532.2258 (0.17 ppm); Anal. Calc. for $C_{28}H_{32}N_6O_3S$: C. 63.14; H. 6.06; N. 15.78; S. 6.02; Found: C. 62.55; H. 6.21; N. 15.41; S. 5.81.

1-(2-(5-(4-butyl-1H-1,2,3-triazol-1-yl)-2-(4-phenethylthiazol-2-yl)phenoxy)ethyl) imidazolidin-2-one (12e). Following the general Hantzsch procedure, thioamide **8f** (50 mg, 0.13 mmol) and the commercially available 1-bromo-4-phenylbutan-2-one **10a** (31 mg, 0.13 mmol) were reacted for 3 h. The crude was purified by CCTLC on the Chromatotron (CH_2Cl_2/MeOH, 96:4) to give **12e** (57 mg, 85%) as a colorless oil. ^{1}H NMR ($CDCl_3$, 400 MHz) δ (ppm): 8.47 (d, *J* = 8.5 Hz, 1H, Ar), 7.73 (s, 1H, Ar), 7.52 (d, *J* = 2.0 Hz, 1H, Ar), 7.28 (dd, *J* = 8.5, 2.0 Hz, 1H, Ar), 7.24 - 7.09 (m, 5H, Ar), 6.87 (s, 1H, Ar),

4.37 (t, *J* = 5.5 Hz, 2H, OCH_2), 3.73 (t, *J* = 5.5 Hz, 2H, $OCH_2C\underline{H_2}$), 3.54 (dd, *J* = 9.0, 6.7 Hz, 2H, $C\underline{H_2}CH_2$NHCON), 3.33 (dd, *J* = 8.7, 7.0 Hz, 2H, $CH_2C\underline{H_2}$NHCON), 3.19 - 2.93 (m, 4H, $C\underline{H_2}C\underline{H_2}$Ph), 2.74 (t, *J* = 7.7 Hz, 2H, TrizCH_2), 1.74 - 1.60 (m, 2H, Triz$CH_2C\underline{H_2}$), 1.46 - 1.30 (m, 2H, $C\underline{H_2}CH_3$), 0.90 (t, *J* = 7.4 Hz, 3H, CH_3); ^{13}C NMR ($CDCl_3$, 75 MHz) δ (ppm): 162.7 (NHCON), 160.3 (OC_{Ar}), 156.1 (C_{Ar}), 156.0 (C_{Ar}), 149.6 (C_{Ar}), 141.7 (C_{Ar}), 138.4 (C_{Ar}), 130.0 (CH_{Ar}), 128.6 (CH_{Ar}), 128.5 (CH_{Ar}), 126.1 (CH_{Ar}), 122.7 (C_{Ar}), 118.8 (CH_{Ar}), 115.2 (CH_{Ar}), 112.4 (CH_{Ar}), 104.6 (CH_{Ar}), 68.2 (OCH_2), 46.5 ($\underline{C}H_2CH_2$NHCON), 43.2 ($OCH_2\underline{C}H_2$), 38.5 ($CH_2\underline{C}H_2$NHCON), 35.7 (CH_2CH_2Ph), 33.5 (CH_2CH_2Ph), 31.6 (Triz$CH_2\underline{C}H_2$), 25.5 (Triz$\underline{C}H_2$), 22.5 ($\underline{C}H_2CH_3$), 14.0 (CH_3); HPLC (*Gradient A, Agilent*): R_t = 9.6 min; HRMS (ES, positive mode) m/z: calculated for $C_{28}H_{32}N_6O_2S$ 516.2308; Found 516.2310 (0.52 ppm); Anal. Calc. for $C_{28}H_{32}N_6O_2S$: C. 65.09; H. 6.24; N. 16.27; S. 6.21; Found: C. 64.69; H. 6.33; N. 15.79; S. 6.01.

1-(2-(5-(4-Butyl-1H-1,2,3-triazol-1-yl)-2-(4-(2-([1,1'-biphenyl]-4-yl)ethyl)thiazol-2-yl)phenoxy)ethyl)imidazolidin-2-one (12f). A solution of **11f** (100 mg, 0.17 mmol) in a 1:1 mixture of THF/MeOH (30 mL) and in the presence of catalytic amount of Pd/C 10% (20% wt/wt) was hydrogenated with hydrogen balloon for 2 h at room temperature. After filtration over PTFE membrane filters, volatiles were removed and the crude was co-evaporated with mixtures of CH_2Cl_2/MeOH (5 x 10 mL). The residue was purified by CCTLC on the Chromatotron (CH_2Cl_2/MeOH, 97:3) to give **12f** (40 mg, 39%) as a white solid. ^{1}H NMR ($CDCl_3$, 400 MHz) δ (ppm): 8.48 (d, *J* = 8.5 Hz, 1H, Ar), 7.73 (s, 1H, Ar), 7.57 - 7.49 (m, 3H, Ar), 7.45 (d, *J* = 8.1 Hz, 2H, Ar), 7.35 (t, *J* = 7.5 Hz, 2H, Ar), 7.30 - 7.14 (m, 4H, Ar), 6.90 (s, 1H, Ar), 4.46 (br s, 1H, NHCON), 4.37 (t, *J* = 5.5 Hz, 2H, OCH_2), 3.74 (t, *J* = 5.5 Hz, 2H, $OCH_2C\underline{H_2}$), 3.54 (dd, *J* = 9.0, 6.7 Hz, 2H, $C\underline{H_2}CH_2$NHCON), 3.33 (dd, *J* = 9.1, 6.6 Hz, 2H, $CH_2C\underline{H_2}$NHCON), 3.17 - 3.02 (m, 4H, Thiaz$C\underline{H_2}C\underline{H_2}$), 2.75 (t, *J* = 7.7 Hz, 2H, TrizCH_2), 1.68 (quin, *J* = 7.5 Hz, 2H, Triz$CH_2C\underline{H_2}$), 1.38 (sex, *J* = 7.5 Hz, 2H, $C\underline{H_2}CH_3$), 0.90 (t, *J* = 7.4 Hz, 3H, CH_3); ^{13}C NMR ($CDCl_3$, 75 MHz) δ (ppm): 162.6 (NHCON), 160.4 (OC_{Ar}), 156.0 (C_{Ar}), 156.0 (C_{Ar}), 149.6 (C_{Ar}), 141.1 (C_{Ar}), 139.0 (C_{Ar}),

138.5 (C_{Ar}), 130.0 (CH_{Ar}), 129.0 (CH_{Ar}), 128.9 (CH_{Ar}), 127.2 (CH_{Ar}), 127.2 (CH_{Ar}), 127.1 (CH_{Ar}), 122.7 (C_{Ar}), 118.8 (CH_{Ar}), 115.3 (CH_{Ar}), 112.6 (CH_{Ar}), 104.6 (CH_{Ar}), 68.2 (OCH_2), 46.5 ($\underline{C}H_2CH_2NHCON$), 43.2 ($OCH_2\underline{C}H_2$), 38.5 ($CH_2\underline{C}H_2NHCON$), 35.3 ($ThiazCH_2CH_2$), 33.4 ($ThiazCH_2CH_2$), 31.6 ($TrizCH_2\underline{C}H_2$), 25.5 ($Triz\underline{C}H_2$), 22.5 ($\underline{C}H_2CH_3$), 14.0 ($CH_3$); HPLC (*Gradient A, Agilent*): R_t = 9.6 min; HRMS (ES, positive mode) m/z: calculated for $C_{34}H_{36}N_6O_2S$ 592.2621; Found 592.2610 (-1.83 ppm); Anal. Calc. for $C_{34}H_{36}N_6O_2S$: C. 68.89; H. 6.12; N. 14.18; S. 5.41; Found: C. 68.38; H. 6.27; N. 13.90; S. 5.00.

4-(1-(3-(2-(2-oxoimidazolidin-1-yl)ethoxy)-4-(4-phenethylthiazol-2-yl)phenyl)-1H-1,2,3-triazol-4-yl)butanoic acid (12g). Methyl ester **11g** (150 mg, 0.27 mmol) dissolved in THF (4 mL) was treated with 1 mL of an aqueous solution of LiOH (23 mg, 0.54 mmol) for 3 h at room temperature. Change from colorless to yellow was observed after the addition of the base. The crude was quenched with aq HCl 1N to pH = 1 and the acidic solution was concentrated under reduced pressure. The resulting solid was filtered and washed with H_2O (3 x 5 mL) to give pure **12g** (136 mg, 93%) as a white solid. M.p.: 174.5 - 177.5 ^oC; ^1H NMR (DMSO-d_6, 400 MHz) δ (ppm): 8.47 (d, J = 8.5 Hz, 1H, Ar), 7.80 (s, 1H, Ar), 7.52 (d, J = 2.0 Hz, 1H, Ar), 7.28 (dd, J = 8.5, 2.0 Hz, 1H, Ar), 7.22 - 7.03 (m, 5H, Ar), 6.87 (s, 1H, Ar), 4.81 (br s, 1H, NHCON), 4.36 (t, J = 5.5 Hz, 2H, OCH_2), 3.72 (t, J = 5.5 Hz, 2H, $OCH_2C\underline{H_2}$), 3.61 (s, 3H, Me), 3.53 (dd, J = 9.0, 6.6 Hz, 2H, $C\underline{H_2}CH_2NHCON$), 3.33 (dd, J = 9.1, 6.6 Hz, 2H, $CH_2C\underline{H_2}NHCON$), 3.14 - 2.93 (m, 4H, $C\underline{H_2}C\underline{H_2}Ph$), 2.79 (t, J = 7.5 Hz, 2H, $TrizCH_2$), 2.37 (t, J = 7.4 Hz, 2H, $C\underline{H_2}COOMe$), 2.02 (quin, J = 7.5 Hz, 2H, $TrizCH_2C\underline{H_2}$); ^{13}C NMR (DMSO-d_6, 75 MHz) δ (ppm): 174.2 (COO), 162.2 (NHCON), 159.4 (OC_{Ar}), 155.6 (C_{Ar}), 156.4 (C_{Ar}), 147.7 (C_{Ar}), 141.4 (C_{Ar}), 138.0 (C_{Ar}), 129.0 (CH_{Ar}), 128.4 (CH_{Ar}), 128.3 (CH_{Ar}), 125.9 (CH_{Ar}), 121.3 (CH_{Ar}), 120.4 (C_{Ar}), 115.9 (CH_{Ar}), 112.0 (CH_{Ar}), 104.3 (CH_{Ar}), 67.6 (OCH_2), 45.3 ($\underline{C}H_2CH_2NHCON$), 42.4 ($OCH_2\underline{C}H_2$), 37.6 ($CH_2\underline{C}H_2NHCON$), 34.8 ($CH_2CH_2Ph$), 33.0 ($CH_2CH_2Ph$), 32.7 ($\underline{C}H_2COOH$), 24.5 ($TrizCH_2\underline{C}H_2$), 24.2 ($Triz\underline{C}H_2$); HRMS (ES, positive mode) m/z: calculated for $C_{29}H_{32}N_6O_4S$ 560.2206; Found 560.2200 (-1.11 ppm). HPLC (*Gradient A, Agilent*): R_t = 8.2 min; HRMS

(ES, negative mode) m/z: calculated for $C_{28}H_{30}N_6O_4S$ 546.2049; Found 546.2059 (1.81 ppm); Anal. Calc. for $C_{28}H_{30}N_6O_4S$: C. 61.52; H. 5.53; N. 15.37; S. 5.86; Found: C. 61.17; H. 5.60; N. 15.21; S. 5.81.

General procedure for N-Cbz deprotection. A solution of the corresponding Cbz-protected compound (1 eq) in a 1:1 mixture of THF/MeOH (20 mL) containing Pd/C (10%) (20% wt/wt) and TFA (0.5 – 1.5 mL), was hydrogenated at room temperature for 2 h, under atmospheric pressure using a balloon filled with hydrogen gas (3 cycles of vacuum + hydrogen). The Pd/C was filtered through Whatman PTFE filter paper, the solvent was removed under reduced pressure, and co-evaporated with mixtures of CH_2Cl_2/MeOH several times (5 x 10 mL). The residue was purified by HPFC on a SP1 Isolera Biotage using reverse phase columns (From 0% of CH_3CN to 100% of CH_3CN in 45 min) to give the final deprotected compounds as trifluoroacetate salts.

1-(2-(5-(4-(3-Ammoniumpropyl)-1H-1,2,3-triazol-1-yl)-2-(4-phenethylthiazol-2-yl)phenoxy)ethyl)imidazolidin-2-one 2,2,2 trifluoroacetate (12h). Following the general procedure of Cbz removal, **11h** (160 mg, 0.25 mmol), Pd/C 10 % (32 mg) and TFA (1.2 mL) were reacted. Work-up and purification gave **12h** (76 mg, 49%) as a colorless oil. ^1H NMR (CD_3OD, 400 MHz) δ (ppm): 8.47 (s, 1H, Ar), 8.46 (d, *J* = 8.6 Hz, 1H, Ar), 7.71 (d, *J* = 2.1 Hz, 1H, Ar), 7.59 (dd, *J* = 8.6, 2.1 Hz, 1H, Ar), 7.33 - 7.17 (m, 5H, Ar), 7.15 (s, 1H, Ar), 4.49 (t, *J* = 5.5 Hz, 2H, OCH_2), 3.75 (t, *J* = 5.5 Hz, 2H, $OCH_2C\underline{H_2}$), 3.60 (dd, *J* = 9.3, 6.9 Hz, 2H, $C\underline{H_2}CH_2NHCON$), 3.37 (dd, *J* = 9.3, 6.9 Hz, 2H, $CH_2C\underline{H_2}NHCON$), 3.18 - 3.03 (m, 6H, $C\underline{H_2}C\underline{H_2}Ph$, $C\underline{H_2}NH_3^+$), 2.93 (t, *J* = 7.4 Hz, 2H, $TrizCH_2$), 2.12 (quin, *J* = 7.6 Hz, 2H, $C\underline{H_2}CH_2NH_3^+$); ^{13}C NMR (CD_3OD, 75 MHz) δ (ppm): 165.1 (NHCON), 162.0 (OC_{Ar}), 157.5 (C_{Ar}), 157.1 (C_{Ar}), 148.4 (C_{Ar}), 142.8 (C_{Ar}), 139.6 (C_{Ar}), 130.8 (CH_{Ar}), 129.5 (CH_{Ar}), 129.4 (CH_{Ar}), 127.0 (CH_{Ar}), 123.9 (C_{Ar}), 121.8 (CH_{Ar}), 117.0 (CH_{Ar}), 113.6 (CH_{Ar}), 105.8 (CH_{Ar}), 68.5 (OCH_2), 47.0 ($\underline{C}H_2CH_2NHCON$), 43.8 ($OCH_2\underline{C}H_2$), 40.2 ($CH_2NH_3^+$), 39.3 ($CH_2\underline{C}H_2NHCON$), 36.7 ($CH_2CH_2Ph$), 34.3 ($CH_2CH_2Ph$), 28.2 ($\underline{C}H_2CH_2NH_3^+$), 23.2 ($Triz\underline{C}H_2$); HPLC (*Gradient A, Agilent*): R_t = 7.2 min; HRMS (ES, positive mode) m/z:

calculated for $C_{27}H_{31}N_7O_2S$ 517.2260; Found 517.2266 (1.17 ppm); Anal. Calc. for $C_{27}H_{31}N_7O_2S$.TFA: C. 55.14; H. 5.11; N. 15.52; S. 5.08; Found: C. 54.99; H. 5.24; N. 15.09; S. 5.00.

1-(2-(2-(4-(2-([1,1'-Biphenyl]-4-yl)ethyl)thiazol-2-yl)-5-(4-(3-ammoniumpropyl)-1H-1,2,3-triazol-1-yl)phenoxy)ethyl)imidazolidin-2-one 2,2,2 trifluoroacetate (12i). Following the general Cbz deprotection procedure, **11i** (107 mg, 0.15 mmol), Pd/C 10 % (21 mg) and TFA (1 mL) were reacted, to give **12i** (26 mg, 25%) as a colorless oil. ^{1}H NMR (DMSO-d$_6$, 400 MHz) δ (ppm): 8.77 (s, 1H, Ar), 8.49 (d, *J* = 8.6 Hz, 1H, Ar), 7.88 - 7.55 (m, 8H, Ar), 7.53 - 7.41 (m, 2H, Ar), 7.36 - 7.31 (m, 2H, Ar), 6.40 (br s, 1H, NHCON), 4.47 (t, *J* = 5.7 Hz, 2H, OCH_2), 3.64 (t, *J* = 5.6 Hz, 2H, $OCH_2C\underline{H_2}$), 3.49 (dd, *J* = 9.0, 6.7 Hz, 2H, $C\underline{H_2}CH_2$NHCON), 3.22 (dd, *J* = 9.0, 6.7 Hz, 2H, $CH_2C\underline{H_2}$NHCON), 3.17 - 3.03 (m, 4H, Thiaz$C\underline{H_2}C\underline{H_2}$), 2.93 (t, *J* = 7.5, 2H, $C\underline{H_2}NH_3^+$), 2.83 (t, J = 8.5 Hz, 2H, TrizCH_2), 1.98 (quin, *J* = 7.6 Hz, 2H, $C\underline{H_2}CH_2NH_3^+$); ^{13}C NMR (DMSO-d$_6$, 75 MHz) δ (ppm): 162.2 (NHCON), 159.4 (OC_{Ar}), 155.6 (C_{Ar}), 155.4 (C_{Ar}), 147.0 (C_{Ar}), 140.7 (C_{Ar}), 140.0 (C_{Ar}), 137.9 (C_{Ar}), 137.8 (C_{Ar}), 129.0 (CH_{Ar}), 128.9 (CH_{Ar}), 128.9 (CH_{Ar}), 127.2 (CH_{Ar}), 126.6 (CH_{Ar}), 126.5 (CH_{Ar}), 121.5 (C_{Ar}), 120.6 (CH_{Ar}), 116.1 (CH_{Ar}), 112.1 (CH_{Ar}), 104.5 (CH_{Ar}), 67.6 (OCH_2), 45.4 ($\underline{C}H_2CH_2$NHCON), 42.4 ($OCH_2\underline{C}H_2$), 37.6 ($CH_2NH_3^+$), 37.5 ($CH_2\underline{C}H_2$NHCON), 34.3 (ThiazCH_2CH_2), 32.6 (ThiazCH_2CH_2), 26.7 ($\underline{C}H_2CH_2NH_3^+$), 22.0 (Triz$\underline{C}H_2$); HPLC (*Gradient A, Agilent*): R_t = 8.4 min; HRMS (ES, positive mode) m/z: calculated for $C_{33}H_{35}N_7O_2S$ 593.2573; Found 593.2572 (-0.09 ppm); Anal. Calc. for $C_{33}H_{35}N_7O_2S$.TFA: C. 59.40; H. 5.13; N. 13.85; S. 4.53; Found: C. 59.67; H. 5.41; N. 13.66; S. 4.25.

4-(1-(3-Methoxy-4-(4-phenethylthiazol-2-yl)phenyl)-1H-1,2,3-triazol-4-yl)butan-1-ammonium 2,2,2 trifluoroacetate (12j). A solution of **11j** (60 mg, 0.11 mmol) in CH_2Cl_2 (7 mL) was treated with Pd/C 10 % (20 mg) and TFA (1 mL) according to the general Cbz deprotection procedure. Work-up and purification afforded **12j** (20 mg, 36%) as a

colorless oil. ^{1}H NMR (CD_3OD, 400 MHz) δ (ppm): 8.45 (s, 1H, Ar), 8.45 (d, *J* = 8.3 Hz, 1H, Ar), 7.67 (d, *J* = 2.1 Hz, 1H, Ar), 7.55 (dd, *J* = 8.6, 2.1 Hz, 1H, Ar), 7.36 - 7.16 (m, 5H, Ar), 7.15 (s, 1H, Ar), 4.12 (s, 3H, OCH_3), 3.20 - 3.02 (m, 4H, $C\underline{H_2}C\underline{H_2}Ph$), 3.00 (t, *J* = 7.5 Hz, 2H, $C\underline{H_2}NH_3^+$), 2.87 (t, *J* = 7.3 Hz, 2H, $TrizCH_2$), 1.95 - 1.85 (m, 2H, $TrizCH_2C\underline{H_2}$), 1.84 - 1.75 (m, 2H, $C\underline{H_2}CH_2NH_3^+$); ^{13}C NMR ($CD_3OD$, 100 MHz) δ (ppm): 162.1 (OC_{Ar}), 158.6 (C_{Ar}), 156.8 (C_{Ar}), 149.4 (C_{Ar}), 142.8 (C_{Ar}), 139.8 (C_{Ar}), 130.5 (CH_{Ar}), 129.5 (CH_{Ar}), 129.4 (CH_{Ar}), 127.0 (CH_{Ar}), 123.8 (C_{Ar}), 121.6 (CH_{Ar}), 116.9 (CH_{Ar}), 113.2 (CH_{Ar}), 104.8 (CH_{Ar}), 56.7 (OCH_3), 40.4 ($CH_2NH_3^+$), 36.7 (CH_2CH_2Ph), 34.2 (CH_2CH_2Ph), 28.0 ($\underline{C}H_2CH_2NH_3^+$), 27.1 ($TrizCH_2\underline{C}H_2$), 25.6 ($Triz\underline{C}H_2$); HPLC (*Gradient A, Agilent*): R_t = 8.1 min; HRMS (ES, positive mode) m/z: calculated for $C_{24}H_{27}N_5OS$ 433.1936; Found 433.1931 (-1.25 ppm); Anal. Calc. for $C_{24}H_{27}N_5OS$.TFA: C. 57.03; H. 5.15; N. 12.79; S. 5.85; Found: C. 57.25; H. 5.31; N. 12.61; S. 5.39.

4-(1-(3-Methoxy-4-(4-(2-([1,1'-Biphenyl]-4-yl)ethyl)thiazol-2-yl)phenyl)-1H-1,2,3-triazol-4-yl)butan-1-ammonium 2,2,2 trifluoroacetate (12k). Following the general Cbz deprotection procedure, **11k** (120 mg, 0.19 mmol), Pd/C 10 % (24 mg) and TFA (1 mL) were reacted. Work-up and purification yielded **12k** (42 mg, 36%) as a colorless oil. ^{1}H NMR (DMSO-d_6, 400 MHz) δ (ppm): 8.76 (s, 1H, Ar), 8.48 (d, *J* = 8.6 Hz, 1H, Ar), 7.82 - 7.70 (m, 3H, NH_3^+), 7.73 (d, *J* = 2.1 Hz, 1H, Ar), 7.68 - 7.61 (m, 3H, Ar), 7.58 (d, *J* = 8.2 Hz, 2H, Ar), 7.48 - 7.41 (m, 3H, Ar), 7.38 - 7.27 (m, 3H, Ar), 4.13 (s, 3H, OCH_3), 3.20 - 3.05 (m, 4H, $ThiazC\underline{H_2}C\underline{H_2}$), 2.85 (t, *J* = 7.5 Hz, 2H, $C\underline{H_2}NH_3^+$), 2.77 (t, *J* = 7.3 Hz, 2H, $TrizCH_2$), 1.75 (quin, *J* = 7.2 Hz, 2H, $TrizCH_2C\underline{H_2}$), 1.63 (quin, *J* = 7.2 Hz, 2H, $C\underline{H_2}CH_2NH_3^+$); ^{13}C NMR (DMSO-$d_6$, 100 MHz) δ (ppm): 159.3 (OC_{Ar}), 156.6 (C_{Ar}), 155.3 (C_{Ar}), 147.7 (C_{Ar}), 140.7 (C_{Ar}), 140.0 (C_{Ar}), 138.0 (C_{Ar}), 137.8 (C_{Ar}), 129.0 (CH_{Ar}), 128.9 (CH_{Ar}), 126.6 (CH_{Ar}), 126.5 (CH_{Ar}), 121.2 (CH_{Ar}), 120.5 (CH_{Ar}), 120.4 (CH_{Ar}), 118.8 (C_{Ar}), 116.0 (CH_{Ar}), 111.9 (CH_{Ar}), 103.7 (CH_{Ar}), 56.5 (OCH_3), 38.6 ($CH_2NH_3^+$), 34.3 ($Thiaz\underline{C}H_2CH_2$), 32.6 ($ThiazCH_2\underline{C}H_2$), 26.5 ($\underline{C}H_2CH_2NH_3^+$), 25.6 ($TrizCH_2\underline{C}H_2$), 24.4 ($Triz\underline{C}H_2$), HPLC (*Gradient A, Agilent*): R_t = 9.6 min; HRMS (ES, positive mode) m/z:

calculated for $C_{30}H_{31}N_5OS$ 509.2249; Found 509.2256 (1.37 ppm); Anal. Calc. for $C_{30}H_{31}N_5OS$.TFA: C. 61.62; H. 5.17; N. 11.23; S. 5.14; Found: C. 61.26; H. 5.08; N. 11.22; S. 5.13.

1-(2-(5-(4-(4-Ammoniobutyl)-1H-1,2,3-triazol-1-yl)-2-(4-phenylthiazol-2-yl)phenoxy) ethyl)imidazolidin-2-one 2,2,2 trifluoroacetate (12n). According to the general Cbz deprotection procedure, **11n** (90 mg, 0.14 mmol), Pd/C 10 % (18 mg) and TFA (1 mL) were reacted. After work-up and purification compound **12n** (21 mg, 26%) was obtained as a colorless oil. ^1H NMR (CD_3OD, 400 MHz) δ (ppm): 8.64 (d, *J* = 8.6 Hz, 1H, Ar), 8.46 (s, 1H, Ar), 8.54 (dd, *J* = 8.2, 1.3 Hz, 2H, Ar), 7.81 (s, 1H, Ar), 7.71 (d, *J* = 2.0 Hz, 1H, Ar), 7.61 (dd, *J* = 8.6, 2.0 Hz, 1H, Ar), 7.45 (dd, *J* = 8.3, 7.0 Hz, 2H, Ar), 7.36 (d, *J* = 7.4 Hz, 1H, Ar), 4.51 (t, *J* = 5.5 Hz, 2H, OCH_2), 3.78 (t, *J* = 5.5 Hz, 2H, $OCH_2\underline{CH_2}$), 3.64 (dd, *J* = 9.3, 6.9 Hz, 2H, $\underline{CH_2}CH_2$NHCON), 3.39 (dd, *J* = 9.2, 6.9 Hz, 2H, $CH_2\underline{CH_2}$NHCON), 3.03 (t, *J* = 7.4 Hz, 2H, $\underline{CH_2}NH_3^+$), 2.87 (t, *J* = 7.2 Hz, 2H, TrizCH_2), 1.92 - 1.83 (m, 2H, Triz$CH_2\underline{CH_2}$), 1.83 - 1.73 (m, 2H, $\underline{CH_2}CH_2NH_3^+$); ^{13}C NMR (CD_3OD, 100 MHz) δ (ppm): 165.1 (NHCON), 162.0 (OC_{Ar}), 157.5 (C_{Ar}), 155.8 (C_{Ar}), 149.3 (C_{Ar}), 139.7 (C_{Ar}), 135.9 (C_{Ar}), 131.0 (CH_{Ar}), 129.8 (CH_{Ar}), 129.2 (CH_{Ar}), 127.4 (CH_{Ar}), 123.9 (C_{Ar}), 121.6 (CH_{Ar}), 116.0 (CH_{Ar}), 113.6 (CH_{Ar}), 105.6 (CH_{Ar}), 68.4 (OCH_2), 47.0 ($\underline{C}H_2CH_2$NHCON), 43.9 ($OCH_2\underline{C}H_2$), 40.4 ($CH_2NH_3^+$), 39.3 ($CH_2\underline{C}H_2$NHCON), 28.0 ($\underline{C}H_2CH_2NH_3^+$), 27.1 (Triz$CH_2\underline{C}H_2$), 25.6 (Triz$\underline{C}H_2$); HPLC (*Gradient A, Agilent*): R_t = 7.5 min; HRMS (ES, positive mode) m/z: calculated for $C_{26}H_{29}N_7O_2S$ 503.2103; Found 503.2100 (-0.66 ppm); Anal. Calc. for $C_{26}H_{29}N_7O_2S$.TFA: C. 54.45; H. 4.90; N. 15.87; S. 5.19; Found: C. 54.66; H. 4.48; N. 15.90; S. 5.15.

1-(2-(5-(4-(4-Ammoniumbutyl)-1H-1,2,3-triazol-1-yl)-2-(4-(3-phenylpropyl)thiazol-2-yl)phenoxy)ethyl)imidazolidin-2-one 2,2,2 trifluoroacetate (12p).The general Cbz deprotection procedure was followed with **11p** (160 mg, 0.24 mmol), Pd/C 10 % (32 mg) and TFA (1.3 mL) to give, after work-up and purification, compound **12p** (16 mg, 11%) as a colorless oil. ^1H NMR (CD_3OD, 400 MHz) δ (ppm): 8.50 (s, 1H, Ar), 8.48 (d, *J* = 8.5 Hz,

1H, Ar), 7.71 (d, *J* = 2.1 Hz, 1H, Ar), 7.58 (dd, *J* = 8.6, 2.0 Hz, 1H, Ar), 7.38 - 7.06 (m, 6H, Ar), 4.49 (t, *J* = 5.6 Hz, 2H, OCH_2), 3.75 (t, *J* = 5.5 Hz, 2H, $OCH_2C\underline{H_2}$), 3.61 (dd, *J* = 9.3, 6.9 Hz, 2H, $C\underline{H_2}CH_2NHCON$), 3.37 (dd, *J* = 9.3, 6.9 Hz, 2H, $CH_2C\underline{H_2}NHCON$), 3.00 (t, *J* = 7.5 Hz, 2H, $C\underline{H_2}NH_3^+$), 2.93 - 2.84 (m, *J* = 7.0 Hz, 4H, $ThiazCH_2$, CH_2Ph), 2.71 (t, *J* = 7.6 Hz, 2H, $TrizCH_2$), 2.09 (quin, *J* = 7.6 Hz, 2H, $ThiazCH_2C\underline{H_2}$), 1.99 - 1.85 (m, 2H, $TrizCH_2C\underline{H_2}$), 1.78 - 1.72 (m, 2H, $C\underline{H_2}CH_2NH_3^+$); ^{13}C NMR (CD_3OD, 75 MHz) δ (ppm): 165.1 (NHCON), 162.0 (OC_{Ar}), 157.8 (C_{Ar}), 157.5 (C_{Ar}), 149.3 (C_{Ar}), 143.4 (C_{Ar}), 139.6 (C_{Ar}), 130.8 (CH_{Ar}), 129.5 (CH_{Ar}), 129.4 (CH_{Ar}), 126.8 (CH_{Ar}), 123.9 (C_{Ar}), 121.6 (CH_{Ar}), 116.6 (CH_{Ar}), 113.6 (CH_{Ar}), 105.7 (CH_{Ar}), 68.5 (OCH_2), 46.9 ($\underline{C}H_2CH_2NHCON$), 43.9 ($OCH_2\underline{C}H_2$), 40.4 ($CH_2NH_3^+$), 39.3 ($CH_2\underline{C}H_2NHCON$), 36.4 ($CH_2CH_2CH_2Ph$), 32.4 ($CH_2CH_2CH_2Ph$), 31.7 ($CH_2CH_2CH_2Ph$), 28.0 ($\underline{C}H_2CH_2NH_3^+$), 27.1 ($TrizCH_2\underline{C}H_2$), 25.6 ($Triz\underline{C}H_2$); HPLC (*Gradient A, Agilent*): R_t = 7.7 min; HRMS (ES, positive mode) m/z: calculated for $C_{29}H_{35}N_7O_2S$ 545.2573; Found 545.2573 (0.03 ppm); Anal. Calc. for $C_{29}H_{35}N_7O_2S$.TFA: C. 56.44; H. 5.50; N. 14.86; S. 4.86; Found: C. 56.04; H. 5.66; N. 14.59; S. 4.48.

1-(2-(5-(4-(4-Ammoniumbutyl)-1H-1,2,3-triazol-1-yl)-2-(4-(2-(3,4-dihydro-2H-1 λ ²-quinolin-5-yl)ethyl)thiazol-2-yl)phenoxy)ethyl)imidazolidin-2-one 2,2,2 trifluoroacetate (12q). Following the general Cbz deprotection procedure, **11q** (115 mg, 0.16 mmol) was hydrogenated to give **12q** (24 mg, 15%) as a colorless oil. ^1H NMR (CD_3OD, 400 MHz) δ (ppm): 8.47 (s, 1H, Ar), 8.46 (d, *J* = 8.9 Hz, 1H, Ar), 7.71 (d, *J* = 2.1 Hz, 1H, Ar), 7.59 (dd, *J* = 8.6, 2.0 Hz, 1H, Ar), 7.15 (s, 1H, Ar), 6.93 - 6.82 (m, 2H, Ar), 6.63 (d, *J* = 8.7 Hz, 1H, Ar), 4.49 (t, *J* = 5.5 Hz, 2H, OCH_2), 3.75 (t, *J* = 5.5 Hz, 2H, $OCH_2C\underline{H_2}$), 3.61 (dd, *J* = 9.3, 6.9 Hz, 2H, $C\underline{H_2}CH_2NHCON$), 3.37 (dd, *J* = 9.3, 6.9 Hz, 2H, $CH_2C\underline{H_2}NHCON$), 3.27 (t, *J* = 5.5 Hz, 2H, $PhNHC\underline{H_2}$), 3.08 (t, *J* = 7.5 Hz, 2H, $ThiazC\underline{H_2}CH_2$), 3.03 - 2.92 (m, 4H, $ThiazCH_2C\underline{H_2}$, $C\underline{H_2}NH_3^+$), 2.88 (t, *J* = 7.2 Hz, 2H, $TrizCH_2$), 2.75 (m, *J* = 6.5 Hz, 2H, $PhNHCH_2CH_2C\underline{H_2}$), 1.95 (quin, *J* = 6.3 Hz, 2H, $PhNHCH_2C\underline{H_2}$), 1.89 - 1.72 (m, 4H, $TrizCH_2C\underline{H_2}$, $C\underline{H_2}CH_2NH_3^+$); ^{13}C NMR (CD_3OD, 100

MHz) δ (ppm): 165.1 (NHCON), 161.0 (OC_{Ar}), 157.5 (C_{Ar}), 157.3 (C_{Ar}), 149.3 (C_{Ar}), 141.5 (C_{Ar}), 139.7 (C_{Ar}), 134.9 (C_{Ar}), 130.8 (CH_{Ar}), 130.8 (CH_{Ar}), 128.0 (CH_{Ar}), 125.6 (C_{Ar}), 123.8 (C_{Ar}), 121.6 (CH_{Ar}), 118.2 (CH_{Ar}), 116.9 (CH_{Ar}), 113.6 (CH_{Ar}), 105.7 (CH_{Ar}), 68.4 (OCH_2), 46.9 ($\underline{C}H_2CH_2NHCON$), 43.8 ($OCH_2\underline{C}H_2$), 43.8 ($PhNH\underline{C}H_2$), 40.4 ($\underline{C}H_2NH_3^+$), 39.3 ($CH_2\underline{C}H_2NHCON$), 36.0 ($ThiazCH_2\underline{C}H_2$), 34.5 ($Thiaz\underline{C}H_2CH_2$), 28.0 ($\underline{C}H_2CH_2NH_3^+$), 27.5 ($PhNHCH_2CH_2\underline{C}H_2$), 27.1 ($TrizCH_2\underline{C}H_2$), 25.6 ($Triz\underline{C}H_2$), 22.8 ($PhNHCH_2\underline{C}H_2$); HPLC: *(Gradient from 2% of CH_3CN to 30% of CH_3CN, Agilent)*, R_t = 1.4 min; HRMS (ES, positive mode) m/z: calculated for $C_{31}H_{38}N_8O_2S$ 586.2838; Found 586.2859 (3.60 ppm); Anal. Calc. for $C_{31}H_{38}N_8O_2S$.2TFA: C. 51.59; H. 4.95; N. 13.75; S. 3.93; Found: C. 51.11; H. 4.86; N. 13.26; S. 3.56.

1-(2-(5-(4-(4-Ammoniumbutyl)-1H-1,2,3-triazol-1-yl)-2-(4-(2-(2,3-dihydrobenzofuran-6-yl)ethyl)thiazol-2-yl)phenoxy)ethyl)imidazolidin-2-one 2,2,2 trifluoroacetate (12r). Following the general procedure of Cbz deprotection, **11r** (106 mg, 0.15 mmol) was hydrogenated with Pd/C 10 % (21 mg) and TFA (1 mL). Work-up and purification yielded **12r** (43 mg, 42%) as a colorless oil. ^1H NMR (D_2O, 400 MHz, 90 °C) δ (ppm): 8.58 (d, J = 8.5 Hz, 1H, Ar), 8.55 (s, 1H, Ar), 7.84 (d, J = 2.0 Hz, 1H, Ar), 7.67 (dd, J = 8.6, 2.0 Hz, 1H, Ar), 7.35 (d, J = 1.9 Hz, 1H, Ar), 7.30 (s, 1H, Ar), 7.25 (dd, J = 8.1, 1.9 Hz, 1H, Ar), 7.06 (d, J = 8.0 Hz, 1H, Ar), 4.40 (t, J = 5.5 Hz, 2H, $OC\underline{H_2}CH_2N$), 4.17 (t, J = 8.7 Hz, 2H, $OC\underline{H_2}CH_2Ph$), 4.06 (t, J = 5.5 Hz, 2H, $OCH_2C\underline{H_2}N$), 3.92 (dd, J = 9.7, 6.8 Hz, 2H, $C\underline{H_2}CH_2NHCON$), 3.78 (dd, J = 9.5, 6.8 Hz, 2H, $CH_2C\underline{H_2}NHCON$), 3.67 (t, J = 8.7 Hz, $OCH_2C\underline{H_2}Ph$), 3.48 - 3.21 (m, 8H, $ThiazC\underline{H_2}C\underline{H_2}$, $C\underline{H_2}NH_3^+$, $TrizCH_2$), 2.38 - 2.26 (m, 2H, $TrizCH_2C\underline{H_2}$, $C\underline{H_2}CH_2NH_3^+$); ^{13}C NMR (D_2O, 100 MHz, 90 °C) δ (ppm): 164.5 (NHCON), 160.7 (OC_{Ar}), 158.2 (OC_{Ar}), 156.3 (C_{Ar}), 156.1 (C_{Ar}), 149.1 (C_{Ar}), 138.1 (C_{Ar}), 134.2 (C_{Ar}), 129.7 (CH_{Ar}), 127.9 (CH_{Ar}), 127.8 (CH_{Ar}), 125.3 (CH_{Ar}), 122.7 (CH_{Ar}), 120.8 (CH_{Ar}), 115.7 (C_{Ar}), 112.9 (C_{Ar}), 109.1 (CH_{Ar}), 105.2 (CH_{Ar}), 71.7 ($O\underline{C}H_2CH_2Ph$), 68.4 ($O\underline{C}H_2CH_2N$), 46.3 ($\underline{C}H_2CH_2NHCON$), 43.2 ($OCH_2\underline{C}H_2N$), 39.9 ($CH_2NH_3^+$), 38.4 ($CH_2\underline{C}H_2NHCON$), 34.7 ($ThiazCH_2CH_2$), 33.3 ($ThiazCH_2CH_2$), 29.8 ($OCH_2\underline{C}H_2Ph$), 27.0 ($\underline{C}H_2CH_2NH_3^+$), 25.9

(TrizCH$_2\underline{C}$H$_2$), 24.7 (Triz$\underline{C}$H$_2$); HPLC (*Gradient A, Agilent*): R_t = 7.2 min; HRMS (ES, positive mode) m/z: calculated for $C_{30}H_{35}N_7O_3S$ 573.2522; Found 573.2520 (-0.43 ppm); Anal. Calc. for $C_{30}H_{35}N_7O_3S$.TFA: C. 55.89; H. 5.28; N. 14.26; S. 4.66; Found: C. 54.96; H. 5.72; N. 13.67; S. 4.89.

1-(2-(5-(4-(4-Ammoniumbutyl)-1H-1,2,3-triazol-1-yl)-2-(4-(2-(dibenzo[b,d]furan-1-yl)ethyl)thiazol-2-yl)phenoxy)ethyl)imidazolidin-2-one 2,2,2 trifluoroacetate (12s). Following the general procedure of hydrogenation, **11s** (176 mg, 0.23 mmol) was treated with Pd/C 10 % (35 mg) and TFA (1.2 mL) to give, after the work-up and purification, compound **12s** (19 mg, 13%) as a colorless oil. ^{1}H NMR (DMSO-d$_6$, 400 MHz) δ (ppm): 8.66 (s, 1H, Ar), 8.48 (d, *J* = 8.6 Hz, 1H, Ar), 8.09 (dd, *J* = 7.7, 1.4 Hz, 1H, Ar), 8.03 (d, *J* = 1.8 Hz, 1H, Ar), 7.80 - 7.30 (m, 11H, Ar, NH_3^+), 6.18 (br s, 1H, NHCON), 4.48 (t, *J* = 5.8 Hz, 2H, OCH$_2$), 3.64 (t, *J* = 5.7 Hz, 2H, OCH$_2$C$\underline{H_2}$), 3.49 (dd, *J* = 8.9, 6.8 Hz, 2H, C$\underline{H_2}$CH$_2$NHCON), 3.27 - 3.20 (m, 4H, ThiazCH$_2$CH$_2$), 2.87 (t, *J* = 7.4 Hz, 2H, C$\underline{H_2}$NH$_3^+$), 2.79 (t, *J* = 7.2 Hz, 2H, TrizCH$_2$), 1.78 (quin, *J* = 7.4 Hz, 2H, TrizCH$_2$C$\underline{H_2}$), 1.68 (quin, *J* = 7.6 Hz, 2H, C$\underline{H_2}$CH$_2$NH$_3^+$); ^{13}C NMR (CDCl$_3$, 75 MHz) δ (ppm): 162.1 (NHCON), 159.4 (OC$_{Ar}$), 155.7 (OC$_{Ar}$), 155.6 (OC$_{Ar}$), 155.3, (NHCOO), 154.0 (C$_{Ar}$), 147.7 (C$_{Ar}$), 137.9 (C$_{Ar}$), 136.3 (C$_{Ar}$), 129.1 (CH$_{Ar}$), 128.0 (CH$_{Ar}$), 127.4 (CH$_{Ar}$), 123.5 (C$_{Ar}$), 123.0 (CH$_{Ar}$), 121.4 (C$_{Ar}$), 121.0 (CH$_{Ar}$), 120.5 (CH$_{Ar}$), 120.4 (CH$_{Ar}$), 116.1 (CH$_{Ar}$), 112.0 (CH$_{Ar}$), 111.6 (CH$_{Ar}$), 111.2 (CH$_{Ar}$), 104.3 (CH$_{Ar}$), 67.6 (OCH$_2$), 45.3 ($\underline{C}$H$_2$CH$_2$NHCON), 42.4 (OCH$_2\underline{C}$H$_2$), 38.7 (CH$_2$NH$_3^+$), 37.5 (CH$_2\underline{C}$H$_2$NHCON), 34.7 (ThiazCH$_2$CH$_2$), 33.2 (ThiazCH$_2$CH$_2$), 26.5 ($\underline{C}$H$_2$CH$_2$NH$_3^+$), 25.5 (TrizCH$_2\underline{C}$H$_2$), 24.4 (Triz$\underline{C}$H$_2$); HPLC (*Gradient A, Waters*): R_t = 7.0 min; HRMS (ES, positive mode) m/z: calculated for $C_{34}H_{35}N_7O_3S$ 621.2522; Found 621.2517 (-0.78 ppm); Anal. Calc. for $C_{34}H_{35}N_7O_3S$.TFA: C. 58.77; H. 4.93; N. 13.33; S. 4.36; Found: C. 59.15; H. 5.09; N. 13.46; S. 4.21

Synthesis of truncated analogues 13 and 14

4-Bromo-2-(2-(2-oxoimidazolidin-1-yl)ethoxy)benzothioamide (A).

Following the general procedure for the synthesis of thioamides (**8a-g**), the bromobenzonitrile **4a**[1] (1.15 g, 3.71 mmol) dissolved in DMF (25 mL) was treated with a solution of $(NH_4)_2S$ 20% aq (17.6 mL, 0.26 mol). After the work-up, the crude was purified by flash column chromatography (CH_2Cl_2/MeOH, 100:2) to give thioamide **A** (845 mg, 69%) as a yellow solid. M.p.: Decompose without melting; ^1H NMR (DMSO-d_6, 400 MHz) δ (ppm): 10.03 (br s, 1H, $SCNH_2$), 9.35 (br s, 1H, $SCNH_2$), 8.64 (d, *J* = 8.3 Hz, 1H, Ar), 7.31 (d, *J* = 1.9 Hz, 1H, Ar), 7.17 (dd, *J* = 8.4, 1.8 Hz, 1H, Ar), 6.35 (br s, 1H, NHCON), 4.16 (t, *J* = 5.2 Hz, 2H, OCH_2), 3.47 (dd, *J* = 9.0, 6.7 Hz, 2H, $C\underline{H_2}CH_2$NHCON), 3.42 (t, *J* = 5.1 Hz, 2H, $OCH_2C\underline{H_2}$), 3.22 (t, *J* = 7.9 Hz, 2H, $CH_2C\underline{H_2}$NHCON); ^{13}C NMR (DMSO-d_6, 75 MHz) δ (ppm): 197.6 ($SCNH_2$), 162.3 (NHCOO), 154.1 (OC_{Ar}), 132.6 (CH_{Ar}), 129.7 (C_{Ar}), 123.9 (CH_{Ar}), 123.2 (CH_{Ar}), 115.6 (C_{Ar}), 67.6 (OCH_2), 45.6 ($\underline{C}H_2CH_2$NHCON), 42.6 ($OCH_2\underline{C}H_2$), 37.6 ($CH_2\underline{C}H_2$NHCON); MS (ESI, positive mode) m/z: 346.0 $[M+H]^+$, with a Br isotopic pattern.

1-(2-(2-(4-Phenethylthiazol-2-yl)-5-bromophenoxy)ethyl)imidazolidin-2-one (13).

Following the general Hantzsch procedure, thioamide **A** (200 mg, 0.58 mmol) and commercially available 1-bromo-4-phenylbutan-2-one (132 mg, 0.58 mmol) were reacted for 4 h. After the work-up, the final residue was purified by CCTLC on the Chromatotron (CH_2Cl_2/MeOH, 98:2) to yield **13** (241 mg, 85%) as a white solid. M.p.: 160-162 °C; ^1H NMR ($CDCl_3$, 400 MHz) δ (ppm): 8.20 (d, *J* = 8.4 Hz, 1H, Ar), 7.36 - 6.99 (m, 7H, Ar), 6.83 (s, 1H, Ar), 4.64 (br s, 1H, NHCON), 4.25 (t, *J* = 5.3 Hz, 2H, OCH_2), 3.69 (t, *J* = 5.3 Hz, 2H, $OCH_2C\underline{H_2}$), 3.52 (dd, *J* = 9.0, 6.7 Hz, 2H, $C\underline{H_2}CH_2$NHCON), 3.31 (dd, *J* = 9.1, 6.6 Hz, 2H, $CH_2C\underline{H_2}$NHCON), 3.11 - 2.97 (m, 4H, ThiazCH_2CH_2); ^{13}C NMR ($CDCl_3$, 75 MHz) δ (ppm): 162.7 (NHCON), 160.6 (OC_{Ar}), 155.9 (C_{Ar}), 155.7 (C_{Ar}), 141.7 (C_{Ar}), 130.0 (CH_{Ar}), 128.6 (CH_{Ar}), 128.5 (CH_{Ar}), 126.1 (CH_{Ar}), 124.8 (CH_{Ar}), 124.0 (C_{Ar}), 121.9 (C_{Ar}), 115.9

(CH_{Ar}), 114.9 (CH_{Ar}), 68.5 (OCH_2), 46.7 ($\underline{C}H_2CH_2NHCON$), 43.3 ($OCH_2\underline{C}H_2$), 38.5 ($CH_2\underline{C}H_2NHCON$), 35.7 ($ThiazCH_2CH_2$), 33.5 ($ThiazCH_2CH_2$); HPLC (*Gradient A, Agilent*): R_t = 9.9 min; HRMS (ES, positive mode) m/z: calculated for $C_{22}H_{22}BrN_3O_2S$ 471.0616; Found 471.0622 (1.32 ppm); Anal. Calc. for $C_{22}H_{22}BrN_3O_2S$: C. 55.94; H. 4.69; N. 8.90; S. 6.79; Found: C. 55.77; H. 4.73; N. 8.71; S. 6.62.

4-(4-(4-Ammoniobutyl)-1H-1,2,3-triazol-1-yl)-2-(2-(2-oxoimidazolidin-1-yl)ethoxy) benzonitrile bis(2,2,2 trifluoroacetate) (14). A solution of **7a** (5 mg, 0.01 mmol) in CH_2Cl_2 (3 mL) was treated with TFA (0.3 mL) for 2 h at room temperature. Volatiles were removed and the crude was co-evaporated several times with CH_2Cl_2/MeOH (5 x 10 mL). The final residue was purified HPFC on a SP1 Isolera Biotage using reverse phase columns (From 0% of CH_3CN to 100% of CH_3CN in 45 min) to yield **14** (3 mg, 60%) as a colorless oil. ^1H NMR (DMSO-d_6, 500 MHz) δ (ppm): 8.80 (s, 1H, Ar), 7.97 (d, J = 8.4 Hz, 1H, Ar), 7.76 (d, J = 1.9 Hz, 1H, Ar), 7.67 (dd, J = 8.5, 1.9 Hz, 1H, Ar), 7.40 - 6.65 (m, 3H, NH_3^+), 6.42 (br s, 1H, NHCON), 4.37 (t, J = 5.3 Hz, 2H, OCH_2), 3.55 (dd, J = 8.9, 6.8 Hz, 2H, $CH_2C\underline{H_2}NHCON$), 3.50 (t, J = 5.3 Hz, 2H, $OCH_2C\underline{H_2}$), 3.25 (t, J = 7.9 Hz, 2H, $C\underline{H_2}CH_2NHCON$), 2.82 - 2.72 (m, 4H, $C\underline{H_2}NH_3^+$, $TrizCH_2$), 1.72 (quin, J = 7.5 Hz, 2H, $TrizCH_2C\underline{H_2}$), 1.57 (quin, J = 7.8 Hz, 2H, $C\underline{H_2}CH_2NH_3^+$); ^{13}C NMR (DMSO-d_6, 125 MHz) δ (ppm): 162.1 (NHCON), 161.1 (OC_{Ar}), 148.1 (C_{Ar}), 141.1 (C_{Ar}), 135.4 (CH_{Ar}), 120.6 (CH_{Ar}), 115.7 (CN), 111.9 (CH_{Ar}), 104.1 (CH_{Ar}), 100.0 (C_{Ar}), 68.8 (OCH_2), 46.0 ($\underline{C}H_2CH_2NHCON$), 42.4 ($OCH_2\underline{C}H_2$), 40.0 ($\underline{C}H_2NH_3^+$), 37.6 ($CH_2\underline{C}H_2NHCON$), 27.8 ($\underline{C}H_2CH_2NH_3^+$), 25.6 ($TrizCH_2\underline{C}H_2$), 24.5 ($Triz\underline{C}H_2$); HPLC (*Gradient A, Agilent*): R_t = 4.8 min; HRMS (ES, positive mode) m/z: calculated for $C_{18}H_{23}N_7O_2$ 369.1913; Found 369.1904 (-2.65 ppm); Anal. Calc. for $C_{20}H_{24}F_3N_7O_4$: C. 49.69; H. 5.00; N. 20.28; Found: C. 49.90; H. 4.68; N. 19.84.

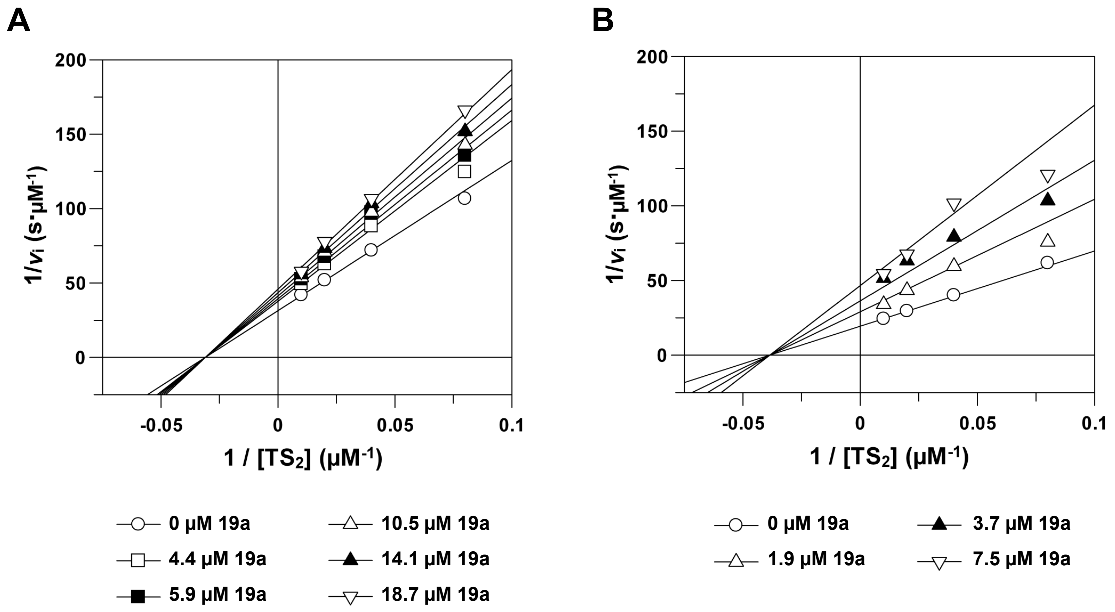

Figure S1. Noncompetitive hyperbolic inhibition of *Li*TryR by **19a**. Initial velocities were used for determination of the K_i value and assessment of the inhibition modality of **19a** by two different methods: DTNB-coupled assay (absorbance readings at 412 nm) (A) and trypanothione-dependent NADPH oxidation assay (absorbance readings at 340 nm) (B). Lineweaver–Burk plots of reciprocal initial velocities (v_i) versus reciprocals of four TS_2 concentrations (12.5, 25, 50 and 100 µM) are shown. Experimental conditions in both experiments were similar to those described in the experimental section except that for the NADPH oxidation assay NADPH concentration was raised to 500 µM, $NADP^+$ to 100 µM and 4.8 mM of oxidized glutathione was added. Data were fitted using the hyperbolic noncompetitive-mode equation described by Leskovac:

$$1/v = 1/V_{max} \times (\alpha K_i + [I] / \alpha K_i + \beta[I]) + \alpha K_m/V_{max} \times (K_i + [I] / \alpha K_i + \beta[I]) \times 1/[S].$$[2]

Results of the fits are shown in Table S1.

Table S1. Estimation of β and K_i in the noncompetitive hyperbolic inhibition mechanism by **19a**.

Parameters	DTNB-coupled assay	NADPH oxidation assay
K_i (µM)$^{\&}$	12.7 ± 3.5	2.5 ± 0.7
β$^{\&}$	0.5 ± 0.1	0.2 ± 0.1

$^{\&}$ Estimated values ± standard error of K_i and β were obtained by fitting the v_i values for every **19a** concentration at the different TS_2 concentrations using an alpha value of 1.

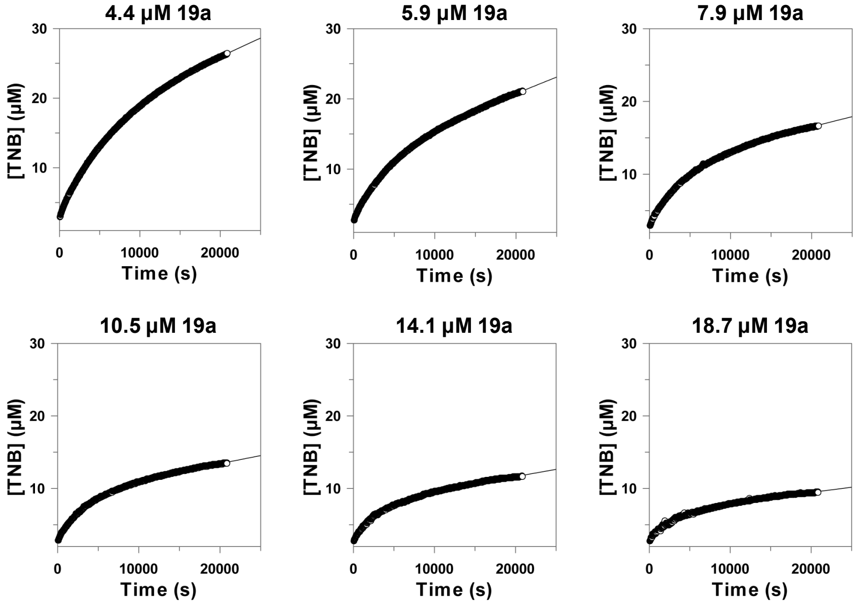

Figure S2. **19a** concentration dependence of the observed rate constant for *Li*TryR inactivation at 3.1 µM TS_2. Progress curves for *Li*TryR enzymatic reactions in the presence of increasing concentrations of **19a** were fitted to Equation 1. Data are the results obtained in a representative assay from three independent experiments. The estimated k_{obs} values are shown in Table S2.

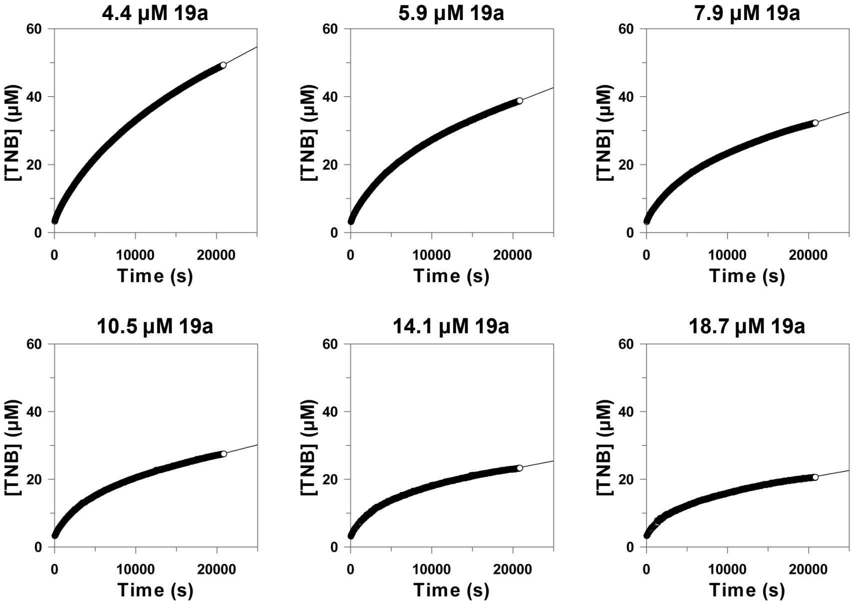

Figure S3. 19a concentration dependence of the observed rate constant for *Li*TryR inactivation at 6.2 µM TS_2. Progress curves for *Li*TryR enzymatic reactions in the presence of increasing concentrations of **19a** were fitted to Equation 1. Data are the results obtained in a representative assay from three independent experiments. The estimated k_{obs} values are shown in Table S2.

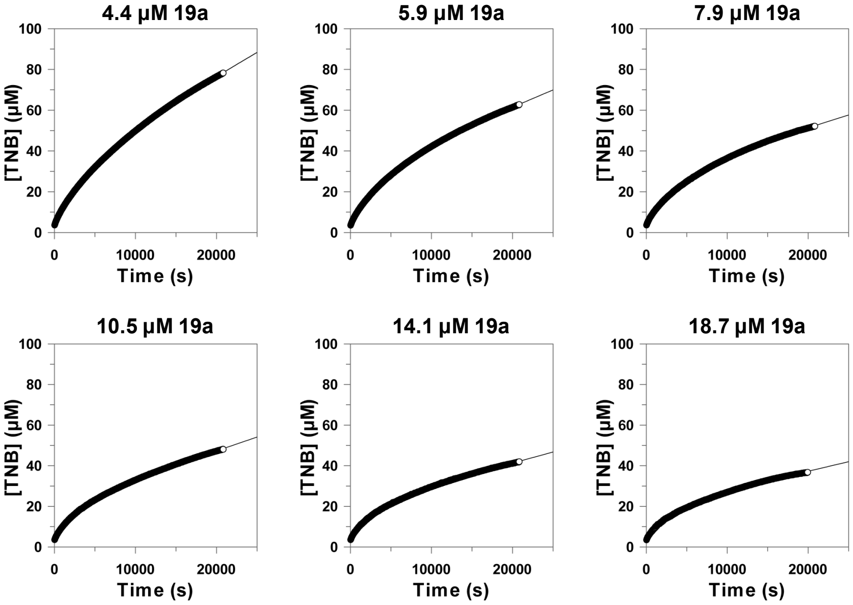

Figure S4. 19a concentration dependence of the observed rate constant for *Li*TryR inactivation at 12.5 µM TS_2. Progress curves for *Li*TryR enzymatic reactions in the presence of increasing concentrations of **19a** were fitted to Equation 1. Data are the results obtained in a representative assay from three independent experiments. The estimated k_{obs} values are shown in Table S2.

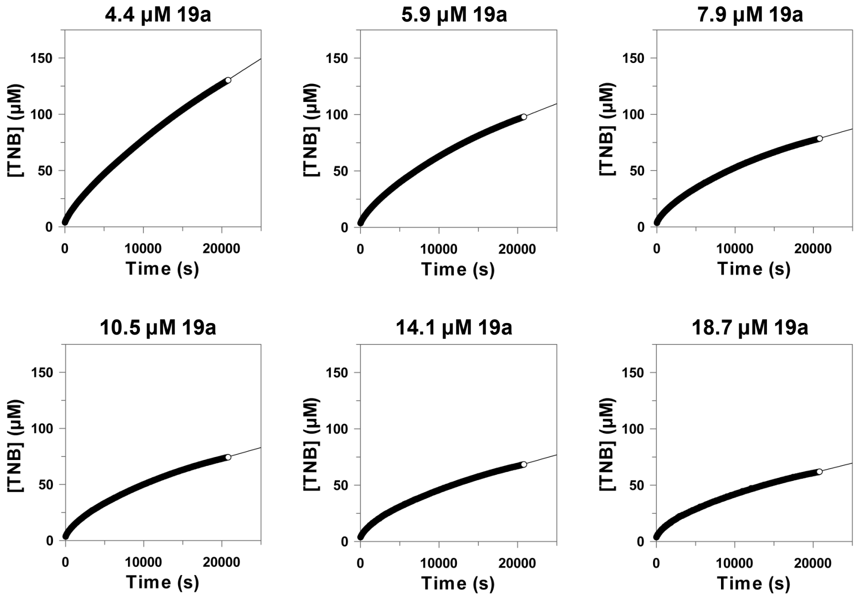

Figure S5. 19a concentration dependence of the observed rate constant for *Li*TryR inactivation at 25 µM TS_2. Progress curves for *Li*TryR enzymatic reactions in the presence of increasing concentrations of **19a** were fitted to Equation 1. Data are the results obtained in a representative assay from three independent experiments. The estimated k_{obs} values are shown in Table S2.

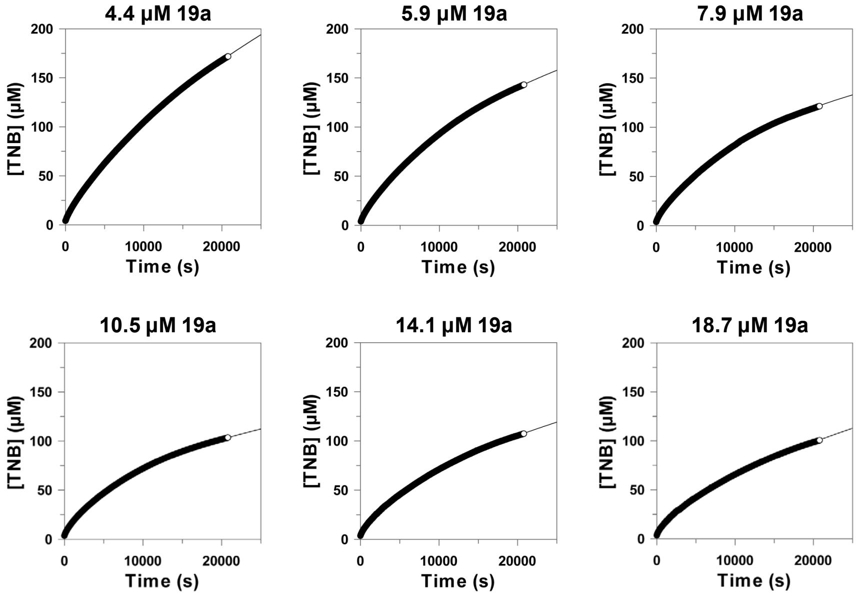

Figure S6. **19a** concentration dependence of the observed rate constant for *Li*TryR inactivation at 50 μM TS_2. Progress curves for *Li*TryR enzymatic reactions in the presence of increasing concentrations of **19a** were fitted to Equation 1. Data are the results obtained in a representative assay from three independent experiments. The estimated k_{obs} values are shown in Table S2.

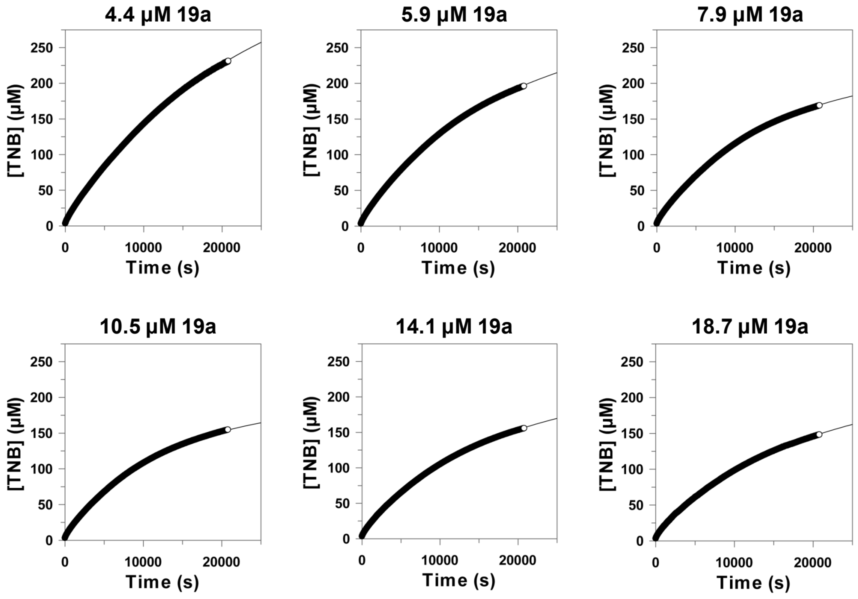

Figure S7. **19a** concentration dependence of the observed rate constant for *Li*TryR inactivation at 100 μM TS_2. Progress curves for *Li*TryR enzymatic reactions in the presence of increasing concentrations of **19a** were fitted to Equation 1. Data are the results obtained in a representative assay from three independent experiments. The estimated k_{obs} values are shown in Table S2.

Table S2. Rate constants for *Li*TryR time-dependent inhibition by 19a at different TS₂ concentrations. k_{obs} values for each **19a** concentration were obtained by fitting to Equation 1 the progress curves shown in Figures S2-S7. Results are the estimated values from the non-linear regression ± the associated standard errors.

	k_{obs} (s^{-1})					
[19a] (μM)	3.1 μM TS_2	6.2 μM TS_2	12.5 μM TS_2	25 μM TS_2	50 μM TS_2	100 μM TS_2
4,4	$1.5 \times 10^{-4} \pm 9.8 \times 10^{-7}$	$1.6 \times 10^{-4} \pm 1.2 \times 10^{-6}$	$1.7 \times 10^{-4} \pm 1.8 \times 10^{-6}$	$1.4 \times 10^{-4} \pm 2.3 \times 10^{-6}$	$9.4 \times 10^{-5} \pm 1.5 \times 10^{-6}$	$6.0 \times 10^{-5} \pm 6.2 \times 10^{-7}$
5,9	$2.1 \times 10^{-4} \pm 1.1 \times 10^{-6}$	$2.0 \times 10^{-4} \pm 1.1 \times 10^{-6}$	$1.9 \times 10^{-4} \pm 1.6 \times 10^{-6}$	$1.4 \times 10^{-4} \pm 1.8 \times 10^{-6}$	$9.4 \times 10^{-5} \pm 1.2 \times 10^{-6}$	$7.9 \times 10^{-5} \pm 6.3 \times 10^{-7}$
7,9	$2.2 \times 10^{-4} \pm 1.6 \times 10^{-6}$	$2.2 \times 10^{-4} \pm 1.4 \times 10^{-6}$	$1.9 \times 10^{-4} \pm 1.9 \times 10^{-6}$	$1.5 \times 10^{-4} \pm 2.0 \times 10^{-6}$	$1.0 \times 10^{-4} \pm 1.1 \times 10^{-6}$	$8.4 \times 10^{-5} \pm 6.3 \times 10^{-7}$
10,5	$2.7 \times 10^{-4} \pm 2.0 \times 10^{-6}$	$2.7 \times 10^{-4} \pm 1.7 \times 10^{-6}$	$2.8 \times 10^{-4} \pm 2.3 \times 10^{-6}$	$1.7 \times 10^{-4} \pm 2.4 \times 10^{-6}$	$1.3 \times 10^{-4} \pm 1.4 \times 10^{-6}$	$9.4 \times 10^{-5} \pm 5.6 \times 10^{-7}$
14,1	$3.1 \times 10^{-4} \pm 3.6 \times 10^{-6}$	$2.8 \times 10^{-4} \pm 2.7 \times 10^{-6}$	$2.7 \times 10^{-4} \pm 2.8 \times 10^{-6}$	$2.1 \times 10^{-4} \pm 2.9 \times 10^{-6}$	$1.3 \times 10^{-4} \pm 2.0 \times 10^{-6}$	$9.0 \times 10^{-5} \pm 1.1 \times 10^{-6}$
18,7	$2.9 \times 10^{-4} \pm 5.1 \times 10^{-6}$	$2.8 \times 10^{-4} \pm 3.7 \times 10^{-6}$	$2.5 \times 10^{-4} \pm 4.2 \times 10^{-6}$	$2.2 \times 10^{-4} \pm 3.3 \times 10^{-6}$	$1.5 \times 10^{-4} \pm 2.5 \times 10^{-6}$	$8.8 \times 10^{-5} \pm 1.5 \times 10^{-6}$

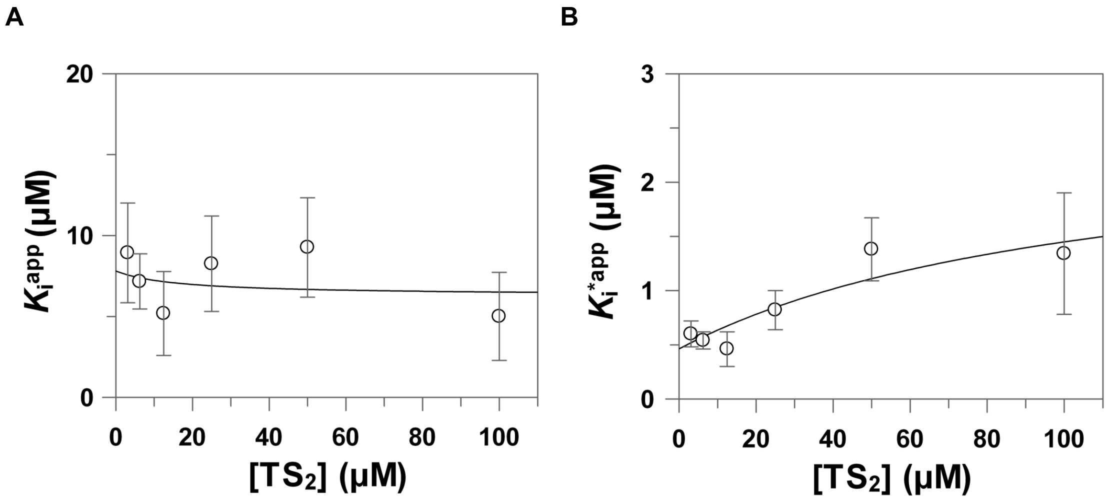

Figure S8. Effect of substrate concentration on the apparent inhibition constants K_i^{app} and K_i^{*app} upon binding of **19a** to *Li*TryR. (A) Plot of the estimated values of K_i^{app} (± SE) as a function of TS_2 concentration. The curve was fitted using Equation 3. (B) Plot of the estimated values of K_i^{*app} (± SE) as a function of TS_2 concentration. The curve was fitted using Equation 4.

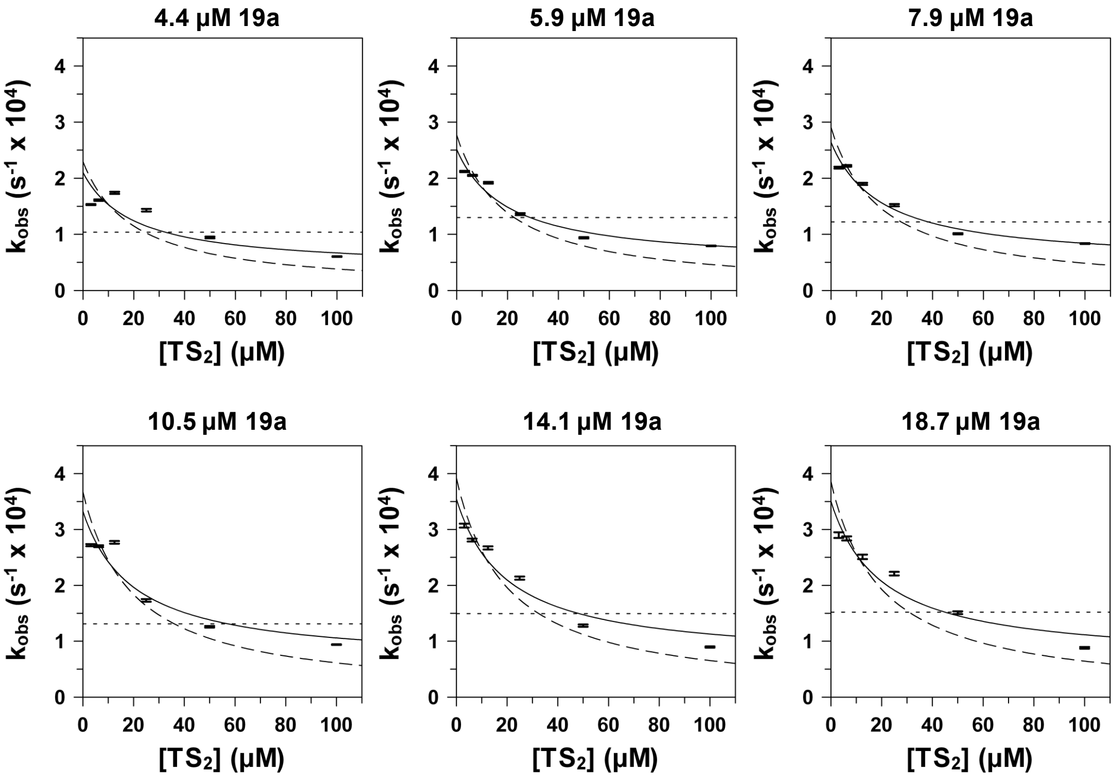

Figure S9. Effect of TS_2 concentration on the rate of *Li*TryR inactivation. Plot of the k_{obs} values (± standard errors) as a function of TS_2 concentration at six different **19a** concentrations (4.4, 5.9, 7.9, 10.5, 14.1 and 18.7 µM). Curves for competitive inhibition (dashed line) were fitted using the following equation: $k_{obs} = k / [1 + ([S] / K_m)]$.[3,4] Curves for pure (dotted line; α = 1) and mixed (solid line; α = 5) noncompetitive inhibition were fitted using the following equation: $k_{obs} = [k \times (K_m + ([S] / \alpha)] / (K_m + S)$.[5]

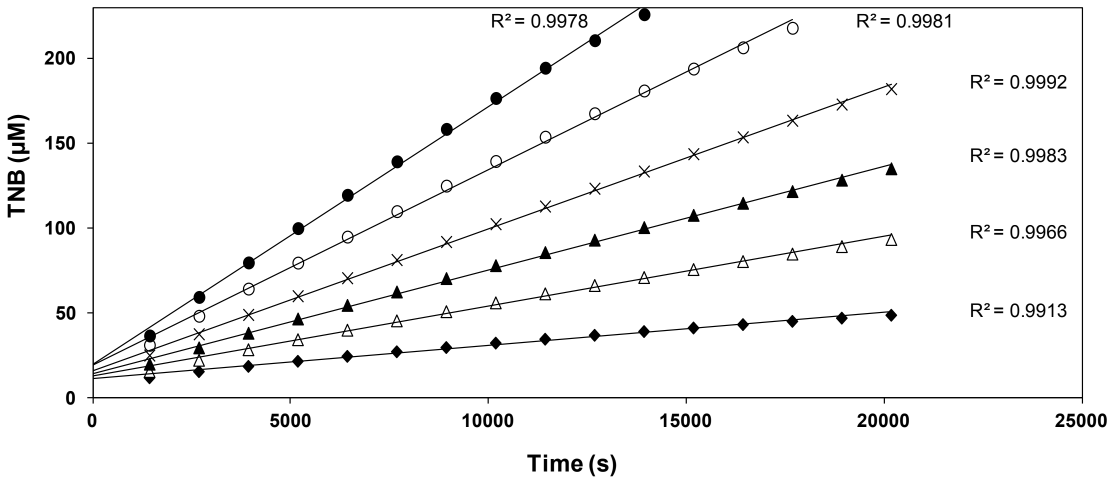

Figure S10. Progress curves for *Li*TryR enzymatic reactions in the absence of inhibitor at six different concentrations of TS_2: 3.1 μM (◆), 6.2 μM (△), 12.5 μM (▲), 25 μM (x), 50 μM (○) and 100 μM (●). Reaction progress curves were fitted to a linear trend line and R^2 values of each fit are shown in the figure. Oxidoreductase reactions were performed in a buffer containing 40 mM HEPES pH 7.5, 0.8 nM *Li*TryR, 1 mM EDTA, 300 μM NADPH, 60 μM $NADP^+$, 150 μM DTNB and 3.1 - 100 μM TS_2.

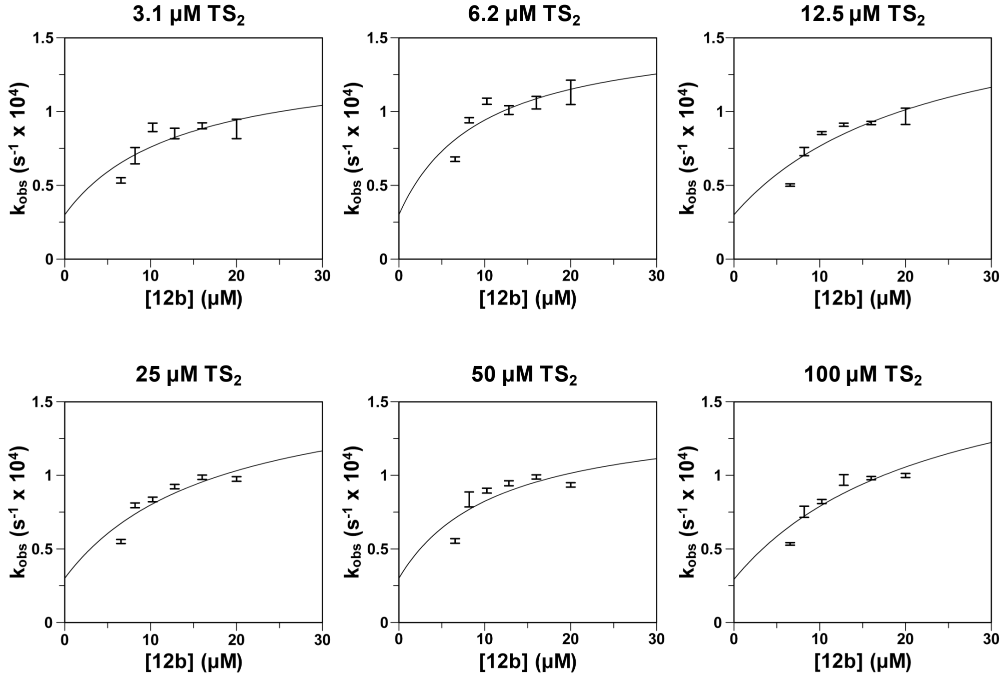

Figure S11. *Li*TryR inhibition by **12b**. Concentration dependence of the observed rate constants of *Li*TryR inactivation by **12b** at different TS_2 concentrations. Plot of the k_{obs} values (± standard errors) as a function of inhibitor concentration at six different TS_2 concentrations (3.1, 6.2, 12.5, 25, 50 and 100 µM). Data for *Li*TryR inactivation by **12b** were fitted using Equation 2 that describes the enzyme isomerization mechanism of time-dependent inhibition.

Table S3. Estimation of the kinetic parameters of *Li*TryR inactivation by **12b** in the reversible two-step mechanism of time-dependent inhibition (Scheme 4).

Parameters	12b
K_i (μM) [&]	10.0 ± 2.7
α [&]	2.0 ± 1.3
K_i^* (μM) [#]	2.6 ± 0.6
α* [#]	1.3 ± 0.6

[&] Estimated values ± standard error of K_i and α for the first rapid equilibrium that generates the EI complex (Scheme 4). Both values were estimated by fitting to Equation 3 the K_i^{app} values obtained at six different TS_2 concentrations (3.1, 6.2, 12.5, 25, 50 and 100 μM) using Equation 2.

[#] Estimated values ± standard error of K_i^* and α* for the overall two-step mechanism of time-dependent inactivation of *Li*TryR (Scheme 4). Both values were estimated by fitting to Equation 4 the K_i^{*app} values obtained at six different TS_2 concentrations (3.1, 6.2, 12.5, 25, 50 and 100 μM) using Equation 2.

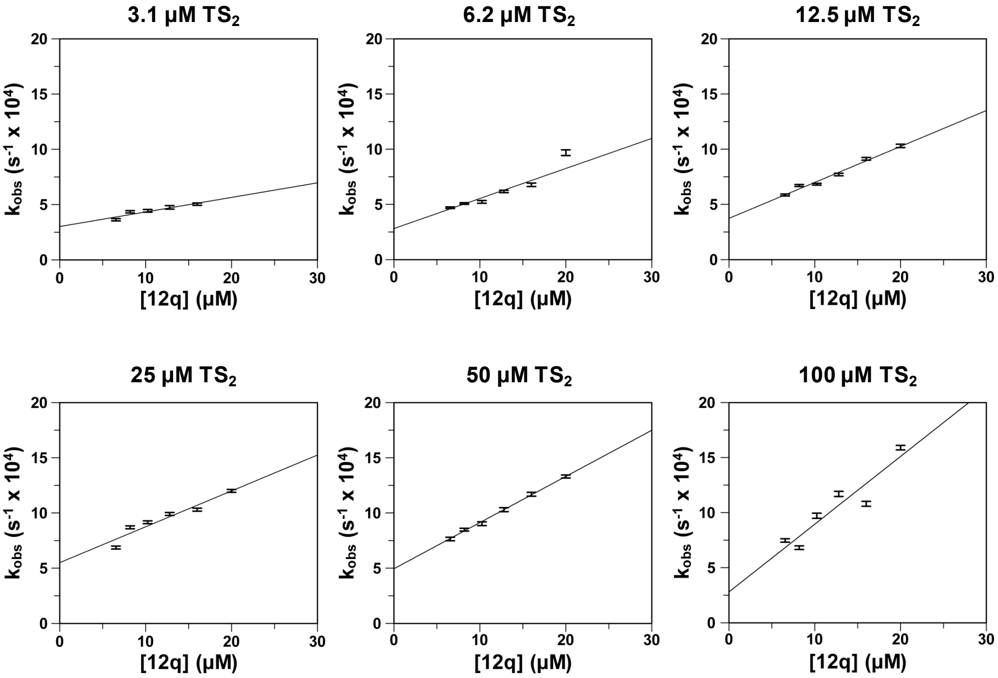

Figure S12. *Li*TryR inhibition by **12q**. Concentration dependence of the observed rate constants of *Li*TryR inactivation by **12q** at different TS_2 concentrations. Plot of the k_{obs} values (± standard errors) as a function of inhibitor concentration at six different TS_2 concentrations (3.1, 6.2, 12.5, 25, 50 and 100 µM). Data for *Li*TryR inactivation by **12q** were fitted using the following equation: $k_{obs} = k_4 \times (1 + [I] / K_i^{app})$ that describes the simple reversible mechanism of slow binding of inhibition.[3]

Table S4. Estimation of the kinetic parameters of *Li*TryR inhibition by **12q** in the reversible single-step mechanism of time-dependent inhibition (Scheme S4).

Parameters	12q
K_i (µM) [&]	13.6 ± 5.1
α [&]	0.3 ± 0.2

[&] Estimated values ± standard error of K_i and α for the slow and reversible equilibrium that generates the EI complex (Scheme S4). Both values were estimated by fitting to Equation 3 the K_i^{app} values obtained at six different TS_2 concentrations using the equation for time-dependent inhibitors with a single-step binding mechanism.

Scheme S4. Reversible single-step mechanism of time-dependent inhibition of *Li*TryR by **12q**&

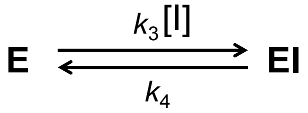

& A slow and reversible equilibrium between enzyme and inhibitor is governed by association and dissociation rate constants (k_3 and k_4, respectively).

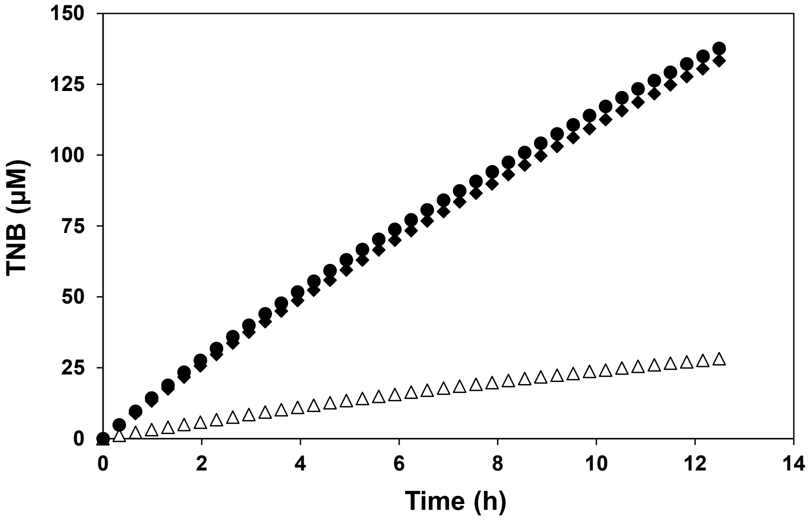

Figure S13. Residual activity of *Li*TryR after incubation with **19a**. *Li*TryR (400 nM) was incubated during 16 h in the absence of inhibitor (◆) or in the presence of 25 µM of mepacrine (●) or **19a** (△). Samples were diluted (2500-fold) and residual activity was evaluated. Oxidoreductase reactions were performed in a buffer containing 40 mM HEPES pH 7.5, 1 mM EDTA, 300 µM NADPH, 60 µM $NADP^+$, 150 µM DTNB and 100 µM TS_2.

Scheme S5. Global mechanism for *Li*TryR inactivation in the presence of substrate (S) and slow-binding inhibitors (I) **12b-c**, **12r-s** and **19a**. E*I is a conformationally inactive form of E produced through a slow process governed by the forward isomerization rate (k_5) and the very small reverse rate constant (k_6).

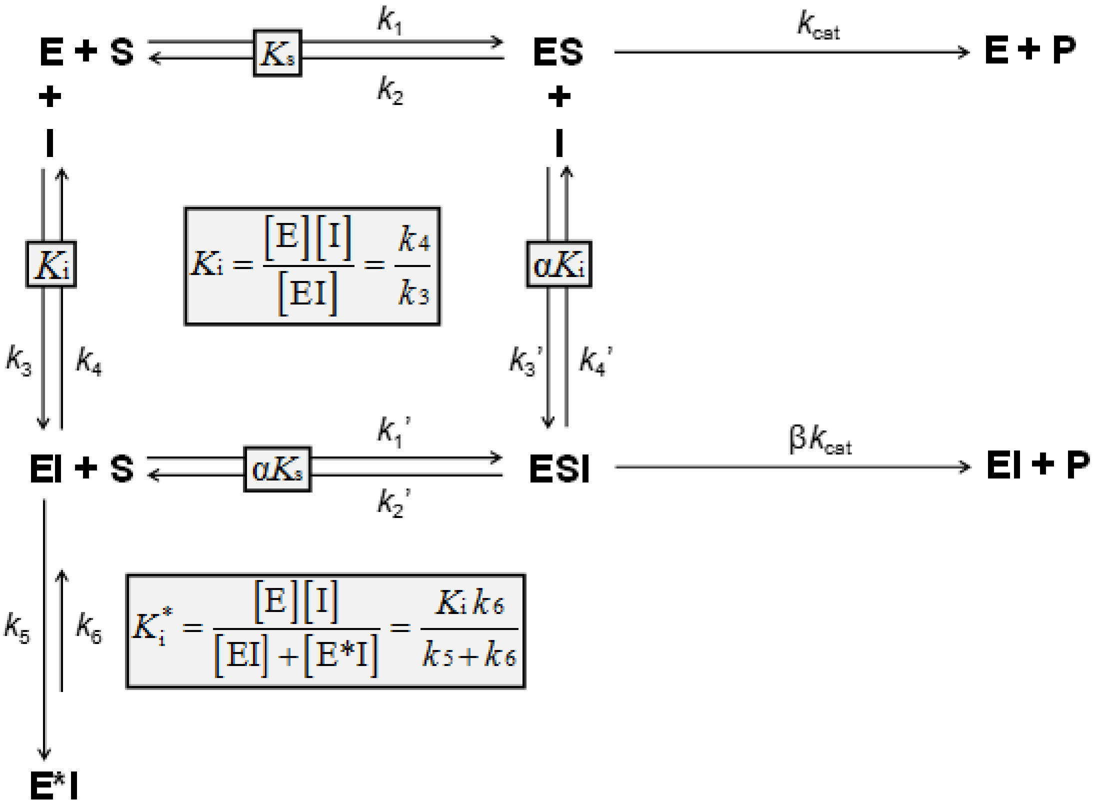

Screening for PAINS

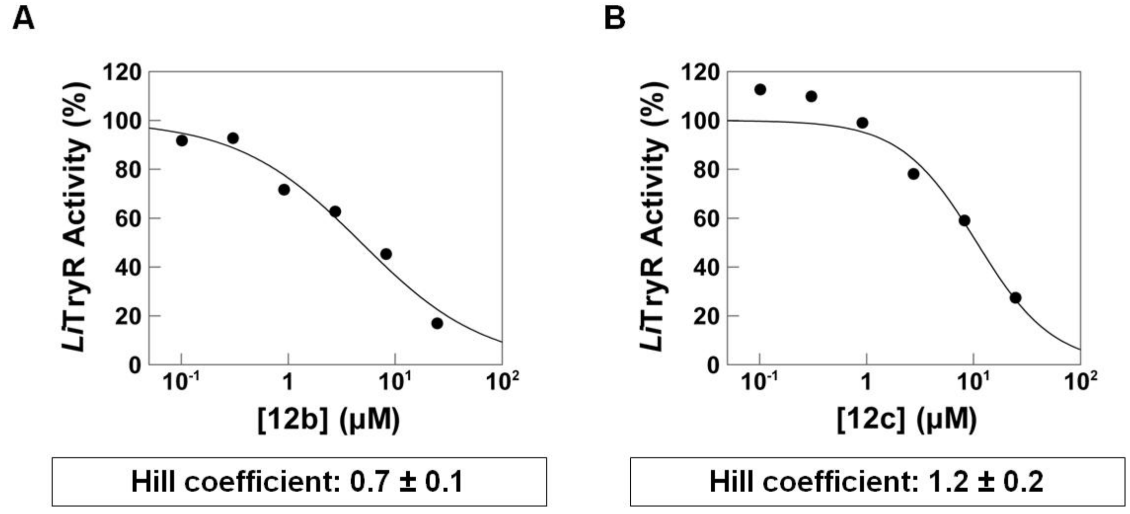

Figure S14. Concentration−response curves of representative triazole-based compounds **12b** (A) and **12c** (B). Hill coefficients are indicated in the boxes.

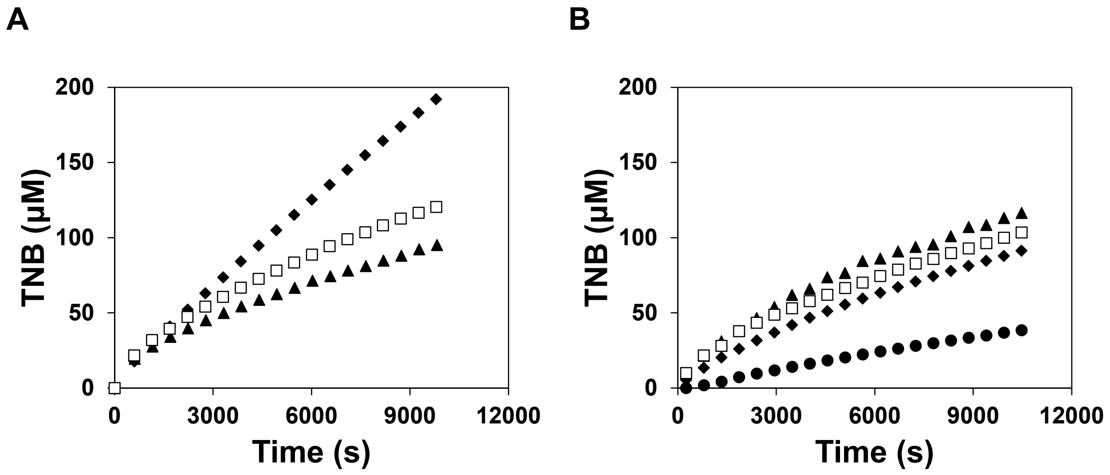

Figure S15. Oxidoreductase activity of *Li*TryR and hGR in the presence of 50 μM TS_2 or GSSG, respectively. (A) Reaction progress curves of *Li*TryR (0.8 nM) in the absence (◆) and in the presence 25 μM **12b** (▲) and 25 μM **12c** (□). (B) Reaction progress curves of hGR (7 nM) in the absence (◆) and in the presence of 25 μM nifurtimox (●), 25 μM **12b** (▲) and 25 μM **12c** (□).

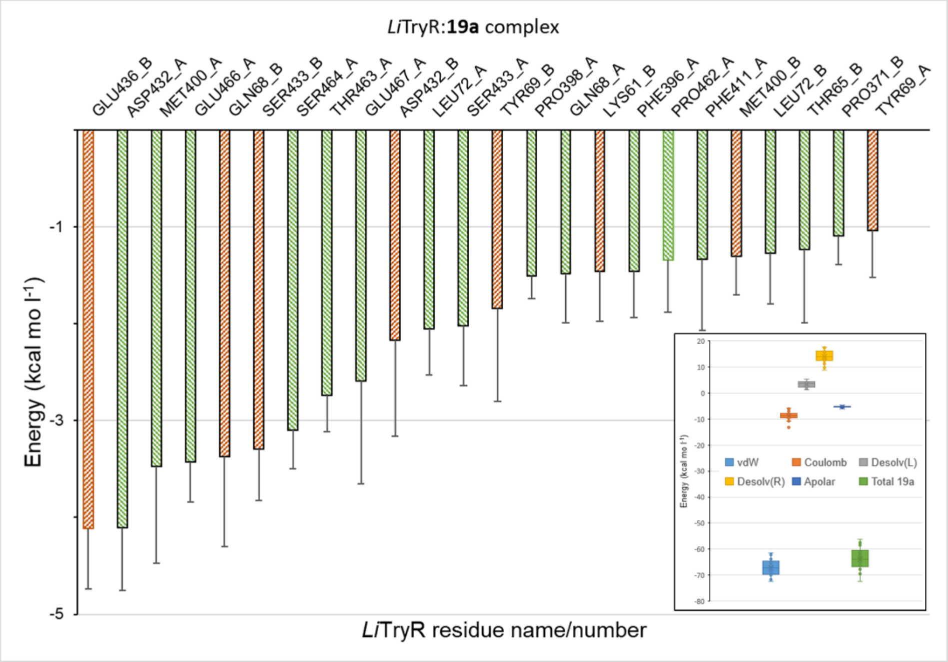

Figure S16. Calculated average (± standard error) contributions of individual *Li*TryR residues (monomers A and B in red and green, respectively) to the overall solvent-corrected interaction energy (kcal mol^{-1}) with **19a**. For simplicity, a cutoff of 1.0 was used. The averages were calculated from a conformational ensemble made up of 20 snapshots taken every 5 ns from the post-equilibrated 10–110 ns interval of the molecular dynamics trajectories and then cooled down to 273 K and energy minimized.

Inset: Box-and-whisker plots of the calculated total interaction energies (green, kcal mol^{-1}) and component contributions [DOI 10.1021/ct300497z][6] (van der Waals (light blue), electrostatic (orange), ligand desolvation (grey), receptor desolvation (yellow), and apolar (dark blue) for the binding of **19a** to *Li*TryR.

A

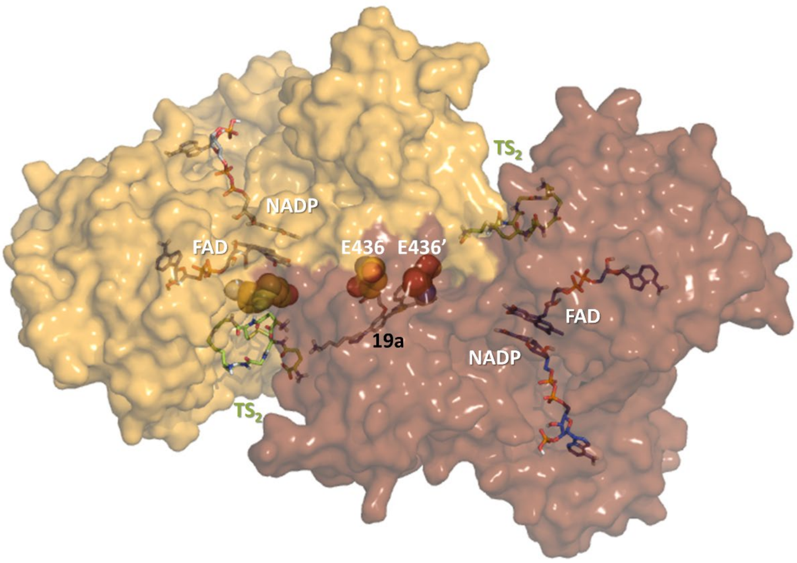

B

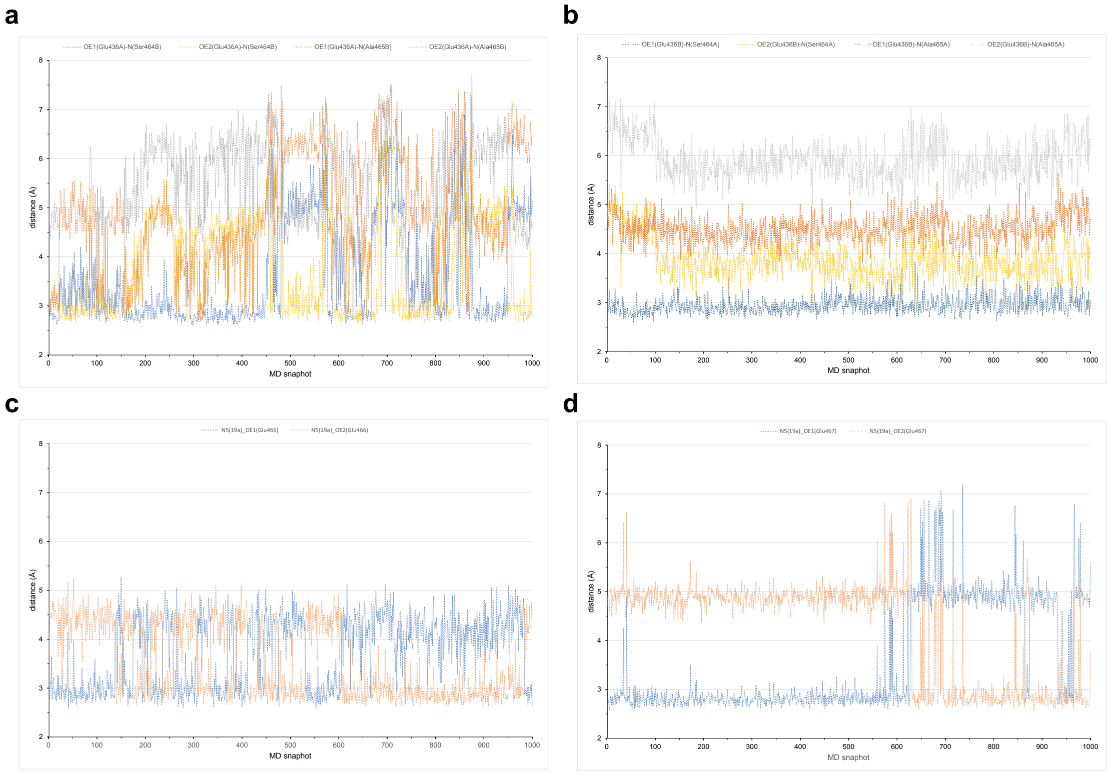

Figure S17. A. Theoretical model of *Li*TryR (enveloped in a semi-transparent surface) in complex with **19a** (sticks) bound in the central interfacial cavity. Each monomer is colored differently and both the flavin adenine dinucleotide (FAD) prosthetic group and the NADP cofactor are displayed as sticks for reference. In addition, two TS2 molecules (sticks, with C atoms in olive) have been included in the active site (as found in PDB entry 1BZL[7]) to highlight that the proposed inhibitor-binding site is separate from it and there is no overlap between the two. The atoms shown as spheres belong to the active site Cys52 and Cys57 in one active site (*left*) and the dimerization hotspot Glu436 residues from both subunits.

B. Monitoring of relevant distances shows the **19a**-induced disruptions in hydrogen bonds involving the Glu436 carboxylates OE1 and OE2 that are essential for enzyme dimerization (**a**,**b**), and the establishment of stable hydrogen bonds between OE1 and OE2 of Glu466 with the amino group of **19a** throughout the MD trajectory.

Table S5. Residues making up the interfacial cavity present in trypanothione-disulfide reductases (TryRs) from trypanosomatids and positionally equivalent residues in human glutathione-disulfide reductase (hGR). The overall lack of identity in several crucial regions is in consonance with the observed marked selectivity for *Li*TryR.

*Li*TryR	*Leishmania sp./Trypanosoma sp.* TryR	Human GR
Lys61	lysine	Lys65
Thr65	threonine	Asn71
Gln68	glutamine	Val74
Tyr69	tyrosine	His75
Leu72	leucine/threonine/histidine	Phe78
Pro371	proline	Pro376
Phe396	phenylalanine	Phe403
Pro398	proline	Pro405
Met400	methionine	Tyr407
Phe411	phenylalanine	Cys417
Asp432	aspartic/glutamic	Leu438
Ser433	serine/glycine/asparagine	Gly439
Glu436	glutamic	Glu442
Pro462	proline	Pro468
Thr463	threonine	Thr469
Ser464	serine	Ser470
Glu466	glutamic	Glu472
Glu467	glutamic	Glu473

^{1}H NMR AND ^{13}C NMR SPECTRA OF FINAL TRIAZOLE COMPOUNDS

Compound 12a

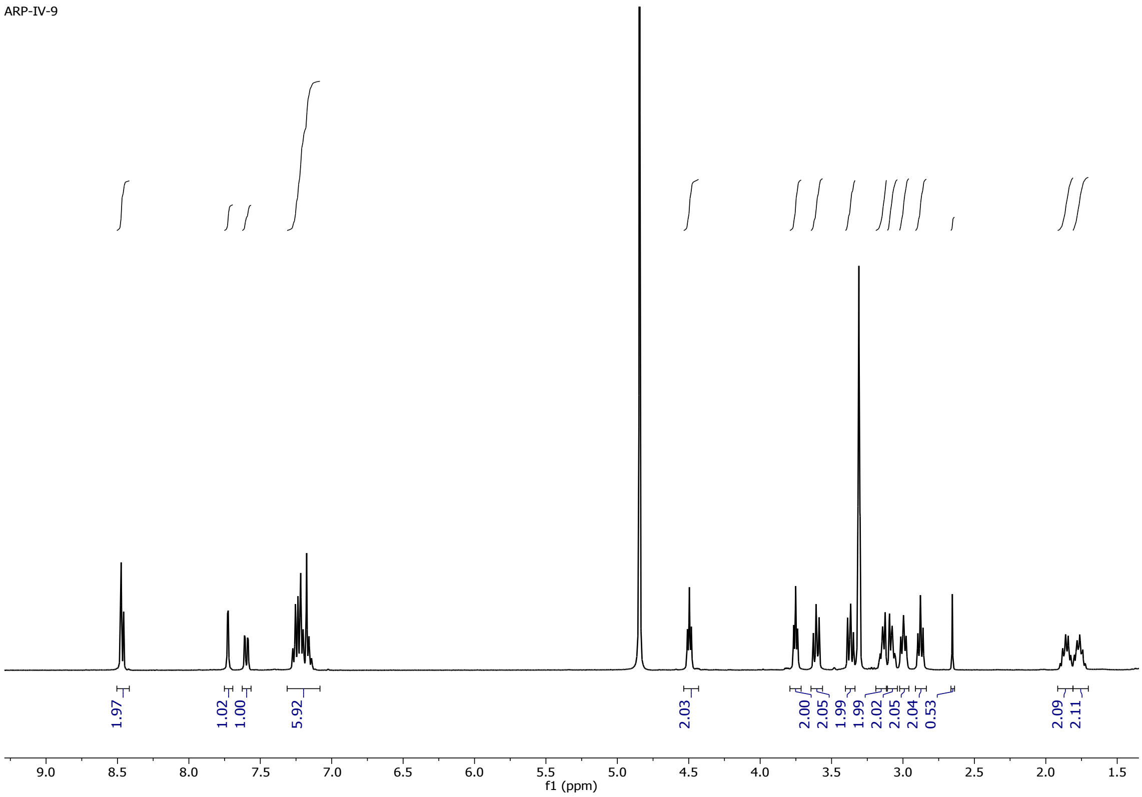

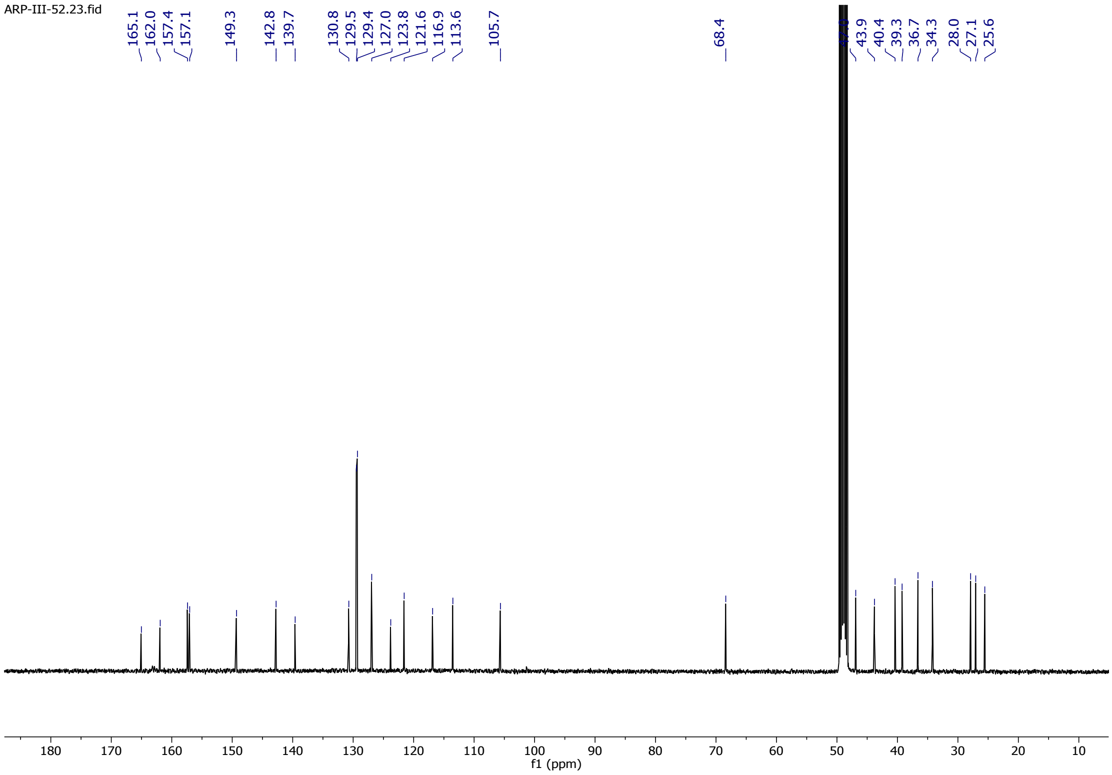

Compound 12b

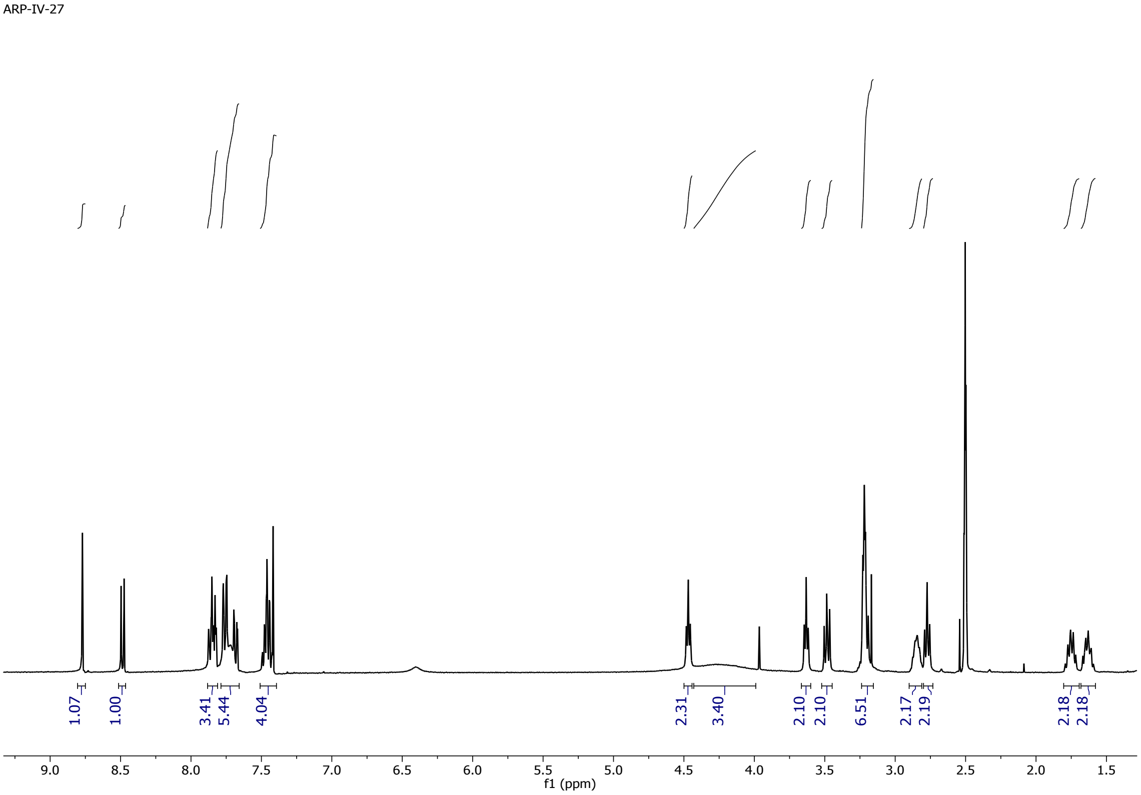

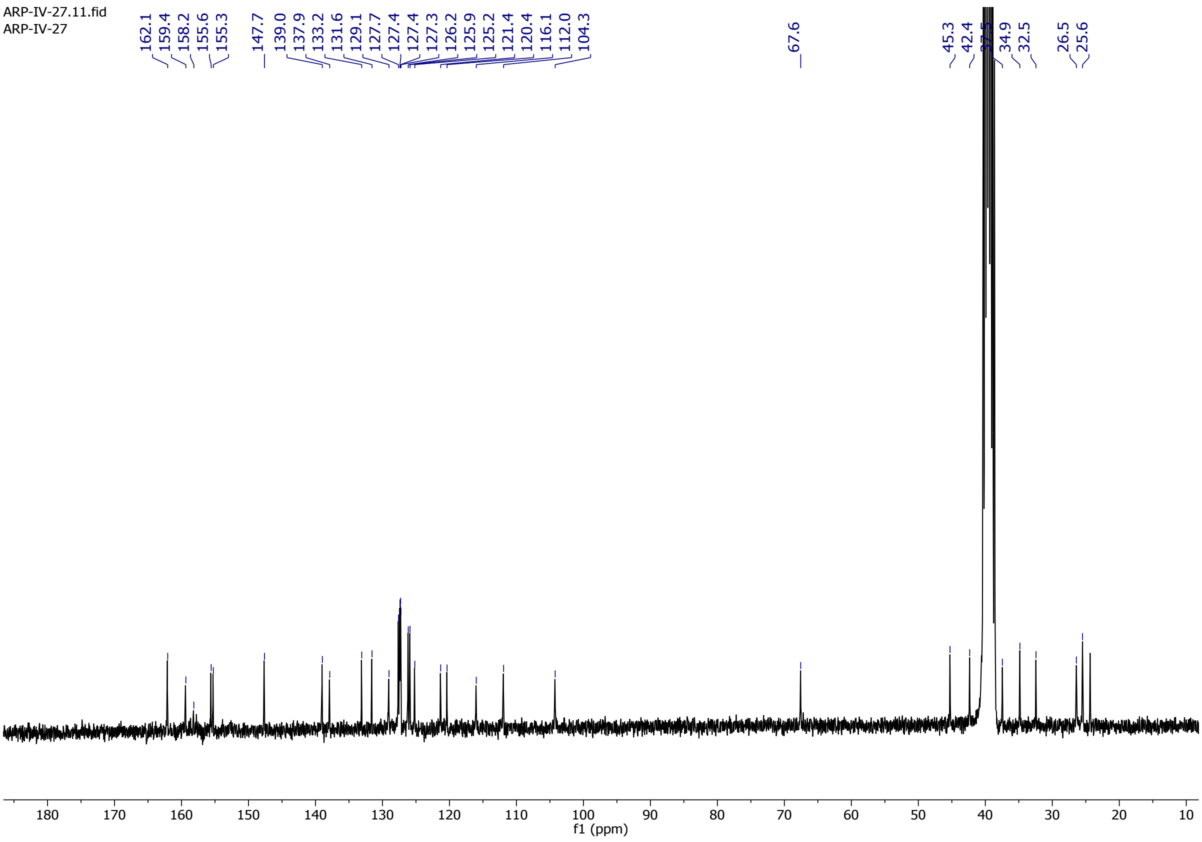

Compound 12c

ARP-V-47ColF19-23

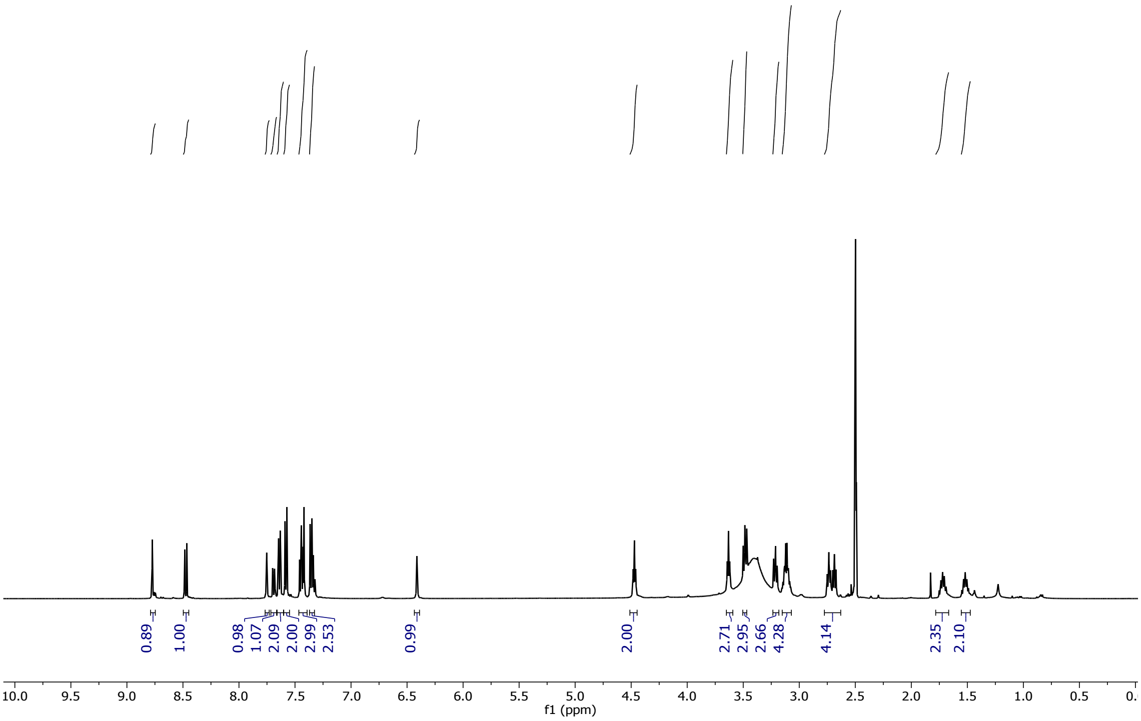

ARP_V_47ColF16-23.10.fid

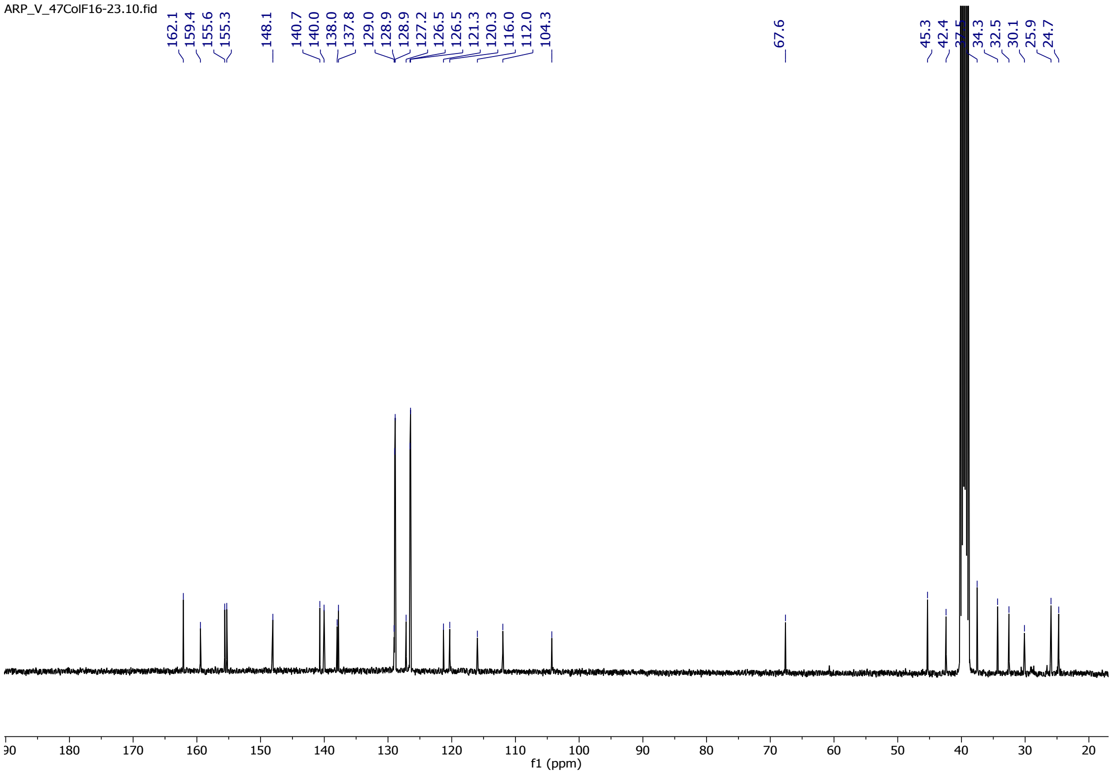

Compound 12d

ARP-IV-42COLF18-25

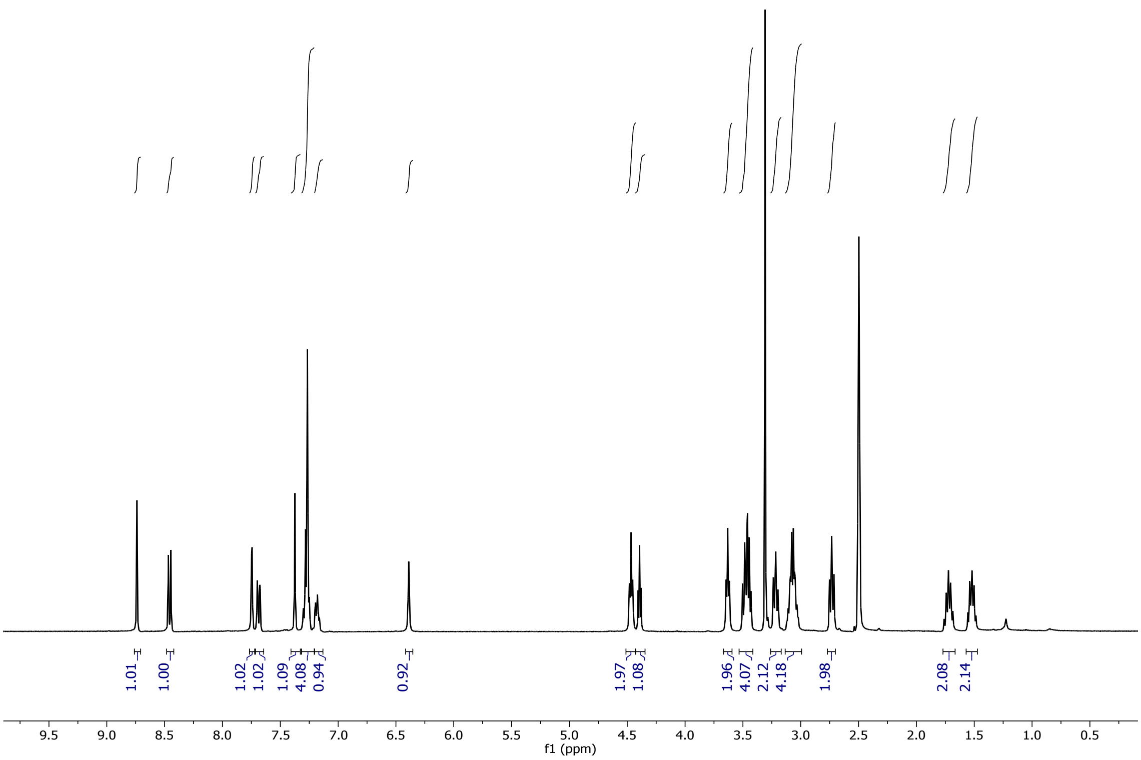

ARP-IV-42ColF18-25.10.fid
ARP-IV-42ColF18-25

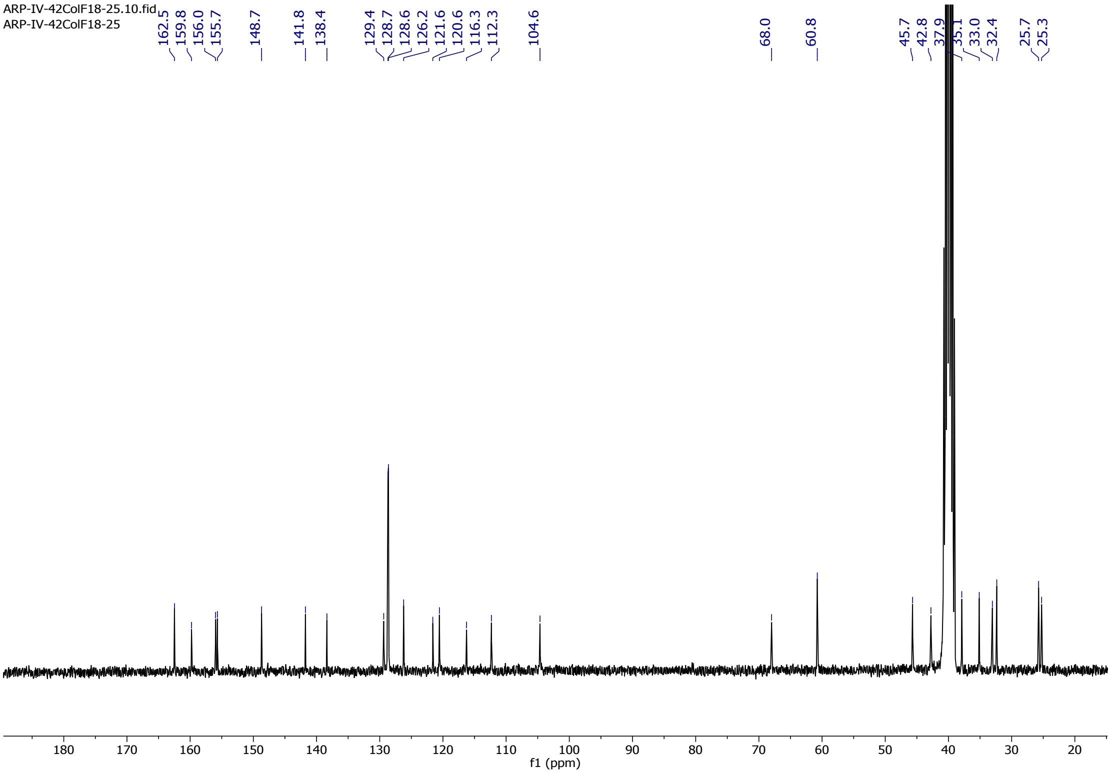

Compound 12e

ARP-IV-36ColF13-14

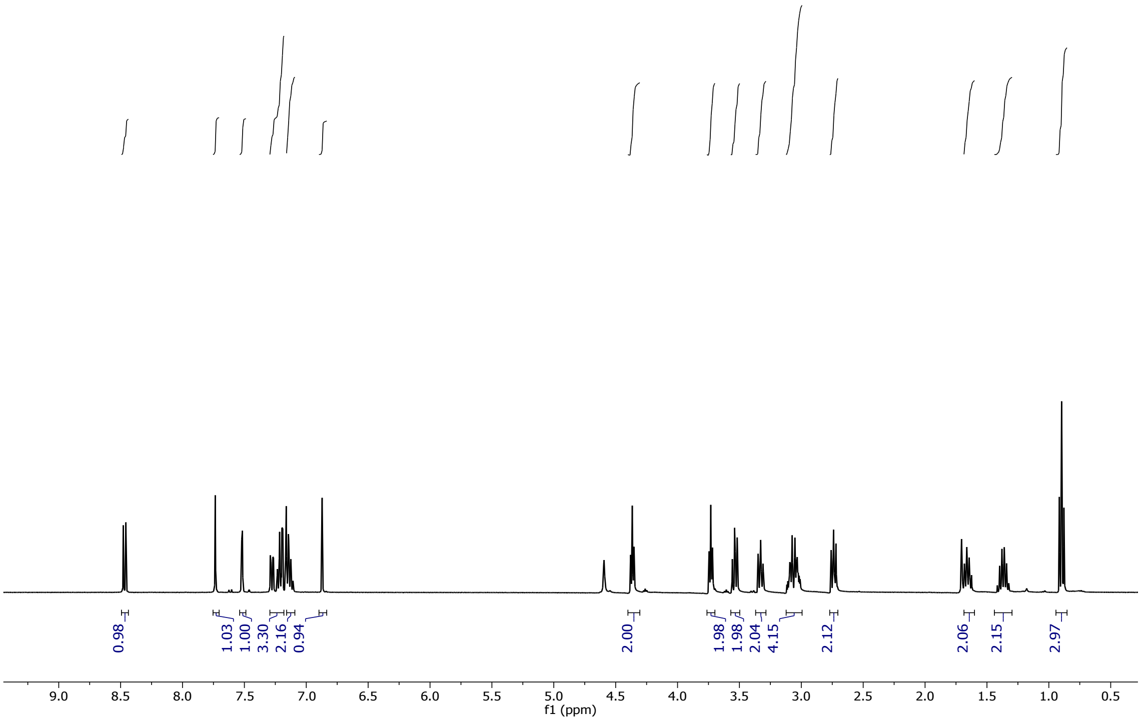

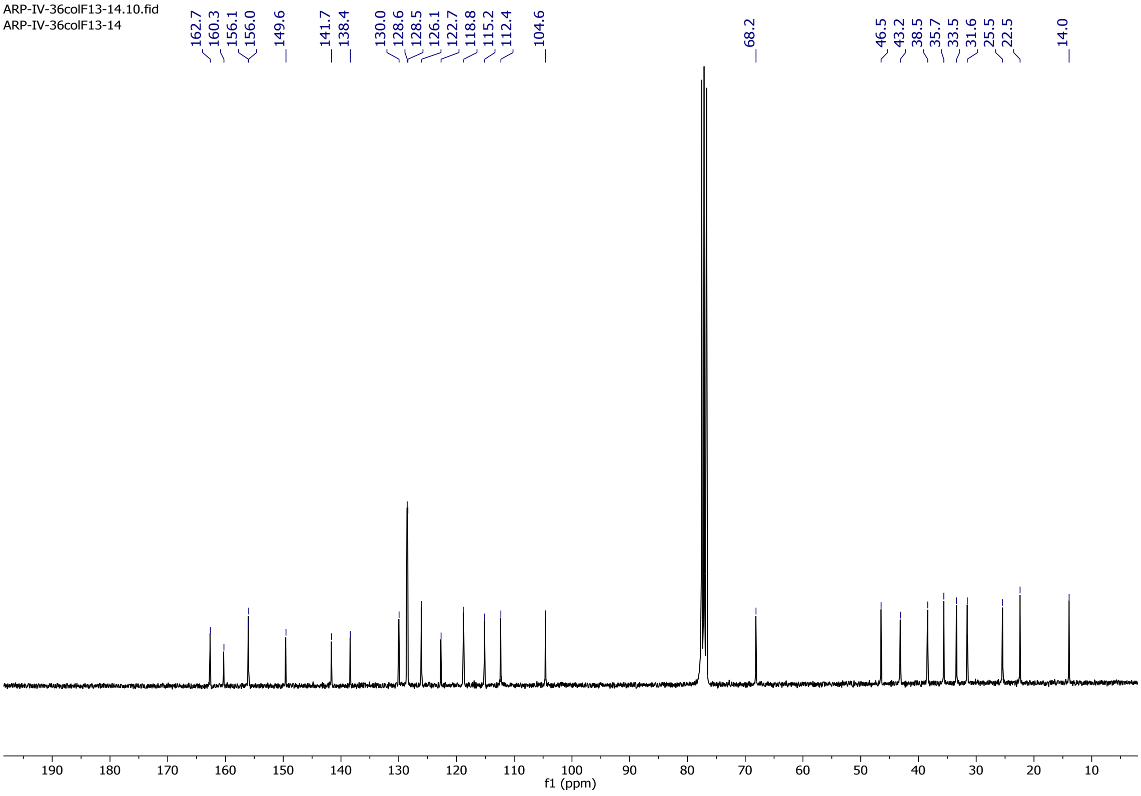

Compound 12f

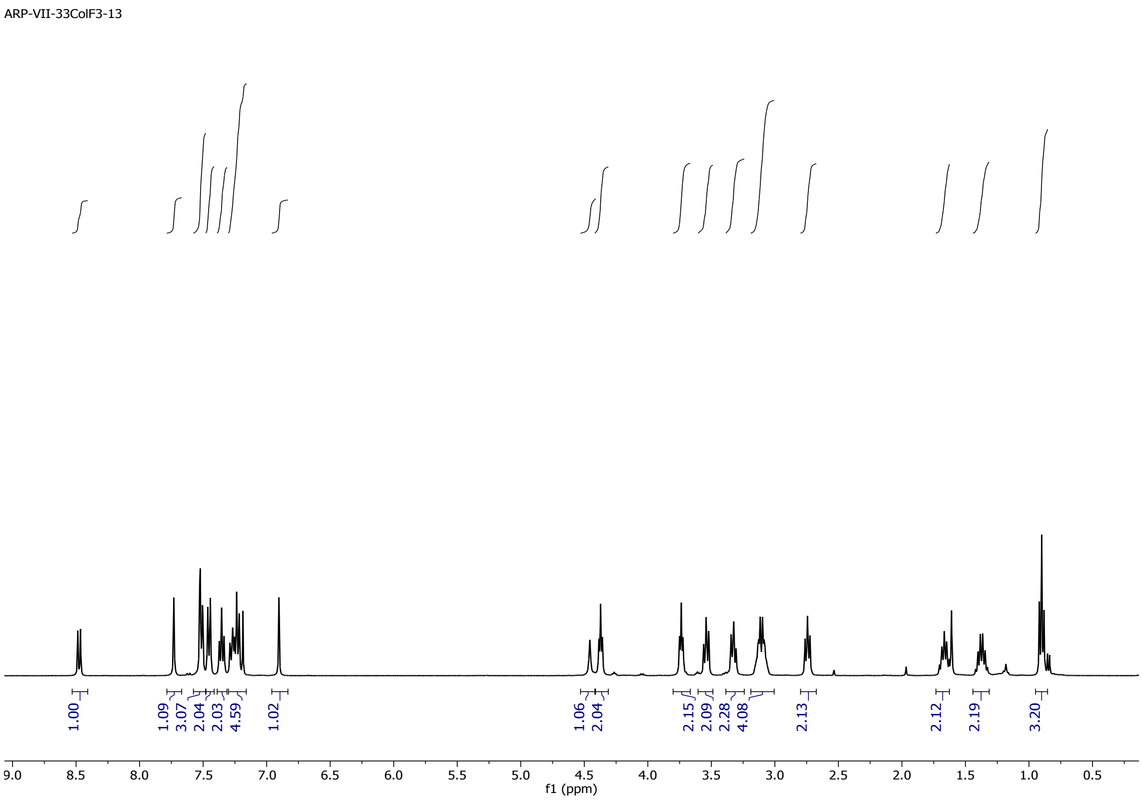

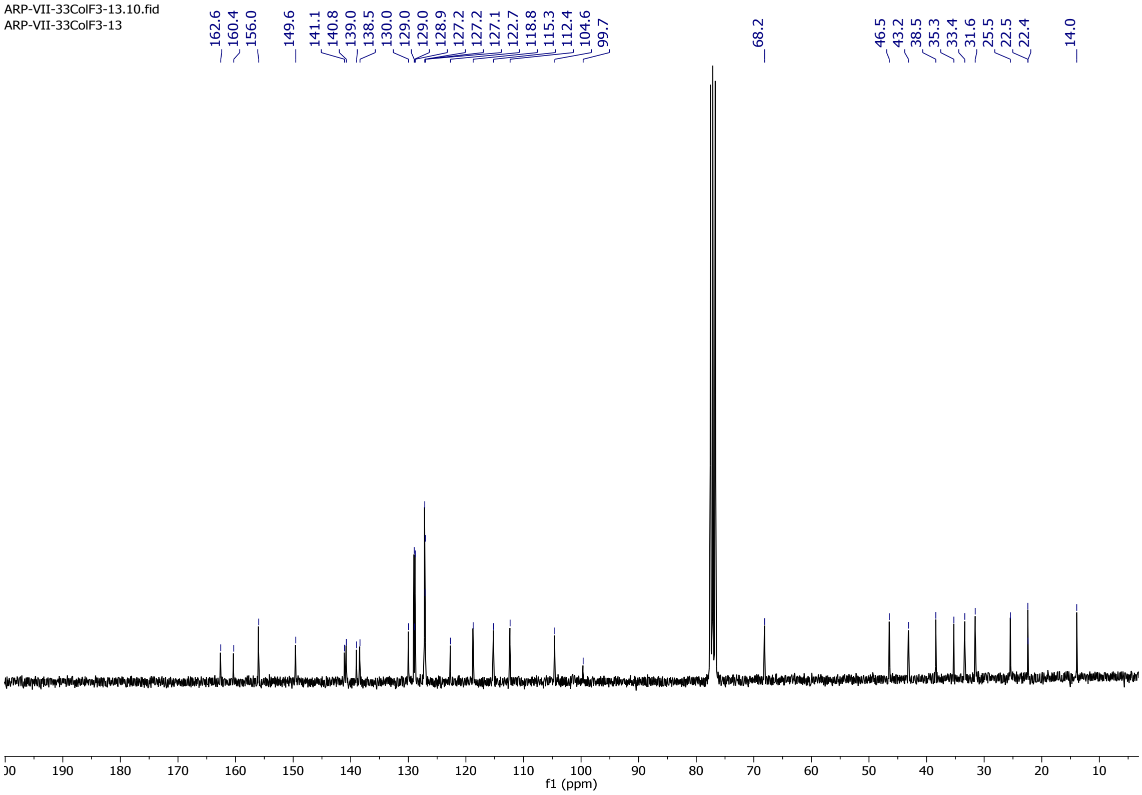

Compound 12g

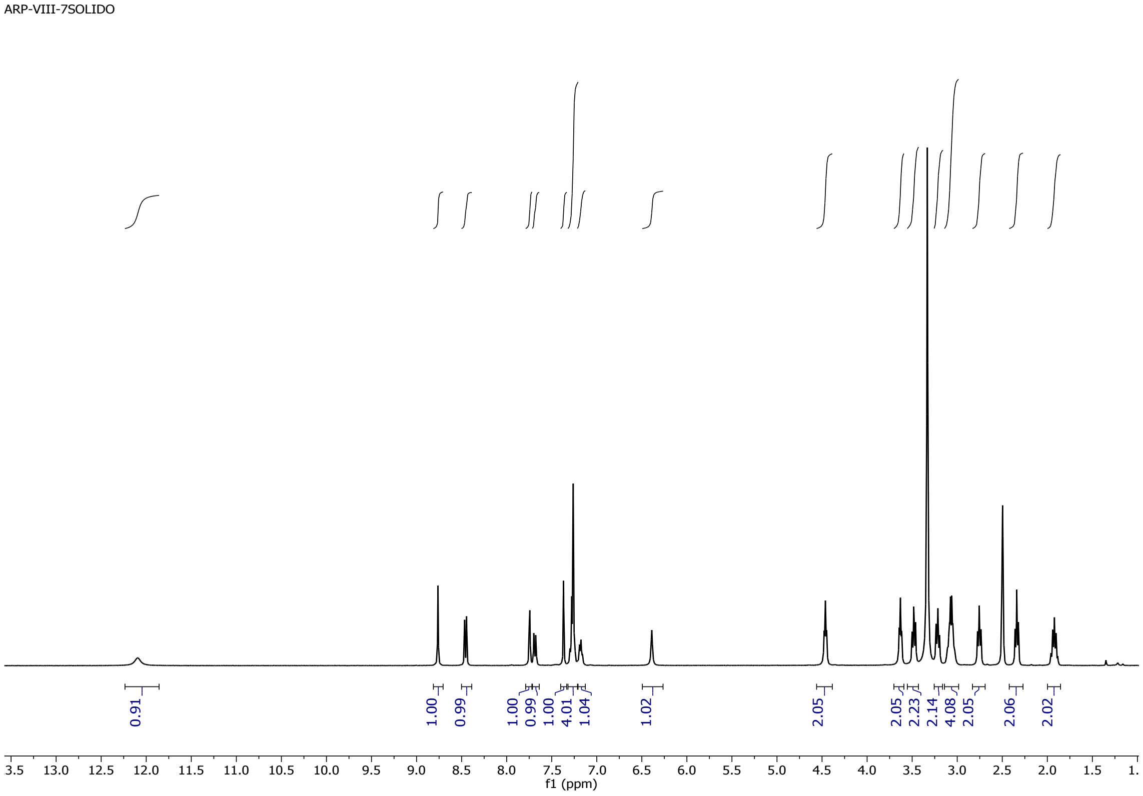

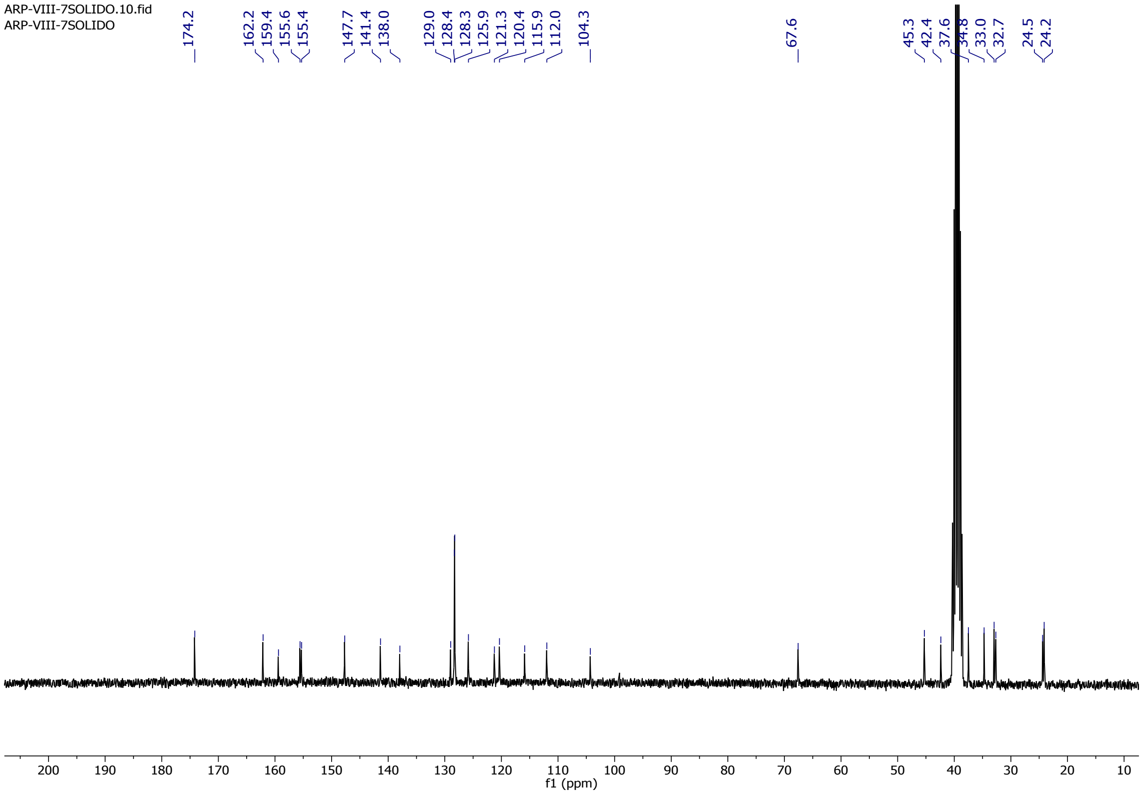

Compound 12h

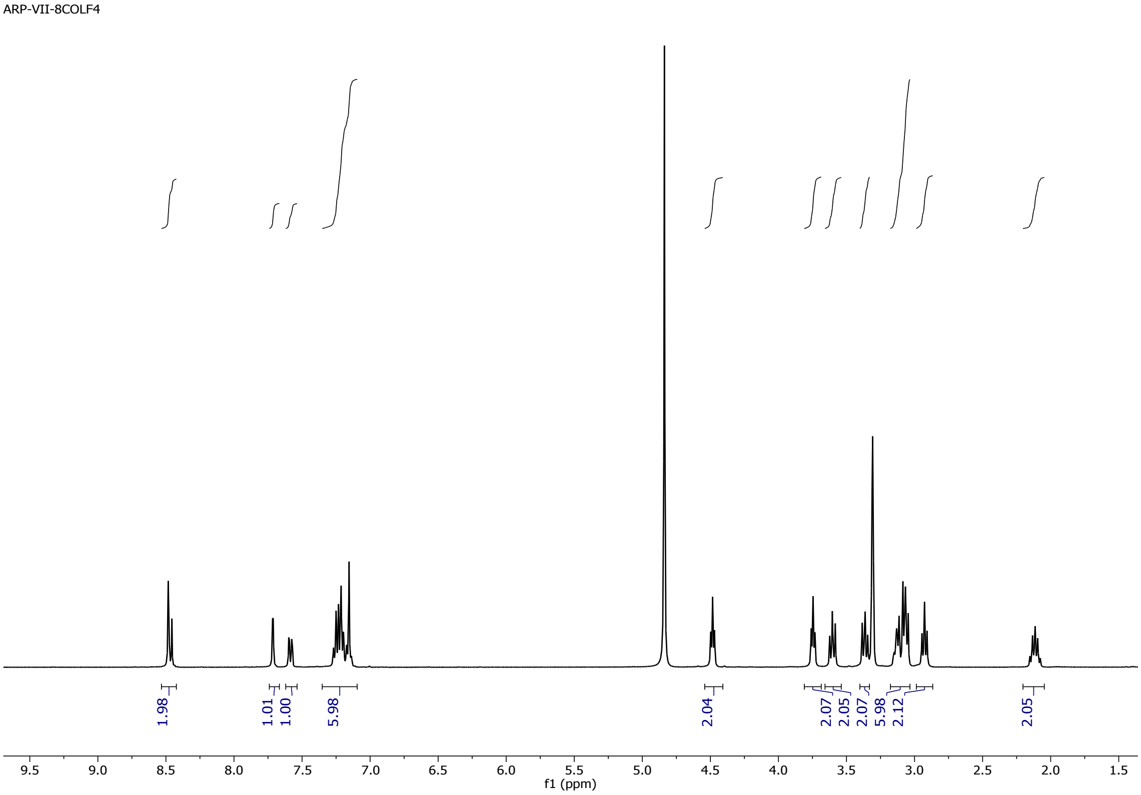

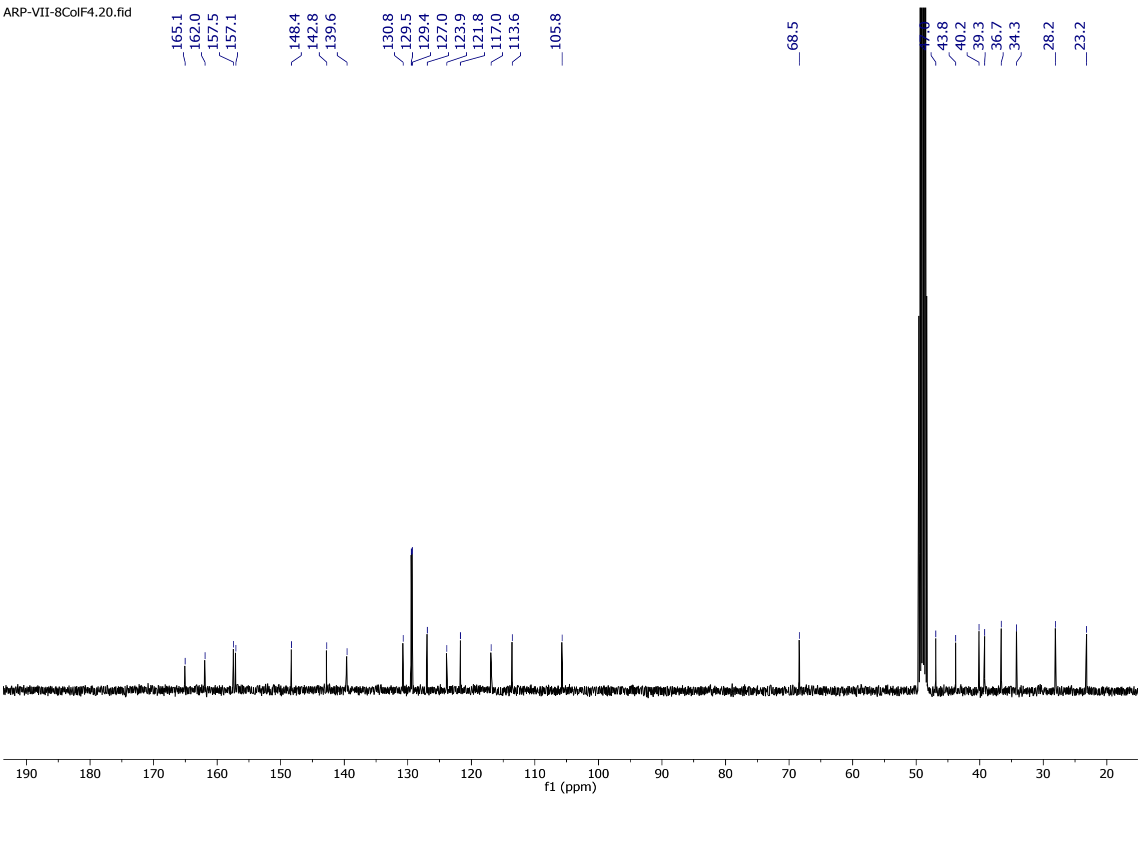

Compound 12i

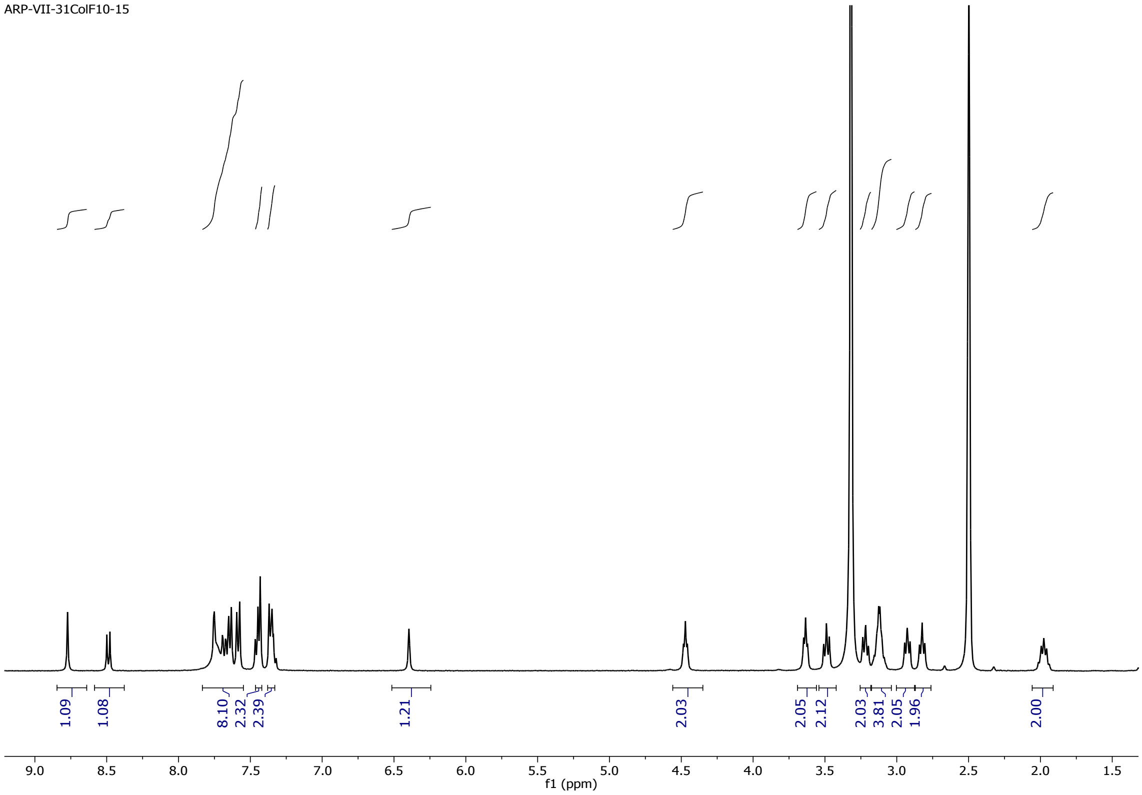

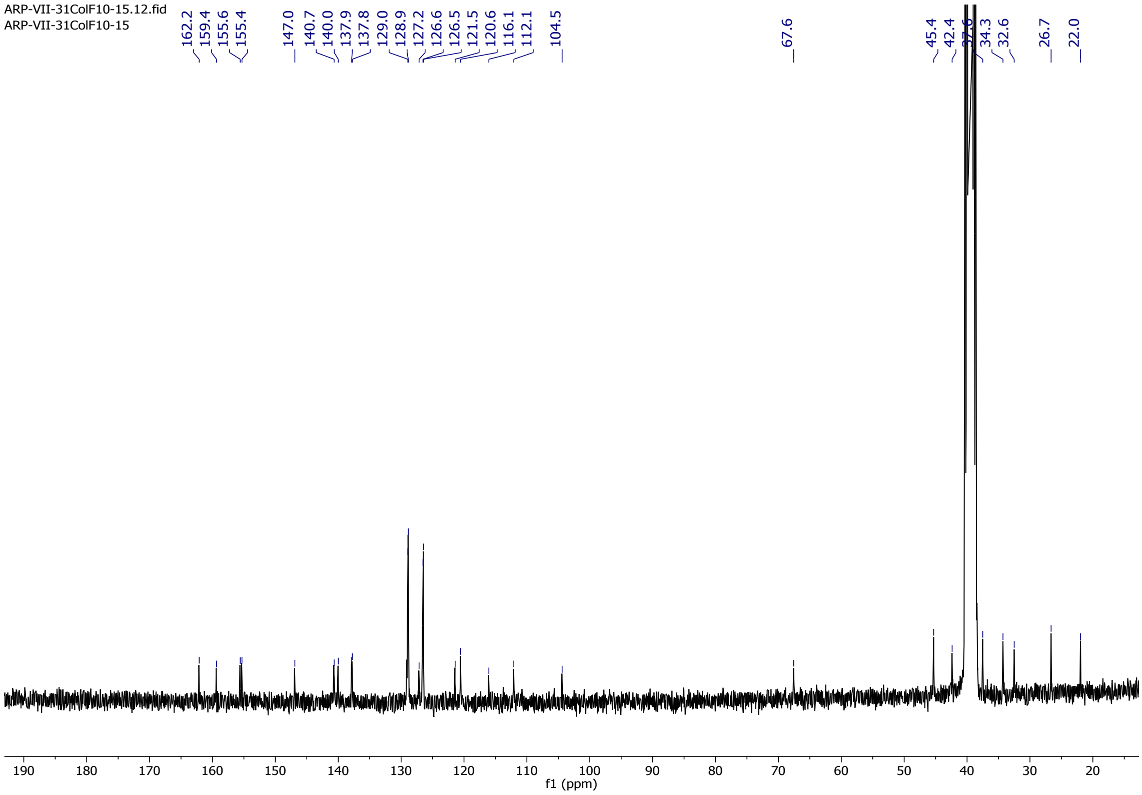

Compound 12j

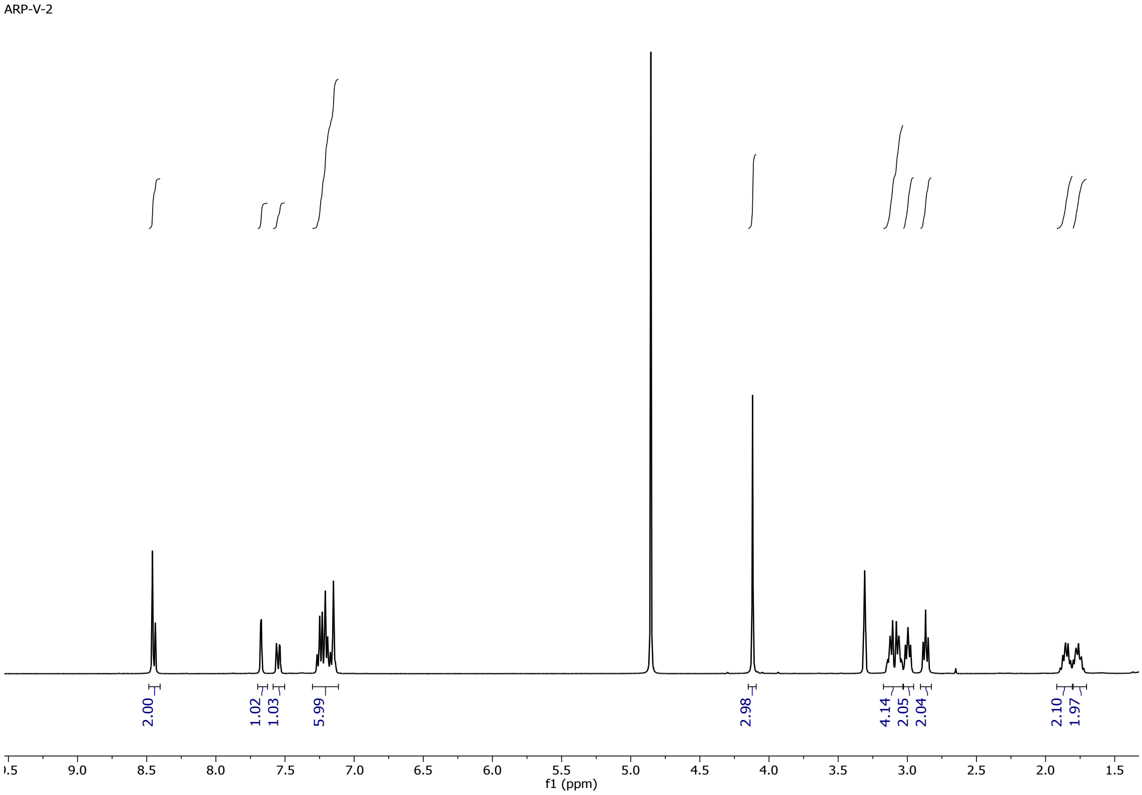

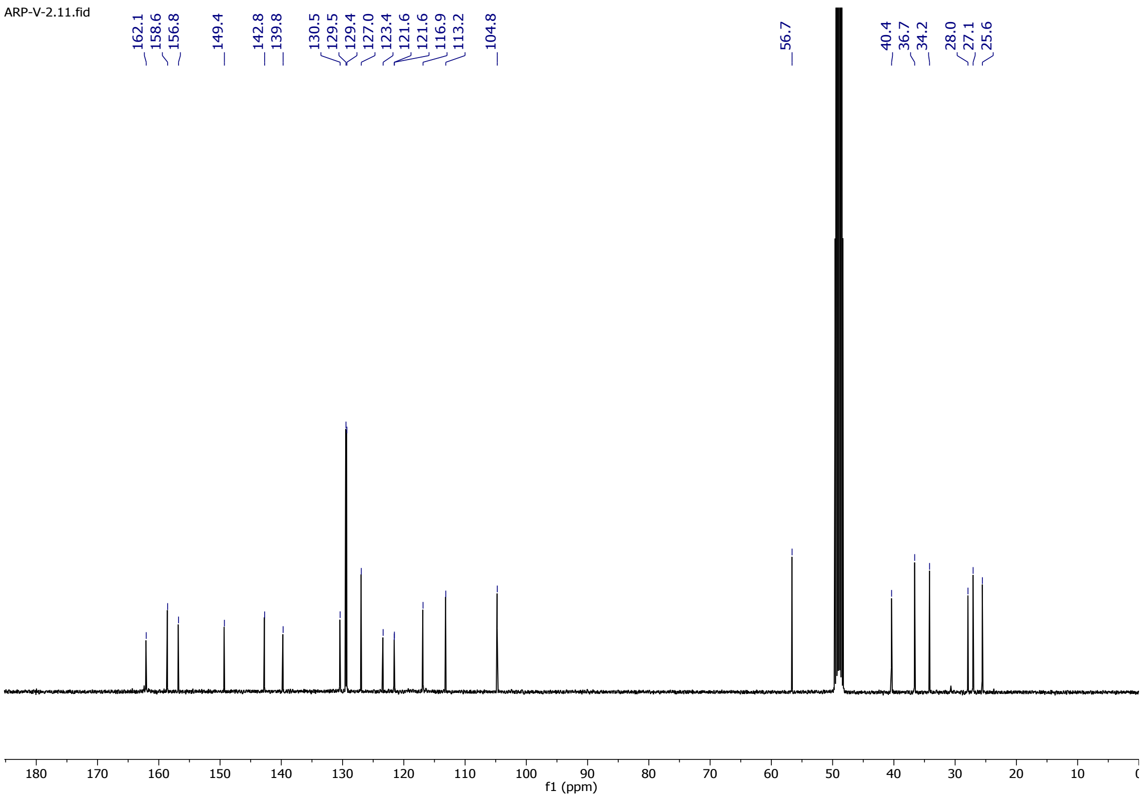

Compound 12k

ARP-X-20COLF4-10

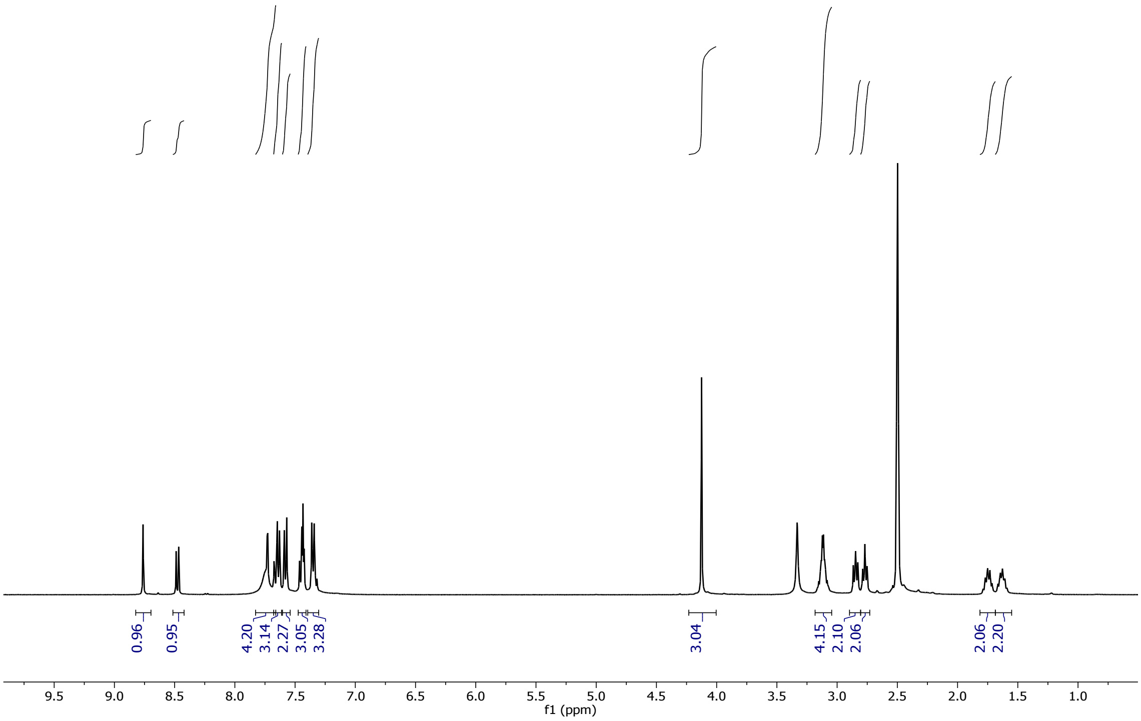

ARP-X-20ColF4-10.20.fid

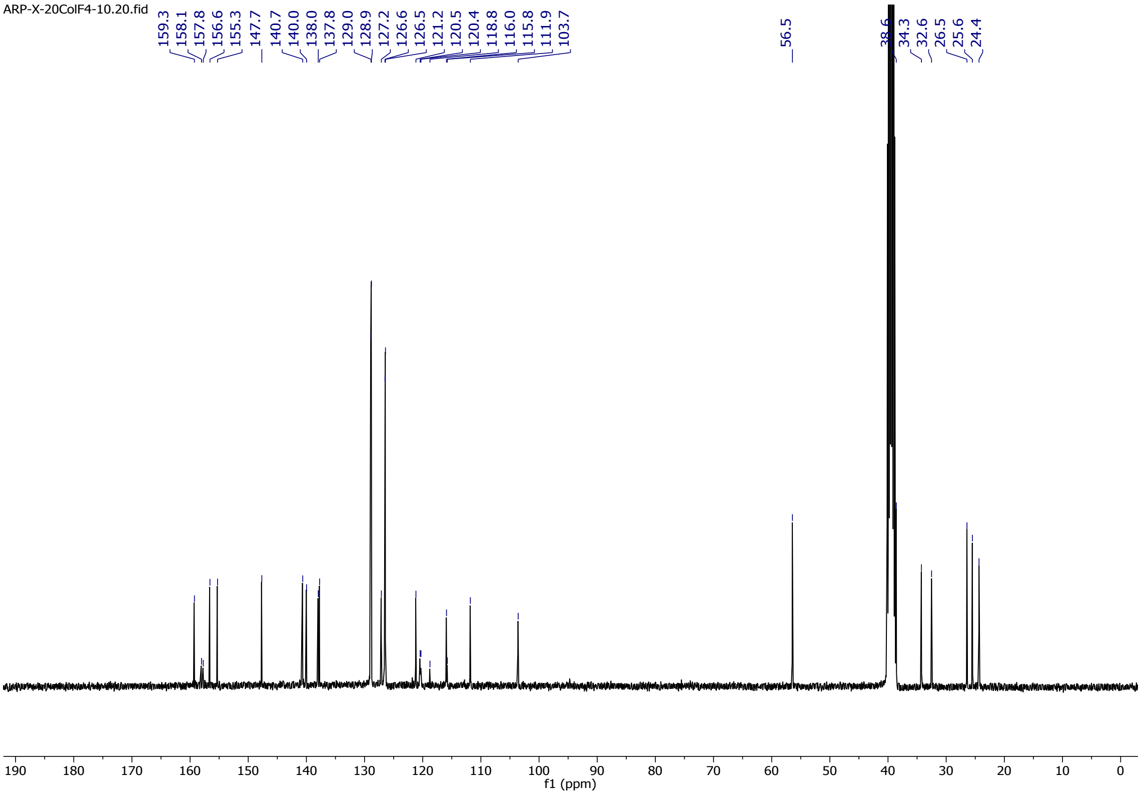

Compound 12l

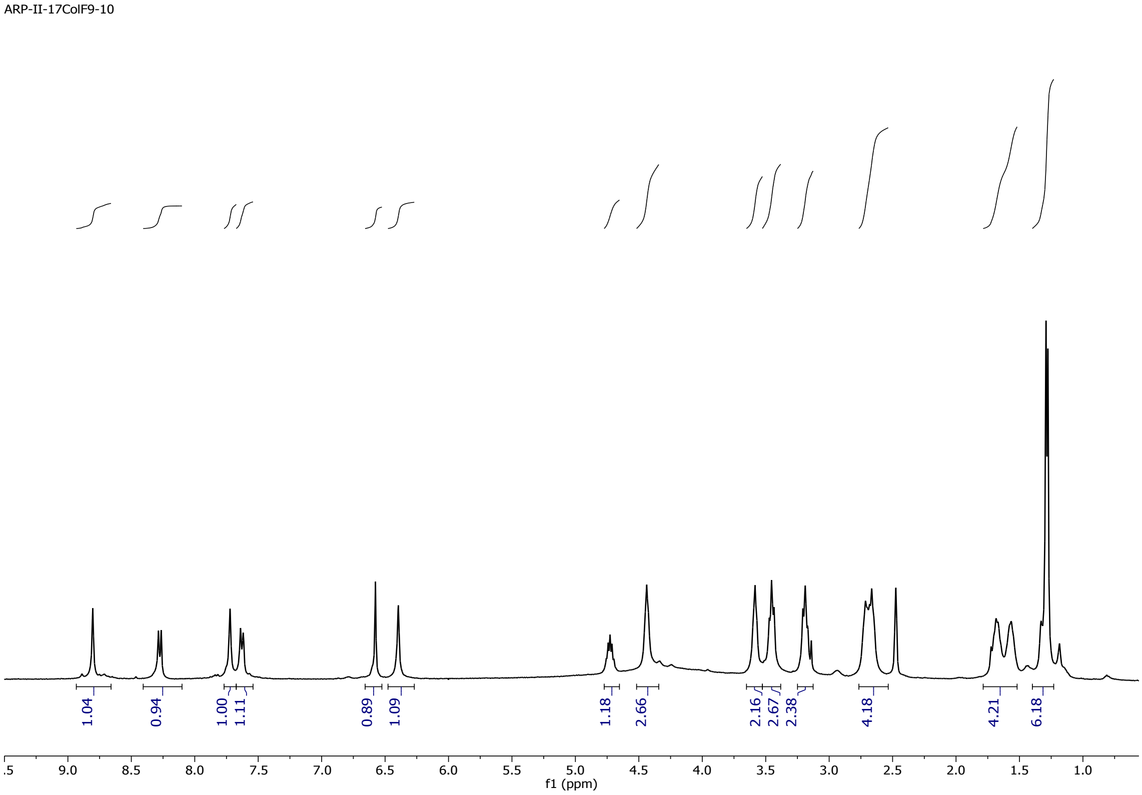

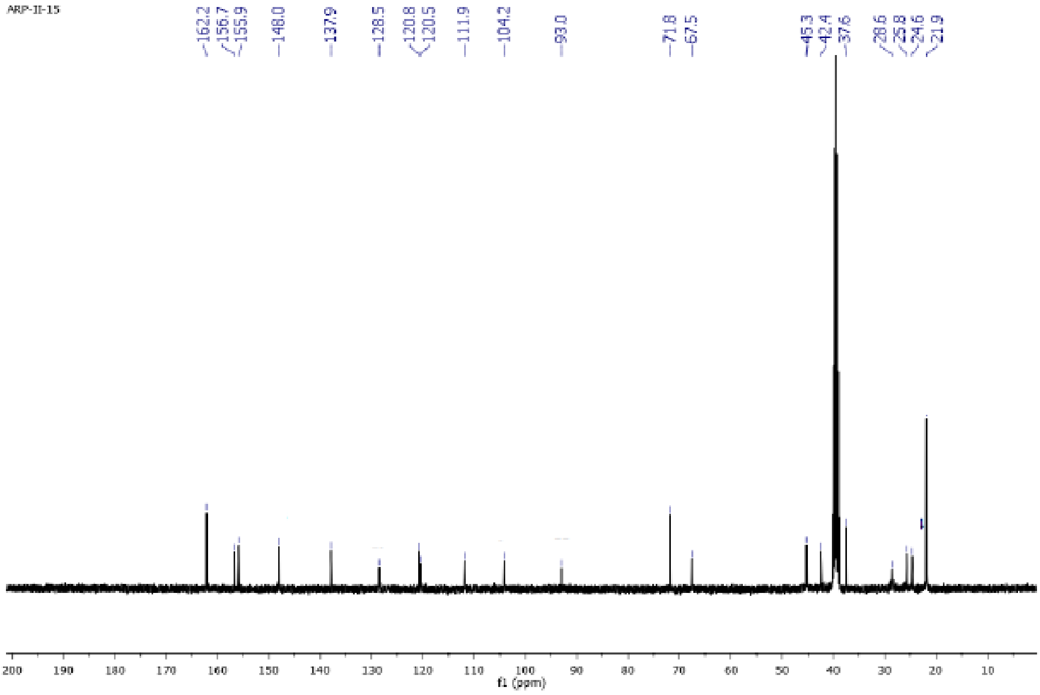

Compound 12m

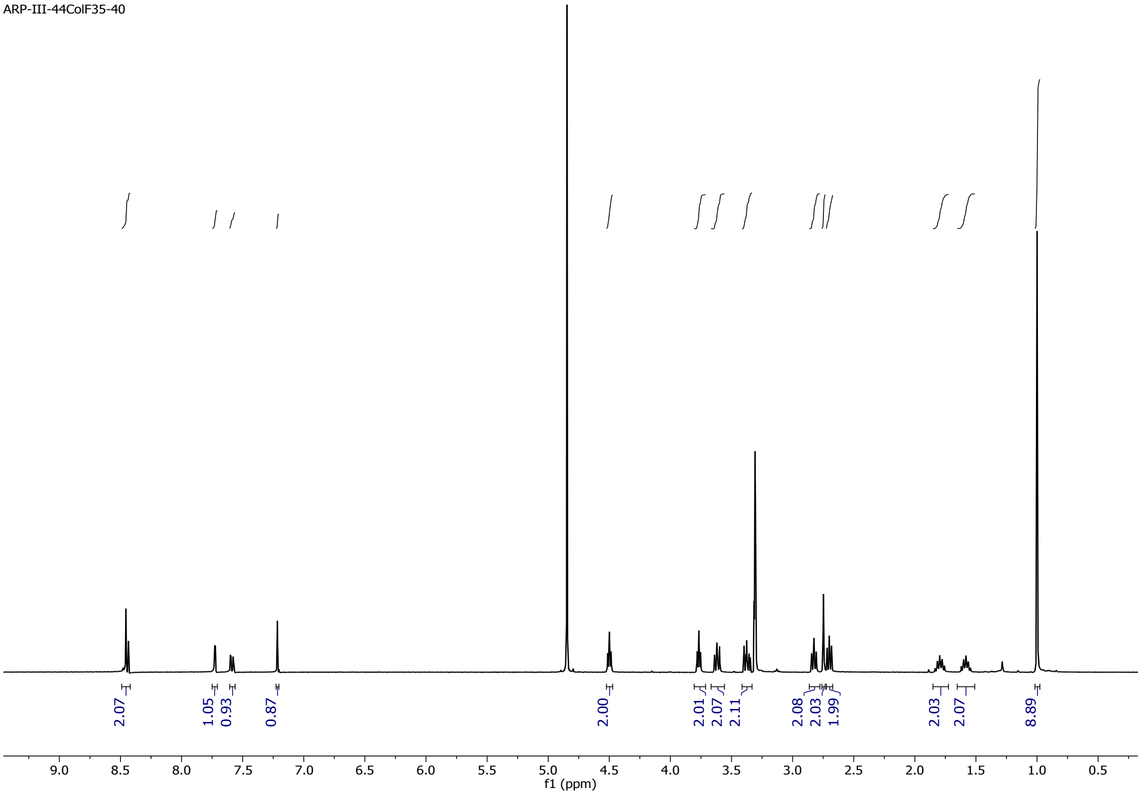

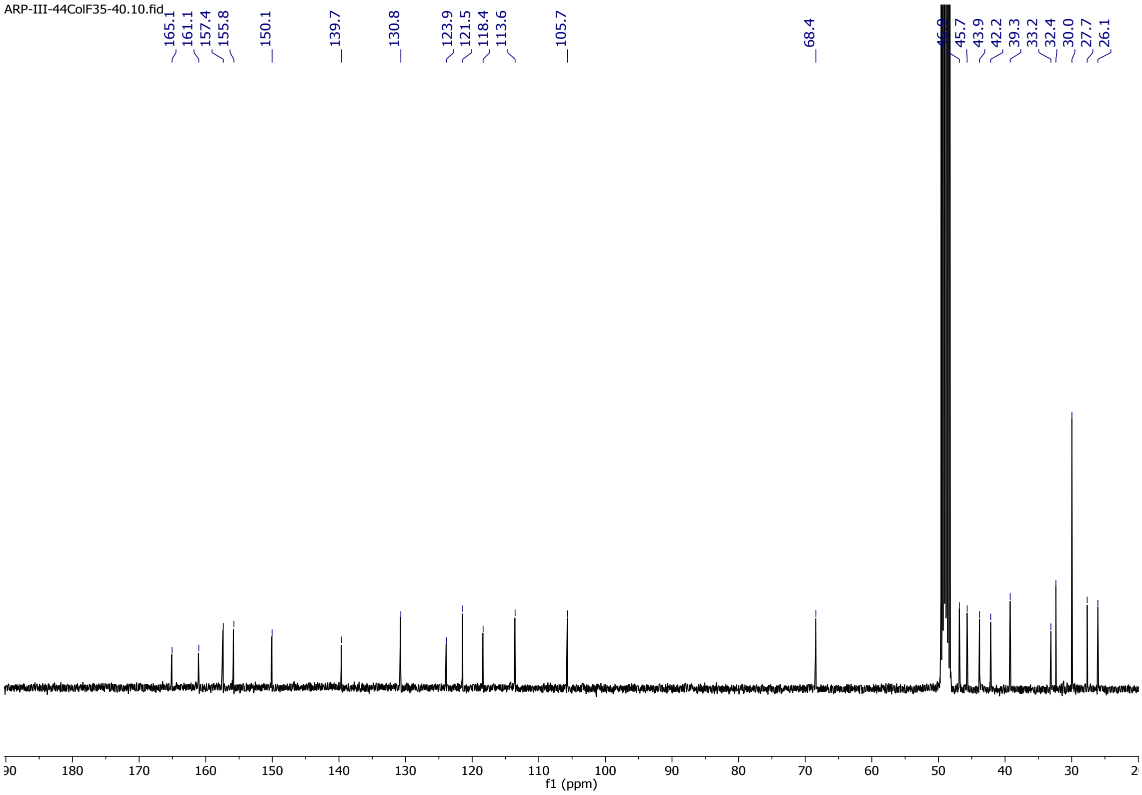

Compound 12n

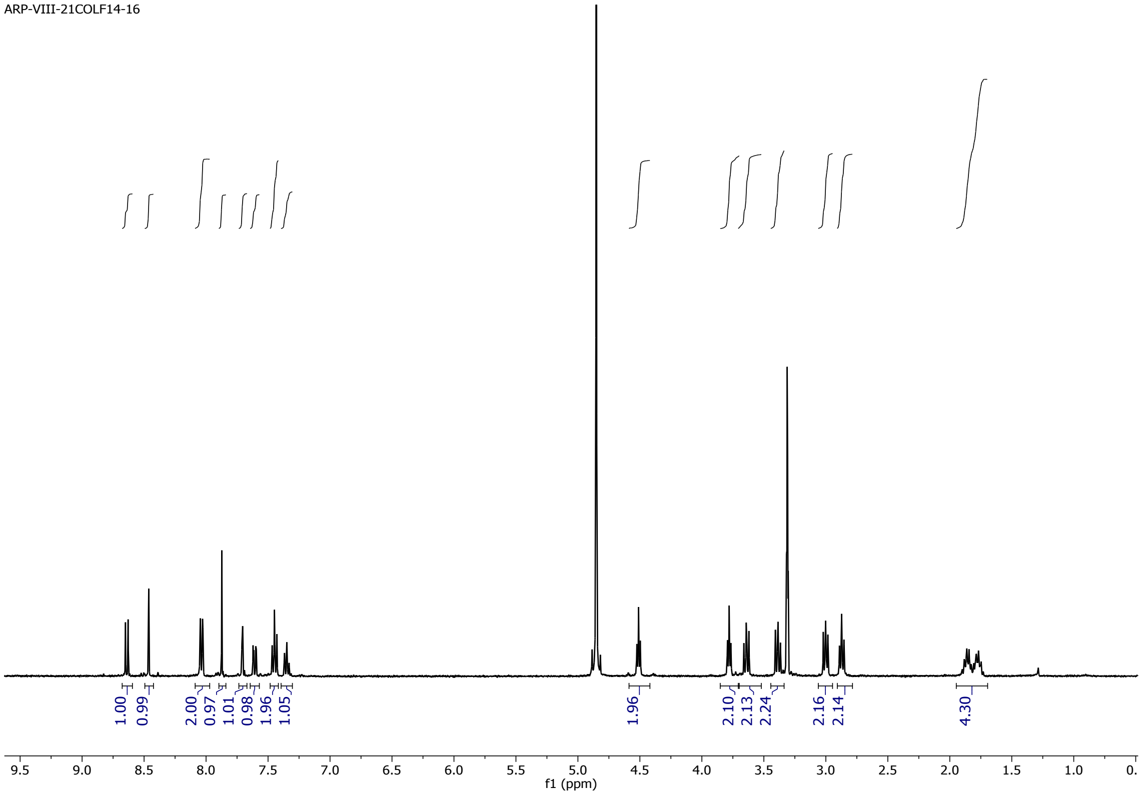

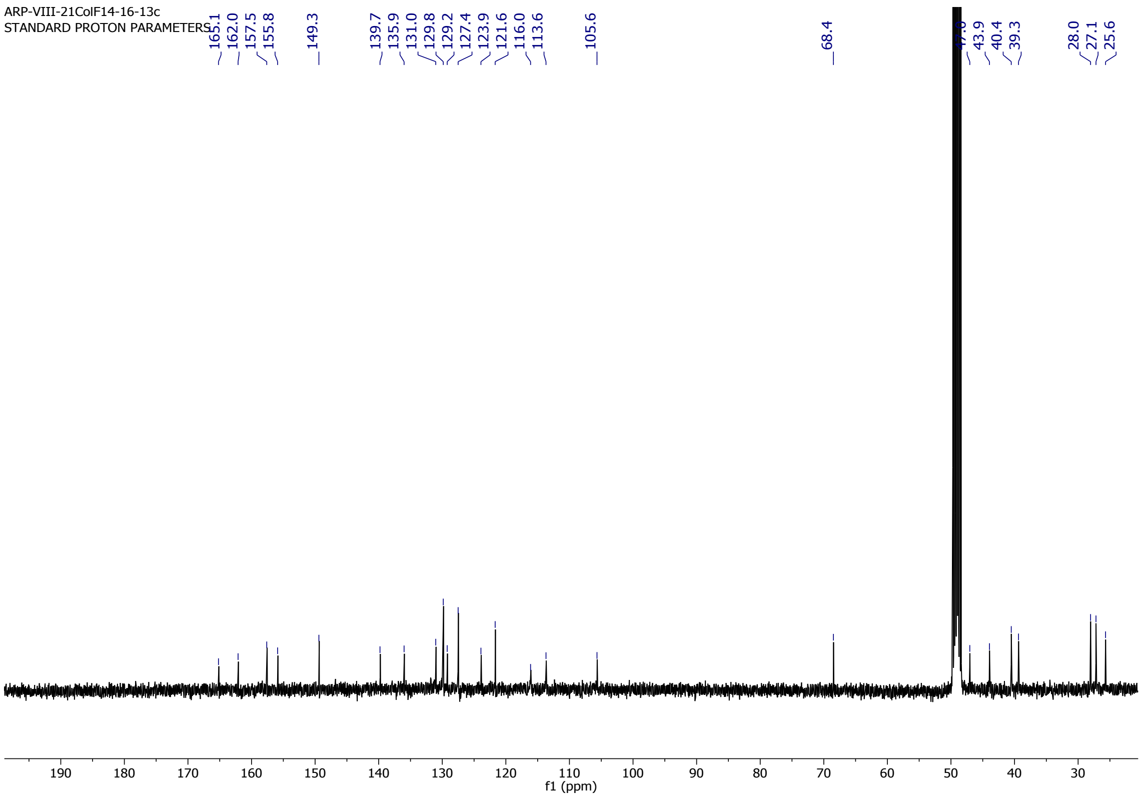

Compound 12o

ARP-II-27ColF42-43

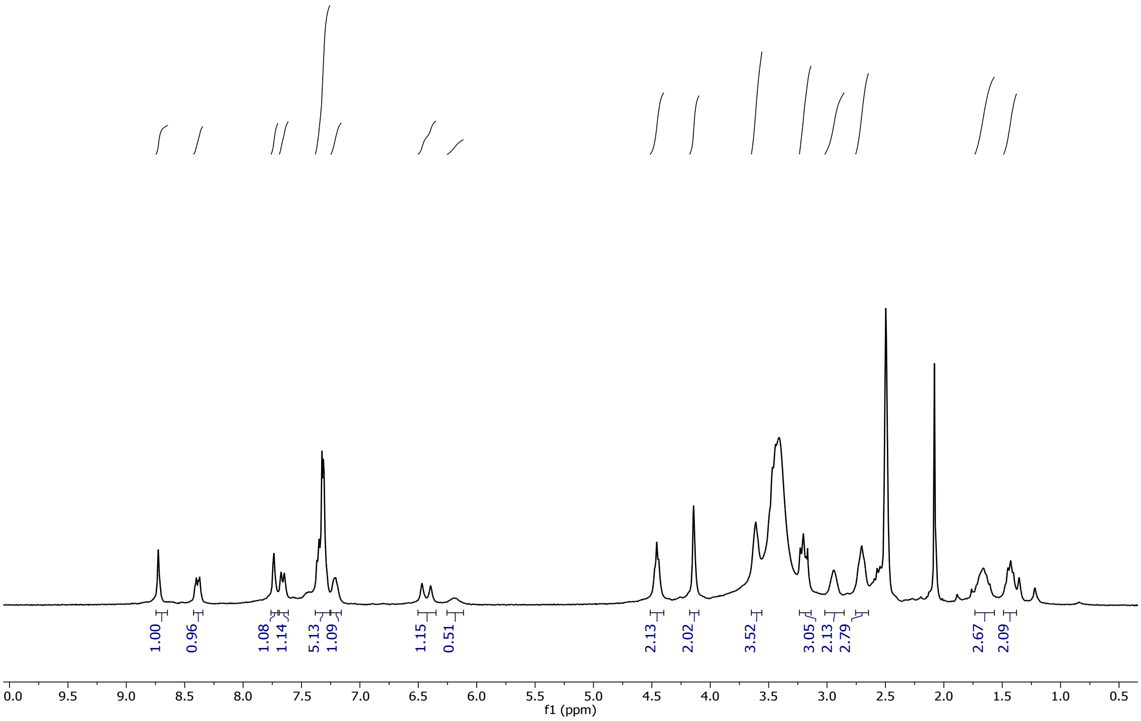

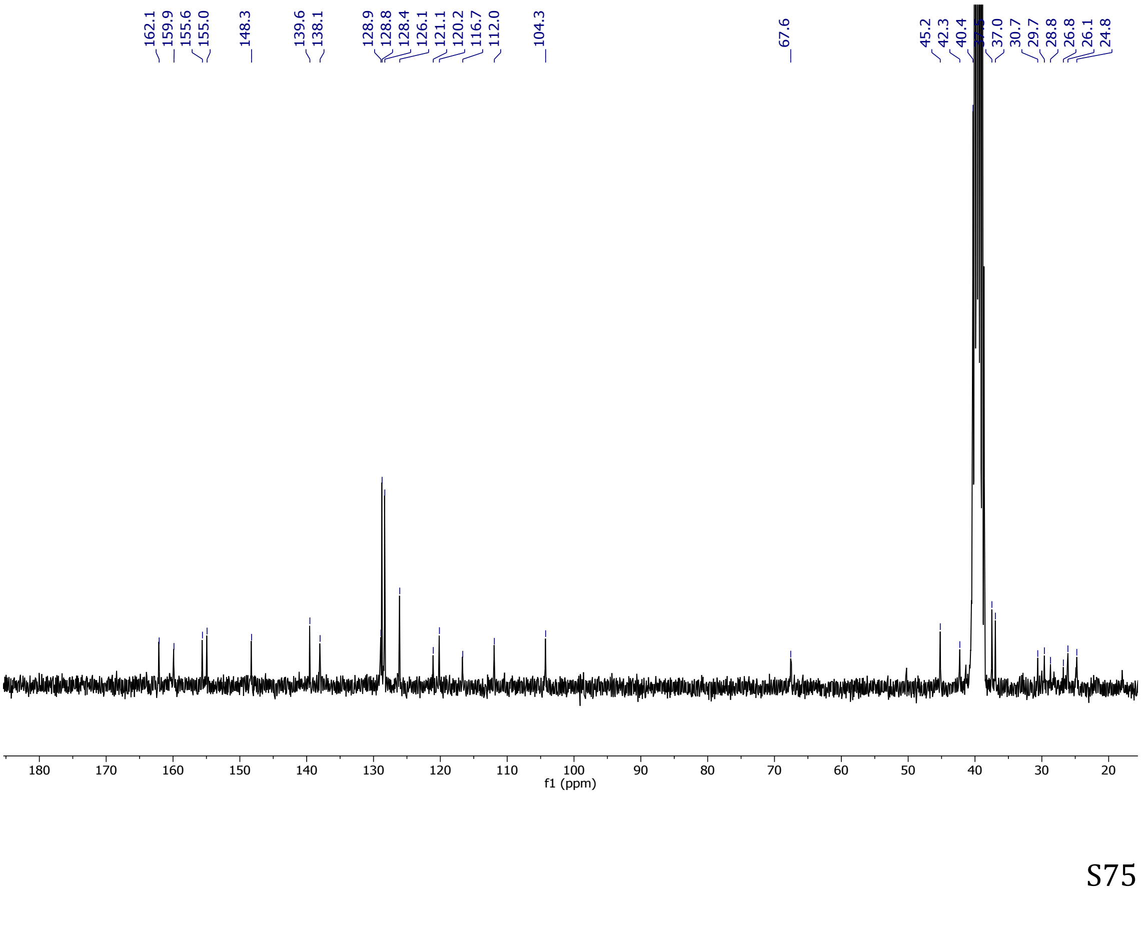

Compound 12p

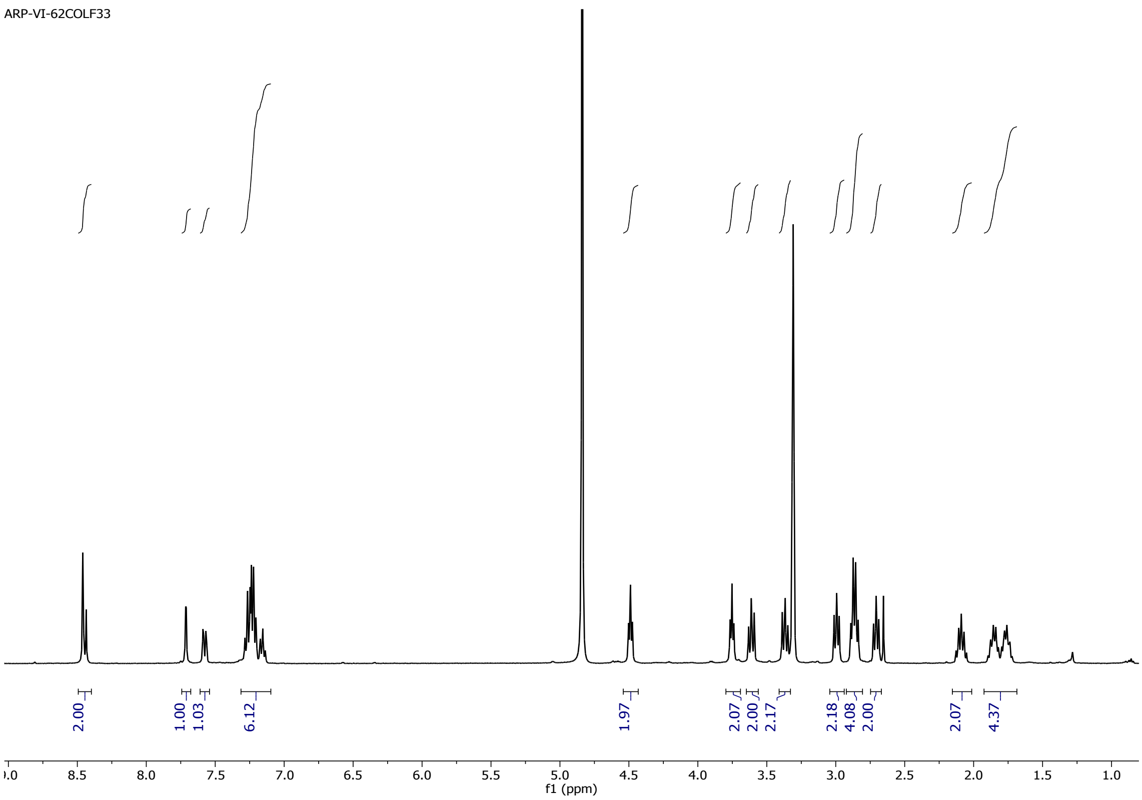

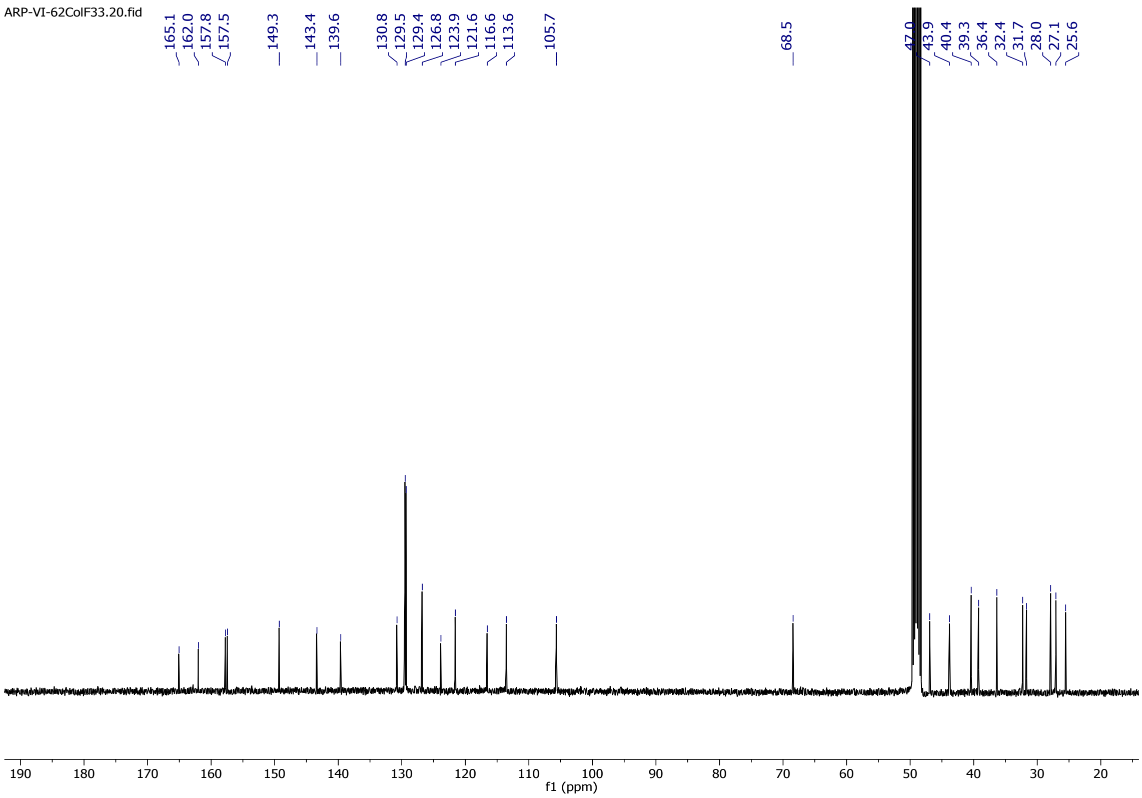

Compound 12q

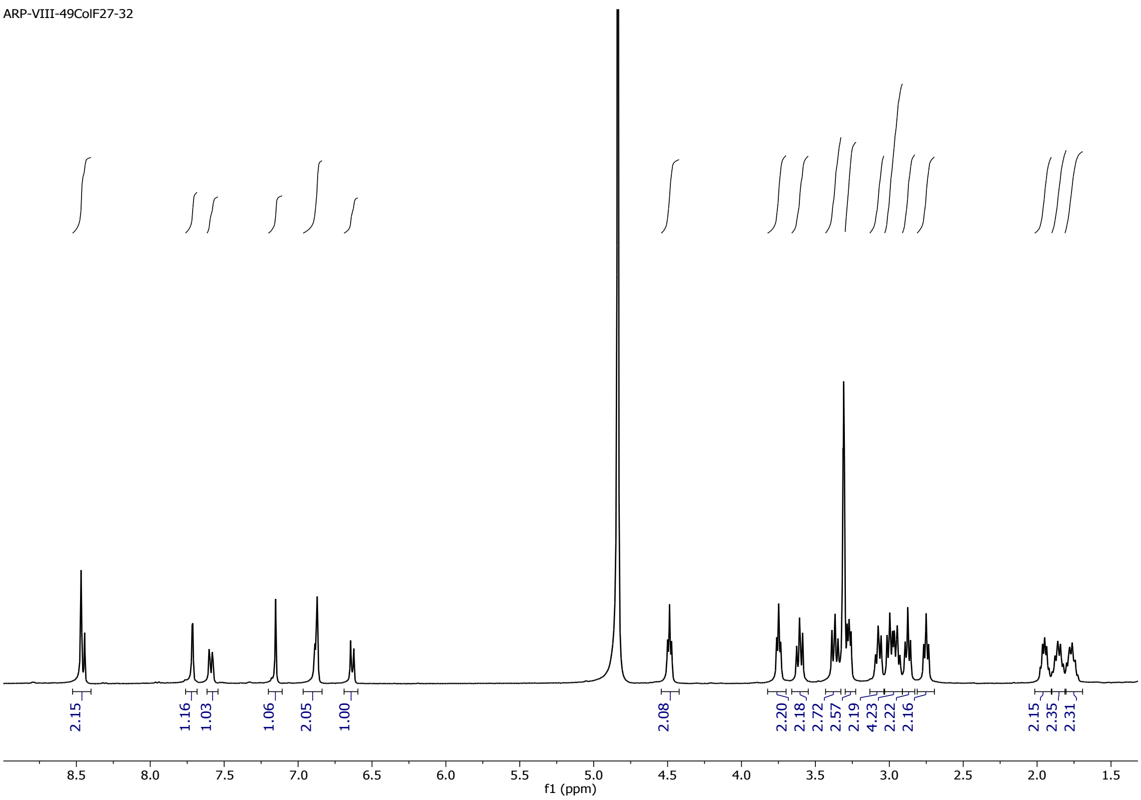

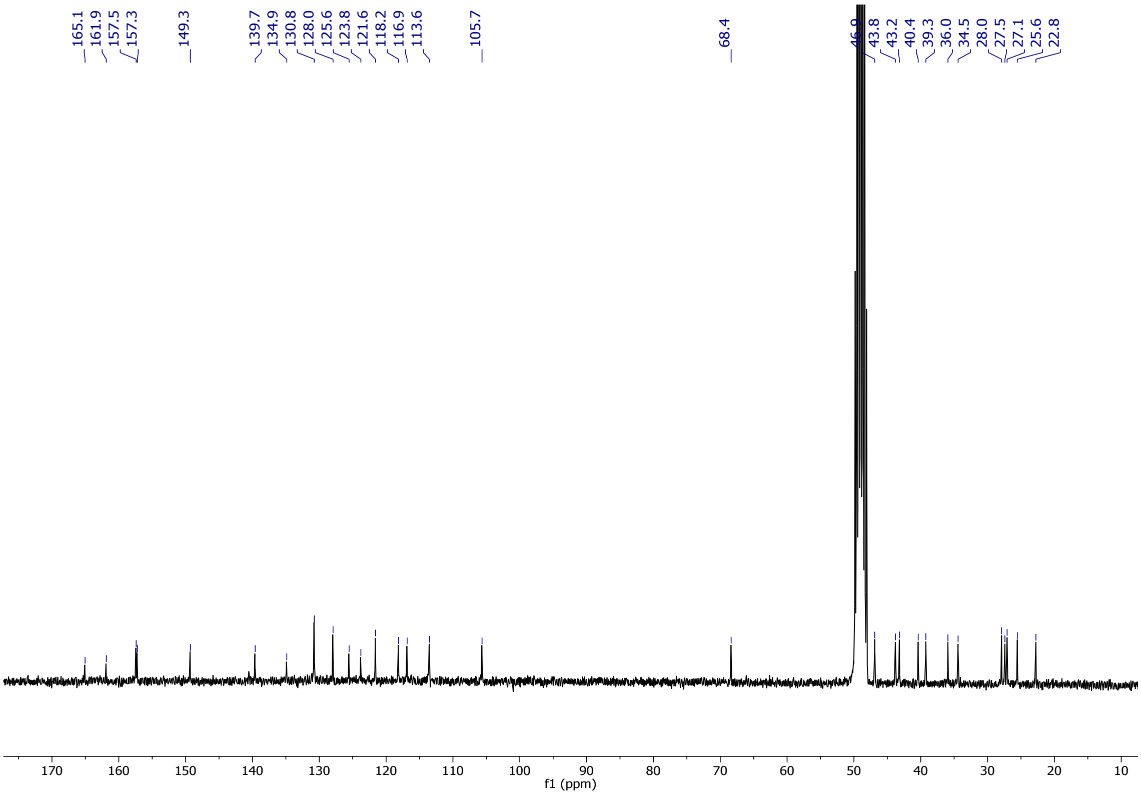

Compound 12r

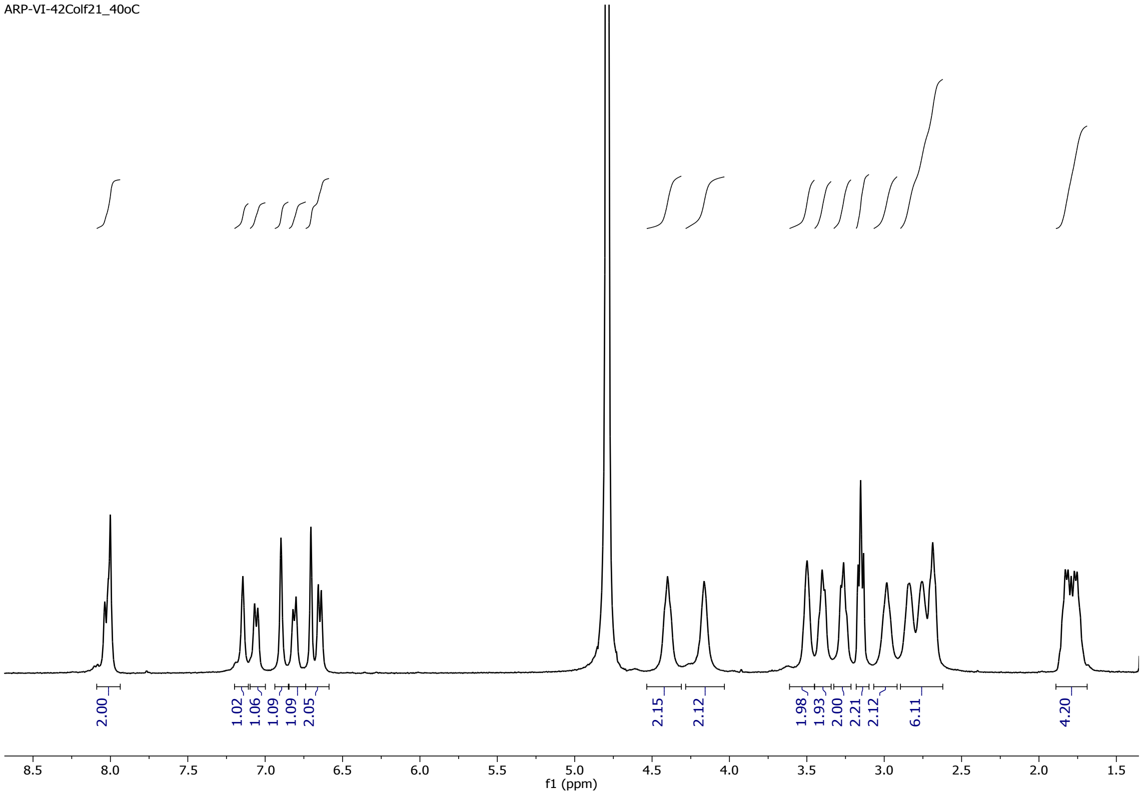

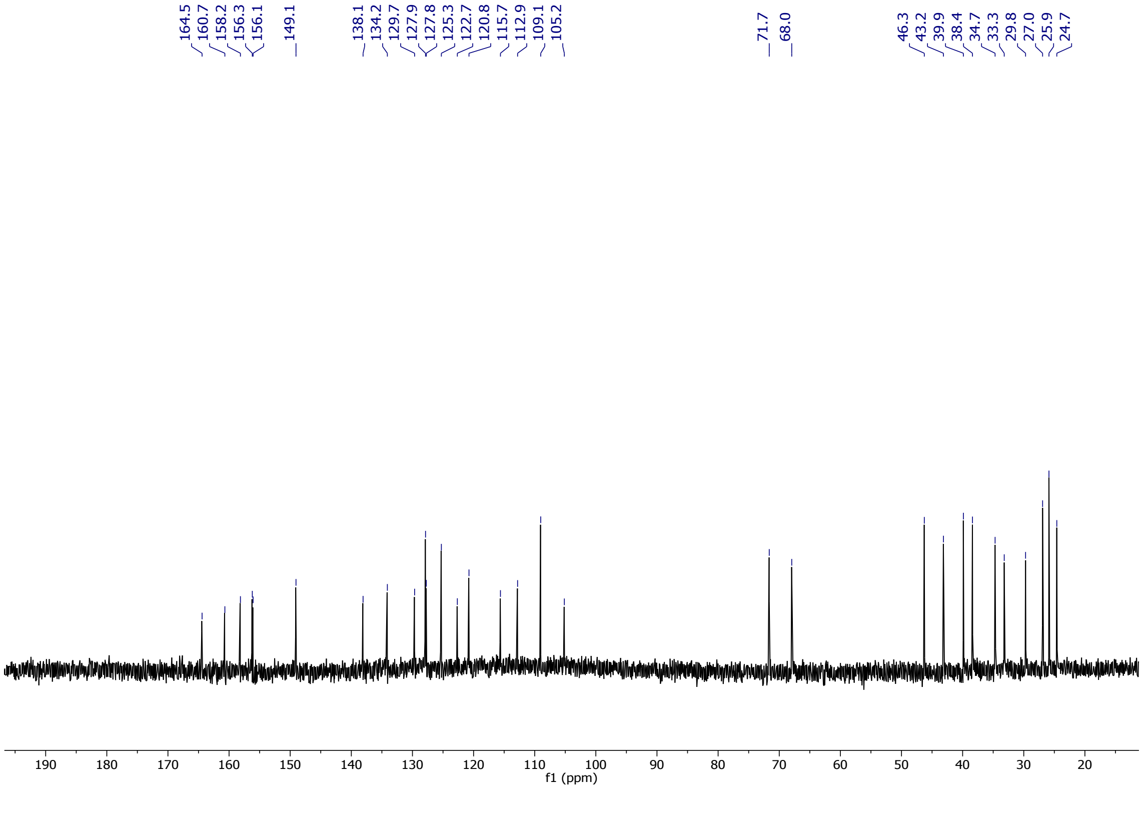

Compound 12s

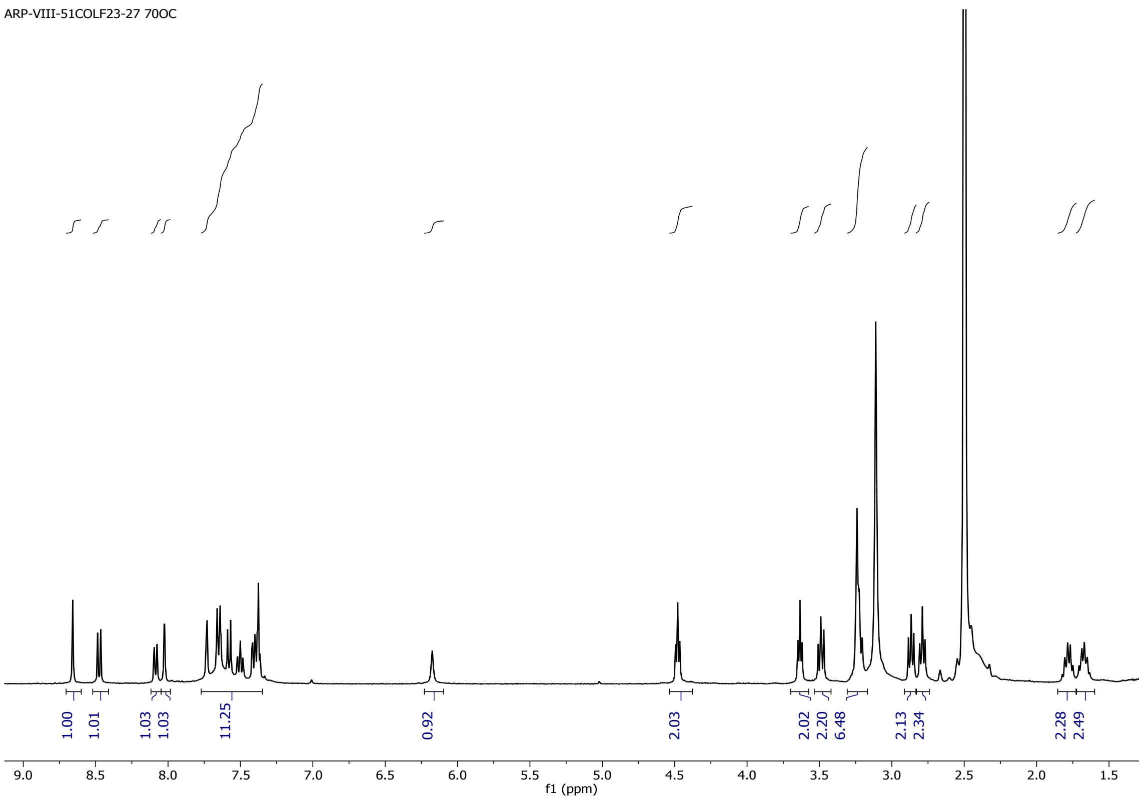

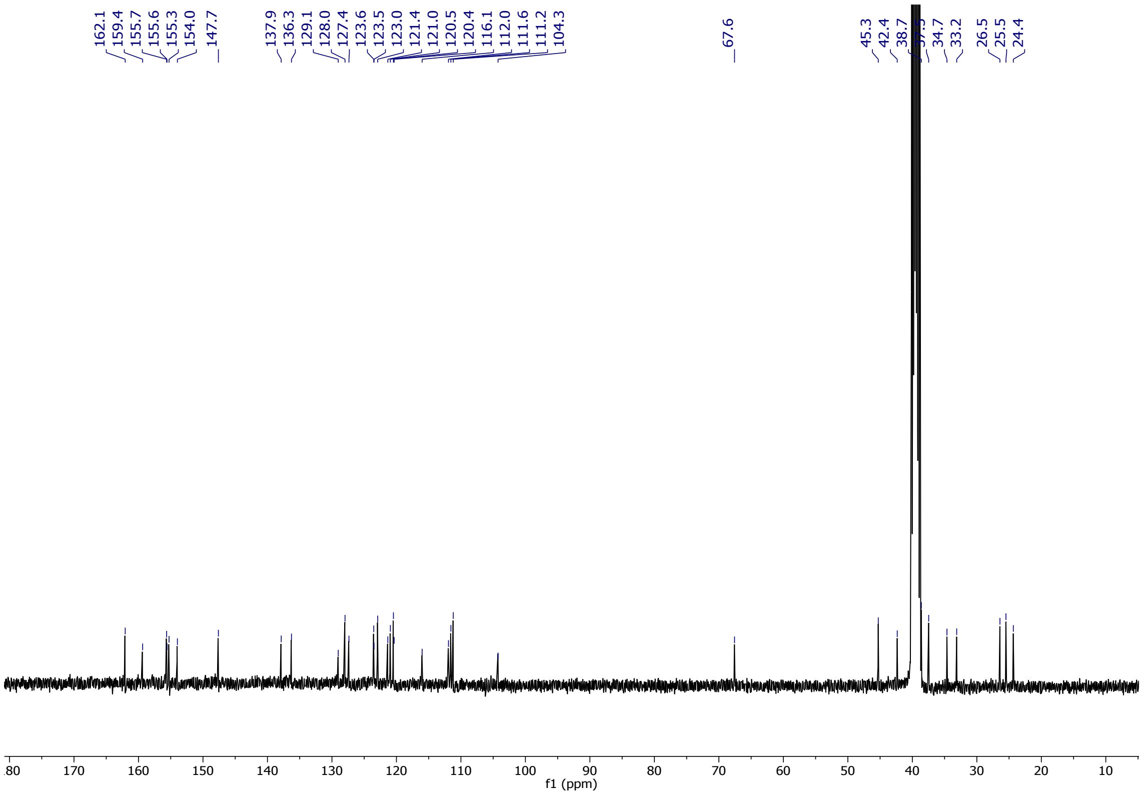

Truncated compound 13

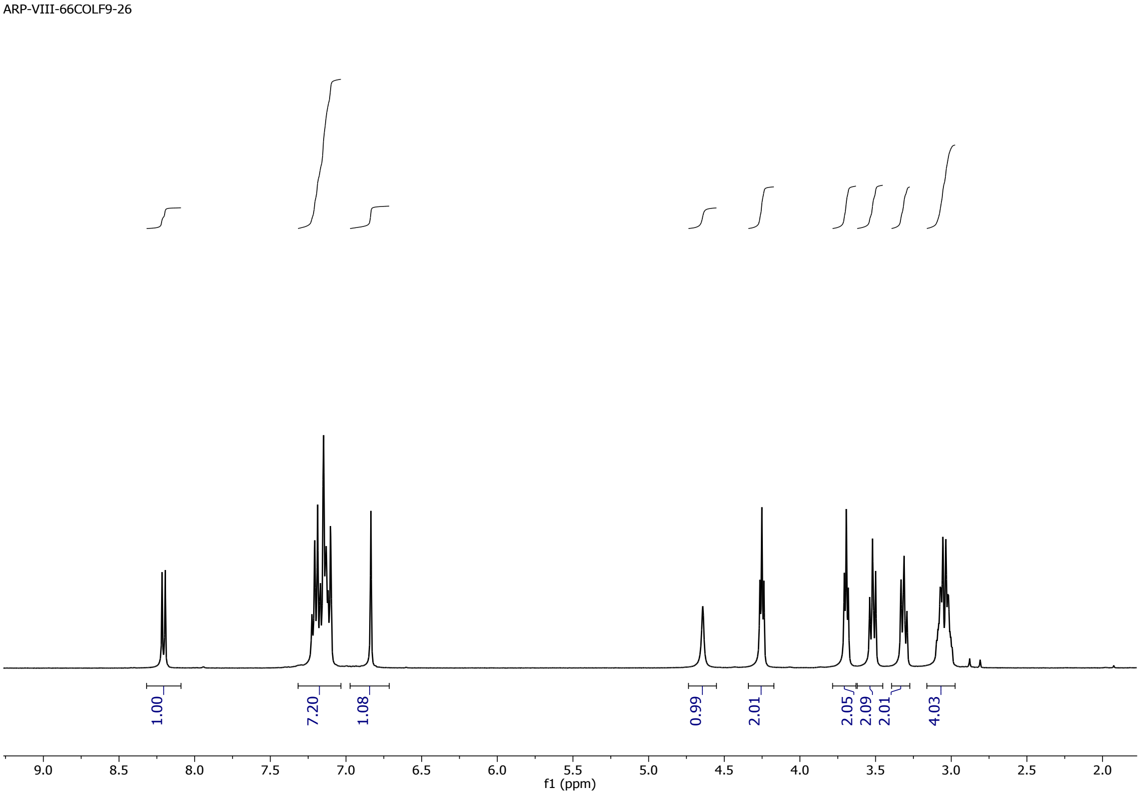

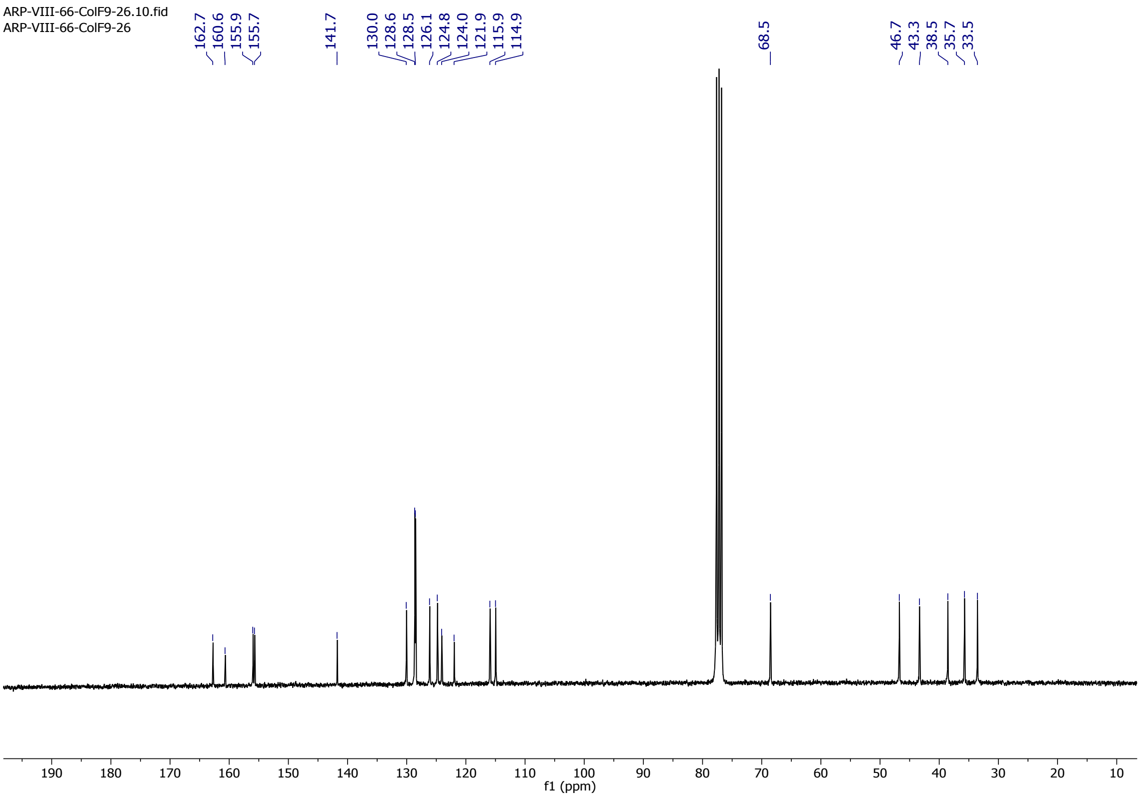

Truncated compound 14

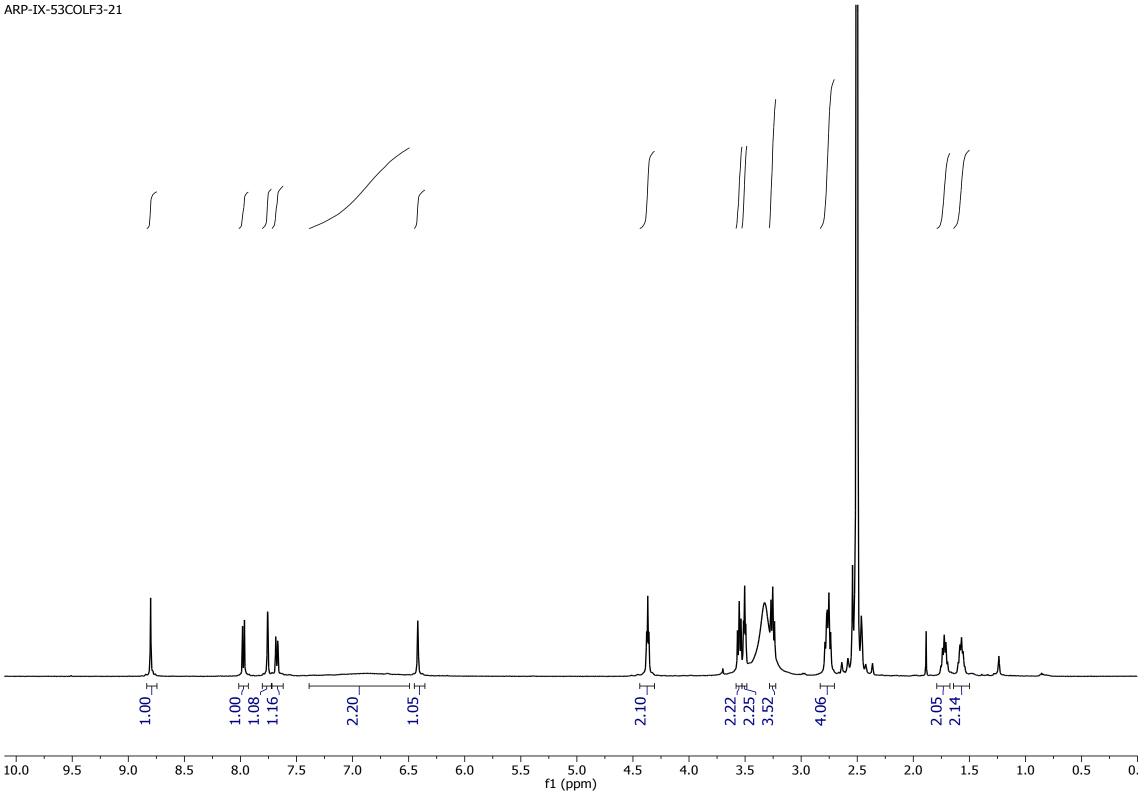

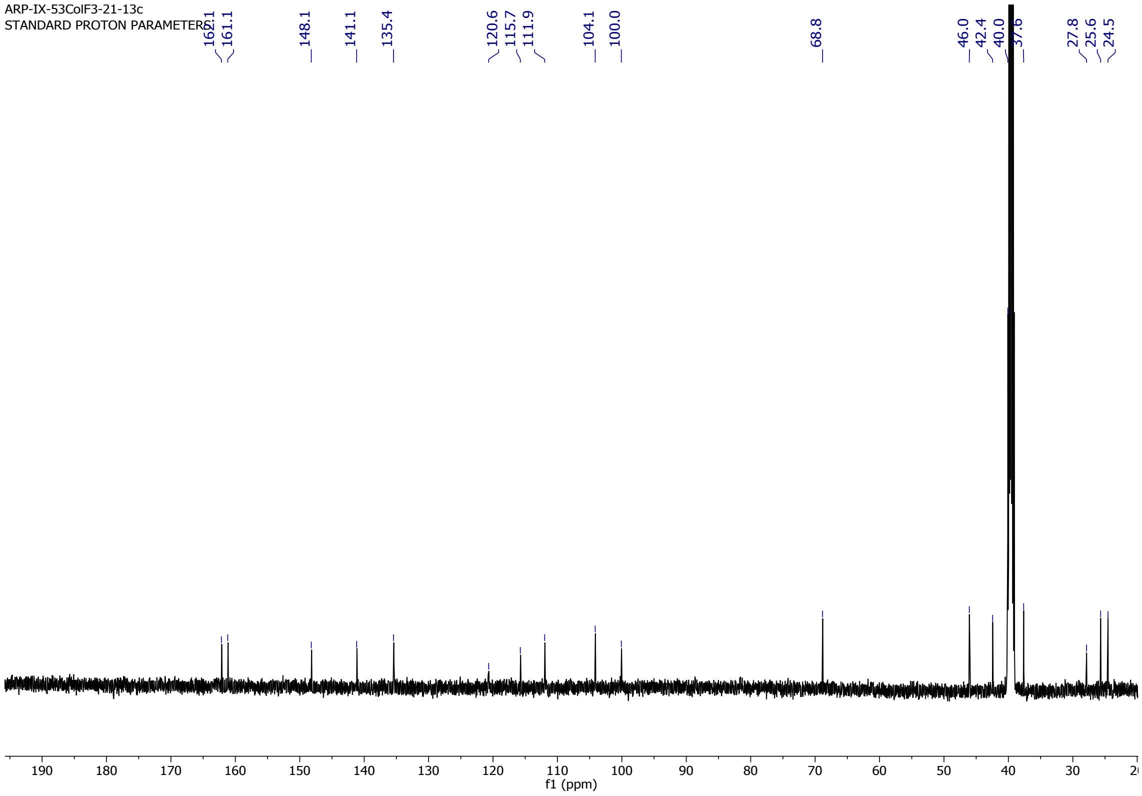

Compound 19a

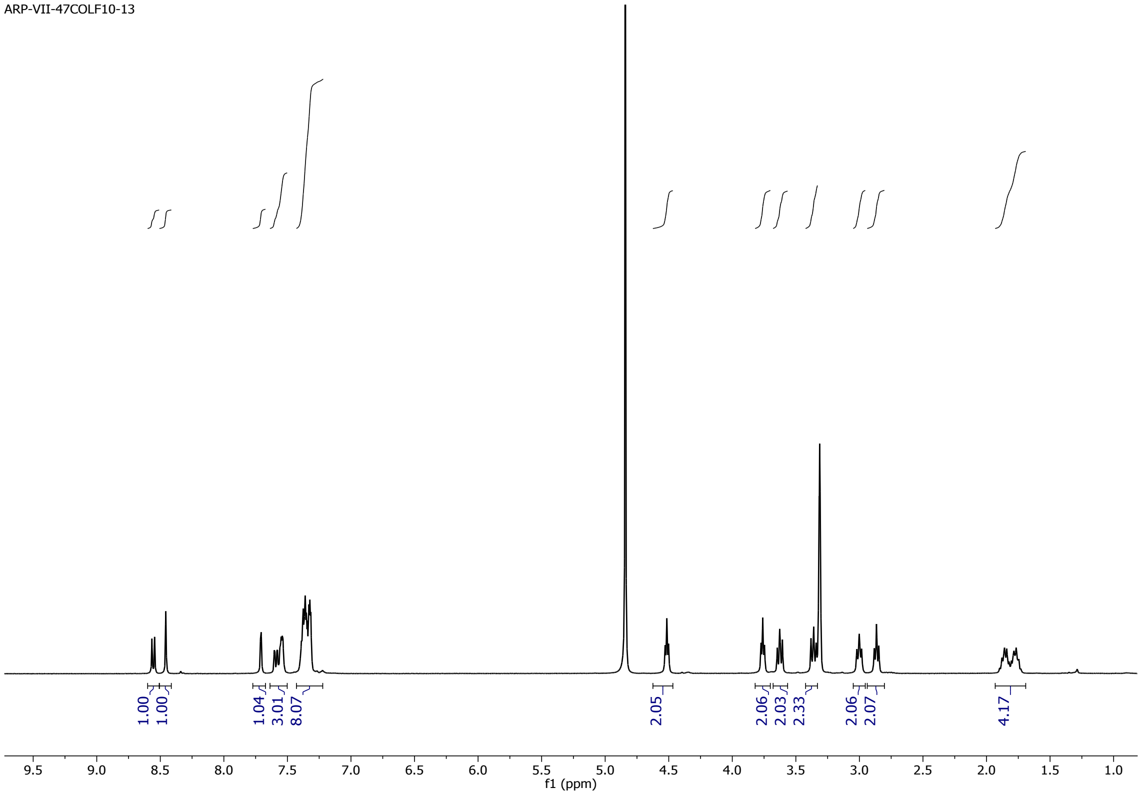

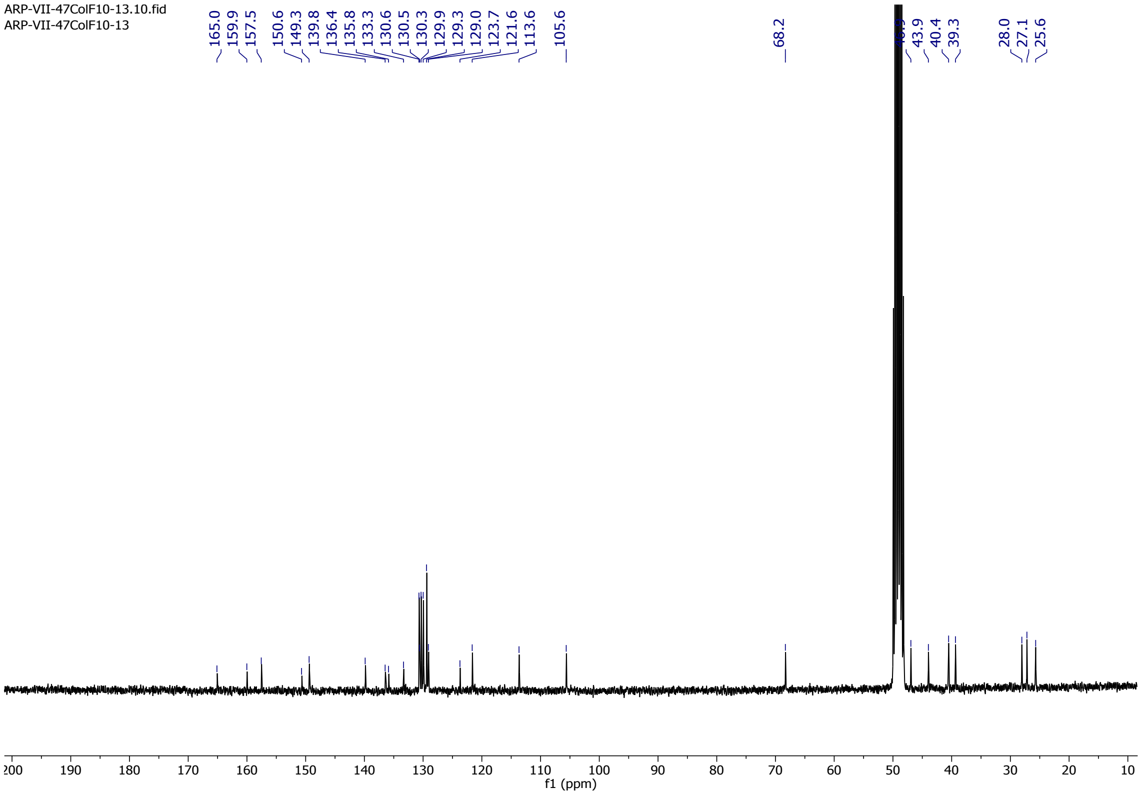

Compound 19b

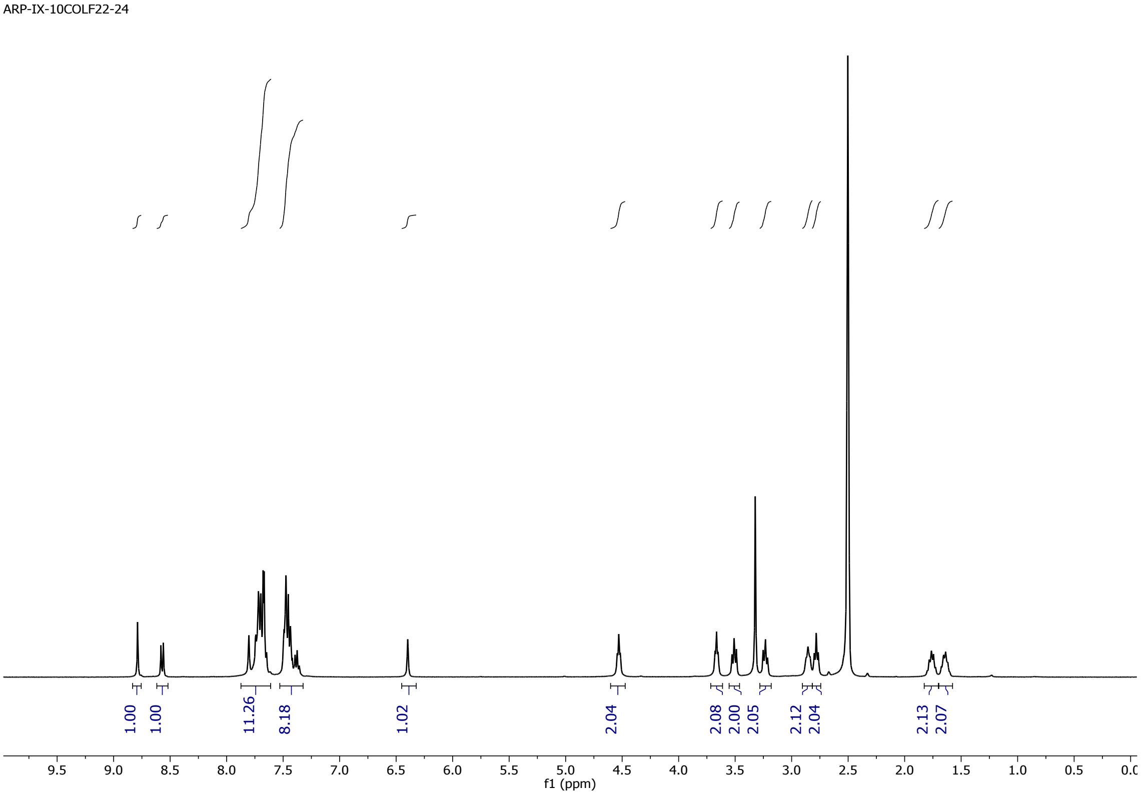

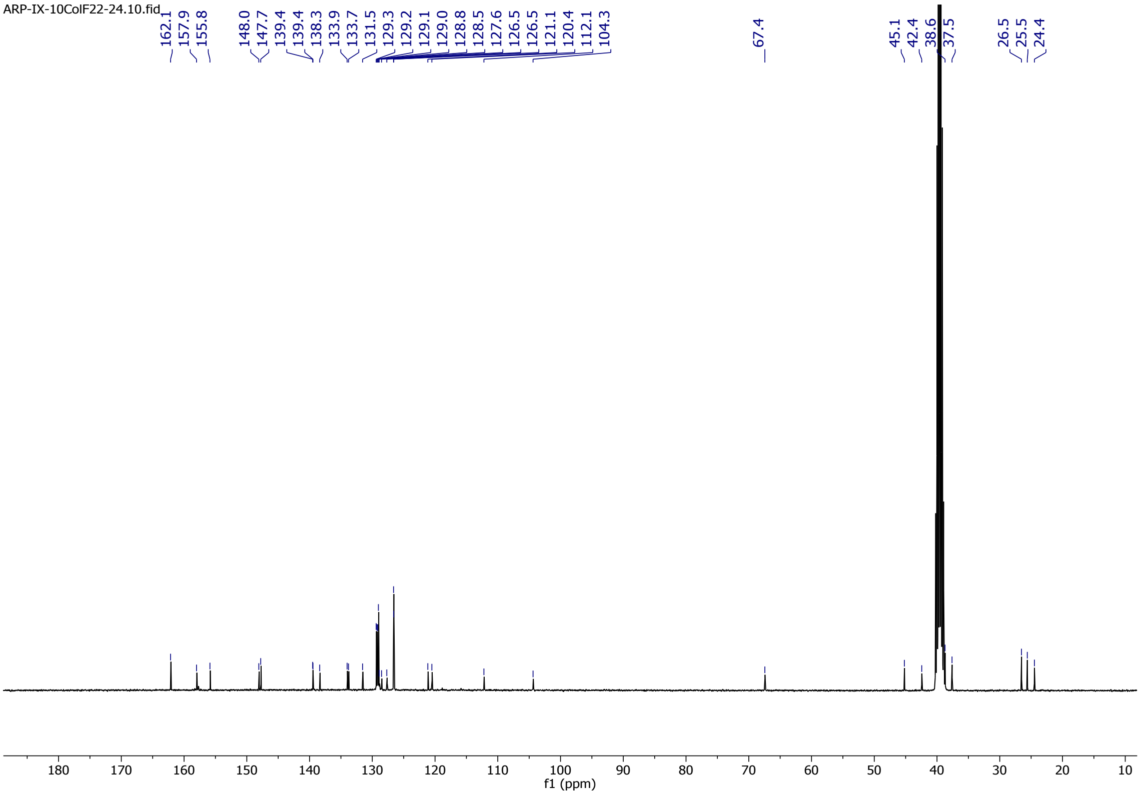

Compound 19c

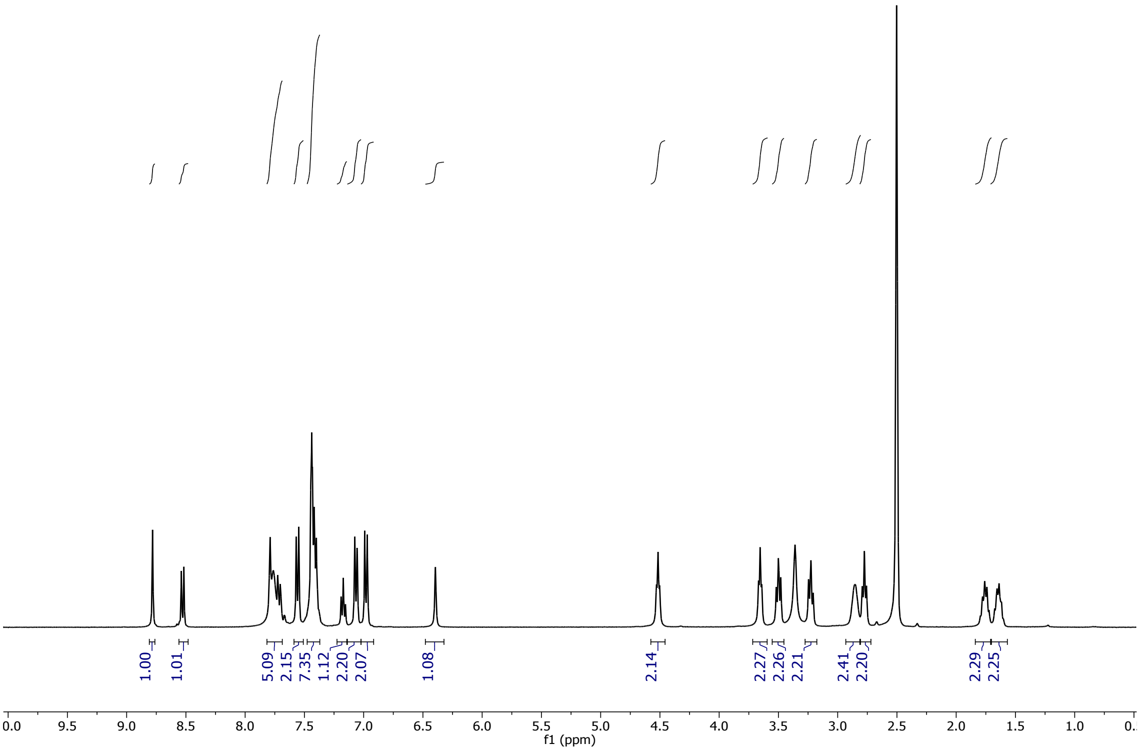

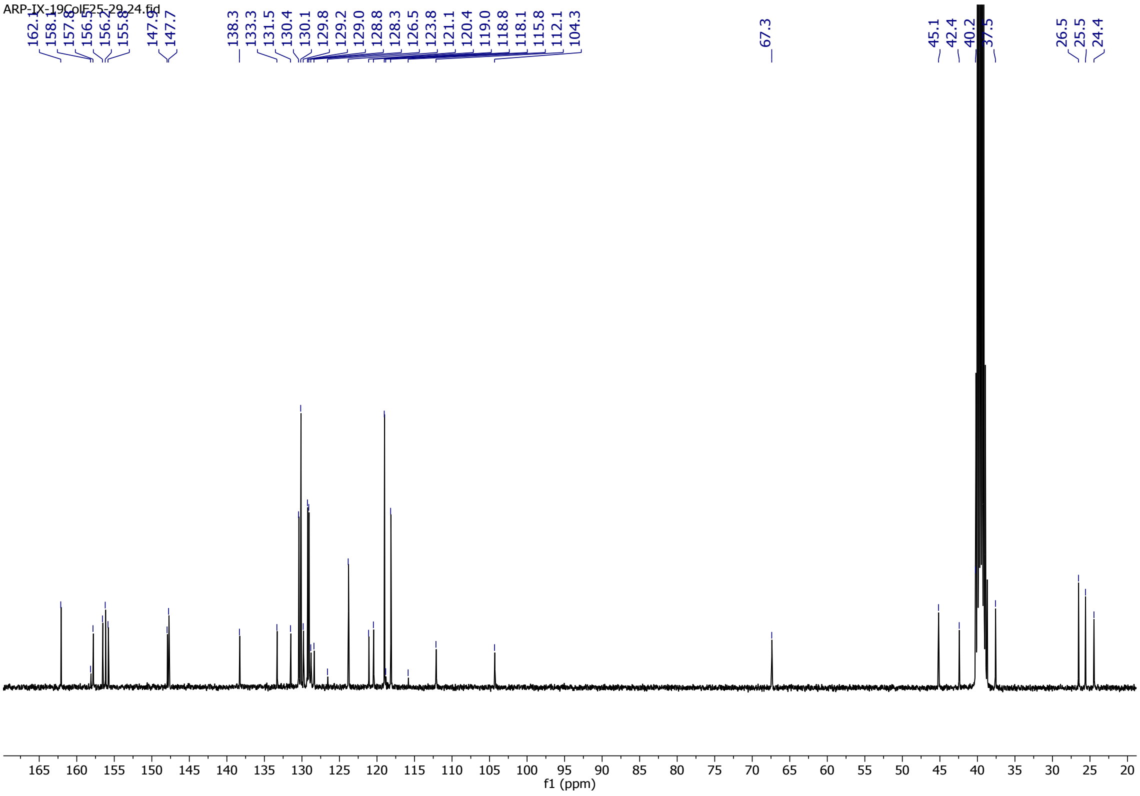

Compound 19d

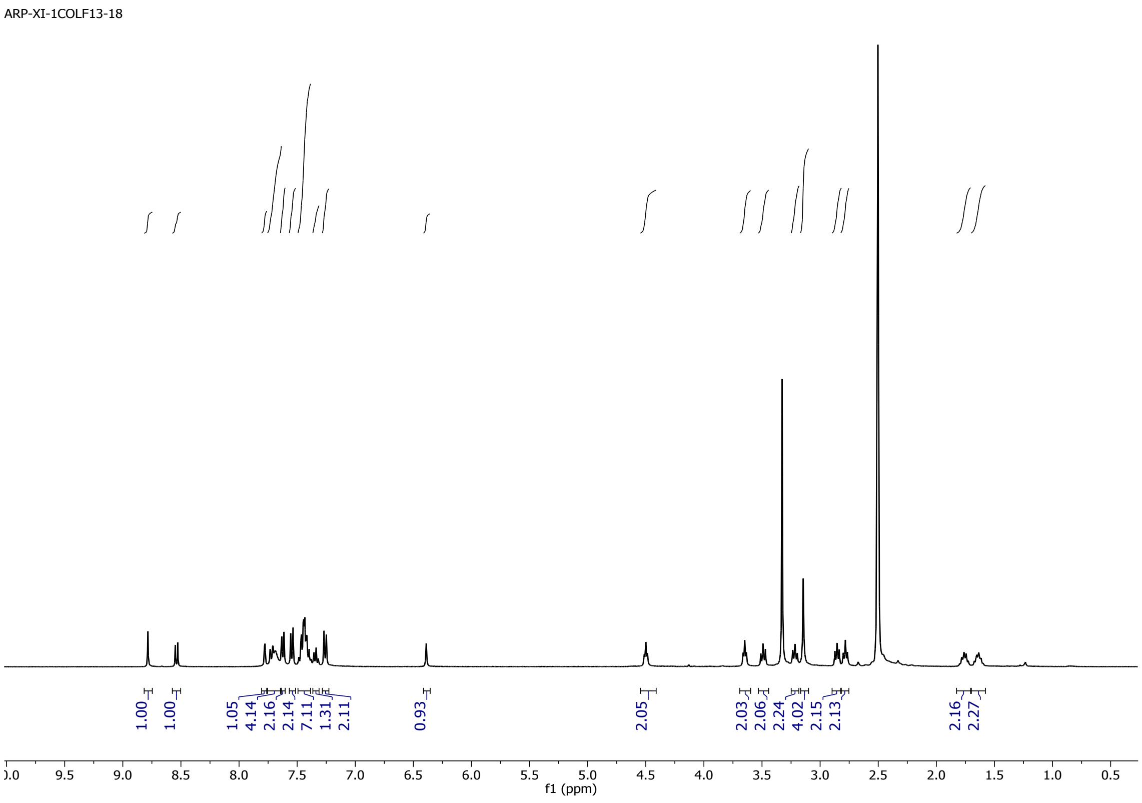

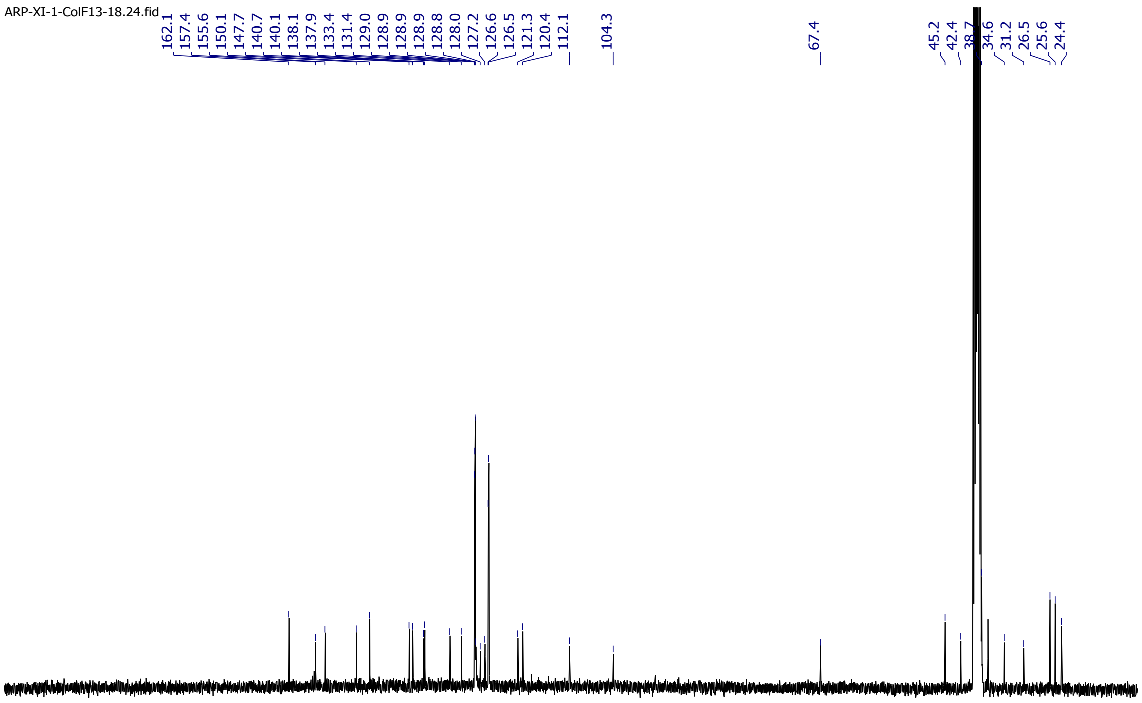

Compound 19e

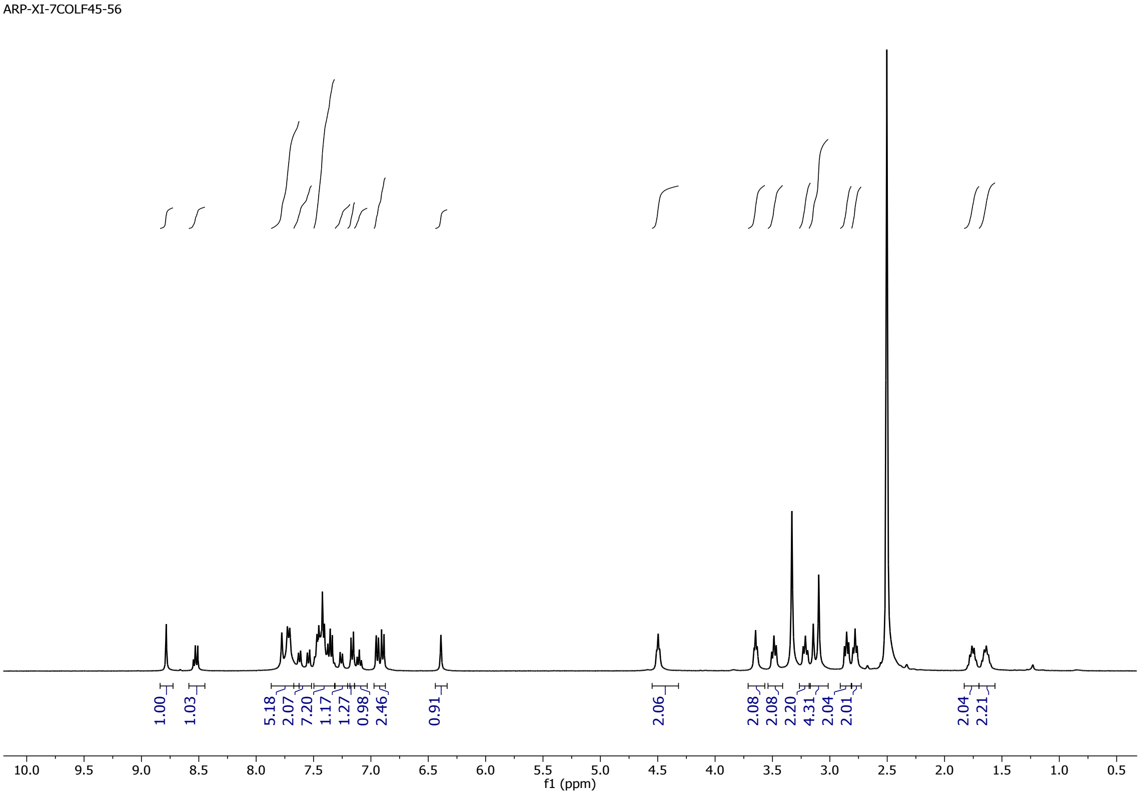

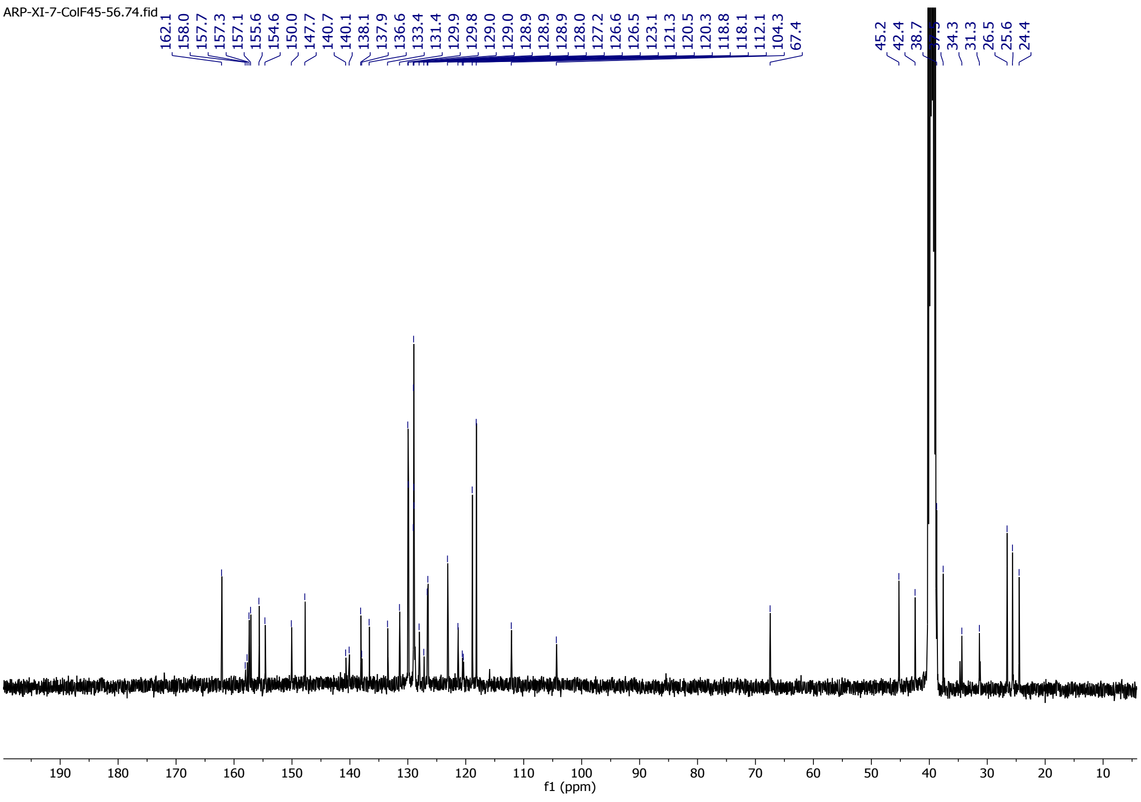

Symmetrical compound 22

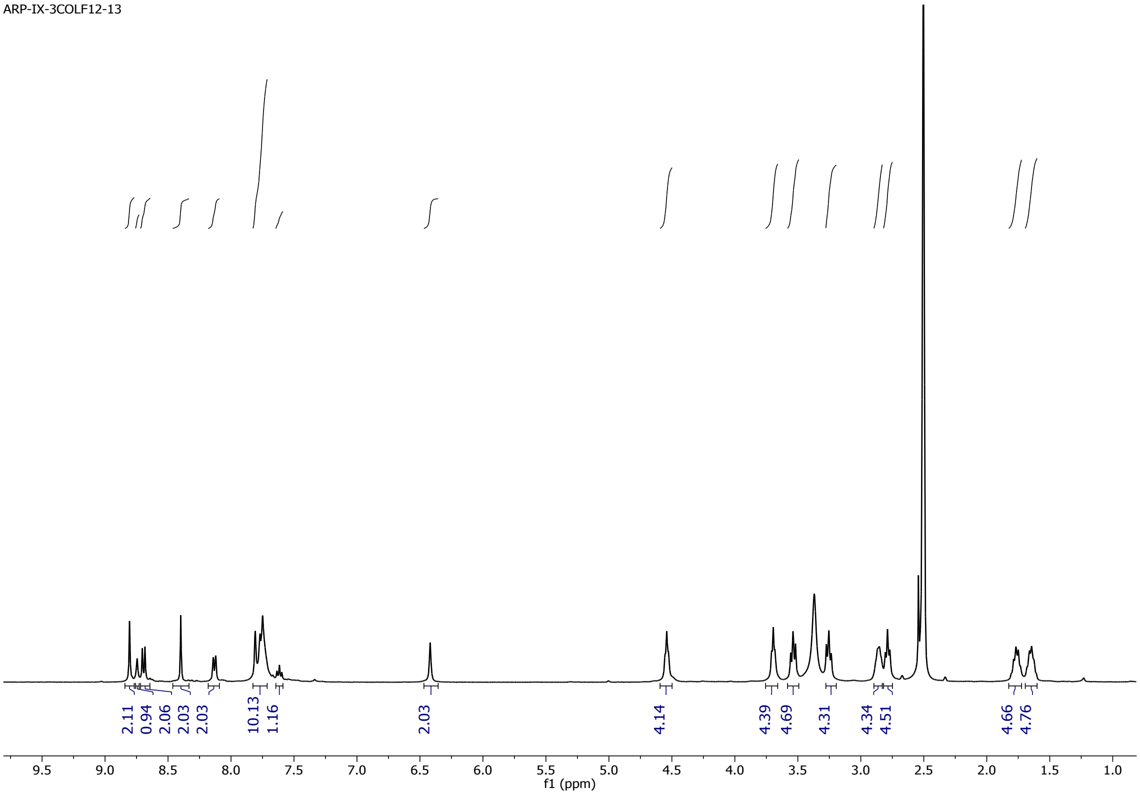

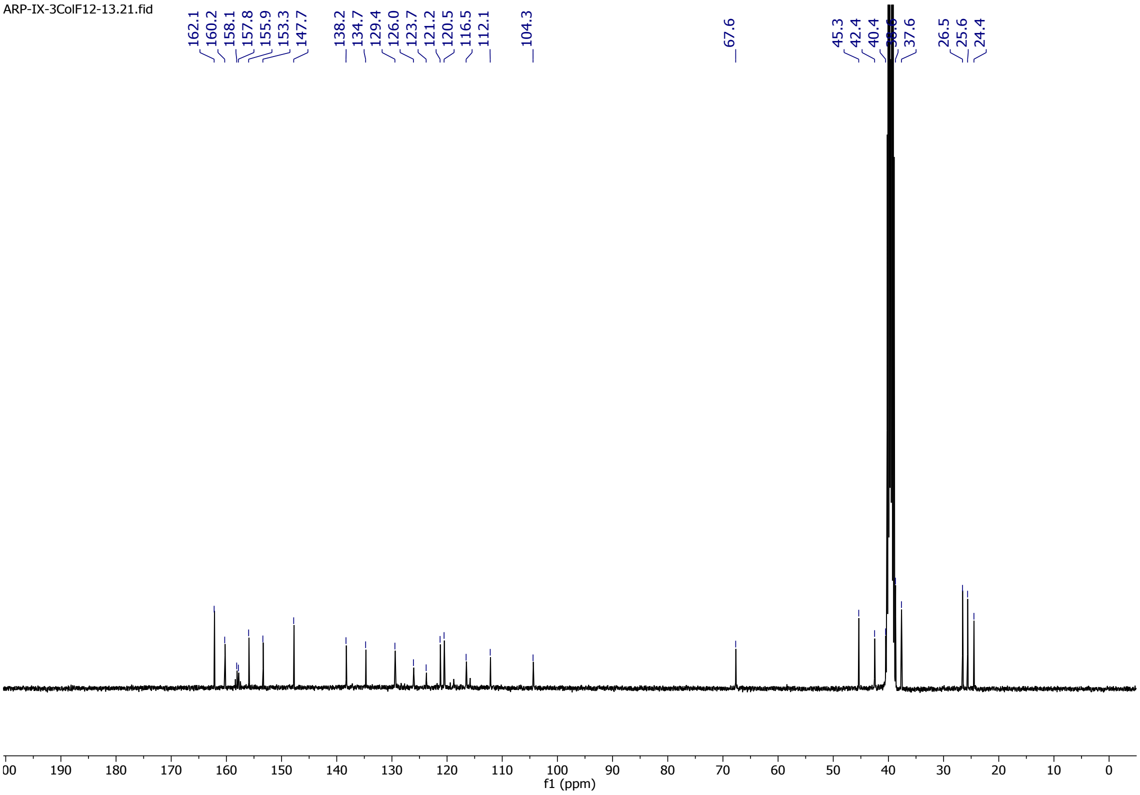

HRMS of final compounds

Compound 12a

Compound Table

Compound Label	RT	Mass	Abund	Formula	Tgt Mass	Diff (ppm)
Cpd 1: C28 H33 N7 O2 S	2.39	531.24236	6661	C28 H33 N7 O2 S	531.24164	1.35

Compound Label	RT	Algorithm	Mass
Cpd 1: C28 H33 N7 O2 S	2.39	Find By Formula	531.24236

MS Zoomed Spectrum

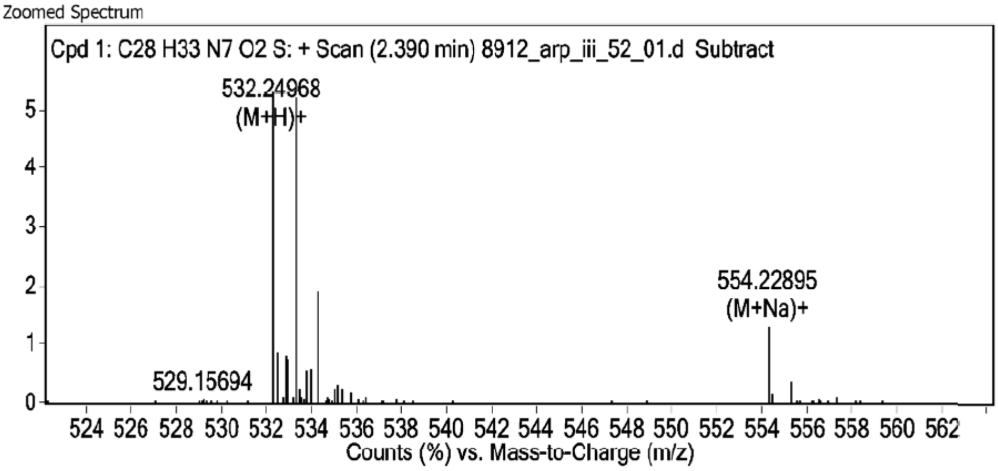

Compound 12b

Compound Table

Compound Label	RT	Mass	Abund	Formula	Tgt Mass	Diff (ppm)
Cpd 1: C32 H35 N7 O2 S	1.999	581.25722	96645	C32 H35 N7 O2 S	581.25729	-0.13

Compound Label	RT	Algorithm	Mass
Cpd 1: C32 H35 N7 O2 S	1.999	Find By Formula	581.25722

MS Zoomed Spectrum

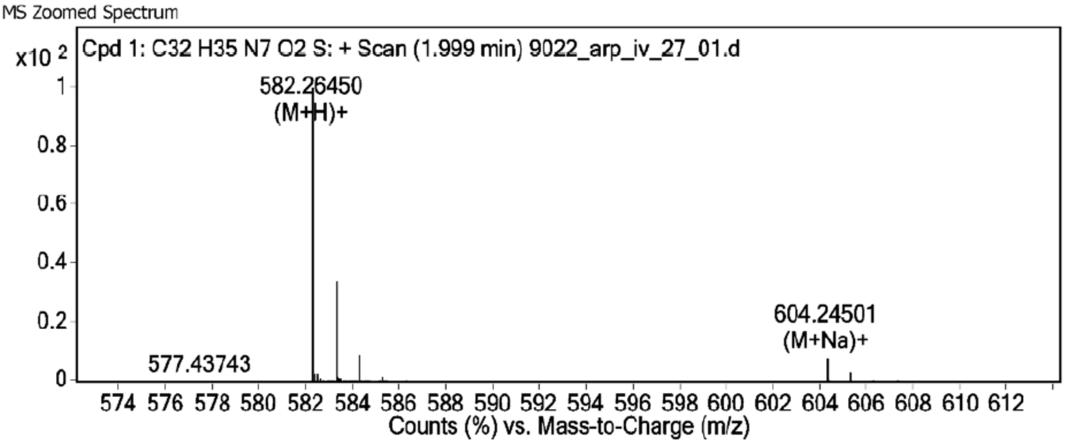

Compound 12c

Compound Table

Compound Label	RT	Mass	Abund	Formula	Tgt Mass	Diff (ppm)
Cpd 1: C34 H37 N7 O2 S	0.256	607.274	5729	C34 H37 N7 O2 S	607.27294	1.73

Compound Label	RT	Algorithm	Mass
Cpd 1: C34 H37 N7 O2 S	0.256	Find By Formula	607.274

MS Zoomed Spectrum

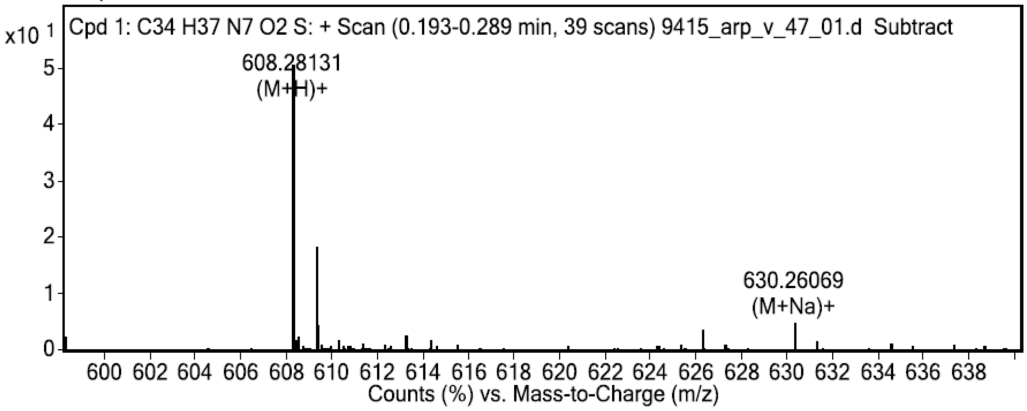

Compound 12d

Compound Table

Compound Label	RT	Mass	Abund	Formula	Tgt Mass	Diff (ppm)
Cpd 1: C28 H32 N6 O3 S	0.415	532.22575	198539	C28 H32 N6 O3 S	532.22566	0.17

Compound Label	RT	Algorithm	Mass
Cpd 1: C28 H32 N6 O3 S	0.415	Find By Formula	532.22575

MS Zoomed Spectrum

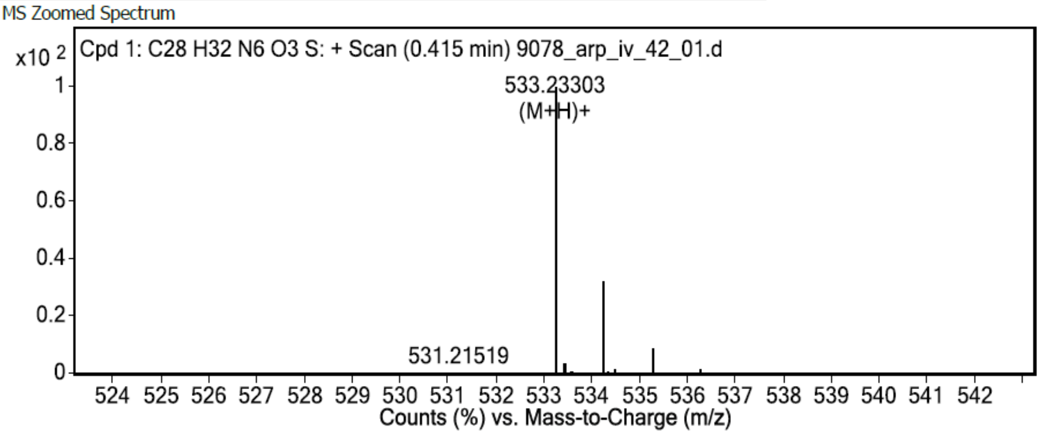

Compound 12e

Compound Table

Compound Label	RT	Mass	Abund	Formula	Tgt Mass	Diff (ppm)
Cpd 1: C28 H32 N6 O2 S	1.559	516.23101	129933	C28 H32 N6 O2 S	516.23075	0.52

Compound Label	RT	Algorithm	Mass
Cpd 1: C28 H32 N6 O2 S	1.559	Find By Formula	516.23101

MS Zoomed Spectrum

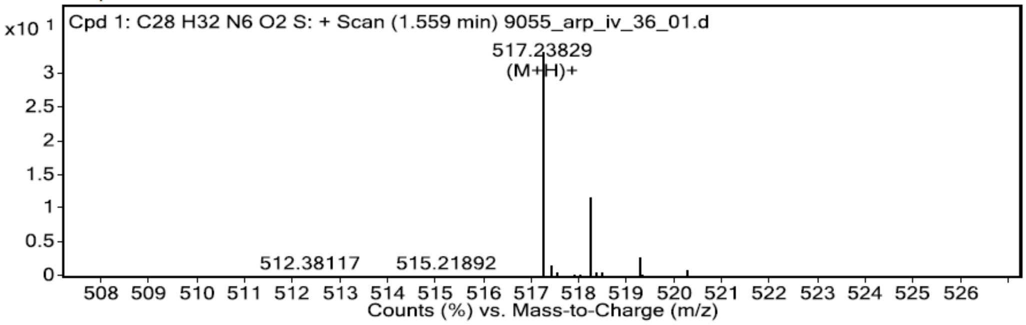

Compound 12f

Compound Table

Compound Label	RT	Mass	Abund	Formula	Tgt Mass	Diff (ppm)
Cpd 1: C34 H36 N6 O2 S	0.267	592.26096	32289	C34 H36 N6 O2 S	592.26205	-1.83

Compound Label	RT	Algorithm	Mass
Cpd 1: C34 H36 N6 O2 S	0.267	Find By Formula	592.26096

MS Zoomed Spectrum

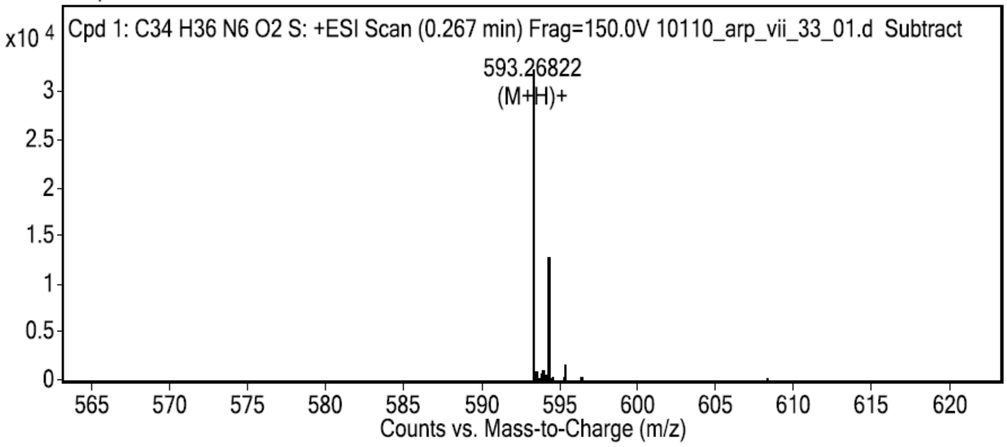

Compound 12g

Compound Table

Compound Label	RT	Mass	Abund	Formula	Tgt Mass	Diff (ppm)
Cpd 1: C28 H30 N6 O4 S	0.199	546.20591	20993	C28 H30 N6 O4 S	546.20492	1.81

Compound Label	RT	Algorithm	Mass
Cpd 1: C28 H30 N6 O4 S	0.199	Find By Formula	546.20591

MS Zoomed Spectrum

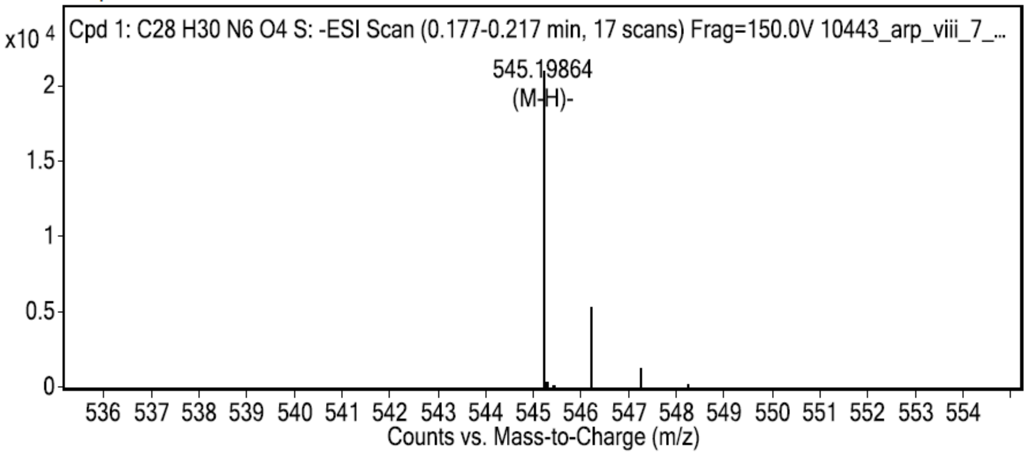

Compound 12h

Compound Table

Compound Label	RT	Mass	Abund	Formula	Tgt Mass	Diff (ppm)
Cpd 1: C27 H31 N7 O2 S	2.666	517.2266	123330	C27 H31 N7 O2 S	517.22599	1.17

Compound Label	RT	Algorithm	Mass
Cpd 1: C27 H31 N7 O2 S	2.666	Find By Formula	517.2266

MS Zoomed Spectrum

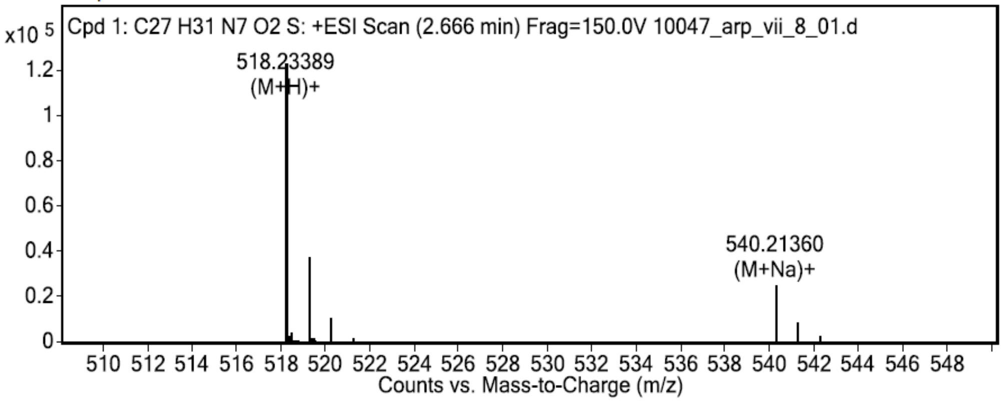

Compound 12i

Compound Table

Compound Label	RT	Mass	Abund	Formula	Tgt Mass	Diff (ppm)
Cpd 1: C33 H35 N7 O2 S	1.073	593.25724	35187	C33 H35 N7 O2 S	593.25729	-0.09

Compound Label	RT	Algorithm	Mass
Cpd 1: C33 H35 N7 O2 S	1.073	Find By Formula	593.25724

MS Zoomed Spectrum

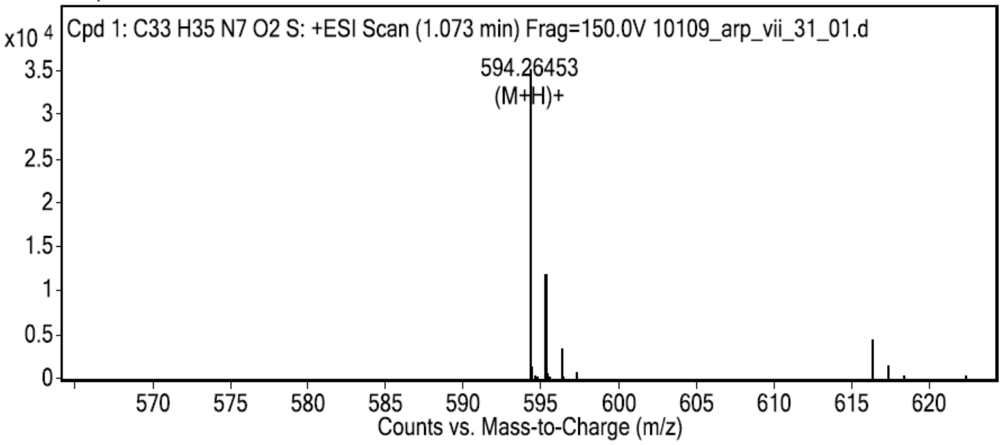

Compound 12j

Compound Table

Compound Label	RT	Mass	Abund	Formula	Tgt Mass	Diff (ppm)
Cpd 1: C24 H27 N5 O S	2.45	433.19309	37751	C24 H27 N5 O S	433.19363	-1.25

Compound Label	RT	Algorithm	Mass
Cpd 1: C24 H27 N5 O S	2.45	Find By Formula	433.19309

MS Zoomed Spectrum

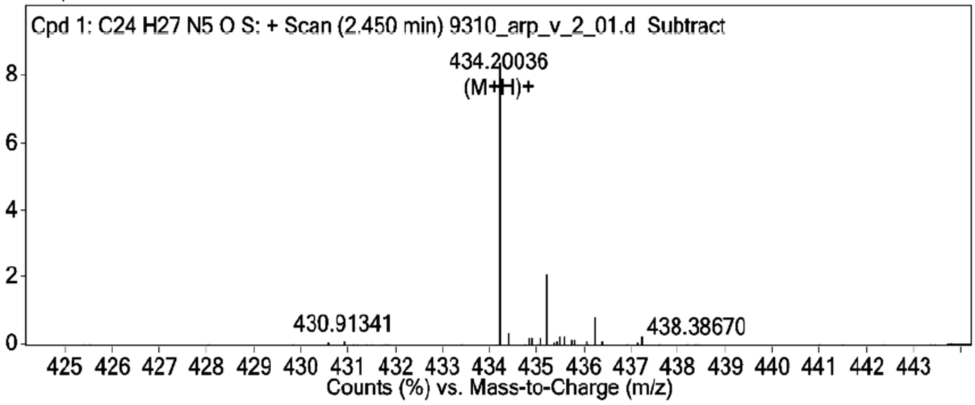

Compound 12k

Compound Table

Compound Label	RT	Mass	Abund	Formula	Tgt Mass	Diff (ppm)
Cpd 1: C30 H31 N5 O S	1.155	509.22563	191135	C30 H31 N5 O S	509.22493	1.37

Compound Label	RT	Algorithm	Mass
Cpd 1: C30 H31 N5 O S	1.155	Find By Formula	509.22563

MS Zoomed Spectrum

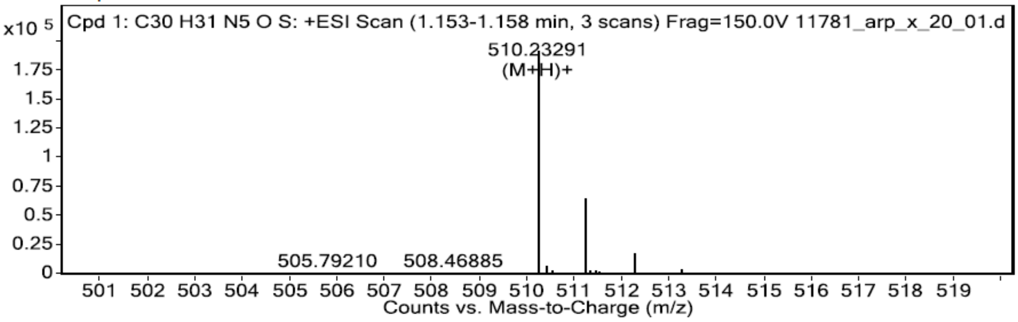

Compound 12l

Compound Table

Compound Label	RT	Mass	Abund	Formula	Tgt Mass	Diff (ppm)
Cpd 1: C23 H31 N7 O3 S	2.198	485.22033	384941	C23 H31 N7 O3 S	485.22091	-1.19

Compound Label	RT	Algorithm	Mass
Cpd 1: C23 H31 N7 O3 S	2.198	Find By Formula	485.22033

MS Zoomed Spectrum

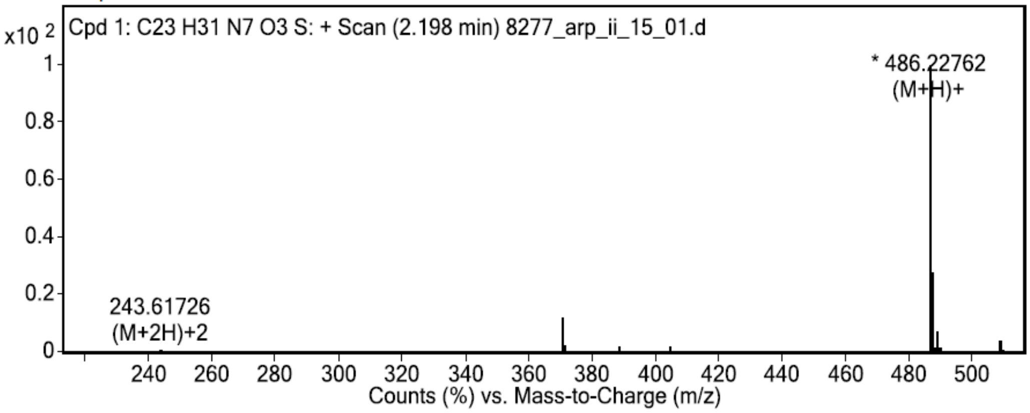

Compound 12m

Compound Table

Compound Label	RT	Mass	Abund	Formula	Tgt Mass	Diff (ppm)
Cpd 1: C25 H35 O2 N7 S	2.794	497.25721	90073	C25 H35 O2 N7 S	497.25729	-0.17

Compound Label	RT	Algorithm	Mass
Cpd 1: C25 H35 O2 N7 S	2.794	Find By Formula	497.25721

MS Zoomed Spectrum

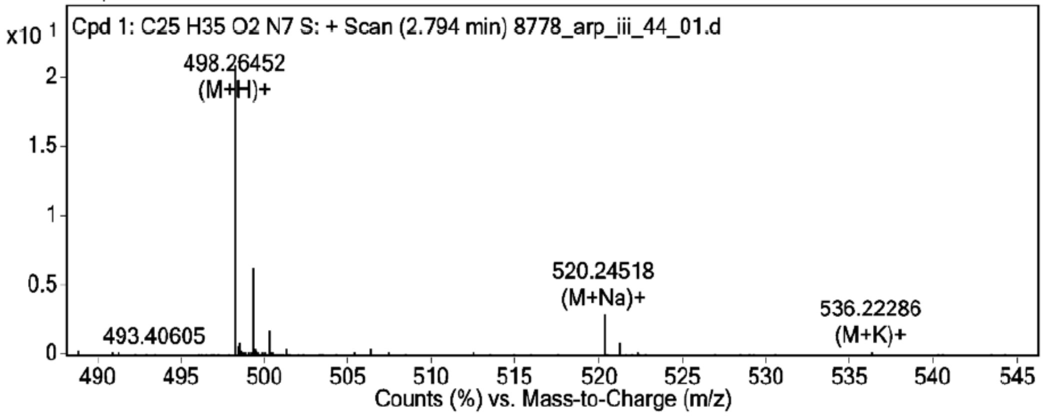

Compound 12n

Compound Table

Compound Label	RT	Mass	Abund	Formula	Tgt Mass	Diff (ppm)
Cpd 1: C26 H29 N7 O2 S	0.453	503.21001	42706	C26 H29 N7 O2 S	503.21034	-0.66

Compound Label	RT	Algorithm	Mass
Cpd 1: C26 H29 N7 O2 S	0.453	Find By Formula	503.21001

MS Zoomed Spectrum

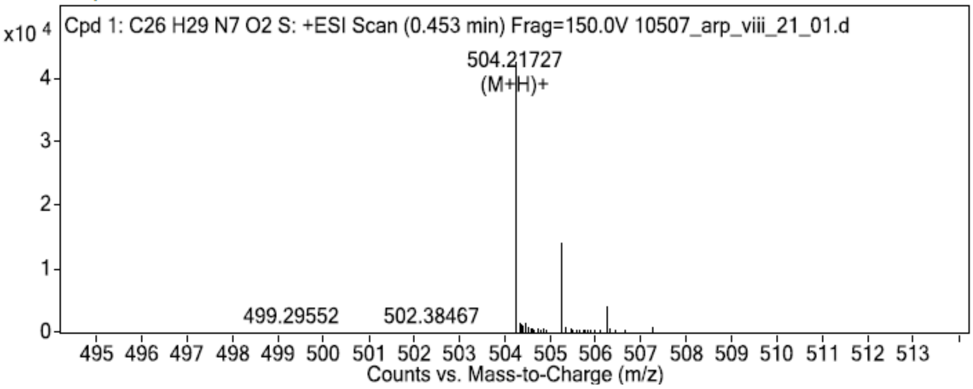

Compound 12o

Compound Table

Compound Label	RT	Mass	Abund	Formula	Tgt Mass	Diff (ppm)
Cpd 1: C27 H31 N7 O2 S	0.424	517.22564	72217	C27 H31 N7 O2 S	517.22599	-0.69

Compound Label	RT	Algorithm	Mass
Cpd 1: C27 H31 N7 O2 S	0.424	Find By Formula	517.22564

MS Zoomed Spectrum

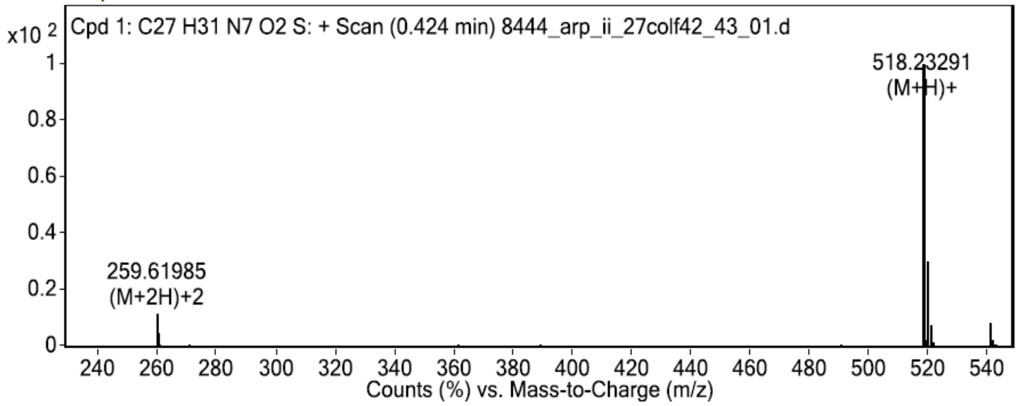

Compound 12p

Compound Table

Compound Label	RT	Mass	Abund	Formula	Tgt Mass	Diff (ppm)
Cpd 1: C29 H35 N7 O2 S	0.281	545.25731	14840	C29 H35 N7 O2 S	545.25729	0.03

Compound Label	RT	Algorithm	Mass
Cpd 1: C29 H35 N7 O2 S	0.281	Find By Formula	545.25731

MS Zoomed Spectrum

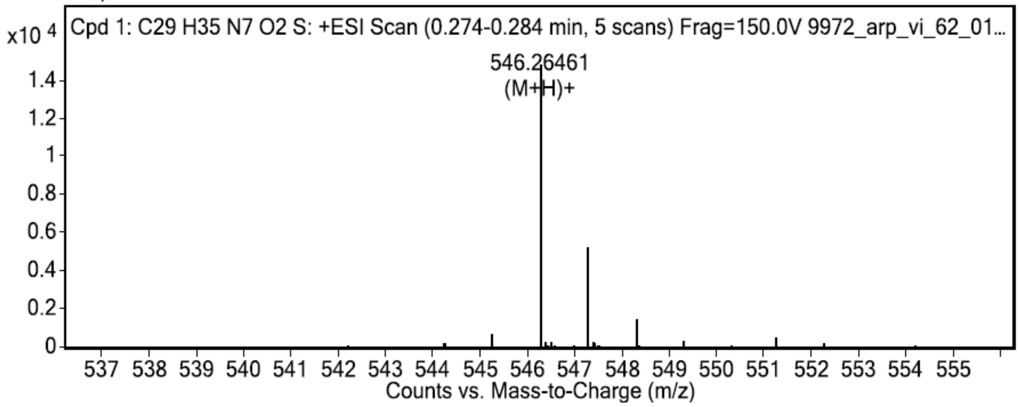

Compound 12q

Compound Table

Compound Label	RT	Mass	Abund	Formula	Tgt Mass	Diff (ppm)
Cpd 1: C31 H38 N8 O2 S	2.504	586.28595	36347	C31 H38 N8 O2 S	586.28384	3.6

Compound Label	RT	Algorithm	Mass
Cpd 1: C31 H38 N8 O2 S	2.504	Find By Formula	586.28595

MS Zoomed Spectrum

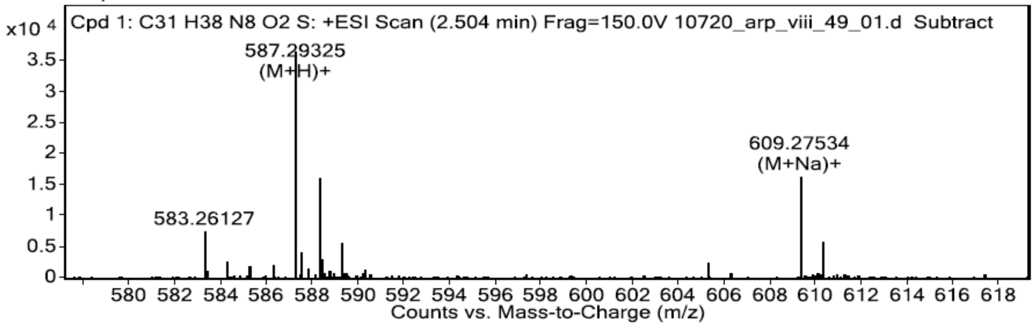

Compound 12r

Compound Table

Compound Label	RT	Mass	Abund	Formula	Tgt Mass	Diff (ppm)
Cpd 1: C31 H38 N7 O3 S	0.261	588.27651	15645	C31 H38 N7 O3 S	588.27568	1.4

Compound Label	RT	Algorithm	Mass
Cpd 1: C31 H38 N7 O3 S	0.261	Find By Formula	588.27651

MS Zoomed Spectrum

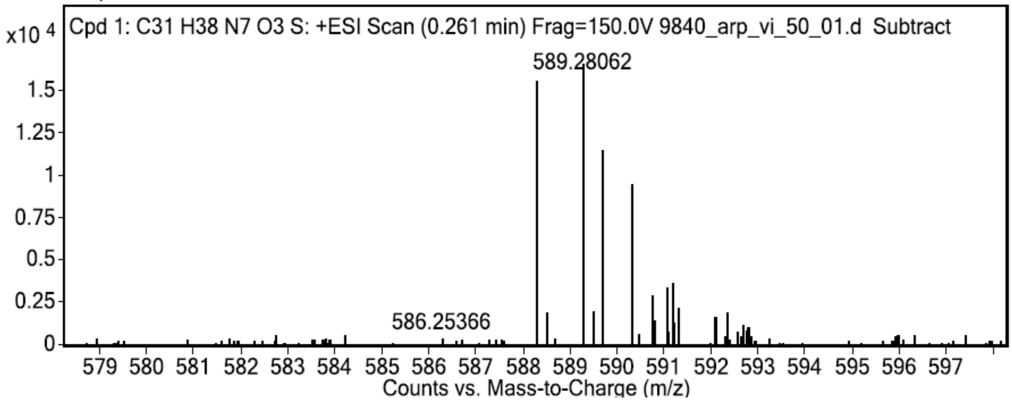

Compound 12s

Compound Table

Compound Label	RT	Mass	Abund	Formula	Tgt Mass	Diff (ppm)
Cpd 1: C34 H35 N7 O3 S	0.277	621.25172	44950	C34 H35 N7 O3 S	621.25221	-0.78

Compound Label	RT	Algorithm	Mass
Cpd 1: C34 H35 N7 O3 S	0.277	Find By Formula	621.25172

MS Zoomed Spectrum

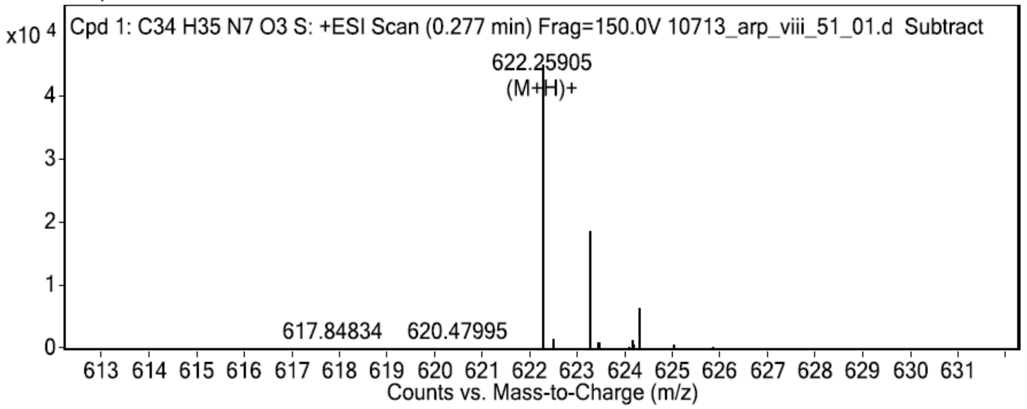

Truncated compound 13

Compound Table

Compound Label	RT	Mass	Abund	Formula	Tgt Mass	Diff (ppm)
Cpd 1: C22 H22 Br O2 N3 S	0.522	471.06223	68514	C22 H22 Br O2 N3 S	471.06161	1.32

Compound Label	RT	Algorithm	Mass
Cpd 1: C22 H22 Br O2 N3 S	0.522	Find By Formula	471.06223

MS Zoomed Spectrum

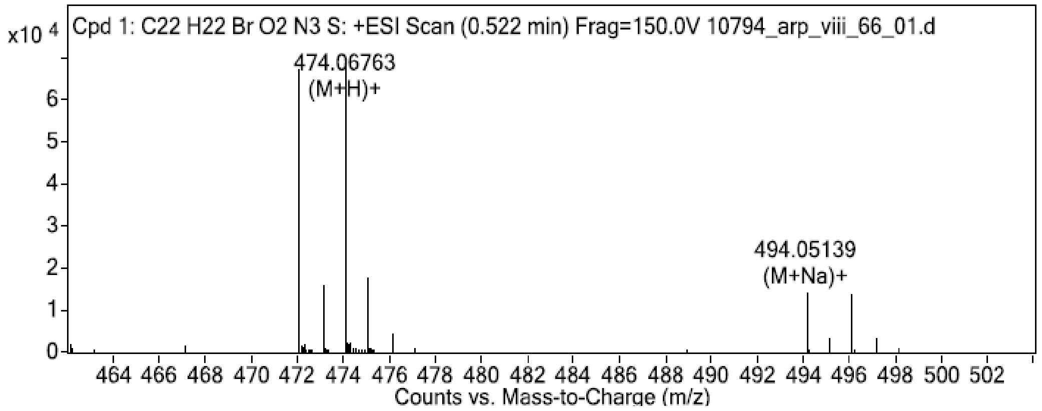

Truncated compound 14

Compound Table

Compound Label	RT	Mass	Abund	Formula	Tgt Mass	Diff (ppm)
Cpd 1: C18 H23 N7 O2	0.446	369.19035	127100	C18 H23 N7 O2	369.19132	-2.65

Compound Label	RT	Algorithm	Mass
Cpd 1: C18 H23 N7 O2	0.446	Find By Formula	369.19035

MS Zoomed Spectrum

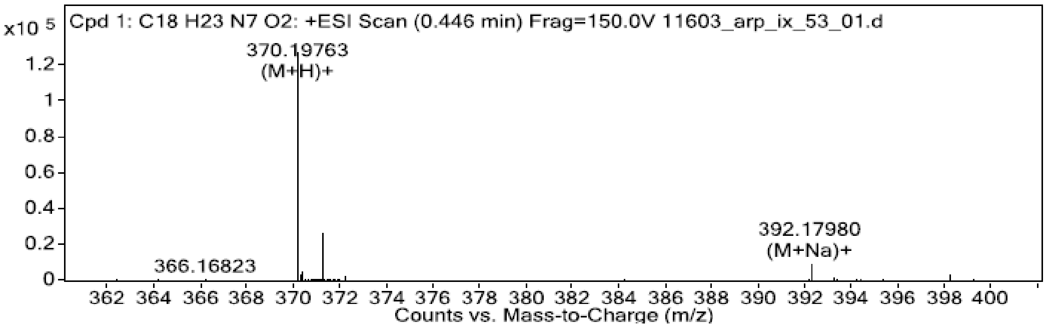

Compound 19a

Compound Table

Compound Label	RT	Mass	Abund	Formula	Tgt Mass	Diff (ppm)
Cpd 1: C32 H33 N7 O2 S	0.264	579.242	133083	C32 H33 N7 O2 S	579.24164	0.61

Compound Label	RT	Algorithm	Mass
Cpd 1: C32 H33 N7 O2 S	0.264	Find By Formula	579.242

MS Zoomed Spectrum

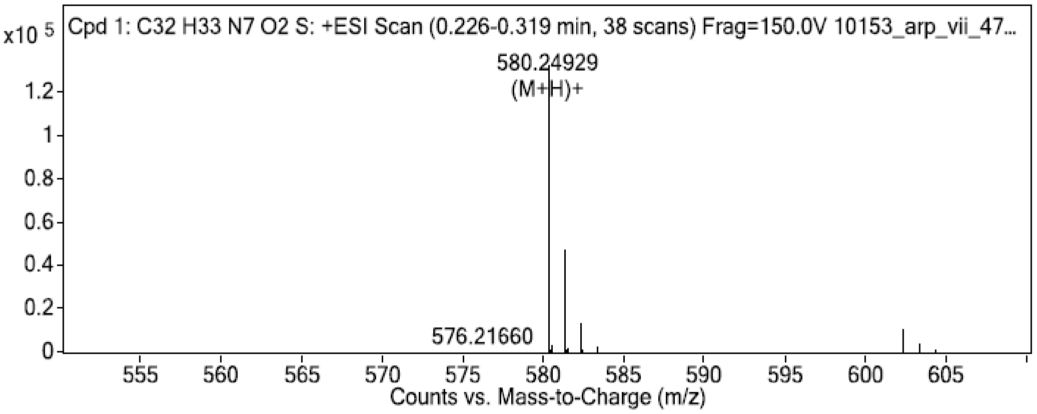

Compound 19b

Compound Table

Compound Label	RT	Mass	Abund	Formula	Tgt Mass	Diff (ppm)
Cpd 1: C38 H37 N7 O2 S	0.911	655.27482	25574	C38 H37 N7 O2 S	655.27294	2.87

Compound Label	RT	Algorithm	Mass
Cpd 1: C38 H37 N7 O2 S	0.911	Find By Formula	655.27482

MS Zoomed Spectrum

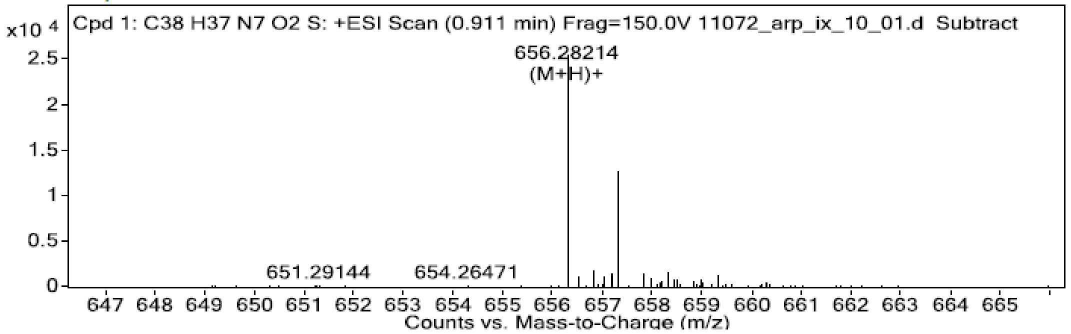

Compound 19c

Compound Table

Compound Label	RT	Mass	Abund	Formula	Tgt Mass	Diff (ppm)
Cpd 1: C38 H37 N7 O3 S	1.803	671.26731	189537	C38 H37 N7 O3 S	671.26786	-0.81

Compound Label	RT	Algorithm	Mass
Cpd 1: C38 H37 N7 O3 S	1.803	Find By Formula	671.26731

MS Zoomed Spectrum

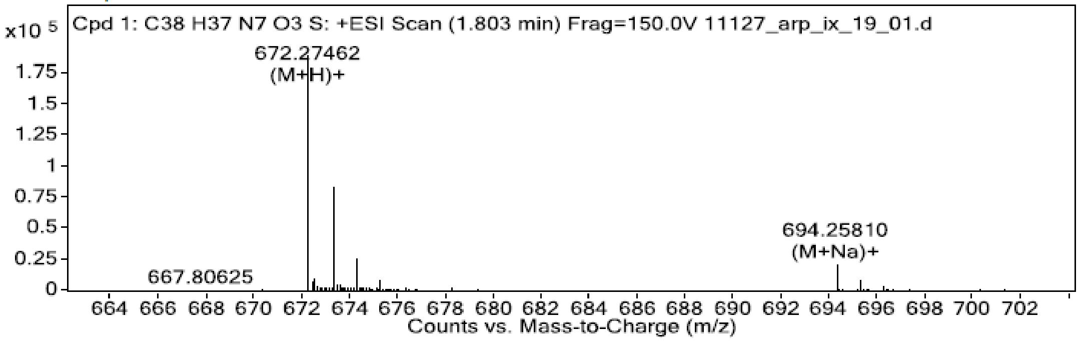

Compound 19d

Compound Table

Compound Label	RT	Mass	Abund	Formula	Tgt Mass	Diff (ppm)
Cpd 1: C40 H41 N7 O2 S	1.125	683.30497	49936	C40 H41 N7 O2 S	683.30424	1.06

Compound Label	RT	Algorithm	Mass
Cpd 1: C40 H41 N7 O2 S	1.125	Find By Formula	683.30497

MS Zoomed Spectrum

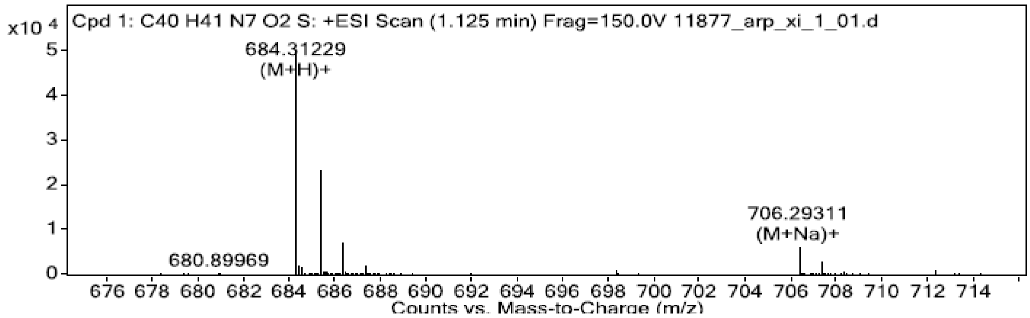

Compound 19e

Compound Table

Compound Label	RT	Mass	Abund	Formula	Tgt Mass	Diff (ppm)
Cpd 1: C40 H41 N7 O3 S	1.534	699.29928	80554	C40 H41 N7 O3 S	699.29916	0.17

Compound Label	RT	Algorithm	Mass
Cpd 1: C40 H41 N7 O3 S	1.534	Find By Formula	699.29928

MS Zoomed Spectrum

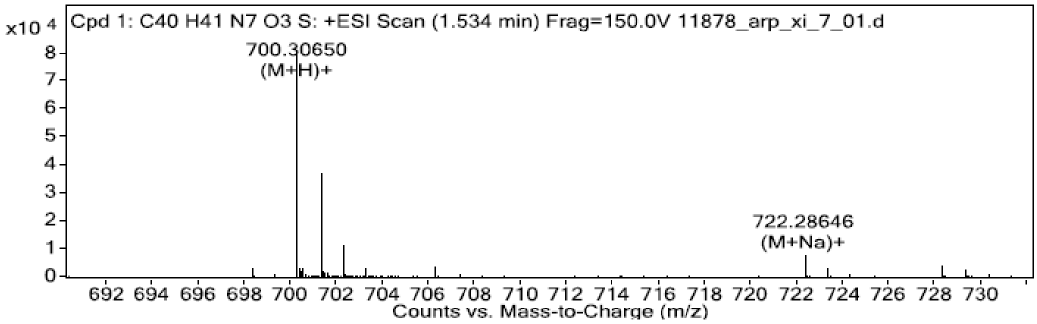

Symmetrical compound 22

Compound Table

Compound Label	RT	Mass	Abund	Formula	Tgt Mass	Diff (ppm)
Cpd 1: C46 H52 N14 O4 S2	0.244	928.37399	966	C46 H52 N14 O4 S2	928.37374	0.27

Compound Label	RT	Algorithm	Mass
Cpd 1: C46 H52 N14 O4 S2	0.244	Find By Formula	928.37399

MS Zoomed Spectrum

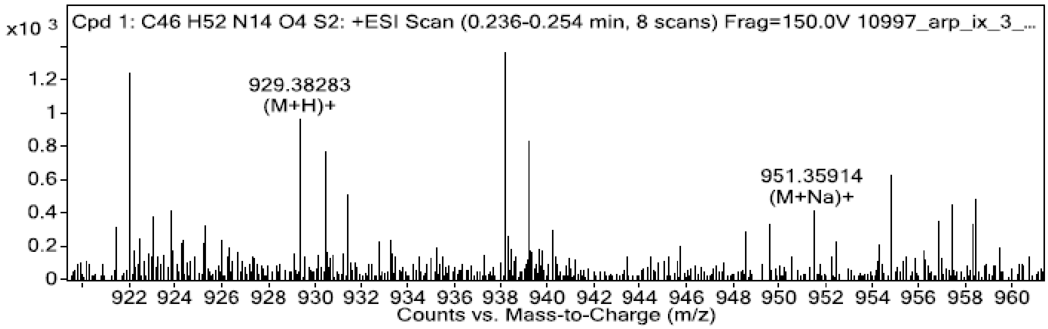

HPLC of final compounds

Compound 12a

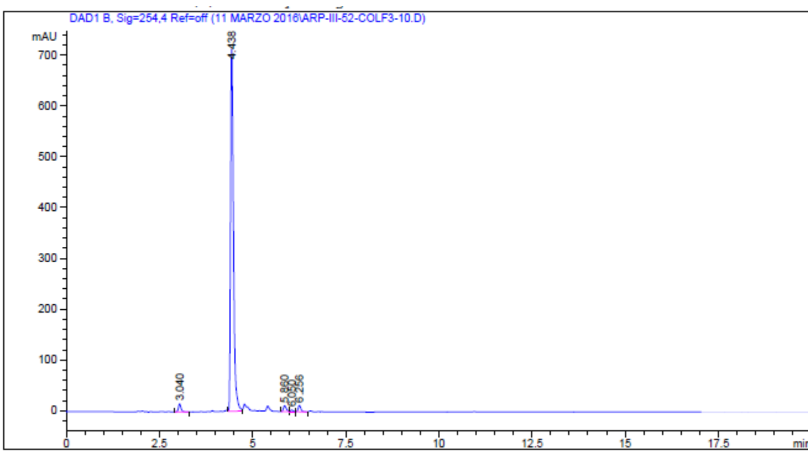


```
=====================================================================
                         Area Percent Report
=====================================================================

Sorted By             :      Signal
Multiplier:                   :      1.0000
Dilution:                     :      1.0000
Use Multiplier & Dilution Factor with ISTDs


Signal 1: DAD1 B, Sig=254,4 Ref=off
```

Peak #	RetTime [min]	Type	Width [min]	Area [mAU*s]	Height [mAU]	Area %
1	3.040	BB	0.0716	75.67520	15.88087	1.8289
2	4.438	VV	0.0860	3921.46582	714.60071	94.7730
3	5.860	BV	0.0874	65.00219	11.60204	1.5710
4	6.050	VB	0.0716	14.37953	3.01937	0.3475
5	6.256	BV	0.0727	61.22287	12.60440	1.4796

Compound 12b

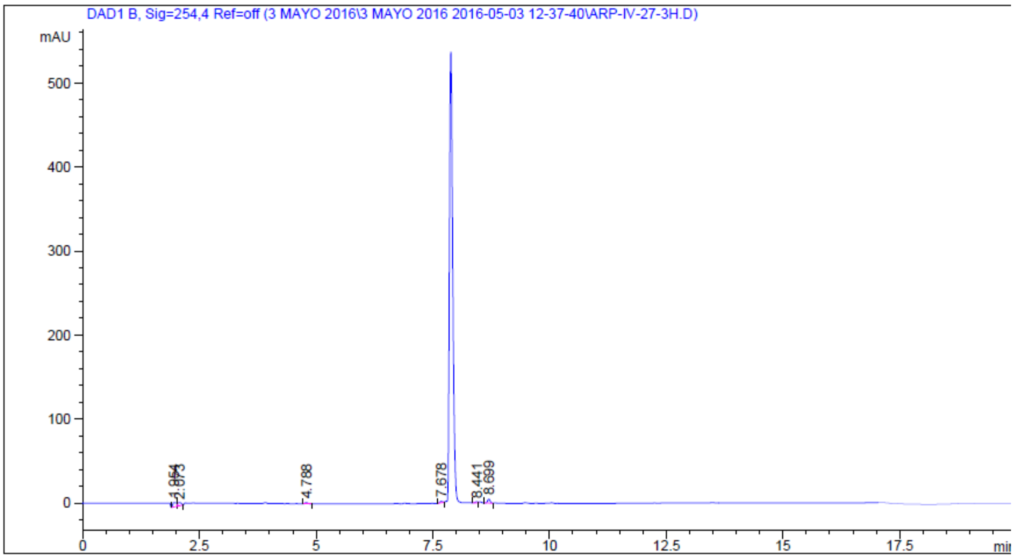


```
=====================================================================
                         Area Percent Report
=====================================================================

Sorted By             :      Signal
Multiplier:                   :      1.0000
Dilution:                     :      1.0000
```

Compound 12c

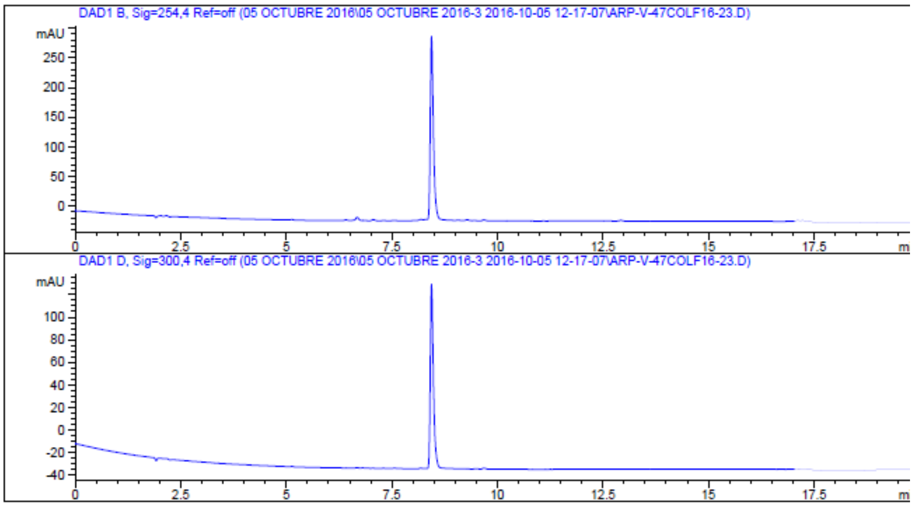


```
=====================================================================
                         Area Percent Report
=====================================================================

Sorted By              :      Signal
Multiplier:                   :       1.0000
Dilution:                     :       1.0000
Use Multiplier & Dilution Factor with ISTDs
```

Compound 12d

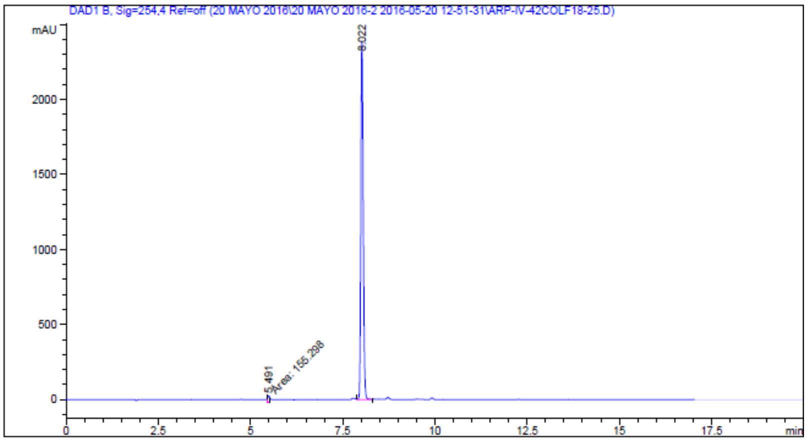


```
=====================================================================
                         Area Percent Report
=====================================================================

Sorted By              :      Signal
Multiplier:                   :       1.0000
Dilution:                     :       1.0000
Use Multiplier & Dilution Factor with ISTDs


Signal 1: DAD1 B, Sig=254,4 Ref=off

Peak RetTime Type  Width     Area      Height     Area
  #   [min]        [min]   [mAU*s]     [mAU]       %
----|-------|----|-------|----------|----------|--------|
   1   5.491 MM    0.0651  155.29810   39.76358   1.4191
   2   8.022 VV    0.0707 1.07884e4  2390.53101  98.5809
```

Compound 12e

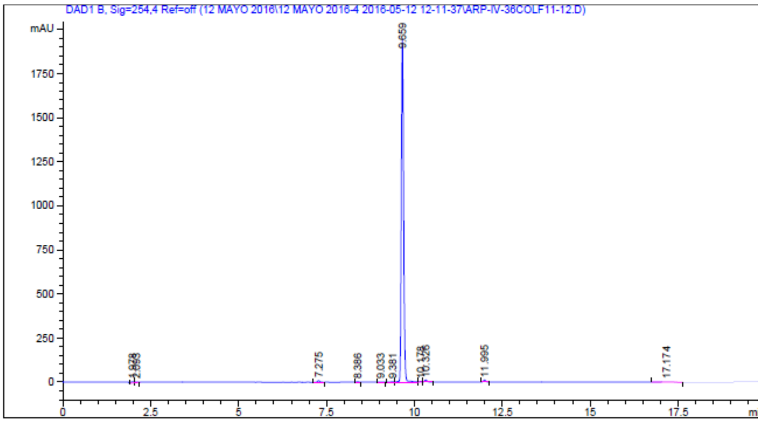


```
==================================================================
                        Area Percent Report
==================================================================

Sorted By             :      Signal
Multiplier:                   :      1.0000
Dilution:                     :      1.0000
Use Multiplier & Dilution Factor with ISTDs


Signal 1: DAD1 B, Sig=254,4 Ref=off
```

Peak #	RetTime [min]	Type	Width [min]	Area [mAU*s]	Height [mAU]	Area %
1	1.978	BV	0.1014	45.92241	6.92196	0.5068
2	2.093	VB	0.0863	27.28303	4.28348	0.3011
3	7.275	BV	0.0651	35.57291	8.45944	0.3926
4	8.386	BV	0.0638	9.26013	2.26117	0.1022
5	9.033	BB	0.0684	6.53143	1.45422	0.0721
6	9.381	BV	0.1092	17.61098	2.25389	0.1944
7	9.659	VV	0.0705	8755.60840	1946.05225	96.6310

Compound 12f

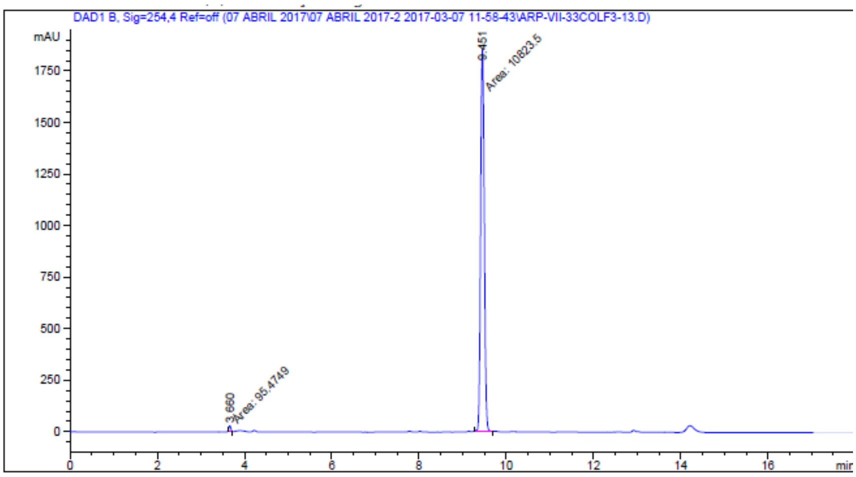


```
==================================================================
                        Area Percent Report
==================================================================

Sorted By             :      Signal
Multiplier:                   :      1.0000
Dilution:                     :      1.0000
Use Multiplier & Dilution Factor with ISTDs


Signal 1: DAD1 B, Sig=254,4 Ref=off
```

Peak #	RetTime [min]	Type	Width [min]	Area [mAU*s]	Height [mAU]	Area %
1	3.660	MM	0.0537	95.47493	29.60547	0.8744
2	9.451	MM	0.0973	1.08235e4	1854.82361	99.1256

Compound 12g

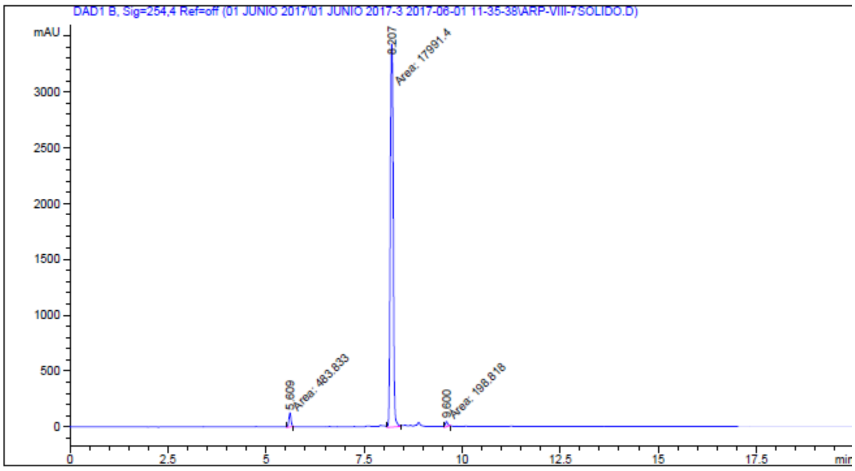

===
Area Percent Report
===

Sorted By : Signal
Multiplier: : 1.0000
Dilution: : 1.0000
Use Multiplier & Dilution Factor with ISTDs

Signal 1: DAD1 B, Sig=254,4 Ref=off

Peak #	RetTime [min]	Type	Width [min]	Area [mAU*s]	Height [mAU]	Area %
1	5.609	MM	0.0631	483.83289	127.75903	2.5909
2	8.207	MM	0.0873	1.79914e4	3433.41919	96.3444
3	9.600	MM	0.0719	198.81828	46.10819	1.0647

Compound 12h

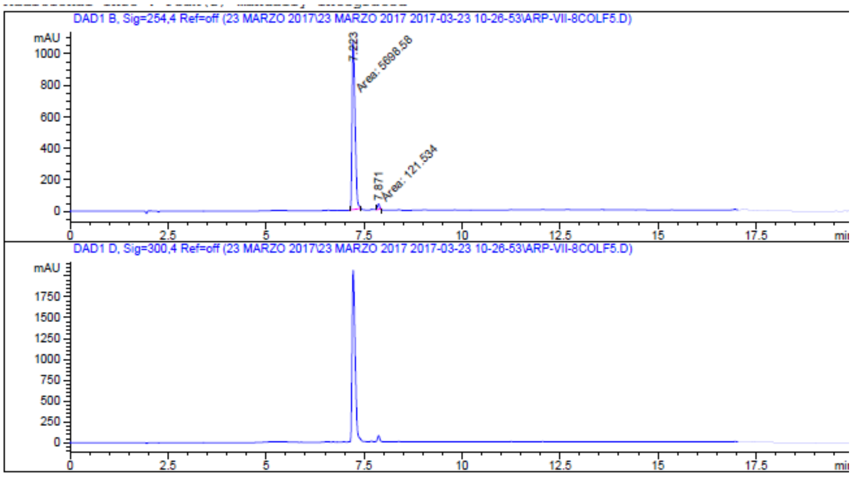

===
Area Percent Report
===

Sorted By : Signal
Multiplier: : 1.0000
Dilution: : 1.0000
Use Multiplier & Dilution Factor with ISTDs

Signal 1: DAD1 B, Sig=254,4 Ref=off

Peak #	RetTime [min]	Type	Width [min]	Area [mAU*s]	Height [mAU]	Area %
1	7.223	MM	0.0877	5698.57959	1082.70068	97.9118
2	7.871	MM	0.0566	121.53374	35.79636	2.0882

Compound 12i

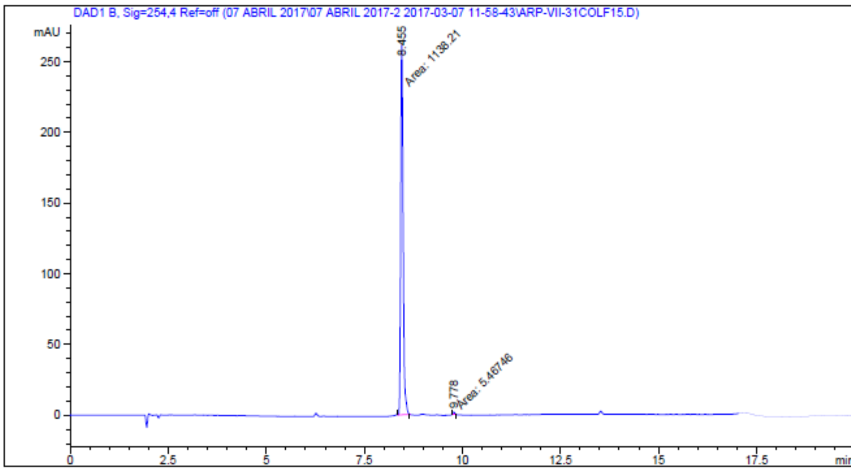


```
=====================================================================
                        Area Percent Report
=====================================================================

Sorted By             :      Signal
Multiplier:                  :      1.0000
Dilution:                    :      1.0000
Use Multiplier & Dilution Factor with ISTDs


Signal 1: DAD1 B, Sig=254,4 Ref=off

Peak RetTime Type  Width     Area      Height     Area
  #   [min]        [min]   [mAU*s]     [mAU]       %
----|-------|----|-------|----------|----------|--------|
   1   8.455 MM    0.0722 1138.21167  262.66385 99.5219
   2   9.778 MM    0.0561    5.46746    1.62535  0.4781
```

Compound 12j

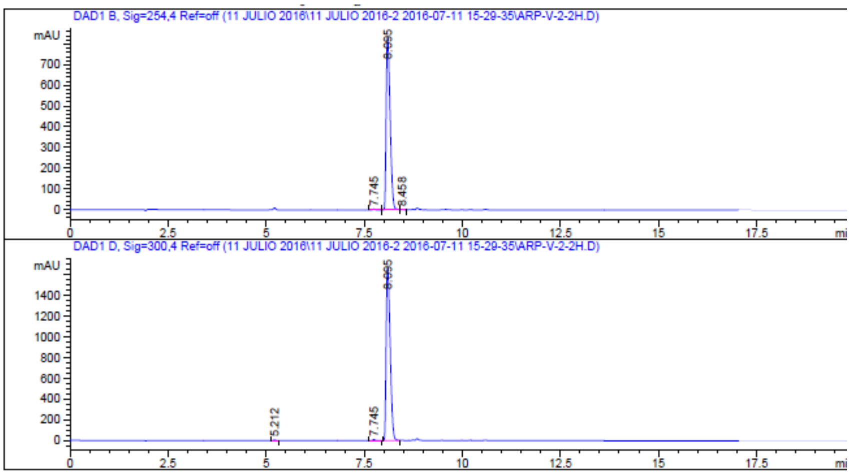


```
=====================================================================
                        Area Percent Report
=====================================================================

Sorted By             :      Signal
Multiplier:                  :      1.0000
Dilution:                    :      1.0000
Use Multiplier & Dilution Factor with ISTDs


Signal 1: DAD1 B, Sig=254,4 Ref=off

Peak RetTime Type  Width     Area      Height     Area
  #   [min]        [min]   [mAU*s]     [mAU]       %
----|-------|----|-------|----------|----------|--------|
   1   7.745 BB    0.0674   18.37225    4.17380  0.3143
   2   8.095 BV    0.1073 5821.30859  835.54053 99.5890
   3   8.458 VB    0.0718    5.65452    1.18208  0.0967
```

Compound 12k

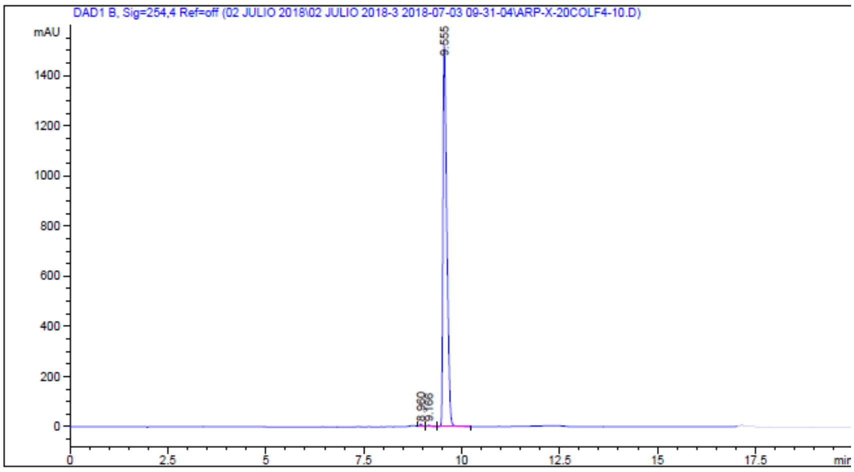


```
=====================================================================
                         Area Percent Report
=====================================================================

Sorted By             :      Signal
Multiplier:                   :      1.0000
Dilution:                     :      1.0000
Use Multiplier & Dilution Factor with ISTDs


Signal 1: DAD1 B, Sig=254,4 Ref=off

Peak RetTime Type  Width     Area       Height     Area
  #   [min]         [min]   [mAU*s]     [mAU]        %
----|-------|----|-------|----------|----------|--------|
   1   8.960 VV     0.0699   50.10977   10.46631   0.4763
   2   9.166 VB     0.0750   29.45800    6.03150   0.2800
   3   9.555 BB     0.1019 1.04420e4  1524.55652  99.2438
```

Compound 12l

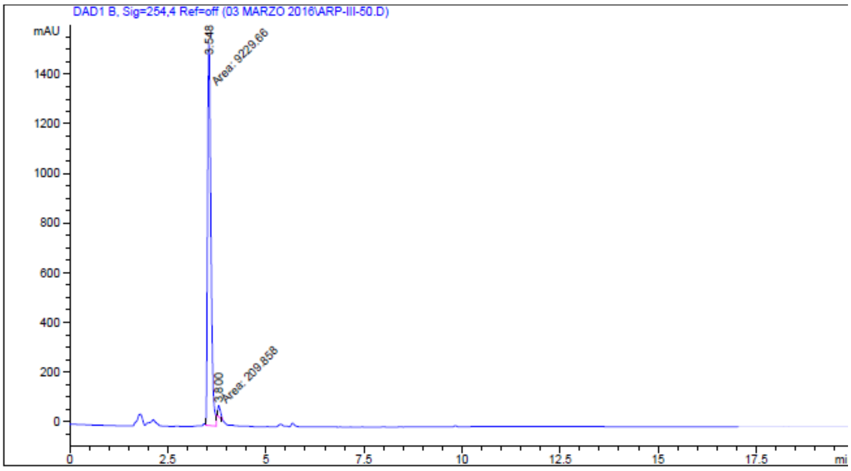


```
=====================================================================
                         Area Percent Report
=====================================================================

Sorted By             :      Signal
Multiplier:                   :      1.0000
Dilution:                     :      1.0000
Use Multiplier & Dilution Factor with ISTDs


Signal 1: DAD1 B, Sig=254,4 Ref=off

Peak RetTime Type  Width     Area       Height     Area
  #   [min]         [min]   [mAU*s]     [mAU]        %
----|-------|----|-------|----------|----------|--------|
   1   3.548 MM     0.0999 9229.65918 1539.14612  97.7768
   2   3.800 MM     0.0787  209.85815   44.45146   2.2232
```

Compound 12m

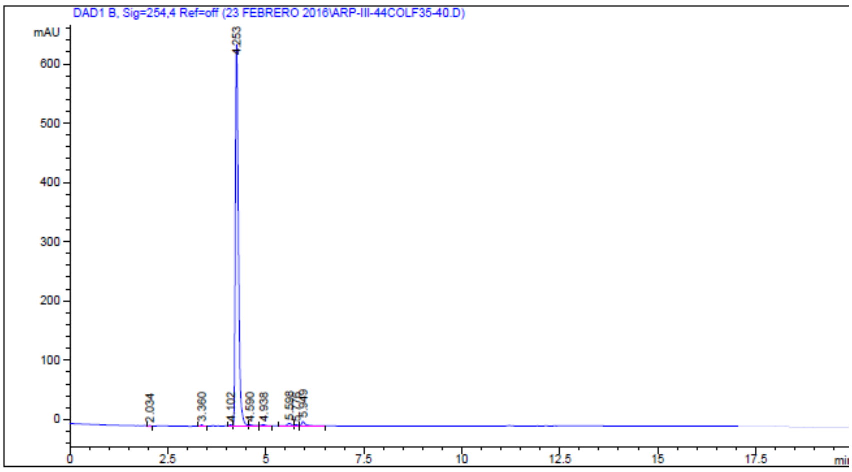


```
==================================================================
                        Area Percent Report
==================================================================

Sorted By              :      Signal
Multiplier:                  :      1.0000
Dilution:                    :      1.0000
Use Multiplier & Dilution Factor with ISTDs


Signal 1: DAD1 B, Sig=254,4 Ref=off

Peak RetTime Type  Width     Area      Height     Area
  #   [min]        [min]   [mAU*s]     [mAU]        %
----|-------|----|-------|----------|----------|--------|
   1   2.034 VB    0.0835    8.47808    1.55815   0.2304
   2   3.360 BB    0.0620   10.98859    2.66981   0.2986
   3   4.102 BV    0.0662    8.66213    1.93698   0.2354
   4   4.253 VV    0.0858 3520.85449  643.70288  95.6801
```

Compound 12n

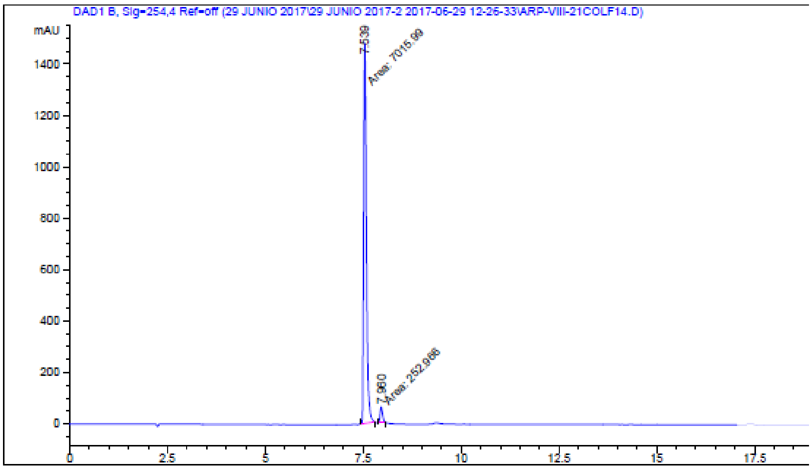


```
==================================================================
                        Area Percent Report
==================================================================

Sorted By              :      Signal
Multiplier:                  :      1.0000
Dilution:                    :      1.0000
Use Multiplier & Dilution Factor with ISTDs


Signal 1: DAD1 B, Sig=254,4 Ref=off

Peak RetTime Type  Width     Area      Height     Area
  #   [min]        [min]   [mAU*s]     [mAU]        %
----|-------|----|-------|----------|----------|--------|
   1   7.539 MM    0.0789 7015.99219 1481.90625  96.5199
   2   7.960 MM    0.0693  252.96605   60.79996   3.4801
```

Compound 12o

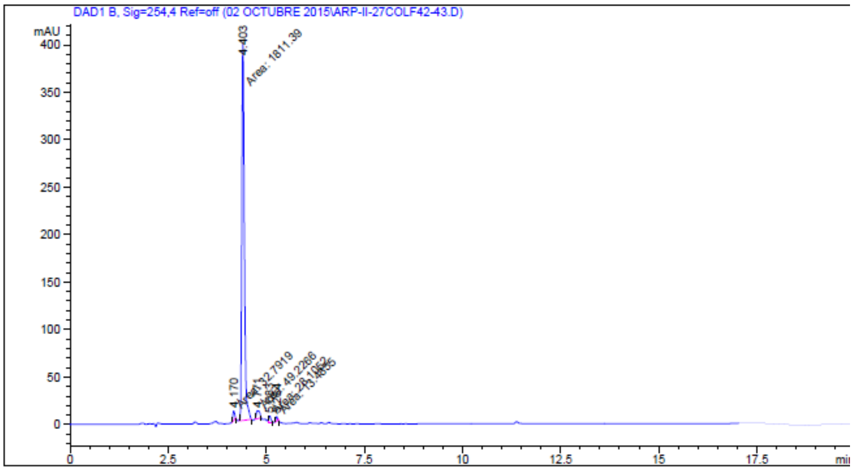

===
Area Percent Report
===

Sorted By : Signal
Multiplier: : 1.0000
Dilution: : 1.0000
Use Multiplier & Dilution Factor with ISTDs

Signal 1: DAD1 B, Sig=254,4 Ref=off

Peak #	RetTime [min]	Type	Width [min]	Area [mAU*s]	Height [mAU]	Area %
1	4.170	MM	0.0531	32.79185	10.29895	1.6947
2	4.403	MM	0.0756	1811.39087	399.45956	93.6119

Compound 12p

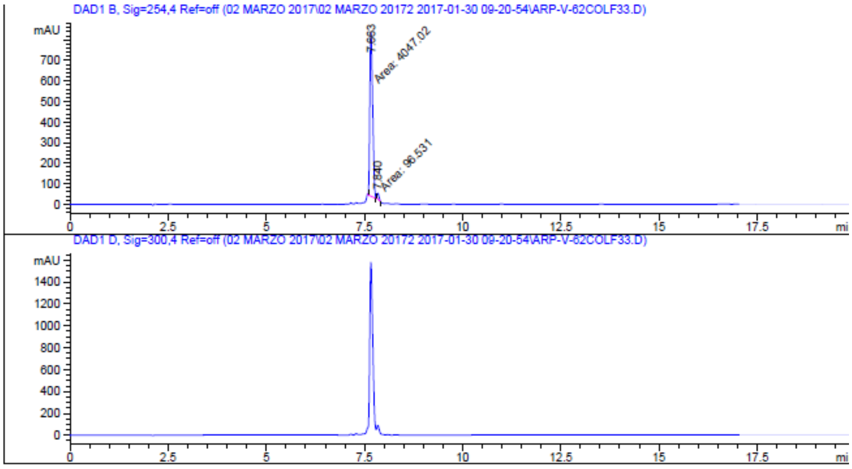

===
Area Percent Report
===

Sorted By : Signal
Multiplier: : 1.0000
Dilution: : 1.0000
Use Multiplier & Dilution Factor with ISTDs

Signal 1: DAD1 B, Sig=254,4 Ref=off

Peak #	RetTime [min]	Type	Width [min]	Area [mAU*s]	Height [mAU]	Area %
1	7.663	MM	0.0839	4047.01660	803.92444	97.6703
2	7.840	MM	0.0565	96.53104	28.46407	2.3297

Compound 12q

Method Set F035_MS_G2_30_t10	Sample Set Name 18_09_2017
Injection Volume 2.00 ul	Date Acquired 9/18/17 12:52:47 PM
Vial 4	Channel Description PDA MaxPlot (230.0 nm to 400.0 nm)
Flow: 1 ml/min	Column: SunFire C18 3.5um (4.6x 50 mm)
Run Time 18.00 Minutes	

Auto-Scaled Chromatogram

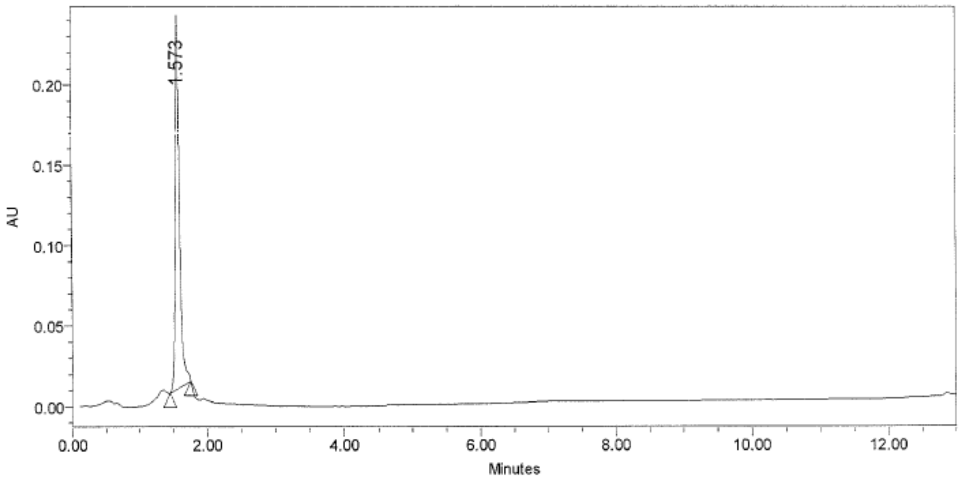

Peak Results

	RT	% Area
1	1.573	100.00

Compound 12r

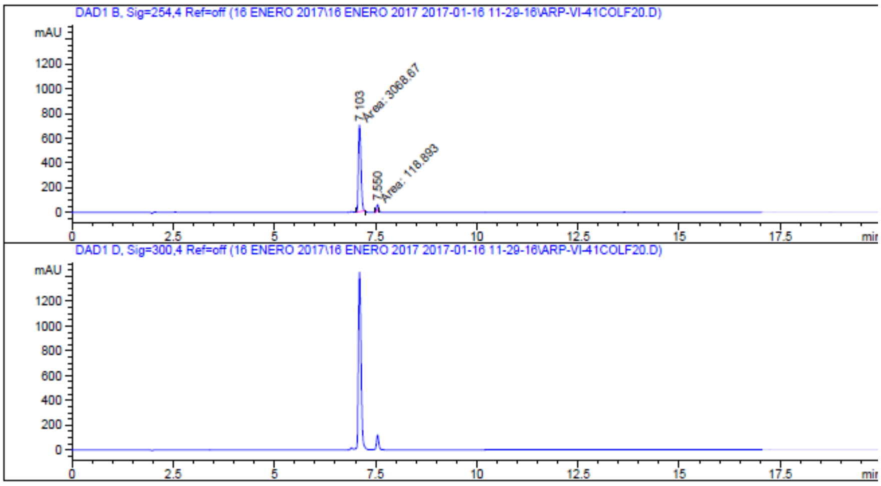


```
=====================================================================
                         Area Percent Report
=====================================================================

Sorted By             :      Signal
Multiplier:                  :      1.0000
Dilution:                    :      1.0000
Use Multiplier & Dilution Factor with ISTDs


Signal 1: DAD1 B, Sig=254,4 Ref=off

Peak RetTime Type  Width     Area      Height     Area
  #   [min]        [min]   [mAU*s]     [mAU]       %
----|-------|----|-------|----------|----------|--------|
   1   7.103 MM    0.0726 3068.66968  704.58185  96.2701
   2   7.550 MM    0.0458  118.89349   43.22698   3.7299
```

Compound 12s

Method Set F035_MS_G10_95_t10
Injection Volume 10.00 ul
Vial 7
Flow: 1 ml/min
Run Time 18.00 Minutes

Sample Set Name 19_09_2017
Date Acquired 9/19/17 12:47:15 PM
Channel Description PDA MaxPlot (230.0 nm to 400.0 nm)
Column: SunFire C18 3.5um (4.6x 50 mm)

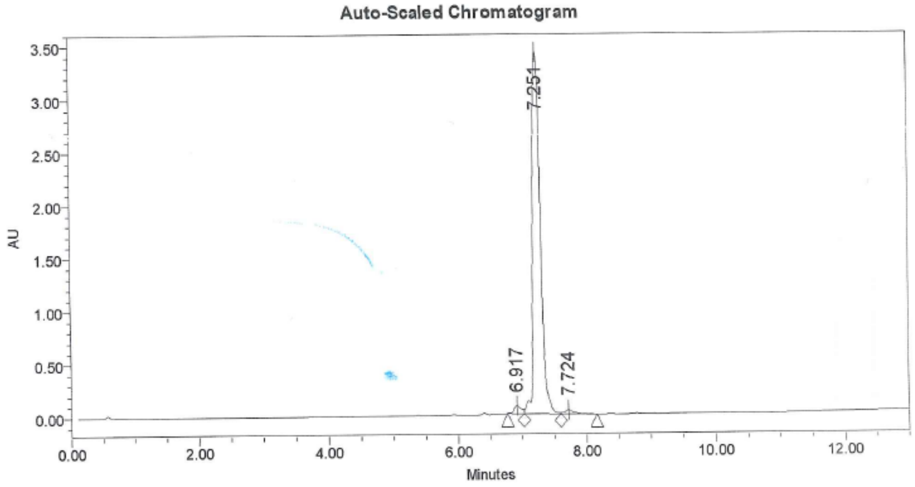

Peak Results

	RT	% Area
1	6.917	1.90
2	7.251	96.91
3	7.724	1.19

Truncated compound 13

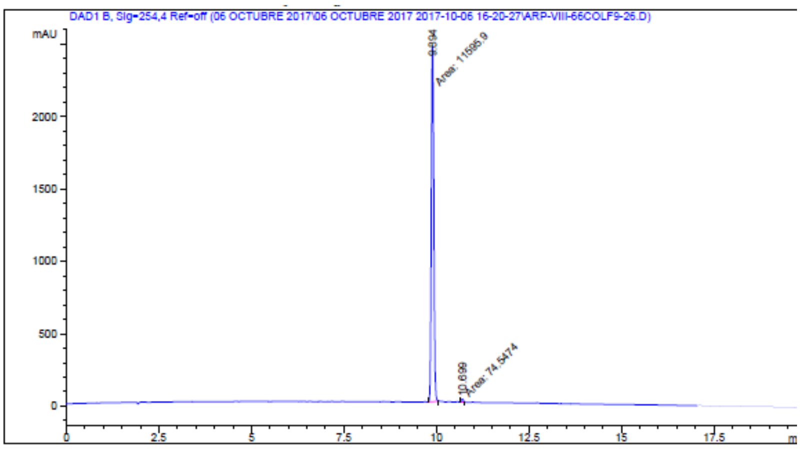


```
=====================================================================
                         Area Percent Report
=====================================================================

Sorted By             :      Signal
Multiplier:                  :      1.0000
Dilution:                    :      1.0000
Use Multiplier & Dilution Factor with ISTDs

Signal 1: DAD1 B, Sig=254,4 Ref=off

Peak RetTime Type  Width     Area      Height     Area
  #   [min]        [min]   [mAU*s]     [mAU]       %
----|-------|----|-------|----------|----------|--------|
   1   9.894 MM    0.0787 1.15959e4  2455.72168  99.3612
   2  10.699 MM    0.0622   74.54743   19.96870   0.6388
```

Truncated compound 14

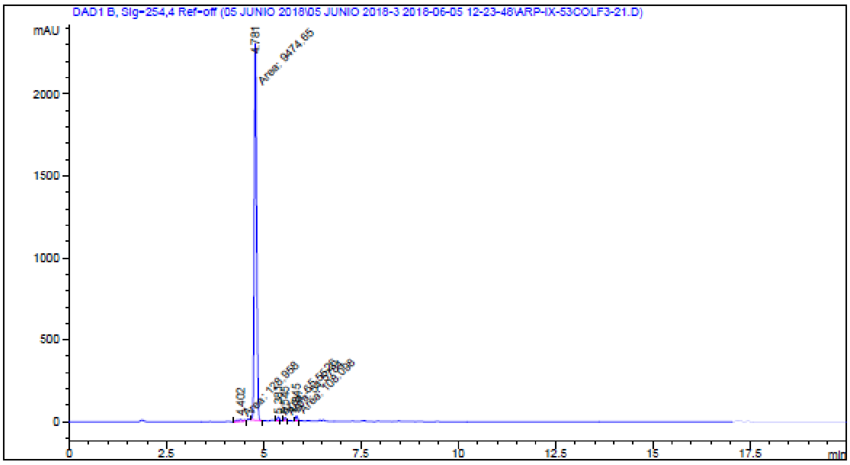


```
------------------------------------------------------------------
                    Area Percent Report
------------------------------------------------------------------

Sorted By             :      Signal
Multiplier:                  :      1.0000
Dilution:                    :      1.0000
Use Multiplier & Dilution Factor with ISTDs

Signal 1: DAD1 B, Sig=254,4 Ref=off

Peak RetTime Type  Width     Area      Height     Area
  #   [min]        [min]   [mAU*s]     [mAU]        %
----|-------|----|-------|----------|----------|--------|
   1   4.402 MM    0.1770  128.95772   12.14579   1.3116
   2   4.781 MM    0.0684 9474.64648 2307.45044  96.3670
   3   5.381 MM    0.0551   65.55256   19.83049   0.6667
   4   5.545 MM    0.0562   54.57840   16.18933   0.5551
   5   5.845 MM    0.0615  108.09762   29.29767   1.0995
```

Compound 19a

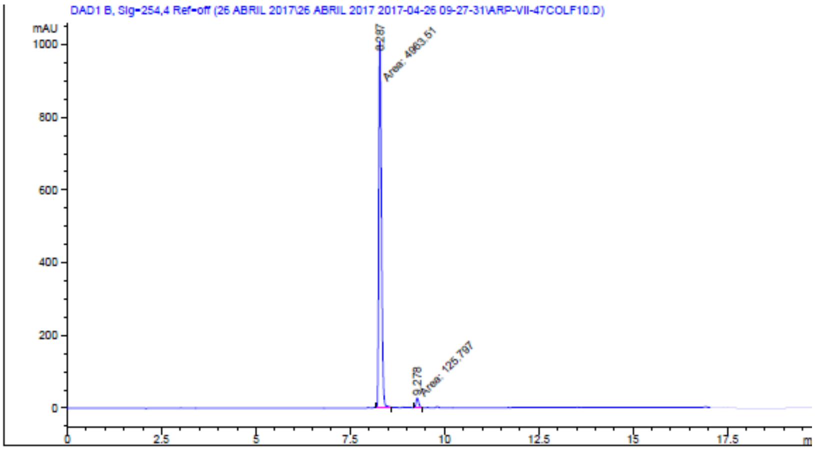


```
------------------------------------------------------------------
                    Area Percent Report
------------------------------------------------------------------

Sorted By             :      Signal
Multiplier:                  :      1.0000
Dilution:                    :      1.0000
Use Multiplier & Dilution Factor with ISTDs

Signal 1: DAD1 B, Sig=254,4 Ref=off

Peak RetTime Type  Width     Area      Height     Area
  #   [min]        [min]   [mAU*s]     [mAU]        %
----|-------|----|-------|----------|----------|--------|
   1   8.287 MM    0.0819 4963.51465 1009.98993  97.5282
   2   9.278 MM    0.0792  125.79665   26.47345   2.4718
```

Compound 19b

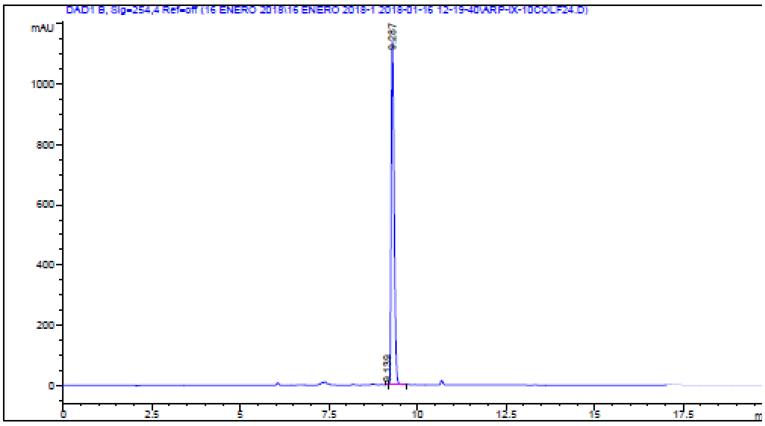

Area Percent Report

Sorted By : Signal
Multiplier: : 1.0000
Dilution: : 1.0000
Use Multiplier & Dilution Factor with ISTDs

Signal 1: DAD1 B, Sig=254,4 Ref=off

Peak #	RetTime [min]	Type	Width [min]	Area [mAU*s]	Height [mAU]	Area %
1	9.139	BV	0.0593	5.19366	1.33868	0.0775
2	9.287	VV	0.0922	6695.38770	1146.08337	99.9225

Compound 19c

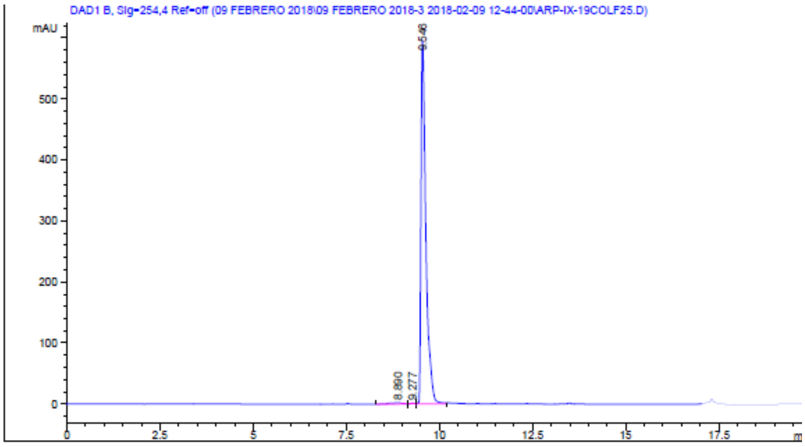

Area Percent Report

Sorted By : Signal
Multiplier: : 1.0000
Dilution: : 1.0000
Use Multiplier & Dilution Factor with ISTDs

Signal 1: DAD1 B, Sig=254,4 Ref=off

Peak #	RetTime [min]	Type	Width [min]	Area [mAU*s]	Height [mAU]	Area %
1	8.890	BB	0.2872	45.34130	2.08939	0.8287
2	9.277	BV	0.0848	6.47058	1.16539	0.1183
3	9.546	VV	0.1343	5419.44727	595.07318	99.0530

Compound 19c

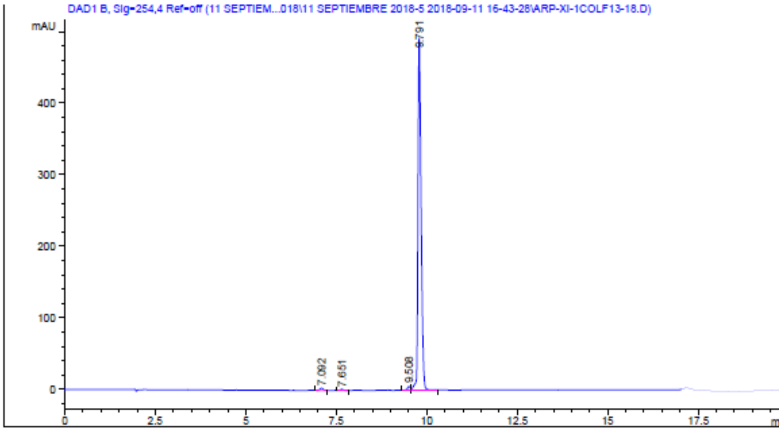

Area Percent Report

Sorted By : Signal
Multiplier: : 1.0000
Dilution: : 1.0000
Use Multiplier & Dilution Factor with ISTDs

Signal 1: DAD1 B, Sig=254,4 Ref=off

Peak #	RetTime [min]	Type	Width [min]	Area [mAU*s]	Height [mAU]	Area %
1	7.092	BB	0.0891	18.41888	3.11199	0.6108
2	7.651	BB	0.0710	8.04033	1.70545	0.2666
3	9.508	BV	0.0840	30.52392	5.39920	1.0123
4	9.791	VB	0.0900	2958.42798	492.99564	98.1103

Compound 19d

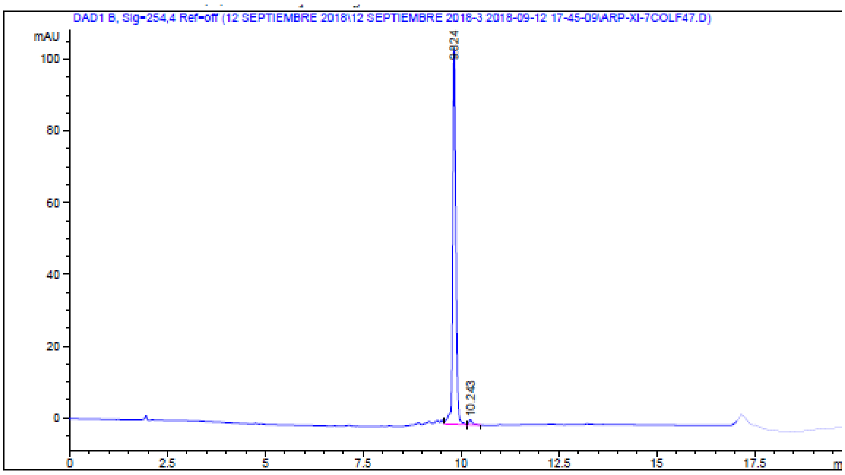

Area Percent Report

Sorted By : Signal
Multiplier: : 1.0000
Dilution: : 1.0000
Use Multiplier & Dilution Factor with ISTDs

Signal 1: DAD1 B, Sig=254,4 Ref=off

Peak #	RetTime [min]	Type	Width [min]	Area [mAU*s]	Height [mAU]	Area %
1	9.824	VB	0.0809	564.22882	104.64286	98.6153
2	10.243	BB	0.0842	7.92274	1.39584	1.3847

Symmetrical compound 22

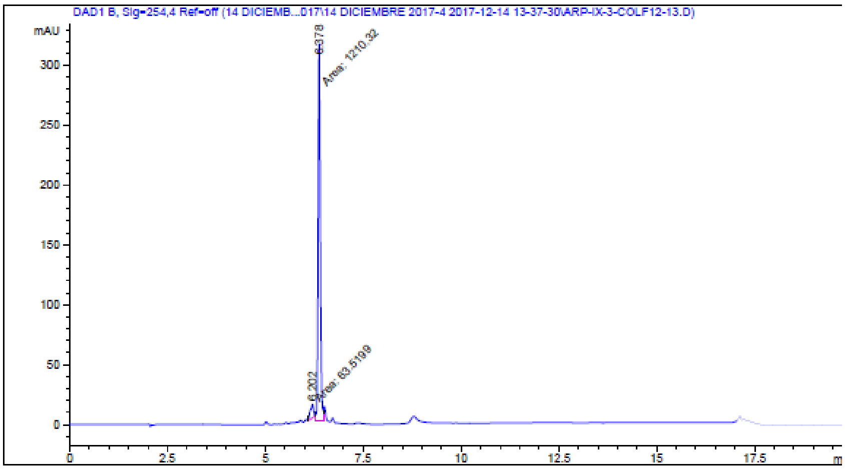


```
=====================================================================
                         Area Percent Report
=====================================================================

Sorted By             :      Signal
Multiplier:                   :      1.0000
Dilution:                     :      1.0000
Use Multiplier & Dilution Factor with ISTDs


Signal 1: DAD1 B, Sig=254,4 Ref=off

Peak RetTime Type  Width     Area      Height     Area
  #   [min]        [min]   [mAU*s]     [mAU]        %
----|-------|----|-------|----------|----------|--------|
   1   6.202 MM    0.0930   63.51986   11.37742   4.9865
   2   6.378 MM    0.0636 1210.32104  317.31894  95.0135
```

REFERENCES

(1) Revuelto, A.; Ruiz-Santaquiteria, M.; De Lucio, H.; Gamo, A.; Carriles, A. A.; Gutiérrez, K. J.; Sánchez-Murcia, P. A.; Hermoso, J. A.; Gago, F.; Camarasa, M. J.; Jiménez-Ruiz, A.; Velázquez, S. Pyrrolopyrimidine *vs* imidazole-phenyl-thiazole scaffolds in nonpeptidic dimerization inhibitors of *Leishmania infantum* trypanothione reductase. *ACS Infect. Dis.* **2019,** *5*, 873-891.

(2) Leskovac, V. Comprehensive enzyme kinetics; Kluwer Academic/Plenum Publishers: New York, 2003.

(3) Copeland, R. A. Enzymes: A practical introduction to structure, mechanism, and data analysis; 2nd ed.; John Wiley & Sons, Inc.: New York, 2000.

(4) Copeland, R. A. Evaluation of enzyme inhibitors in drug discovery: A guide for medicinal chemists and pharmacologists; 2nd ed.; John Wiley & Sons, Inc.: Hoboken, NJ, 2013.

(5) de Lucio, H.; Toro, M. A.; Camarasa, M. J.; Velázquez, S.; Gago, F.; Jiménez-Ruiz, A. Pseudoirreversible slow-binding inhibition of trypanothione reductase by a protein-protein interaction disruptor. *Br. J. Pharmacol*. **2020**, *177*, 5163-5176.

(6) Klett, J.; Núñez-Salgado, A.; Dos Santos, H. G.; Cortés-Cabrera, A.; Perona, A.; Gil-Redondo, R.; Abia, D.; Gago, F.; Morreale, A. MM-ISMSA: An ultrafast and accurate scoring function for protein-protein docking. *J. Chem. Theory Comput*. **2012**, *8*, 3395-408.

(7) Bond, C. S.; Zhang, Y.; Berriman, M.; Cunningham, M. L,; Fairlamb, A. H.; Hunter, W. N. Crystal structure of Trypanosoma cruzi trypanothione reductase in complex with trypanothione, and the structure-based discovery of new natural product inhibitors. *Structure*. **1999,** *7*, 81-89.
